# A New Prognostic Indicator of Immune Microenvironment and Therapeutic Response in Lung Adenocarcinoma Based on Peroxisome-Related Genes

**DOI:** 10.1155/2022/6084589

**Published:** 2022-07-26

**Authors:** Zhen Xiong, Liwen Zhang, Wei Fan

**Affiliations:** ^1^Department of Infection, Chongqing Red Cross Hospital, Chongqing, China; ^2^The Fifth Department of Tuberculosis, Chongqing Public Health Medical Center, Chongqing, China; ^3^Department of Pulmonary and Critical Care Medicine, The First Affiliated Hospital of Army Medical University, Chongqing, China

## Abstract

Lung adenocarcinoma (LUAD) has been the major cause of tumor-associated mortality in recent years and exhibits a poor outcome. New data revealed that peroxisomes have a function in the regulation of the development and progression of several tumors. However, the prognostic values of peroxisome-related genes (PRGs) were rarely reported. Genomic sequence, mutation, and clinical data of 535 LUAD tissues were obtained from TCGA data sets. Within the TCGA cohort, a multigene signature was constructed with the assistance of the LASSO Cox regression model. Three GEO data sets, including GSE3141, GSE31210, and GSE72094, were obtained as validation cohorts. ROC assays, Kaplan-Meier methods, and multivariate assays were applied to examine the prognostic capacities of the novel signature. Gene Set Enrichment Analysis (GSEA) was performed to further understand the underlying molecular mechanisms. In this study, we identified 47 differentially expressed peroxisome-related genes (PRGs), including 25 increased and 22 decreased PRGs. A prognostic model of six PRGs was established. The univariate and multivariate Cox analyses both showed that the *p* value of risk score was less than 0.05. In LUAD patients, the strong connection between the risk score and overall survival was further verified in three other GEO data sets. TMB and cancer stem cell infiltration were shown to be significantly higher in the high-risk group in comparison to the low-risk group. The TIDE score of the group with the low risk was considerably greater than that of the group with the high risk. Several drugs, targeting PRG-related genes, were available for the treatments of LUAD. Overall, we developed a novel peroxisome-related prognostic signature for LUAD patients. This signature could successfully indicate LUAD patients' chances of survival as well as their immune system's responsiveness to treatments. In addition, it has the potential to serve as immunotherapeutic targets for LUAD patients.

## 1. Introduction

Lung carcinoma is one of malignant cancers with the highest incidence and the worst prognosis worldwide [1]. Among all lung carcinoma subtypes, lung adenocarcinoma (LUAD) is the most common and aggressive subgroup [2]. Despite recent breakthroughs in the diagnosis and treatment of malignancies, the clinical outcome for LUAD remains unsatisfactory [3, 4]. Tumor excision, for example, can result in systemic inflammation and the release of cancer cells into the bloodstream, both of which aid cancer dissemination [5, 6]. In this regard, a thorough knowledge of the potential mechanism underlying LUAD formation and progression is essential to enhance diagnosis and prognosis at the beginning.

Peroxisomes are metabolic organelles which play an important role in cellular redox balance and lipid metabolism [7]. Peroxisomal functions are very critical to reactive oxygen species homeostasis, bile acid synthesis, ether phospholipid synthesis, and fatty acid oxidation [8, 9]. The regular functioning of cells depends on the peroxisome's free radical production and scavenging process being in equilibrium [10]. Peroxisomal disorders can be caused by mutations in genes coding for peroxisomal proteins, many of which demonstrate metabolic abnormalities [11, 12]. Peroxisomes have recently been implicated in the formation and progression of a variety of cancers, including LUAD, according to recent researches [13, 14]. In LUAD and other malignancies, a number of peroxisomal enzymes and metabolic processes are changed. Therefore, an in-depth understanding of the peroxisome process in LUAD could provide an important solution for the development of a new treatment method. Gene chips and high-throughput sequencing technology have advanced significantly in the last few years, indicating that the peroxisome's genetic signature can be utilized to predict LUAD's overall survival [15, 16].

In this study, we identified the differentially expressed peroxisome-related genes (PRGs) between LUAD specimens and nontumor specimens. Then, using univariate and least absolute shrinkage and selection operator (LASSO) Cox regression analysis, we established a unique predictive signature from PRGs in LUAD cohorts. Moreover, we confirmed the prognostic value of the novel model and its association with immune cell infiltration. On the basis of our findings, we suggested that this novel signature could aid in developing personalized cancer treatment strategies and that the relevant PRGs could serve as potential LUAD therapeutic targets.

## 2. Materials and Methods

### 2.1. LUAD Sequencing Data and Peroxisome-Related Gene Collection

TCGA database (https://cancergenome.nih.gov/) provides genomic sequence, mutation, and clinical data of more than 30 different types of cancer. For this study, genomic sequence, mutation, and clinical data of 535 LUAD tissues were obtained from it. In addition, the sequencing data of 59 normal tissues were downloaded for differential expression analysis. Of the 535 LUAD patients, 49 patients, which lacked follow-up time or were followed up for less than 1 month, were removed.

GEO database (https://www.ncbi.nlm.nih.gov/geo/) also provides sequence and clinical data of various diseases. GSE3141, GSE31210, and GSE72094, which were consisted of LUAD sequence and clinical data, were downloaded as validation data sets. GSE3141 included 111 LUAD cases. GSE31210 included 174 LUAD cases. GSE72094 included 442 LUAD cases.

The peroxisomeDB database (http://www.peroxisomedb.org) integrates genomic information about peroxisome proteomes in humans and other organisms [17]. Total 73 peroxisome-related genes (PRGs) were obtained from it (Table S1).

### 2.2. Differential Expression Analysis of Peroxisome-Related Genes

The differentially expressed PRGs were screened according to FDR < 0.05 using “limma” package, and the result was demonstrated with boxplot using “ggpubr” package. Functional enrichment analyses of differentially expressed PRGs, including KEGG and GO analysis, were conducted using org “org.Hs.eg.db,” “enrichplot,” and “clusterprofiler” packages. The interaction between protein-protein was analyzed in STRING website (http://string.embl.de/).

### 2.3. Construction of a Prognostic Signature with LASSO Logistic Regression

Based on differentially expressed PRGs, prognosis-related PRGs were selected with the univariate Cox regression analysis using “survival” package (*p* value < 0.05). Based on prognosis-related PRGs, LASSO logistic regression was utilized to construct the prognostic signature using “glmnet” R package. The penalty parameter (*λ*) of LASSO logistic regression was estimated. The formula was displayed: Risk score = sum (Gene_i_′s coefficient∗Gene_i_′s expression). According to the median of risk score, the risk score of patients was classified into binary variables, including high risk and low risk. Survival analysis was performed between the two groups to assess whether there were differences in survival. ROC, PCA, and t-SNE analyses were utilized to evaluate the effectiveness of the signature. The Cox regression analysis was conducted to see if the signature was a significant independent predictor.

### 2.4. Validation of the Signature with GEO Data Sets

To observe whether the performance of the signature was robust, three GEO data sets, including GSE3141, GSE31210, and GSE72094, were obtained as validation cohorts.

### 2.5. Analysis of Immune Microenvironment

To find out the difference of immune microenvironment between two risk groups, infiltration of immune cells and expressions of immune checkpoints were analyzed. Using the “estimate” package, the ESTIMATE method was used to estimate the stromal and immune cell proportions in each of the LUAD samples. ssGSEA was applied to quantify the enrichment degrees of sixteen immune cells in TCGA data set and GSE72094 using “GSEABase” and “GSVA” packages. The expression of 47 immune checkpoints was analyzed between the two risk groups, and the differentially expressed checkpoints were exhibited.

TIDE (tumor immune dysfunction and exclusion) is a computational method that simulates tumor immune evasion. A high TIDE score indicates the greater tendency of tumor immune escape and the worse effect of immune checkpoint blockade treatment (ICB) [18]. TIDE score of patients in TCGA data set was downloaded from TIDE website (http://tide.dfci.harvard.edu/).

### 2.6. Relationship between Risk Score and Ferroptosis

It has been reported that peroxisome induced ferroptosis by synthesizing polyunsaturated lipids [19]. To observe whether there were differences in the activity of ferroptosis between the two risk groups, we obtained four representative ferroptosis-related genes from the literature, including ACSL4, TFRC, PTGS2, and CHAC1 [20]. Ferroptosis in different risk groups was observed by comparing the expression of these four genes in different risk groups.

### 2.7. Relationship between Risk Score and Cancer Stem Cell Infiltration

Cancer stem cells may be the source of tumor cells and could cause drug resistance and lead to distant metastasis [21]. The cancer stem cell index, representing the infiltration degrees of cancer stem cells, was calculated at the DNA and RNA levels, respectively, to examine the differences in stem cell infiltration between risk groups.

### 2.8. Relationship between Risk Score and Tumor Mutation Burden

Tumor mutation burden (TMB) has been proved to be a useful biomarker for the selection of ICB in some cancer types, including lung cancer [22]. We compared TMB of two risk groups by calculating the TMB of each sample in the TCGA data set. The information about signature genes mutation was obtained from cBioPortal database (http://www.cbioportal.org/).

### 2.9. Gene Set Enrichment Analysis

To find out different biological processes and possible pathways between two risk groups, GSEA was conducted, including GO and KEGG assays.

### 2.10. Screening of Targeted Drugs

CellMiner integrates a variety of drugs approved by FDA and the expression of genes, providing drug sensitivity information file for free. Thus, we downloaded gene expression and drug files from it to explore the relationship between signature genes and drugs. Based on the *p* value, the results of the first 20 analyses were visualized.

### 2.11. Immunohistochemistry Assay

The Human Protein Atlas (HPA) database provides immunohistochemical verification of genes in various normal tissues and tumors. We obtained immunohistochemical verification of all signature genes in normal and LUAD tissues from it.

### 2.12. Statistical Methods for Processing Data

All statistics were conducted using R software (version 4.1.2). All results were considered statistically significant with *p* < 0.05.

## 3. Results

### 3.1. Differential Expression Analysis of PRGs

**Figure 1:**
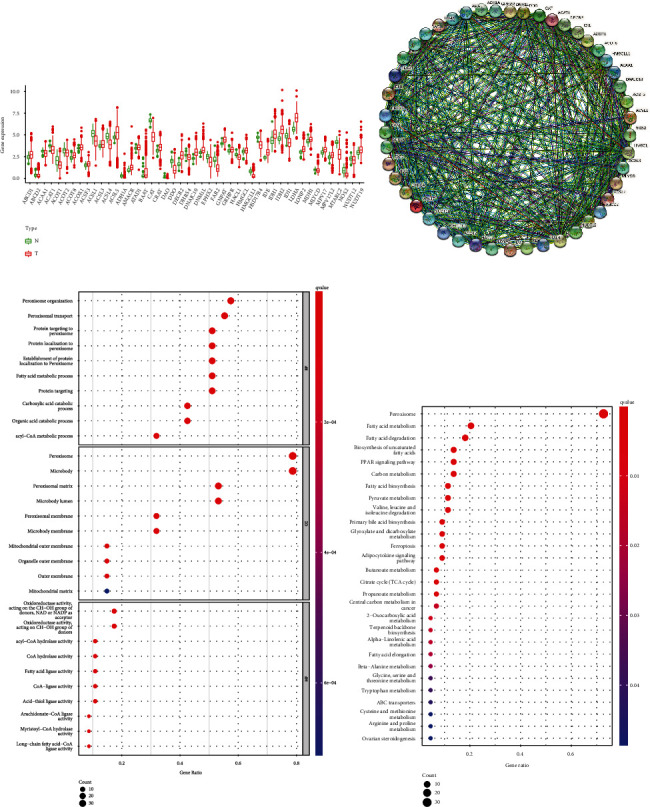
Differentially expressed PRGs and their functional enrichment analysis. (a) Expression of 47 PRGs. (b) Interaction of differentially expressed PRGs' protein. (c) GO analysis of differentially expressed PRGs. (d) KEGG analysis of differentially expressed PRGs. N: normal; T: tumor.

There were 47 differentially expressed PRGs, including 25 increased and 22 decreased PRGs (Figure 1(a)). The proteins expressed by these 47 PRGs were interrelated (Figure 1(b)). The results of GO showed that these differentially expressed PRGs were largely related to the biological processes related to peroxisome and lipid metabolism, such as peroxisome organization, peroxisomal matrix, and fatty acid ligase activity (Figure 1(c)). KEGG showed that these genes were involved in a variety of pathways, in addition to fatty metabolism-related pathways, but also PPAR signaling pathway, glyoxylate and dicarboxylate metabolism, ferroptosis, and so on (Figure 1(d)). These results indicated that these differentially expressed genes were indeed peroxisome-related genes and played some role in a variety of pathways. It was worth further studying their role in LUAD.

### 3.2. Construction of a Prognostic PRG Signature

**Figure 2:**
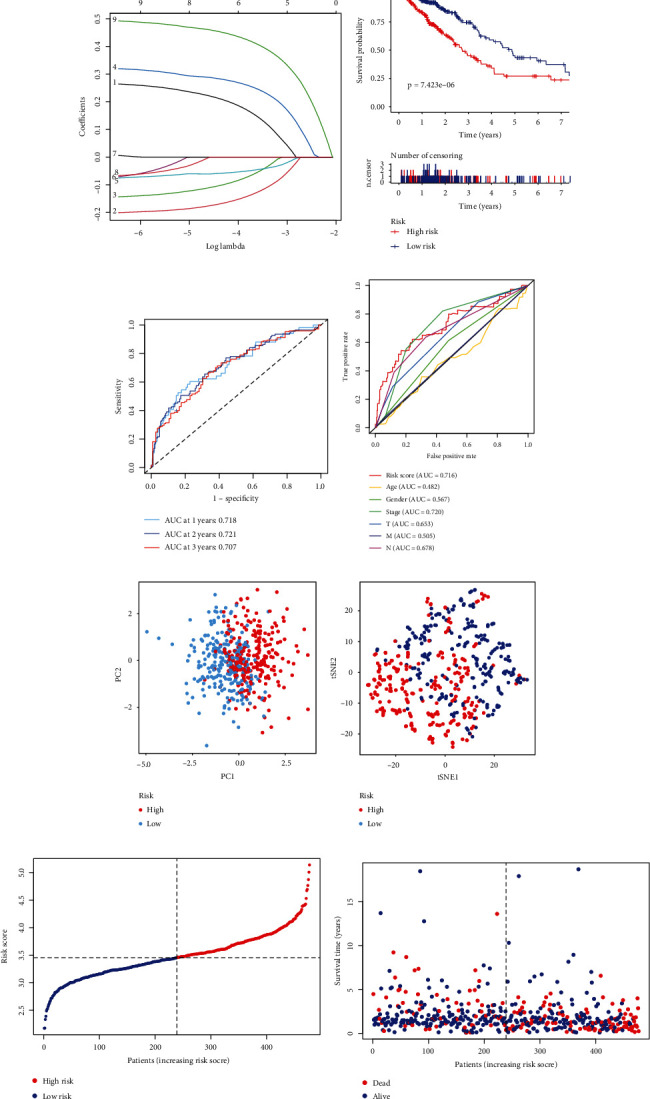
Construction of a prognostic PRG signature. (a) Forest plot showing nine prognosis-related PRGs. (b) Selection of penalty parameter in LASSO logistic regression. (c) Coefficient of the signature genes when the penalty parameter was minimum. (d) Survival analysis of patients in TCGA data set. (e) ROC curve of TCGA data set. (f) ROC curve of risk score and clinical features. (g) PCA of TCGA data set. (h) t-SNE of TCGA data set. (i) Scatter plots showing the distribution of risk scores. (j) Scatter plots the relationship between risk score and survival time. (k) Forest plot of the univariate Cox analysis. (l) Forest plot of the multivariate Cox analysis.

To screen the prognostic genes in LUAD, we performed the univariate Cox regression analysis. The results confirmed that total nine PRGs were associated with clinical outcomes of LUAD (Figure 2(a)). Six PRGs were screened to construct the signature when *λ* was at minimum (Figures 2(b) and 2(c)). The formula was as follows: Risk score = [(0.180594464∗ABCD1) + (−0.14906947∗ACAT1) + (−0.080152934∗ACSL1) + (0.26612956∗ACSL3) + (−0.052574859∗CAT) + (0.431596295∗LDHA)]. The patients with high risk score showed a shorter overall survival than those with low risk score (Figure 2(d)). The AUCs at 1, 2, and 3 years were 0.718, 0.721, and 0.707, respectively (Figure 2(e)). Compared with AUCs of multiple clinical features, the AUC of risk score had obvious advantages (Figure 2(f)). The PCA and t-SEN analyses showed that the signature could well distinguish low-risk patients from high-risk patients (Figures 2(g) and 2(h)). The relationship between risk score and survival time and the distribution of risk score were visualized as scatter plots (Figures 2(i) and 2(j)). The univariate and multivariate Cox analyses both showed that the *p* value of risk score was less than 0.05, indicating that risk score was an independent indicator for patients with LUAD (Figures 2(k) and 2(l)). Our findings suggested that this new model may be used to predict the clinical outcome of LUAD patients.

### 3.3. Validation of the Signature with External Data Sets

**Figure 3:**
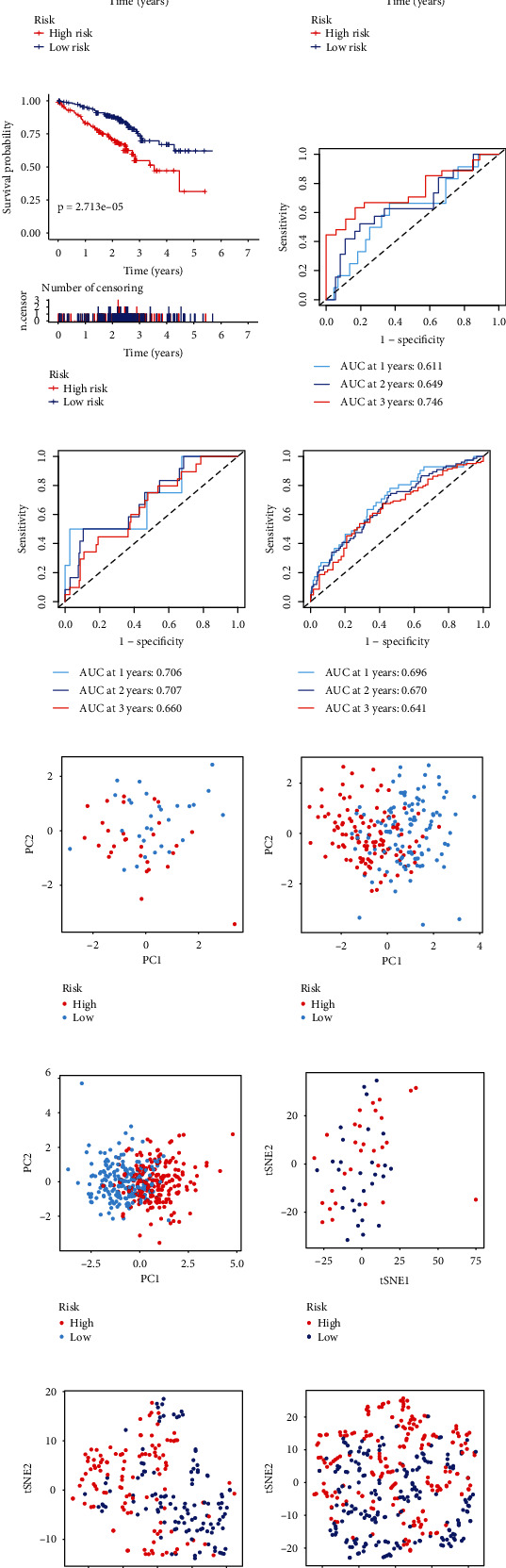
Validation of the signature using GSE3141, GSE31210, and GSE72094. (a–c) Survival analysis of GEO data sets. (d–f) ROC curve of GEO data sets. (g–i) PCA of GEO data sets. (j–l) t-SNE of GEO data sets. (a, d, g, and j) GSE3141. (b, e, h, and k) GSE31210. (c, f, i, and l) GSE72094.

The risk score of all patients in validation data sets was calculated. The survival analysis results of all validation data sets showed that the survival probability of high-risk group was significantly lower than the low-risk group, consisting with the results of TCGA data set (Figures 3(a)–3(c)). The AUCs of GSE3141 at 1, 2, and 3 years were 0.611, 0.649, and 0.746, respectively (Figure 3(d)). The AUCs of GSE31210 at 1, 2, and 3 years were 0.706, 0.707, and 0.660, respectively (Figure 3(e)). The AUCs of GSE72094 at 1, 2, and 3 years were 0.696, 0.670, and 0.641, respectively (Figure 3(f)). PCA and tSNE analyses also demonstrated that the signature can distinguish between low-risk and high-risk patients (Figures 3(g)–3(l)). The relationship between risk score and survival time and the distribution of risk score were also visualized (Figure S1A–F).

### 3.4. Analysis of Immune Microenvironment

**Figure 4:**
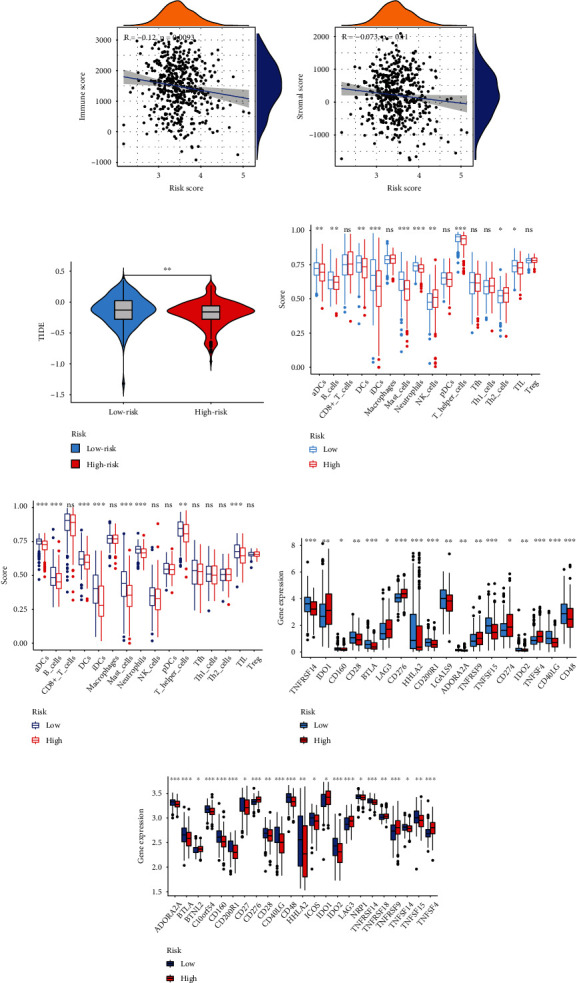
Analysis of immunity. (a) The relationships between infiltration of immune cells and risk score. (b) The relationship between infiltration of stromal cells and risk score. (c) TIDE scores of two groups. (d) Immune cells in TCGA data set. (e) Immune cells in GSE72094. (f) Differentially expressed immune checkpoints in TCGA data set. (g) Differentially expressed immune checkpoints in GSE72094.

It showed that the infiltration of immune cells was negatively associated with the risk score, while there was no correlation between the infiltration of stromal cell and risk score (Figures 4(a) and 4(b)). The TIDE score of low-risk group was significantly higher than high-risk group, suggesting that the high-risk group had a better response to ICB treatment (Figure 4(c)). Furthermore, we analyzed which cells were associated with the risk score. The ssGSEA results of TCGA data set and GSE72094 showed that the infiltration of DCs, B cells, mast cells, neutrophils, T helper cells, and TIL cells were higher in low-risk than high-risk group (Figures 4(d) and 4(e)). There were 18 immune checkpoints in TCGA data set, and 23 immune checkpoints in GSE72094 exhibited a dysregulated level between two risk groups (Figures 4(f) and 4(g)). And there were 16 common differentially expressed immune checkpoints in these two data sets, such as CD276, BTLA, and CD28.

### 3.5. Relationship between Risk Score and Ferroptosis

**Figure 5:**
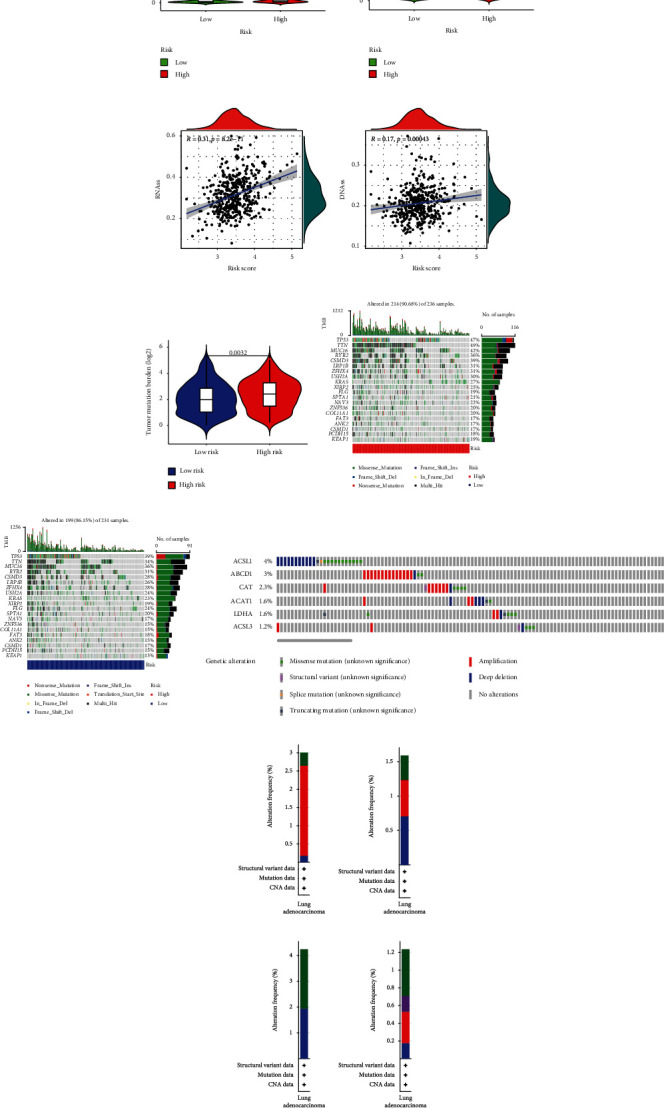
Analyses of ferroptosis, cancer stem cell infiltration, and TMB in two risk groups. (k–p) Mutation type and mutation rate of signature genes. (a) Expression of ACSL4 in two groups. (b) Expression of CHAC1 in two groups. (C) Expression of PTGS2 in two groups. (d) Expression of TFRC in two groups. (e) Measuring of cancer stem cell infiltration at RNA level. (f) Measuring of cancer stem cell infiltration at DNA level. (g) TMB of two groups. (h) Gene mutation in high-risk group. (i) Gene mutation in low-risk group. (j) Summary of mutations of six signature genes. (k) ABCD1. (l) ACAT1. (m) ACSL1. (n) ACSL3. (o) CAT. (p) LDHA.

The expression of ACSL4, TFRC, PTGS2, and CHAC1 was higher in high-risk group than in the low-risk group (Figures 5(a)–5(d)), indicating that ferroptosis in high-risk group is more active.

### 3.6. Relationship between Risk Score and Cancer Stem Cell Infiltration

The *R* value was 0.31 (*p* = 8.2e − 11) in the RNA score, and 0.17 (*p* = 0.00043) in DNA score, indicating that risk score was positively associated with the degree of tumor stem cell infiltration (Figures 5(e) and 5(f)).

### 3.7. Relationship between Risk Score and TMB

The TMB of high-risk group was higher than low-risk group (Figure 5(g)). The gene mutation rate was 90.68% in the high-risk group and 86.15% in the low-risk group (Figures 5(h) and 5(i)). The gene with the highest mutation rate in the high-risk group was TNN. Among six signature genes, the mutation rates of ACSL1 were the highest, and ACSL1 was the lowest (Figure 5(j)). The common mutation type in six genes was deep deletion (Figures 5(k)–5(p)).

### 3.8. Gene Set Enrichment Analysis

**Figure 6:**
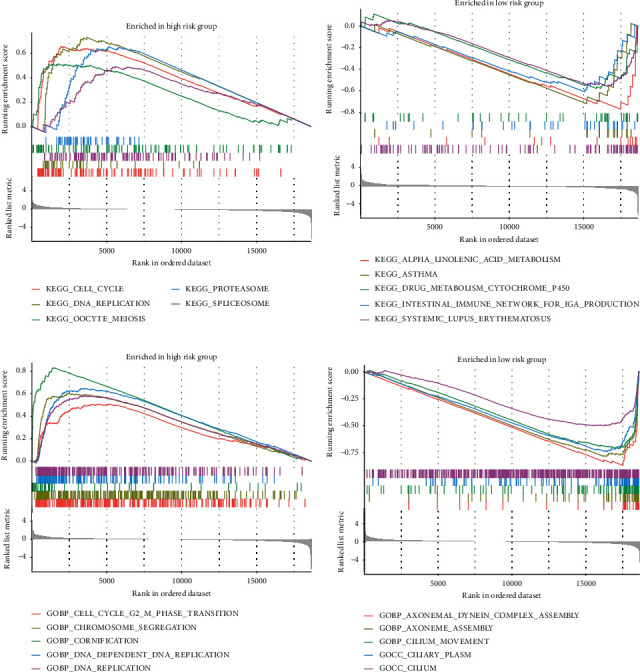
The result of GSVA. (a and b) The result of KEGG analysis. (c and d) The result of GO analysis. (a and c) High-risk group. (b and d) Low-risk group.

KEGG showed that pathways of high-risk group enriched in proteasome, cell cycle, DNA replication, and so on and pathways of low-risk group enriched in *α*-linolenic acid metabolism, asthma, drug metabolism cytochrome P450, and so on (Figures 6(a) and 6(b)). GO showed that biological processes of high-risk group were chromosome segregation, cell cycle G2-M phase transition, DNA replication, and so on, and low-risk group were axoneme assembly, cilium movement, ciliary plasm, and so on (Figures 6(c) and 6(d)). More results of KEGG and GO are listed in Tables S2 and S3.

### 3.9. Screening of Targeted Drugs

**Figure 7:**
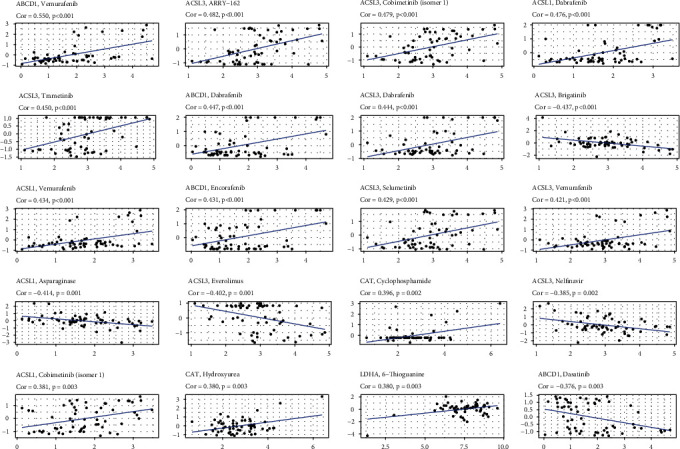
The relationship between expression of signature genes and drugs.

If the correlation coefficient was greater than 0, the gene was sensitive to drugs, and if the correlation coefficient was less than 0, the gene was resistant to drugs. It showed that ABCD1, ACSL1, ACSL3, and LDHA were sensitive to dabrafenib (Figure 7). ACAT1 was sensitive to parthenolide and CAT was sensitive to crizotinib (Table S4).

### 3.10. Validation of Signature Genes with Immunohistochemistry

**Figure 8:**
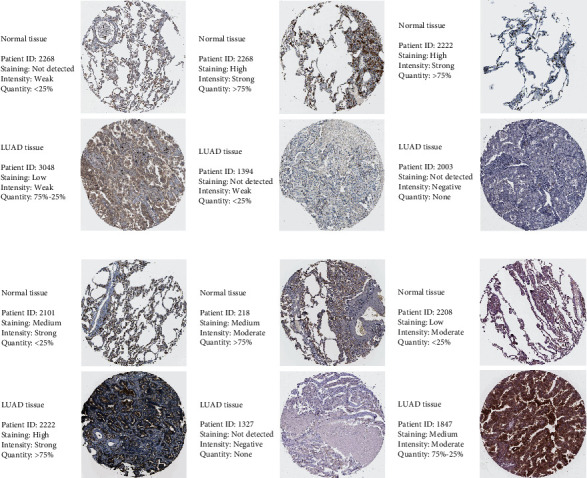
Validation of signature genes with immunohistochemistry. (a) ABCD1. (b) ACAT1. (c) ACSL1. (d) ACSL3. (e) CAT. (f) LDHA.

The result of immunohistochemistry showed that the expression of ABCD1, ACSL3, and LDHA was higher in LUAD tissue than normal tissue and the expression of ACAT1, ACSL1, and CAT was lower in LUAD tissues, consistent with the results of differential expression analysis (Figures 8(a)–8(f)).

## 4. Discussion

LUAD is a public health concern for its high morbidity and mortality [23]. There are numerous difficulties in designing personalized treatment plans for LUAD because of its high tumor heterogeneity and complex tumorigenic mechanism [24, 25]. Therefore, robust prognostic signature is necessary. In recent years, novel prognostic biomarkers based on PRGs have been developed in hepatocellular carcinoma. Wu et al. developed a prognostic model of 9 PRGs. They also confirmed that overall survival was much greater in the low-risk group compared to the high-risk group [26]. However, in other types of tumors, the diagnostic and prognostic models based on PRGs have not been reported.

In this study, we analyzed TCGA data sets and identified 47 differentially expressed PRGs, including 25 increased and 22 decreased PRGs. KEGG showed that these genes were involved in a variety of pathways, in addition to fatty metabolism-related pathways, but also PPAR signaling pathway, glyoxylate and dicarboxylate metabolism, ferroptosis, and so on. These results indicated that these differentially expressed genes were indeed peroxisome-related genes and played some role in a variety of pathways. After that, a prognostic risk model based on the univariate Cox and LASSO Cox regression was developed, which included nine genes. Finally, six PRGs (ABCD1, ACAT1, ACSL1, ACSL3, CAT, and LDHA) were screened to construct the prognostic signature. Survival assays revealed that the patients with high risk score showed a shorter overall survival than those with low risk score. The multivariate Cox analyses further confirmed that risk score was an independent predictor of LUAD, highlighting the potential of the new model used as a novel prognostic biomarkers for LUAD patients. Moreover, the risk model was effective and can be applied to predict clinical outcome of LUAD patients via external validations, including GSE3141, GSE31210, and GSE72094 data sets.

There is clear doubt that the immune system has a role in promoting or suppressing cancer, and one of the most effective new anticancer medications is the immunocheckpoint therapy [27, 28]. Our findings showed that the immunological status of low- and high-risk LUAD patients differed significantly, including DCs, B cells, mast cells, neutrophils, T helper cells, and TIL cells. There were 18 immune checkpoints in TCGA data set, and 23 immune checkpoints in GSE72094 were differentially expressed between two risk groups. And there were 16 common differentially expressed immune checkpoints in these two data sets, such as CD276, BTLA, and CD28. Experiments in vivo and vitro are gradually uncovering the cryptic and complex relationships between immunity and ferroptosis. There was an abundance of lipids and polyunsaturated fats in DCs in tumor-bearing animals, which reduced their ability to deliver antigen and trigger CD8+ T cell responses. DCs and CD8+ T lymphocytes may be involved in ferroptosis via modulating lipid and PUFA levels, according to previous studies. In addition, experimental evidence shows that T cell lipoperoxidation can impair immunity to infection by inducing ferroptosis in the cells. Consistent with the results of previous studies, CD8+ T lymphocytes have been shown to induce ferroptosis-specific lipid peroxidation and ferroptosis, therefore enhancing immunotherapy's efficacy in preclinical models. The PRG signature has emerged as an important prognostic marker and therapy option for LUAD patients, as evidenced by the correlation between risk scores and immunity.

A subset of tumor cells known as cancer stem cells (CSCs) has been identified as having the ability to control self-renewal and differentiation, making it difficult to eradicate the tumor [29, 30]. Breast cancer, glioma, and LUAD are among the many solid tumors where CSCs have been found [31–33]. In addition, we checked whether there was a link between the expression of PRG and RNAss in LUAD. Intriguingly, the outcomes were shown to be negatively related to the expression of stem cells in distinct malignancies. As a result, it was clear that the model we developed was capable of identifying CSC scores and that focusing on those genes might alter CSCs in order to prevent LUAD.

Drug resistance in LUAD treatment is on the rise, according to mounting evidence, and this is resulting in less-than-optimal therapeutic outcomes [34, 35]. The increased expression of genes associated with multidrug resistance and the decrease in drug sensitivity have piqued interest. As a result, the LUAD chemotherapy prognostic model was given additional attention. Consistently, the findings exhibited that ABCD1, ACSL1, ACSL3, and LDHA were sensitive to dabrafenib. ACAT1 was sensitive to parthenolide and CAT was sensitive to crizotinib. Our findings suggested that chemotherapy for LUAD patients with lower expression of prognostic genes was beneficial.

However, our study has several limitations. First, using a single characteristic (peroxisome-related genes) to establish the predictive model was an intrinsic weakness. In fact, LUAD progress and development were influenced by a wide range of factors. Second, retrospective data sets from a public database were used to both create and validate the current model. In order to confirm its clinical value, additional data from prospective studies were still required. Third, these prognostic PRGs in LUAD necessitated more investigation to better understand their functions and processes.

## 5. Conclusion

A stable prognostic signature was established based on peroxisome-related genes to help the classification of LUAD patients. In addition, we conducted a complete review of immunology, TMB, and tumor stem cell invasion to find new therapy options. A wide range of medications targeting various genes and patient populations were examined. Novel prognostic biomarkers and therapeutic targets can be derived from the genes found in the prognostic signature.

## Data Availability

The data sets used and/or analyzed during the current study are available from the corresponding author on reasonable request.

## Supplementary Materials

Supplementary 1 Figure S1: the distribution of risk scores in GEO data sets and the relationship between risk score and survival time. (A, C, and E) The distribution of risk scores. (B, D, and F) The relationship between risk score and survival time. (A and B) GSE3141. (C and D) GSE31210. (E and F) GSE72094.

Supplementary 2 Table S1: total 73 peroxisome-related genes.

Supplementary 3 Table S2: the result of GSVA-KEGG.

Supplementary 4 Table S3: the result of GSVA-GO.

Supplementary 5 Table S4: drugs targeting signature genes.
