# Supplementary figures and images for: A New Prognostic Indicator of Immune Microenvironment and Therapeutic Response in Lung Adenocarcinoma Based on Peroxisome-Related Genes
Source: J Immunol Res. 2022 Jul 26;2022:6084589. doi: 10.1155/2022/6084589 (PMC9346542; doi:10.1155/2022/6084589)

A

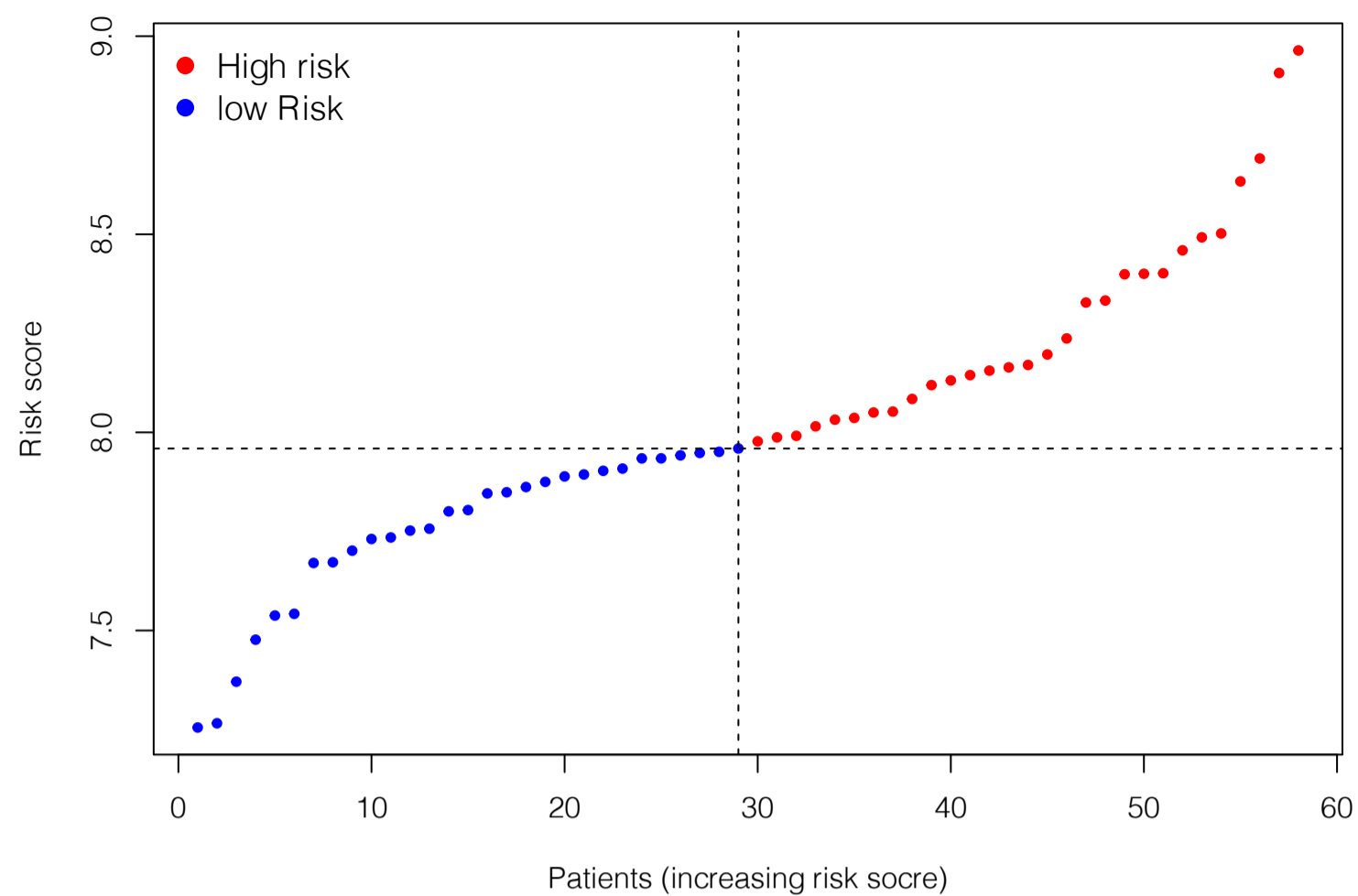

B

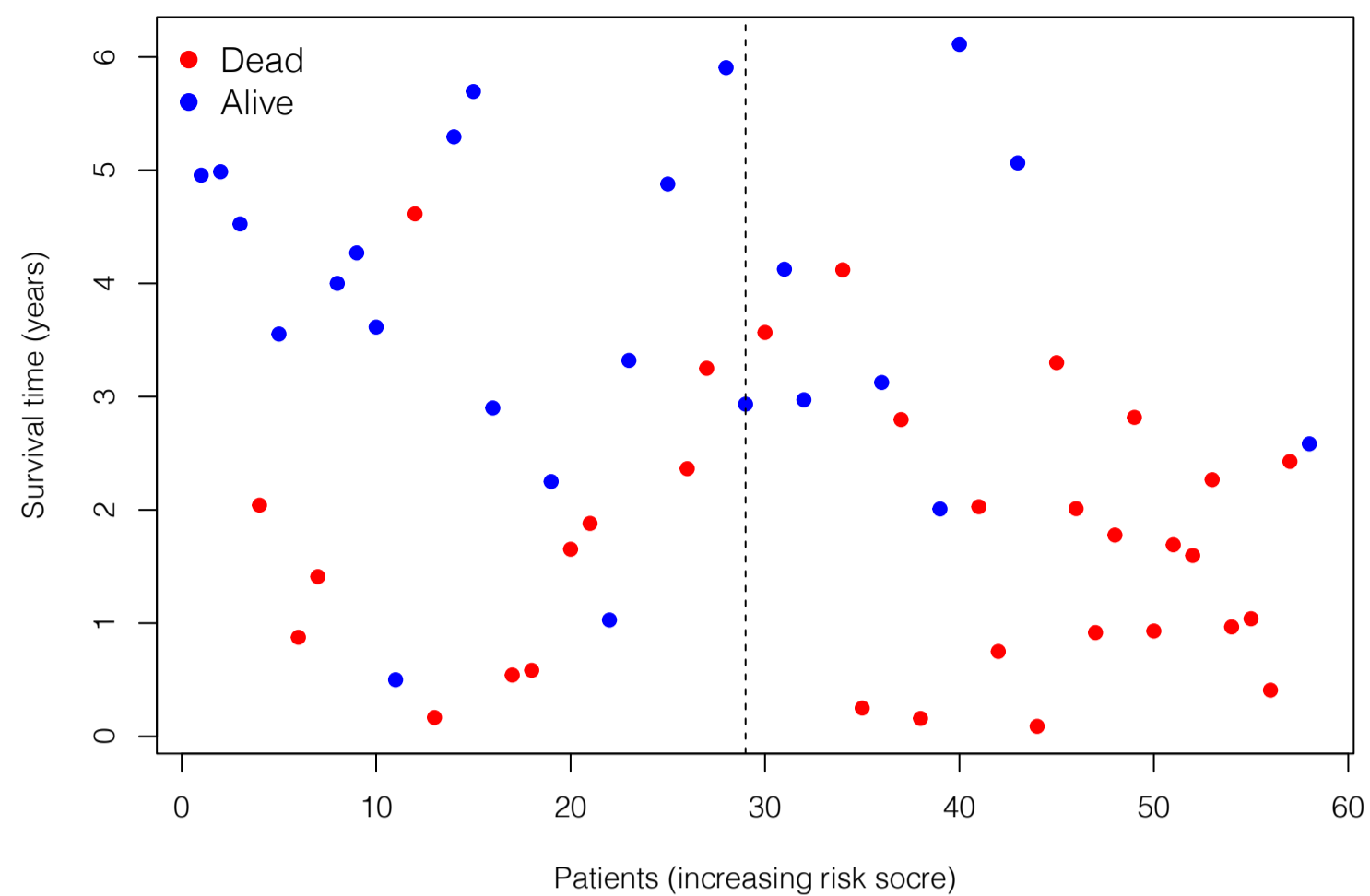

C

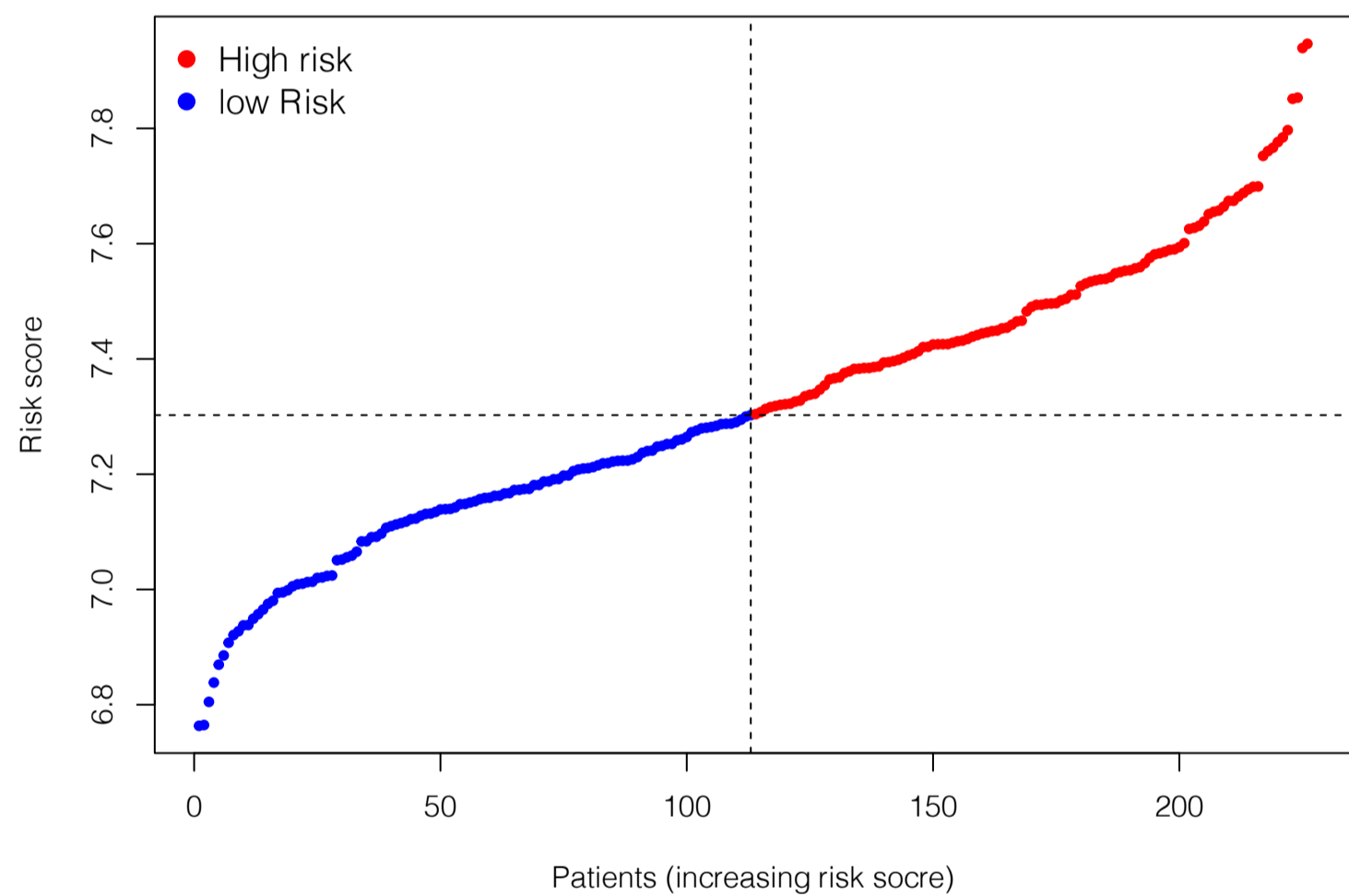

D

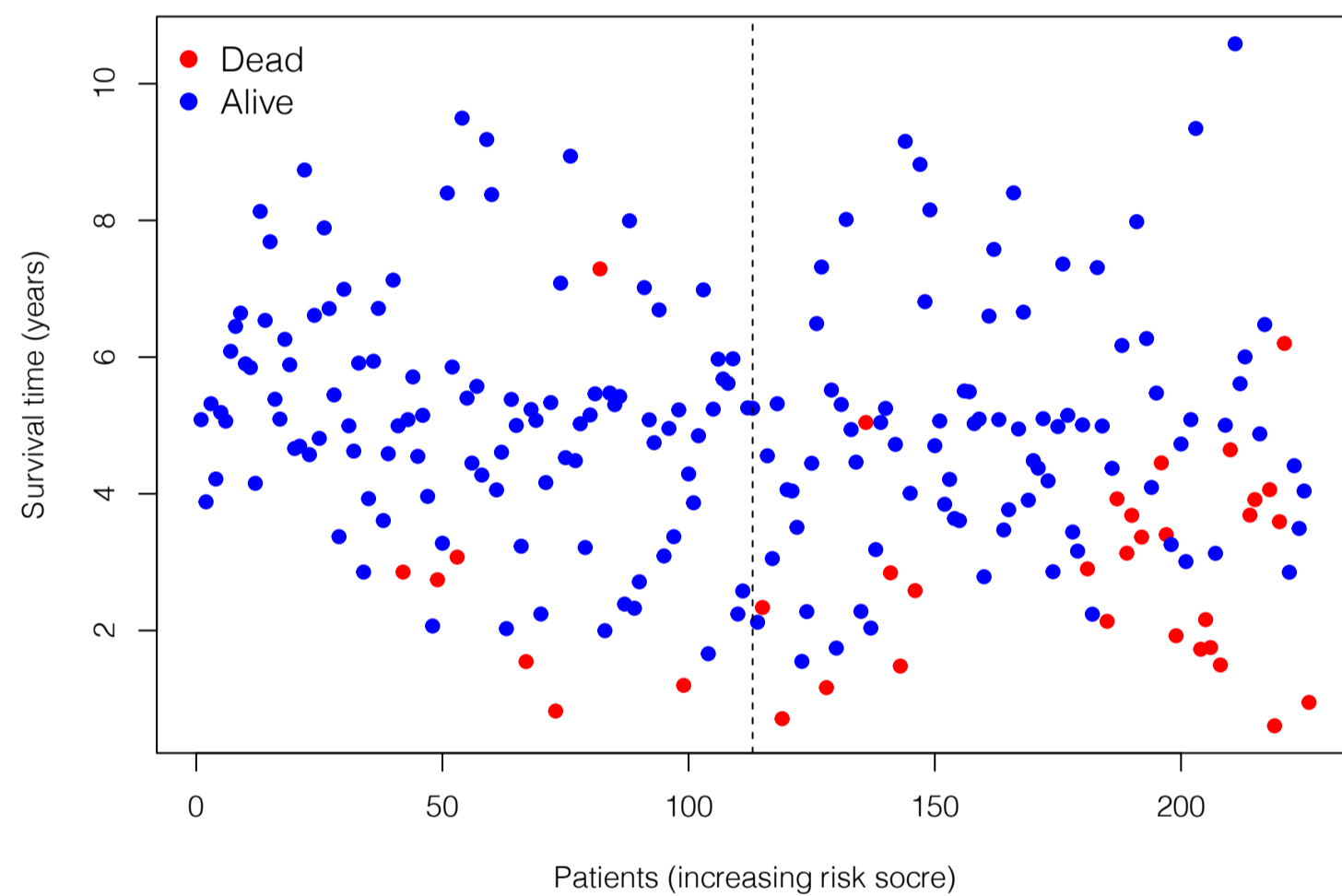

E

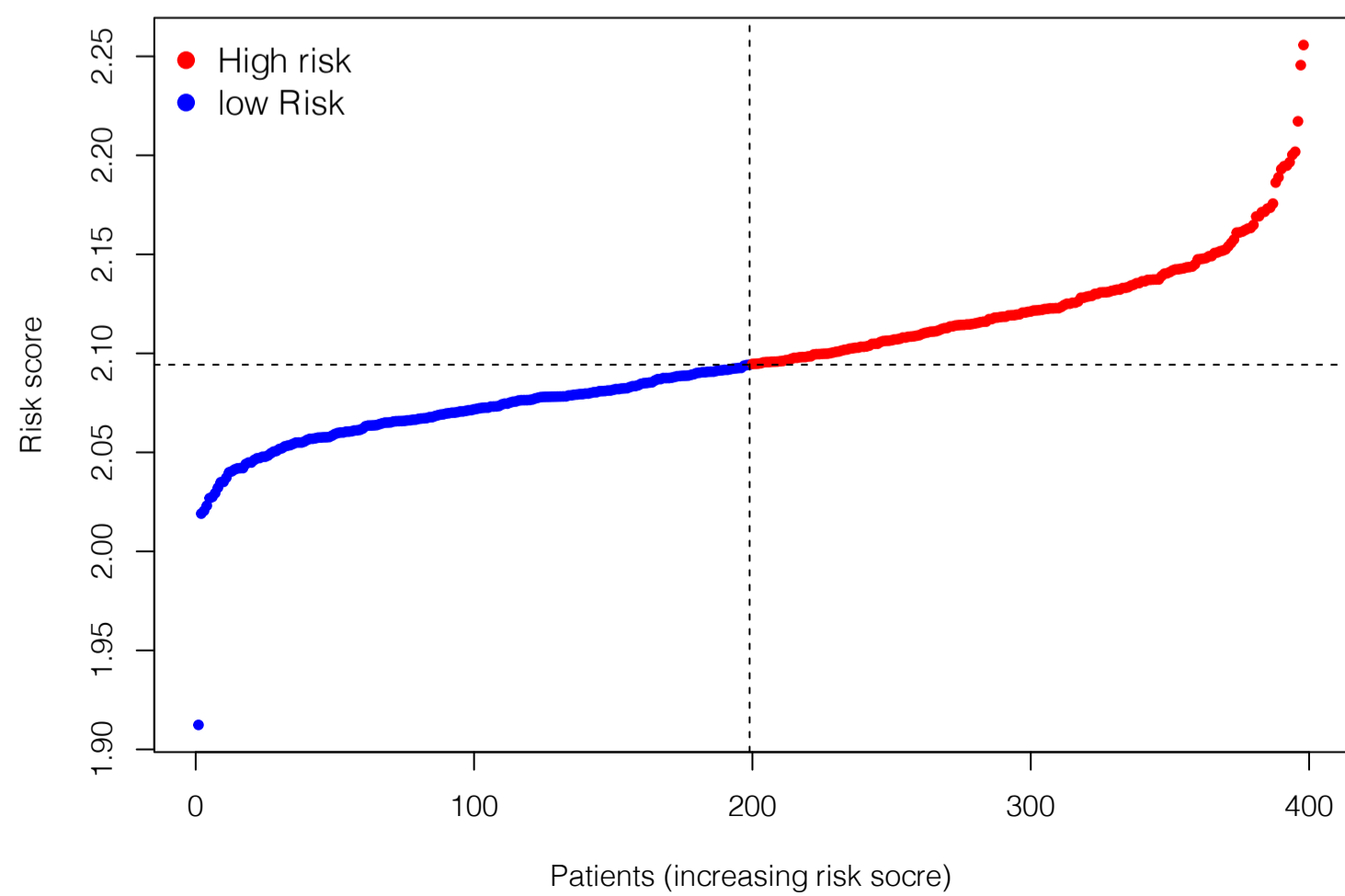

F

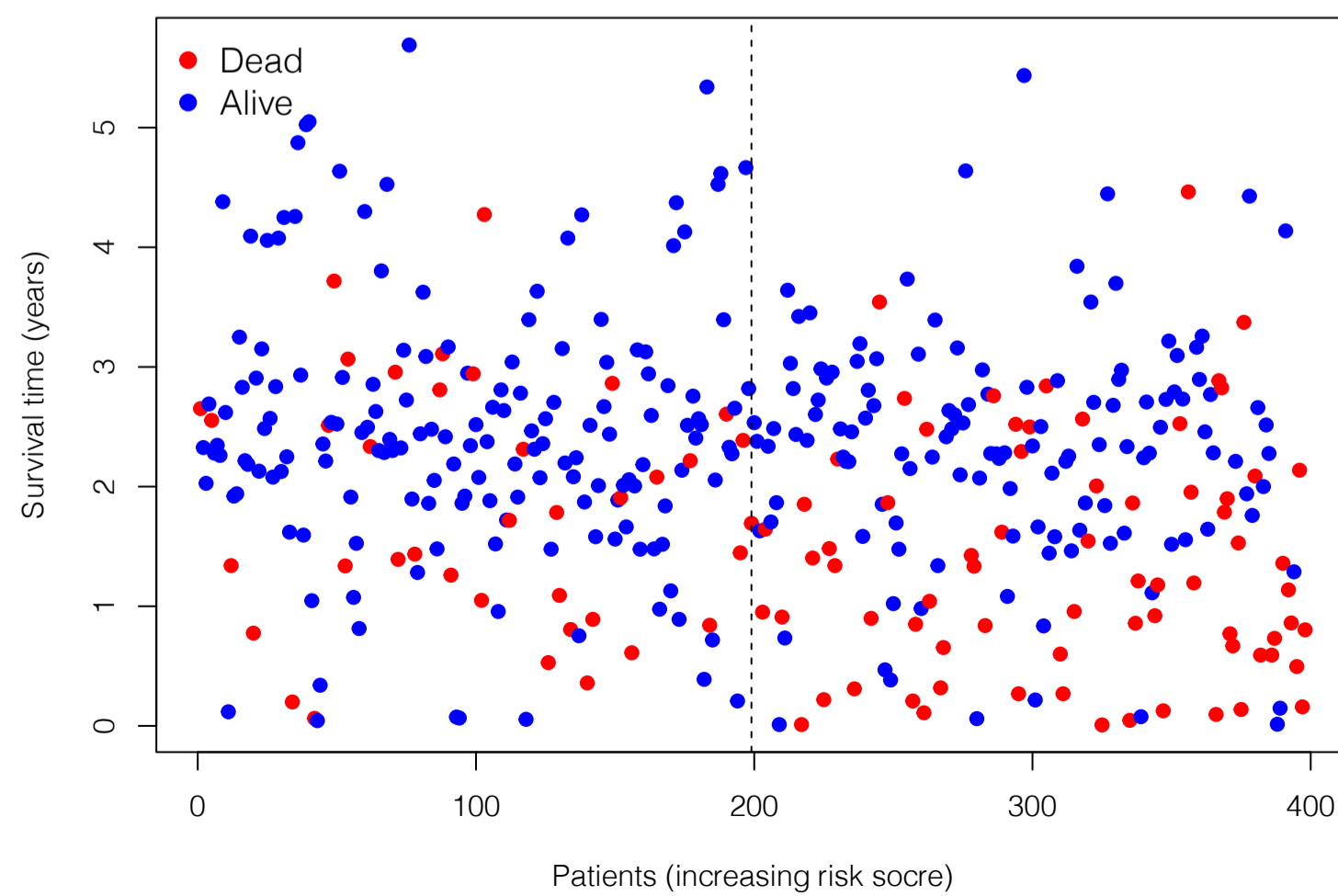

Supplement: Supplementary 1 — Figure S1: the distribution of risk scores in GEO data sets and the relationship between risk score and survival time. (A, C, and E) The distribution of risk scores. (B, D, and F) The relationship between risk score and survival time. (A and B) GSE3141. (C and D) GSE31210. (E and F) GSE72094. [file 6084589.f1.pdf]
