# Supplementary material for: A New Prognostic Indicator of Immune Microenvironment and Therapeutic Response in Lung Adenocarcinoma Based on Peroxisome-Related Genes
Source: J Immunol Res. 2022 Jul 26;2022:6084589. doi: 10.1155/2022/6084589 (PMC9346542; doi:10.1155/2022/6084589)
Supplement: Supplementary 2 — Table S1: total 73 peroxisome-related genes. [file 6084589.f2.docx]

Table S1 Total 73 Peroxisome-Related Genes

| Genes Symbol | Full Name | Also Known as |
| --- | --- | --- |
| ABCD1 | ATP binding cassette subfamily D member 1 | ALD/ AMN/ ALDP/ ABC42 |
| ABCD2 | ATP binding cassette subfamily D member 2 | ABC39/ ALDL1/ ALDR/ ALDRP/ hALDR |
| ABCD3 | ATP binding cassette subfamily D member 3 | ABC43/ CBAS5/ PMP70/ PXMP1/ ZWS2 |
| ABCD4 | ATP binding cassette subfamily D member 4 | ABC41/ EST352188/ MAHCJ/ P70R/ P79R/ PMP69/ PXMP1L |
| ACAA1 | acetyl-CoA acyltransferase 1 | ACAA/ Lnc-Myd88/ PTHIO/ THIO |
| ACAD11 | acyl-CoA dehydrogenase family member 11 | ACAD-11 |
| ACAT1 | acetyl-CoA acetyltransferase 1 | ACAT/ MAT/ T2/ THIL |
| ACBD5 | acyl-CoA binding domain containing 5 | RDLKD |
| ACOT1 | acyl-CoA thioesterase 1 | ACH2/ CTE-1/ LACH2 |
| ACOT2 | acyl-CoA thioesterase 2 | CTE-IA/ CTE1A/ MTE1/ PTE2/ PTE2A/ ZAP128 |
| ACOT4 | acyl-CoA thioesterase 4 | PTE-Ib/ PTE1B/ PTE2B |
| ACOT8 | acyl-CoA thioesterase 8 | HNAACTE/ NAP1/ PTE-1/ PTE-2/ PTE1/ PTE2/ hACTE-III/ hTE |
| ACOX1 | acyl-CoA oxidase 1 | ACOX/ MITCH/ PALMCOX/ SCOX |
| ACOX2 | acyl-CoA oxidase 2 | BCOX/ BRCACOX/ BRCOX/ CBAS6/ THCCox |
| ACOX3 | acyl-CoA oxidase 3, pristanoyl | / |
| ACSF3 | acyl-CoA synthetase family member 3 | / |
| ACSL1 | acyl-CoA synthetase long chain family member 1 | ACS1/ FACL1/ FACL2/ LACS/ LACS1/ LACS2 |
| ACSL3 | acyl-CoA synthetase long chain family member 3 | ACS3/ FACL3/ LACS 3/ LACS3/ PRO2194 |
| ACSL4 | acyl-CoA synthetase long chain family member 4 | ACS4/ FACL4/ LACS4/ MRX63/ MRX68/ XLID63 |
| ACSL5 | acyl-CoA synthetase long chain family member 5 | ACS2/ ACS5/ FACL5 |
| ACSL6 | acyl-CoA synthetase long chain family member 6 | ACS2/ FACL6/ LACS6/ LACS2/ LACS5 |
| ADH1A | alcohol dehydrogenase 1A (class I), alpha polypeptide | ADH1 |
| AGPS | alkylglycerone phosphate synthase | ADAP-S/ ADAS/ ADHAPS/ ADPS/ ALDHPSY/ RCDP3 |
| AGXT | alanine--glyoxylate and serine--pyruvate aminotransferase | AGT/ AGT1/ AGXT1/ PH1/ SPAT/ SPT/ Ser-PyrAT/ TLH6 |
| ALDH3A2 | aldehyde dehydrogenase 3 family member A2 | ALDH10/ FALDH/ SLS |
| AMACR | alpha-methylacyl-CoA racemase | AMACRD/ CBAS4/ P504S/ RACE/ RM |
| ATAD1 | ATPase family AAA domain containing 1 | AFDC1/ FNP001/ HKPX4/ Msp1/ THORASE/ hATAD1 |
| BAAT | bile acid-CoA: amino acid N-acyltransferase | BACAT/ BACD1/ BAT/ HCHO |
| CAT | catalase | / |
| CRAT | carnitine O-acetyltransferase | CAT/ CAT1/ NBIA8 |
| CROT | carnitine O-octanoyltransferase | COT |
| DAO | D-amino acid oxidase | DAAO/ DAMOX/ OXDA |
| DDO | D-aspartate oxidase | DASOX/ DDO-1/ DDO-2 |
| DECR2 | 2,4-dienoyl-CoA reductase 2 | PDCR/ SDR17C1 |
| DHRS4 | dehydrogenase/reductase 4 | CR/ NRDR/ PHCR/ PSCD/ SCAD-SRL/ SDR-SRL/ SDR25C1/ SDR25C2 |
| DHRS4L1 | dehydrogenase/reductase 4 like 1 | SDR25C4 |
| DNAJC10 | DnaJ heat shock protein family (Hsp40) member C10 | ERdj5/ JPDI/ MTHr/ PDIA19 |
| DNM1L | dynamin 1 like | DLP1/ DRP1/ DVLP/ DYMPLE/ EMPF/ EMPF1/ HDYNIV/ OPA5 |
| ECH1 | enoyl-CoA hydratase 1 | HPXEL |
| ECI2 | enoyl-CoA delta isomerase 2 | ACBD2/ DRS-1/ DRS1/ HCA88/ PECI/ dJ1013A10.3 |
| EHHADH | enoyl-CoA hydratase and 3-hydroxyacyl CoA dehydrogenase | ECHD/ FRTS3/ L-PBE/ LBFP/ LBP/ MFE1/ PBFE |
| EPHX2 | epoxide hydrolase 2 | ABHD20/ CEH/ SEH |
| FAR1 | fatty acyl-CoA reductase 1 | CSPSD/ MLSTD2/ PFCRD/ SDR10E1 |
| FAR2 | fatty acyl-CoA reductase 2 | HEL-S-81/ MLSTD1/ SDR10E2 |
| FIS1 | fission, mitochondrial 1 | CGI-135/ TTC11 |
| FNDC5 | fibronectin type III domain containing 5 | FRCP2/ irisin |
| GNPAT | glyceronephosphate O-acyltransferase | DAP-AT/ DAPAT/ DHAPAT/ RCDP2 |
| GRHPR | glyoxylate and hydroxypyruvate reductase | GLXR/ GLYD/ PH2 |
| GSTK1 | glutathione S-transferase kappa 1 | GST/ GST13-13/ GST13/ GSTK1-1/ hGSTK1 |
| HACL1 | 2-hydroxyacyl-CoA lyase 1 | 2-HPCL/ HPCL/ HPCL2/ PHYH2 |
| HAO1 | hydroxyacid oxidase 1 | GO/ GOX/ GOX1/ HAOX1 |
| HAO2 | hydroxyacid oxidase 2 | GIG16/ HAOX2 |
| HMGCL | 3-hydroxy-3-methylglutaryl-CoA lyase | HL |
| HMGCLL1 | 3-hydroxymethyl-3-methylglutaryl-CoA lyase like 1 | ERCHL/ bA418P12.1/ er-cHL |
| HSD17B4 | hydroxysteroid 17-beta dehydrogenase 4 | DBP/ MFE-2/ MFP-2/ MPF-2/ PRLTS1/ SDR8C1 |
| IDE | insulin degrading enzyme | INSULYSIN |
| IDH1 | isocitrate dehydrogenase (NADP (+)) 1 | HEL-216/ HEL-S-26/ IDCD/ IDH/ IDP/ IDPC/ PICD |
| IDH2 | isocitrate dehydrogenase (NADP (+)) 2 | D2HGA2/ ICD-M/ IDH/ IDH-2/ IDHM/ IDP/ IDPM/ mNADP-IDH |
| IDI1 | isopentenyl-diphosphate delta isomerase 1 | IPP1/ IPPI1 |
| IDI2 | isopentenyl-diphosphate delta isomerase 2 | IPPI2 |
| ISOC1 | isochorismatase domain containing 1 | CGI-111 |
| LDHA | lactate dehydrogenase A | GSD11/ HEL-S-133P/ LDHM/ PIG19 |
| LONP2 | Lon peptidase 2, peroxisomal | LONP/ LONPL/ PLON/ PSLON |
| MDH1 | malate dehydrogenase 1 | DEE88/ EIEE88/ HEL-S-32/ KAR/ MDH-s/ MDHA/ MGC:1375/ MOR2 |
| MLYCD | malonyl-CoA decarboxylase | MCD |
| MPV17 | mitochondrial inner membrane protein MPV17 | CMT2EE/ MTDPS6/ SYM1 |
| MPV17L | MPV17 mitochondrial inner membrane protein like | M-LPH/ MLPH1/ MLPH2/ MPV17L1 |
| MPV17L2 | MPV17 mitochondrial inner membrane protein like 2 | FKSG24 |
| MTARC2 | mitochondrial amidoxime reducing component 2 | MARC2/ MOSC2 |
| MVK | mevalonate kinase | LRBP/ MK/ MVLK/ POROK3 |
| NOS2 | nitric oxide synthase 2 | HEP-NOS/ INOS/ NOS/ NOS2A |
| NUDT12 | nudix hydrolase 12 | / |
| NUDT19 | nudix hydrolase 19 | RP2 |
