# Supplementary material for: A New Prognostic Indicator of Immune Microenvironment and Therapeutic Response in Lung Adenocarcinoma Based on Peroxisome-Related Genes
Source: J Immunol Res. 2022 Jul 26;2022:6084589. doi: 10.1155/2022/6084589 (PMC9346542; doi:10.1155/2022/6084589)
Supplement: Supplementary 3 — Table S2: the result of GSVA-KEGG. [file 6084589.f3.docx]

Table S2 The result of GSVA-KEGG.

| ID | Description | setSize | enrichmentScore | NES | pvalue | p.adjust | qvalues |
| --- | --- | --- | --- | --- | --- | --- | --- |
| KEGG_CELL_CYCLE | KEGG_CELL_CYCLE | 121 | 0.658177451 | 2.481037066 | 1.00E-10 | 1.84E-08 | 1.45E-08 |
| KEGG_SYSTEMIC_LUPUS_ERYTHEMATOSUS | KEGG_SYSTEMIC_LUPUS_ERYTHEMATOSUS | 108 | -0.558928218 | -1.956204078 | 2.84E-06 | 0.000261 | 0.000206053 |
| KEGG_DNA_REPLICATION | KEGG_DNA_REPLICATION | 35 | 0.733811461 | 2.203186756 | 2.31E-05 | 0.001418244 | 0.001119666 |
| KEGG_SPLICEOSOME | KEGG_SPLICEOSOME | 125 | 0.495025887 | 1.87189062 | 3.73E-05 | 0.001713642 | 0.001352875 |
| KEGG_OOCYTE_MEIOSIS | KEGG_OOCYTE_MEIOSIS | 94 | 0.516626006 | 1.883530808 | 0.000106703 | 0.00392666 | 0.003099994 |
| KEGG_PROTEASOME | KEGG_PROTEASOME | 42 | 0.657203464 | 2.043430151 | 0.000205695 | 0.006307975 | 0.004979981 |
| KEGG_P53_SIGNALING_PATHWAY | KEGG_P53_SIGNALING_PATHWAY | 65 | 0.553366946 | 1.861164401 | 0.000254283 | 0.006684022 | 0.005276859 |
| KEGG_PATHWAYS_IN_CANCER | KEGG_PATHWAYS_IN_CANCER | 288 | 0.353143891 | 1.478301319 | 0.000437761 | 0.010068505 | 0.00794882 |
| KEGG_PYRIMIDINE_METABOLISM | KEGG_PYRIMIDINE_METABOLISM | 90 | 0.493825569 | 1.779847457 | 0.000496828 | 0.010157378 | 0.008018983 |
| KEGG_ALPHA_LINOLENIC_ACID_METABOLISM | KEGG_ALPHA_LINOLENIC_ACID_METABOLISM | 15 | -0.770361193 | -1.812173269 | 0.001463325 | 0.02453795 | 0.019372066 |
| KEGG_HOMOLOGOUS_RECOMBINATION | KEGG_HOMOLOGOUS_RECOMBINATION | 26 | 0.69184199 | 1.948358159 | 0.001538922 | 0.02453795 | 0.019372066 |
| KEGG_ASTHMA | KEGG_ASTHMA | 20 | -0.72053536 | -1.827108657 | 0.001600301 | 0.02453795 | 0.019372066 |
| KEGG_ALZHEIMERS_DISEASE | KEGG_ALZHEIMERS_DISEASE | 147 | 0.394970487 | 1.535070715 | 0.002675122 | 0.037863262 | 0.029892049 |
| KEGG_INTESTINAL_IMMUNE_NETWORK_FOR_IGA_PRODUCTION | KEGG_INTESTINAL_IMMUNE_NETWORK_FOR_IGA_PRODUCTION | 36 | -0.610457748 | -1.773030448 | 0.003224484 | 0.042378933 | 0.033457052 |
| KEGG_MISMATCH_REPAIR | KEGG_MISMATCH_REPAIR | 22 | 0.69168084 | 1.892869177 | 0.003760079 | 0.04612363 | 0.036413392 |
| KEGG_DRUG_METABOLISM_CYTOCHROME_P450 | KEGG_DRUG_METABOLISM_CYTOCHROME_P450 | 41 | -0.578573728 | -1.72344845 | 0.005365191 | 0.061699692 | 0.048710283 |
| KEGG_CYTOKINE_CYTOKINE_RECEPTOR_INTERACTION | KEGG_CYTOKINE_CYTOKINE_RECEPTOR_INTERACTION | 193 | 0.364231235 | 1.462763946 | 0.00619187 | 0.067017883 | 0.052908855 |
| KEGG_ECM_RECEPTOR_INTERACTION | KEGG_ECM_RECEPTOR_INTERACTION | 71 | 0.465561547 | 1.609498533 | 0.006576789 | 0.067229401 | 0.053075843 |
| KEGG_LINOLEIC_ACID_METABOLISM | KEGG_LINOLEIC_ACID_METABOLISM | 17 | -0.701290029 | -1.71395326 | 0.008123769 | 0.077665174 | 0.061314611 |
| KEGG_ARACHIDONIC_ACID_METABOLISM | KEGG_ARACHIDONIC_ACID_METABOLISM | 42 | -0.564106036 | -1.692225387 | 0.008441867 | 0.077665174 | 0.061314611 |
| KEGG_FOCAL_ADHESION | KEGG_FOCAL_ADHESION | 177 | 0.35400204 | 1.407915226 | 0.008926144 | 0.078210022 | 0.061744754 |
| KEGG_GLYCOLYSIS_GLUCONEOGENESIS | KEGG_GLYCOLYSIS_GLUCONEOGENESIS | 47 | 0.517800122 | 1.632933485 | 0.011727626 | 0.092527201 | 0.07304779 |
| KEGG_SMALL_CELL_LUNG_CANCER | KEGG_SMALL_CELL_LUNG_CANCER | 79 | 0.421031704 | 1.471958866 | 0.012634599 | 0.092527201 | 0.07304779 |
| KEGG_RENIN_ANGIOTENSIN_SYSTEM | KEGG_RENIN_ANGIOTENSIN_SYSTEM | 15 | -0.695486849 | -1.636041235 | 0.012799838 | 0.092527201 | 0.07304779 |
| KEGG_ETHER_LIPID_METABOLISM | KEGG_ETHER_LIPID_METABOLISM | 28 | -0.607441927 | -1.66818459 | 0.012870953 | 0.092527201 | 0.07304779 |
| KEGG_LONG_TERM_DEPRESSION | KEGG_LONG_TERM_DEPRESSION | 54 | -0.504081522 | -1.589848878 | 0.013121413 | 0.092527201 | 0.07304779 |
| KEGG_FC_EPSILON_RI_SIGNALING_PATHWAY | KEGG_FC_EPSILON_RI_SIGNALING_PATHWAY | 69 | -0.472523088 | -1.536668333 | 0.013611919 | 0.092527201 | 0.07304779 |
| KEGG_PROGESTERONE_MEDIATED_OOCYTE_MATURATION | KEGG_PROGESTERONE_MEDIATED_OOCYTE_MATURATION | 73 | 0.427594923 | 1.48526791 | 0.014678888 | 0.092527201 | 0.07304779 |
| KEGG_VASCULAR_SMOOTH_MUSCLE_CONTRACTION | KEGG_VASCULAR_SMOOTH_MUSCLE_CONTRACTION | 93 | -0.435757663 | -1.491171719 | 0.014984781 | 0.092527201 | 0.07304779 |
| KEGG_CITRATE_CYCLE_TCA_CYCLE | KEGG_CITRATE_CYCLE_TCA_CYCLE | 29 | 0.58499674 | 1.688908328 | 0.015085957 | 0.092527201 | 0.07304779 |
| KEGG_REGULATION_OF_ACTIN_CYTOSKELETON | KEGG_REGULATION_OF_ACTIN_CYTOSKELETON | 175 | 0.351219289 | 1.389393708 | 0.016375977 | 0.097199348 | 0.076736327 |
| KEGG_UBIQUITIN_MEDIATED_PROTEOLYSIS | KEGG_UBIQUITIN_MEDIATED_PROTEOLYSIS | 129 | 0.36492758 | 1.391727341 | 0.017629597 | 0.101370184 | 0.080029092 |
| KEGG_RNA_DEGRADATION | KEGG_RNA_DEGRADATION | 56 | 0.467128264 | 1.530793665 | 0.019088605 | 0.106433432 | 0.084026394 |
| KEGG_MELANOMA | KEGG_MELANOMA | 54 | 0.468892389 | 1.533616221 | 0.02270839 | 0.122892461 | 0.097020364 |
| KEGG_NUCLEOTIDE_EXCISION_REPAIR | KEGG_NUCLEOTIDE_EXCISION_REPAIR | 42 | 0.519837026 | 1.616319312 | 0.027252648 | 0.143271063 | 0.113108734 |
| KEGG_PROTEIN_EXPORT | KEGG_PROTEIN_EXPORT | 24 | 0.589797669 | 1.633809739 | 0.028051395 | 0.143373796 | 0.113189839 |
| KEGG_PARKINSONS_DISEASE | KEGG_PARKINSONS_DISEASE | 120 | 0.364463076 | 1.373788578 | 0.028963059 | 0.144032509 | 0.113709876 |
| KEGG_AMINOACYL_TRNA_BIOSYNTHESIS | KEGG_AMINOACYL_TRNA_BIOSYNTHESIS | 41 | 0.50596282 | 1.563067991 | 0.032515151 | 0.157441786 | 0.124296146 |
| KEGG_PATHOGENIC_ESCHERICHIA_COLI_INFECTION | KEGG_PATHOGENIC_ESCHERICHIA_COLI_INFECTION | 49 | 0.464206939 | 1.480224334 | 0.033525985 | 0.158173878 | 0.124874114 |
| KEGG_PENTOSE_PHOSPHATE_PATHWAY | KEGG_PENTOSE_PHOSPHATE_PATHWAY | 23 | 0.557940874 | 1.535671467 | 0.039829466 | 0.178919245 | 0.141252036 |
| KEGG_VIRAL_MYOCARDITIS | KEGG_VIRAL_MYOCARDITIS | 58 | -0.46016619 | -1.461652536 | 0.041092968 | 0.178919245 | 0.141252036 |
| KEGG_ADIPOCYTOKINE_SIGNALING_PATHWAY | KEGG_ADIPOCYTOKINE_SIGNALING_PATHWAY | 59 | 0.422283383 | 1.397345397 | 0.041383485 | 0.178919245 | 0.141252036 |
| KEGG_BASE_EXCISION_REPAIR | KEGG_BASE_EXCISION_REPAIR | 34 | 0.515896576 | 1.539904958 | 0.04206793 | 0.178919245 | 0.141252036 |
| KEGG_NEUROACTIVE_LIGAND_RECEPTOR_INTERACTION | KEGG_NEUROACTIVE_LIGAND_RECEPTOR_INTERACTION | 104 | 0.358251821 | 1.326306246 | 0.042785037 | 0.178919245 | 0.141252036 |
| KEGG_DILATED_CARDIOMYOPATHY | KEGG_DILATED_CARDIOMYOPATHY | 66 | -0.431459586 | -1.393946959 | 0.04616534 | 0.188764946 | 0.149024958 |
| KEGG_GLYCEROPHOSPHOLIPID_METABOLISM | KEGG_GLYCEROPHOSPHOLIPID_METABOLISM | 68 | -0.438665175 | -1.423739706 | 0.048554324 | 0.194217296 | 0.153329444 |
