# Supplementary material for: A New Prognostic Indicator of Immune Microenvironment and Therapeutic Response in Lung Adenocarcinoma Based on Peroxisome-Related Genes
Source: J Immunol Res. 2022 Jul 26;2022:6084589. doi: 10.1155/2022/6084589 (PMC9346542; doi:10.1155/2022/6084589)
Supplement: Supplementary 4 — Table S3: the result of GSVA-GO. [file 6084589.f4.docx]

Table S3 The result of GSVA-GO.

| ID | Description | setSize | enrichmentScore | NES | pvalue | p.adjust | qvalues |
| --- | --- | --- | --- | --- | --- | --- | --- |
| GOBP_AXONEMAL_DYNEIN_COMPLEX_ASSEMBLY | GOBP_AXONEMAL_DYNEIN_COMPLEX_ASSEMBLY | 30 | -0.870901522 | -2.440929119 | 1.00E-10 | 2.07E-08 | 1.75E-08 |
| GOBP_AXONEME_ASSEMBLY | GOBP_AXONEME_ASSEMBLY | 59 | -0.788228404 | -2.493428021 | 1.00E-10 | 2.07E-08 | 1.75E-08 |
| GOBP_CELL_CYCLE_G2_M_PHASE_TRANSITION | GOBP_CELL_CYCLE_G2_M_PHASE_TRANSITION | 260 | 0.50914018 | 2.135198327 | 1.00E-10 | 2.07E-08 | 1.75E-08 |
| GOBP_CHROMOSOME_SEGREGATION | GOBP_CHROMOSOME_SEGREGATION | 291 | 0.604163566 | 2.560997405 | 1.00E-10 | 2.07E-08 | 1.75E-08 |
| GOBP_CILIUM_MOVEMENT | GOBP_CILIUM_MOVEMENT | 104 | -0.715654623 | -2.456461771 | 1.00E-10 | 2.07E-08 | 1.75E-08 |
| GOBP_CORNIFICATION | GOBP_CORNIFICATION | 61 | 0.832445342 | 2.816914685 | 1.00E-10 | 2.07E-08 | 1.75E-08 |
| GOBP_DNA_DEPENDENT_DNA_REPLICATION | GOBP_DNA_DEPENDENT_DNA_REPLICATION | 147 | 0.649010568 | 2.525402273 | 1.00E-10 | 2.07E-08 | 1.75E-08 |
| GOBP_DNA_REPLICATION | GOBP_DNA_REPLICATION | 262 | 0.581266276 | 2.436926988 | 1.00E-10 | 2.07E-08 | 1.75E-08 |
| GOBP_EPIDERMAL_CELL_DIFFERENTIATION | GOBP_EPIDERMAL_CELL_DIFFERENTIATION | 178 | 0.660220115 | 2.635511672 | 1.00E-10 | 2.07E-08 | 1.75E-08 |
| GOBP_EPIDERMIS_DEVELOPMENT | GOBP_EPIDERMIS_DEVELOPMENT | 272 | 0.587867271 | 2.47639713 | 1.00E-10 | 2.07E-08 | 1.75E-08 |
| GOBP_KERATINIZATION | GOBP_KERATINIZATION | 69 | 0.824166843 | 2.85717824 | 1.00E-10 | 2.07E-08 | 1.75E-08 |
| GOBP_KERATINOCYTE_DIFFERENTIATION | GOBP_KERATINOCYTE_DIFFERENTIATION | 136 | 0.711639446 | 2.740853835 | 1.00E-10 | 2.07E-08 | 1.75E-08 |
| GOBP_MITOTIC_NUCLEAR_DIVISION | GOBP_MITOTIC_NUCLEAR_DIVISION | 274 | 0.600967656 | 2.541108232 | 1.00E-10 | 2.07E-08 | 1.75E-08 |
| GOBP_MITOTIC_SISTER_CHROMATID_SEGREGATION | GOBP_MITOTIC_SISTER_CHROMATID_SEGREGATION | 155 | 0.670905607 | 2.660135968 | 1.00E-10 | 2.07E-08 | 1.75E-08 |
| GOBP_NUCLEAR_CHROMOSOME_SEGREGATION | GOBP_NUCLEAR_CHROMOSOME_SEGREGATION | 231 | 0.618463729 | 2.562911324 | 1.00E-10 | 2.07E-08 | 1.75E-08 |
| GOBP_ORGANELLE_FISSION | GOBP_ORGANELLE_FISSION | 406 | 0.551010104 | 2.425255162 | 1.00E-10 | 2.07E-08 | 1.75E-08 |
| GOBP_SISTER_CHROMATID_SEGREGATION | GOBP_SISTER_CHROMATID_SEGREGATION | 184 | 0.64153563 | 2.588814559 | 1.00E-10 | 2.07E-08 | 1.75E-08 |
| GOBP_SKIN_DEVELOPMENT | GOBP_SKIN_DEVELOPMENT | 236 | 0.63532919 | 2.645075075 | 1.00E-10 | 2.07E-08 | 1.75E-08 |
| GOCC_CHROMOSOMAL_REGION | GOCC_CHROMOSOMAL_REGION | 316 | 0.496197095 | 2.122831336 | 1.00E-10 | 2.07E-08 | 1.75E-08 |
| GOCC_CHROMOSOME_CENTROMERIC_REGION | GOCC_CHROMOSOME_CENTROMERIC_REGION | 186 | 0.621733855 | 2.510324222 | 1.00E-10 | 2.07E-08 | 1.75E-08 |
| GOCC_CILIARY_PLASM | GOCC_CILIARY_PLASM | 106 | -0.742562127 | -2.570070563 | 1.00E-10 | 2.07E-08 | 1.75E-08 |
| GOCC_CILIUM | GOCC_CILIUM | 479 | -0.502872961 | -2.054648064 | 1.00E-10 | 2.07E-08 | 1.75E-08 |
| GOCC_CONDENSED_CHROMOSOME | GOCC_CONDENSED_CHROMOSOME | 187 | 0.621014456 | 2.511391354 | 1.00E-10 | 2.07E-08 | 1.75E-08 |
| GOCC_CONDENSED_CHROMOSOME_CENTROMERIC_REGION | GOCC_CONDENSED_CHROMOSOME_CENTROMERIC_REGION | 112 | 0.688756343 | 2.580688961 | 1.00E-10 | 2.07E-08 | 1.75E-08 |
| GOCC_INTERMEDIATE_FILAMENT | GOCC_INTERMEDIATE_FILAMENT | 71 | 0.741589819 | 2.584086975 | 1.00E-10 | 2.07E-08 | 1.75E-08 |
| GOCC_INTERMEDIATE_FILAMENT_CYTOSKELETON | GOCC_INTERMEDIATE_FILAMENT_CYTOSKELETON | 108 | 0.6713976 | 2.52240581 | 1.00E-10 | 2.07E-08 | 1.75E-08 |
| GOCC_KINETOCHORE | GOCC_KINETOCHORE | 133 | 0.647010288 | 2.487584241 | 1.00E-10 | 2.07E-08 | 1.75E-08 |
| GOCC_MOTILE_CILIUM | GOCC_MOTILE_CILIUM | 132 | -0.658916734 | -2.348066357 | 1.00E-10 | 2.07E-08 | 1.75E-08 |
| GOCC_PLASMA_MEMBRANE_BOUNDED_CELL_PROJECTION_CYTOPLASM | GOCC_PLASMA_MEMBRANE_BOUNDED_CELL_PROJECTION_CYTOPLASM | 178 | -0.584045273 | -2.167646325 | 1.00E-10 | 2.07E-08 | 1.75E-08 |
| GOMF_RECEPTOR_REGULATOR_ACTIVITY | GOMF_RECEPTOR_REGULATOR_ACTIVITY | 315 | 0.509707778 | 2.184676682 | 1.00E-10 | 2.07E-08 | 1.75E-08 |
| GOBP_MICROTUBULE_CYTOSKELETON_ORGANIZATION_INVOLVED_IN_MITOSIS | GOBP_MICROTUBULE_CYTOSKELETON_ORGANIZATION_INVOLVED_IN_MITOSIS | 135 | 0.612214556 | 2.35739757 | 1.03E-10 | 2.07E-08 | 1.75E-08 |
| GOBP_CELL_CYCLE_CHECKPOINT | GOBP_CELL_CYCLE_CHECKPOINT | 197 | 0.545161251 | 2.199012182 | 1.32E-10 | 2.55E-08 | 2.17E-08 |
| GOBP_REGULATION_OF_CELL_CYCLE_PHASE_TRANSITION | GOBP_REGULATION_OF_CELL_CYCLE_PHASE_TRANSITION | 420 | 0.441052372 | 1.945196129 | 1.76E-10 | 3.32E-08 | 2.81E-08 |
| GOBP_MEIOTIC_CELL_CYCLE | GOBP_MEIOTIC_CELL_CYCLE | 174 | 0.558650362 | 2.24154672 | 3.06E-10 | 5.59E-08 | 4.74E-08 |
| GOBP_MICROTUBULE_BUNDLE_FORMATION | GOBP_MICROTUBULE_BUNDLE_FORMATION | 89 | -0.677063518 | -2.273922018 | 8.35E-10 | 1.48E-07 | 1.26E-07 |
| GOBP_MEIOTIC_CELL_CYCLE_PROCESS | GOBP_MEIOTIC_CELL_CYCLE_PROCESS | 130 | 0.60833806 | 2.344585152 | 1.21E-09 | 2.09E-07 | 1.77E-07 |
| GOBP_REGULATION_OF_CELL_CYCLE_G2_M_PHASE_TRANSITION | GOBP_REGULATION_OF_CELL_CYCLE_G2_M_PHASE_TRANSITION | 207 | 0.524339153 | 2.12225603 | 1.55E-09 | 2.61E-07 | 2.21E-07 |
| GOBP_CILIUM_ORGANIZATION | GOBP_CILIUM_ORGANIZATION | 362 | -0.467018278 | -1.858836211 | 2.24E-09 | 3.66E-07 | 3.10E-07 |
| GOCC_SPINDLE | GOCC_SPINDLE | 360 | 0.438722327 | 1.900957436 | 2.93E-09 | 4.67E-07 | 3.96E-07 |
| GOCC_KERATIN_FILAMENT | GOCC_KERATIN_FILAMENT | 23 | 0.873860411 | 2.374784573 | 6.98E-09 | 1.08E-06 | 9.19E-07 |
| GOBP_POSITIVE_REGULATION_OF_CELL_CYCLE_PROCESS | GOBP_POSITIVE_REGULATION_OF_CELL_CYCLE_PROCESS | 250 | 0.480556505 | 2.007925754 | 7.60E-09 | 1.15E-06 | 9.72E-07 |
| GOCC_9PLUS2_MOTILE_CILIUM | GOCC_9PLUS2_MOTILE_CILIUM | 73 | -0.683447943 | -2.202711423 | 7.75E-09 | 1.15E-06 | 9.72E-07 |
| GOCC_CYTOPLASMIC_REGION | GOCC_CYTOPLASMIC_REGION | 209 | -0.530381076 | -1.998891047 | 1.25E-08 | 1.81E-06 | 1.54E-06 |
| GOBP_MITOTIC_SPINDLE_ORGANIZATION | GOBP_MITOTIC_SPINDLE_ORGANIZATION | 113 | 0.602099541 | 2.260556051 | 1.54E-08 | 2.17E-06 | 1.84E-06 |
| GOBP_REGULATION_OF_MITOTIC_NUCLEAR_DIVISION | GOBP_REGULATION_OF_MITOTIC_NUCLEAR_DIVISION | 98 | 0.6199497 | 2.290224308 | 1.80E-08 | 2.48E-06 | 2.10E-06 |
| GOBP_POST_TRANSLATIONAL_PROTEIN_MODIFICATION | GOBP_POST_TRANSLATIONAL_PROTEIN_MODIFICATION | 319 | 0.435582289 | 1.860034495 | 2.97E-08 | 4.01E-06 | 3.40E-06 |
| GOBP_CELL_CYCLE_DNA_REPLICATION | GOBP_CELL_CYCLE_DNA_REPLICATION | 61 | 0.708879529 | 2.398779902 | 3.55E-08 | 4.69E-06 | 3.97E-06 |
| GOBP_NEGATIVE_REGULATION_OF_MITOTIC_CELL_CYCLE | GOBP_NEGATIVE_REGULATION_OF_MITOTIC_CELL_CYCLE | 283 | 0.455946224 | 1.934713223 | 4.62E-08 | 5.86E-06 | 4.97E-06 |
| GOBP_NEGATIVE_REGULATION_OF_CELL_CYCLE_PROCESS | GOBP_NEGATIVE_REGULATION_OF_CELL_CYCLE_PROCESS | 314 | 0.444525855 | 1.900213935 | 4.62E-08 | 5.86E-06 | 4.97E-06 |
| GOMF_CADHERIN_BINDING | GOMF_CADHERIN_BINDING | 312 | 0.438236244 | 1.877136836 | 5.00E-08 | 6.21E-06 | 5.27E-06 |
| GOCC_DYNEIN_COMPLEX | GOCC_DYNEIN_COMPLEX | 47 | -0.739412167 | -2.259071961 | 5.38E-08 | 6.56E-06 | 5.56E-06 |
| GOBP_MITOTIC_CELL_CYCLE_CHECKPOINT | GOBP_MITOTIC_CELL_CYCLE_CHECKPOINT | 151 | 0.546686003 | 2.155595931 | 6.73E-08 | 8.04E-06 | 6.82E-06 |
| GOBP_RIBOSOME_BIOGENESIS | GOBP_RIBOSOME_BIOGENESIS | 301 | 0.442318489 | 1.883442536 | 7.02E-08 | 8.23E-06 | 6.98E-06 |
| GOCC_ENDOPLASMIC_RETICULUM_LUMEN | GOCC_ENDOPLASMIC_RETICULUM_LUMEN | 261 | 0.455071387 | 1.906981942 | 7.35E-08 | 8.45E-06 | 7.17E-06 |
| GOBP_REGULATION_OF_NUCLEAR_DIVISION | GOBP_REGULATION_OF_NUCLEAR_DIVISION | 114 | 0.595056168 | 2.245549385 | 7.95E-08 | 8.98E-06 | 7.61E-06 |
| GOMF_HORMONE_ACTIVITY | GOMF_HORMONE_ACTIVITY | 51 | 0.717984464 | 2.339554626 | 9.98E-08 | 1.11E-05 | 9.39E-06 |
| GOBP_CELL_CYCLE_G1_S_PHASE_TRANSITION | GOBP_CELL_CYCLE_G1_S_PHASE_TRANSITION | 249 | 0.459449785 | 1.916564915 | 1.88E-07 | 2.05E-05 | 1.73E-05 |
| GOMF_CELL_ADHESION_MOLECULE_BINDING | GOMF_CELL_ADHESION_MOLECULE_BINDING | 483 | 0.377977927 | 1.663071074 | 1.93E-07 | 2.07E-05 | 1.75E-05 |
| GOBP_CILIUM_OR_FLAGELLUM_DEPENDENT_CELL_MOTILITY | GOBP_CILIUM_OR_FLAGELLUM_DEPENDENT_CELL_MOTILITY | 72 | -0.656076054 | -2.117546138 | 1.97E-07 | 2.08E-05 | 1.76E-05 |
| GOBP_NCRNA_METABOLIC_PROCESS | GOBP_NCRNA_METABOLIC_PROCESS | 460 | 0.38418829 | 1.692687036 | 2.03E-07 | 2.10E-05 | 1.78E-05 |
| GOBP_POSITIVE_REGULATION_OF_CELL_CYCLE | GOBP_POSITIVE_REGULATION_OF_CELL_CYCLE | 334 | 0.423935755 | 1.825063563 | 2.70E-07 | 2.75E-05 | 2.33E-05 |
| GOBP_RESPONSE_TO_EXTRACELLULAR_STIMULUS | GOBP_RESPONSE_TO_EXTRACELLULAR_STIMULUS | 389 | 0.391894389 | 1.710029082 | 3.74E-07 | 3.74E-05 | 3.17E-05 |
| GOBP_REPRODUCTIVE_SYSTEM_DEVELOPMENT | GOBP_REPRODUCTIVE_SYSTEM_DEVELOPMENT | 326 | 0.421342828 | 1.802748185 | 3.86E-07 | 3.81E-05 | 3.23E-05 |
| GOBP_ATTACHMENT_OF_SPINDLE_MICROTUBULES_TO_KINETOCHORE | GOBP_ATTACHMENT_OF_SPINDLE_MICROTUBULES_TO_KINETOCHORE | 34 | 0.7783333 | 2.320672461 | 3.93E-07 | 3.81E-05 | 3.23E-05 |
| GOBP_REGULATION_OF_CHROMOSOME_ORGANIZATION | GOBP_REGULATION_OF_CHROMOSOME_ORGANIZATION | 253 | 0.450798649 | 1.880129575 | 4.15E-07 | 3.97E-05 | 3.36E-05 |
| GOBP_NEGATIVE_REGULATION_OF_CELL_CYCLE_PHASE_TRANSITION | GOBP_NEGATIVE_REGULATION_OF_CELL_CYCLE_PHASE_TRANSITION | 234 | 0.4556114 | 1.888099609 | 4.51E-07 | 4.24E-05 | 3.59E-05 |
| GOBP_CHROMOSOME_SEPARATION | GOBP_CHROMOSOME_SEPARATION | 84 | 0.611155068 | 2.187667614 | 5.87E-07 | 5.42E-05 | 4.60E-05 |
| GOBP_MEIOSIS_I_CELL_CYCLE_PROCESS | GOBP_MEIOSIS_I_CELL_CYCLE_PROCESS | 84 | 0.611041521 | 2.187261165 | 5.94E-07 | 5.42E-05 | 4.60E-05 |
| GOBP_SPINDLE_ORGANIZATION | GOBP_SPINDLE_ORGANIZATION | 173 | 0.505472156 | 2.02920835 | 6.34E-07 | 5.71E-05 | 4.84E-05 |
| GOCC_SPINDLE_POLE | GOCC_SPINDLE_POLE | 158 | 0.513580626 | 2.037126744 | 7.59E-07 | 6.74E-05 | 5.71E-05 |
| GOBP_MICROTUBULE_BASED_MOVEMENT | GOBP_MICROTUBULE_BASED_MOVEMENT | 291 | -0.44928722 | -1.759406688 | 7.70E-07 | 6.74E-05 | 5.71E-05 |
| GOBP_EMBRYONIC_MORPHOGENESIS | GOBP_EMBRYONIC_MORPHOGENESIS | 465 | 0.374622221 | 1.650239081 | 7.90E-07 | 6.81E-05 | 5.78E-05 |
| GOBP_DNA_GEOMETRIC_CHANGE | GOBP_DNA_GEOMETRIC_CHANGE | 106 | 0.574975011 | 2.149508731 | 8.33E-07 | 7.09E-05 | 6.01E-05 |
| GOCC_AXONEMAL_DYNEIN_COMPLEX | GOCC_AXONEMAL_DYNEIN_COMPLEX | 18 | -0.863736966 | -2.144486358 | 1.02E-06 | 8.55E-05 | 7.25E-05 |
| GOBP_REGULATION_OF_CHROMOSOME_SEGREGATION | GOBP_REGULATION_OF_CHROMOSOME_SEGREGATION | 80 | 0.625364482 | 2.244073908 | 1.10E-06 | 9.08E-05 | 7.69E-05 |
| GOMF_HELICASE_ACTIVITY | GOMF_HELICASE_ACTIVITY | 147 | 0.514512483 | 2.002049051 | 1.33E-06 | 0.00010833 | 9.18E-05 |
| GOBP_REGULATION_OF_DNA_REPLICATION | GOBP_REGULATION_OF_DNA_REPLICATION | 101 | 0.56902584 | 2.120349038 | 1.51E-06 | 0.00012201 | 0.000103419 |
| GOBP_RECOMBINATIONAL_REPAIR | GOBP_RECOMBINATIONAL_REPAIR | 123 | 0.539268798 | 2.054419399 | 1.79E-06 | 0.000142414 | 0.000120714 |
| GOBP_METAPHASE_ANAPHASE_TRANSITION_OF_CELL_CYCLE | GOBP_METAPHASE_ANAPHASE_TRANSITION_OF_CELL_CYCLE | 61 | 0.651635553 | 2.205071815 | 2.89E-06 | 0.000227033 | 0.00019244 |
| GOMF_DNA_HELICASE_ACTIVITY | GOMF_DNA_HELICASE_ACTIVITY | 69 | 0.639880635 | 2.218304511 | 3.08E-06 | 0.000239018 | 0.000202598 |
| GOBP_INNER_DYNEIN_ARM_ASSEMBLY | GOBP_INNER_DYNEIN_ARM_ASSEMBLY | 14 | -0.881779468 | -2.098841927 | 3.52E-06 | 0.000269088 | 0.000228087 |
| GOCC_CONDENSED_CHROMOSOME_OUTER_KINETOCHORE | GOCC_CONDENSED_CHROMOSOME_OUTER_KINETOCHORE | 13 | 0.892451648 | 2.176657923 | 3.57E-06 | 0.000269088 | 0.000228087 |
| GOBP_SIGNAL_TRANSDUCTION_BY_P53_CLASS_MEDIATOR | GOBP_SIGNAL_TRANSDUCTION_BY_P53_CLASS_MEDIATOR | 249 | 0.429430919 | 1.791343165 | 3.59E-06 | 0.000269088 | 0.000228087 |
| GOCC_MITOCHONDRIAL_PROTEIN_CONTAINING_COMPLEX | GOCC_MITOCHONDRIAL_PROTEIN_CONTAINING_COMPLEX | 257 | 0.422772074 | 1.763849115 | 3.94E-06 | 0.000291458 | 0.000247048 |
| GOBP_RESPONSE_TO_IONIZING_RADIATION | GOBP_RESPONSE_TO_IONIZING_RADIATION | 136 | 0.509949084 | 1.964050629 | 4.23E-06 | 0.000309034 | 0.000261946 |
| GOCC_PEPTIDASE_COMPLEX | GOCC_PEPTIDASE_COMPLEX | 86 | 0.577772161 | 2.085874603 | 4.59E-06 | 0.0003318 | 0.000281243 |
| GOBP_DNA_RECOMBINATION | GOBP_DNA_RECOMBINATION | 249 | 0.425873168 | 1.77650224 | 4.97E-06 | 0.000350047 | 0.000296709 |
| GOBP_DNA_INTEGRITY_CHECKPOINT | GOBP_DNA_INTEGRITY_CHECKPOINT | 148 | 0.506382986 | 1.974104263 | 5.02E-06 | 0.000350047 | 0.000296709 |
| GOBP_REGULATION_OF_SIGNAL_TRANSDUCTION_BY_P53_CLASS_MEDIATOR | GOBP_REGULATION_OF_SIGNAL_TRANSDUCTION_BY_P53_CLASS_MEDIATOR | 166 | 0.478530278 | 1.900360299 | 5.06E-06 | 0.000350047 | 0.000296709 |
| GOBP_SPECIFICATION_OF_SYMMETRY | GOBP_SPECIFICATION_OF_SYMMETRY | 99 | -0.571195164 | -1.943925802 | 5.07E-06 | 0.000350047 | 0.000296709 |
| GOBP_SEQUESTERING_OF_METAL_ION | GOBP_SEQUESTERING_OF_METAL_ION | 10 | 0.932186687 | 2.078953175 | 5.13E-06 | 0.000350386 | 0.000296997 |
| GOBP_PROTEIN_LOCALIZATION_TO_CHROMOSOME | GOBP_PROTEIN_LOCALIZATION_TO_CHROMOSOME | 89 | 0.583497385 | 2.106521309 | 5.32E-06 | 0.000359585 | 0.000304795 |
| GOBP_SKELETAL_SYSTEM_DEVELOPMENT | GOBP_SKELETAL_SYSTEM_DEVELOPMENT | 391 | 0.369053702 | 1.61807562 | 5.49E-06 | 0.000366921 | 0.000311012 |
| GOMF_SINGLE_STRANDED_DNA_HELICASE_ACTIVITY | GOMF_SINGLE_STRANDED_DNA_HELICASE_ACTIVITY | 19 | 0.828190854 | 2.18780498 | 5.56E-06 | 0.000367471 | 0.000311479 |
| GOBP_REGULATION_OF_CHROMOSOME_SEPARATION | GOBP_REGULATION_OF_CHROMOSOME_SEPARATION | 66 | 0.638402671 | 2.189687813 | 5.63E-06 | 0.000367938 | 0.000311874 |
| GOBP_AGING | GOBP_AGING | 257 | 0.418594159 | 1.746418417 | 6.35E-06 | 0.000410989 | 0.000348366 |
| GOBP_NEGATIVE_REGULATION_OF_CHROMOSOME_ORGANIZATION | GOBP_NEGATIVE_REGULATION_OF_CHROMOSOME_ORGANIZATION | 82 | 0.588636777 | 2.11713787 | 6.62E-06 | 0.000424319 | 0.000359665 |
| GOBP_DNA_REPLICATION_INITIATION | GOBP_DNA_REPLICATION_INITIATION | 37 | 0.729517865 | 2.214760578 | 7.16E-06 | 0.000453804 | 0.000384657 |
| GOBP_REGULATION_OF_RESPONSE_TO_DNA_DAMAGE_STIMULUS | GOBP_REGULATION_OF_RESPONSE_TO_DNA_DAMAGE_STIMULUS | 206 | 0.44737022 | 1.806409125 | 7.48E-06 | 0.000469546 | 0.000398 |
| GOBP_NCRNA_PROCESSING | GOBP_NCRNA_PROCESSING | 381 | 0.371017814 | 1.618663114 | 7.92E-06 | 0.000491164 | 0.000416324 |
| GOCC_CORNIFIED_ENVELOPE | GOCC_CORNIFIED_ENVELOPE | 29 | 0.777783336 | 2.240271786 | 8.02E-06 | 0.000491164 | 0.000416324 |
| GOBP_SPLICEOSOMAL_TRI_SNRNP_COMPLEX_ASSEMBLY | GOBP_SPLICEOSOMAL_TRI_SNRNP_COMPLEX_ASSEMBLY | 22 | -0.806398465 | -2.129074589 | 8.06E-06 | 0.000491164 | 0.000416324 |
| GOBP_NEGATIVE_REGULATION_OF_NUCLEAR_DIVISION | GOBP_NEGATIVE_REGULATION_OF_NUCLEAR_DIVISION | 50 | 0.683449706 | 2.221959519 | 8.42E-06 | 0.000508082 | 0.000430665 |
| GOBP_GRANULOCYTE_MIGRATION | GOBP_GRANULOCYTE_MIGRATION | 131 | 0.50550994 | 1.951460362 | 1.18E-05 | 0.00070378 | 0.000596544 |
| GOBP_REGULATION_OF_DNA_DEPENDENT_DNA_REPLICATION | GOBP_REGULATION_OF_DNA_DEPENDENT_DNA_REPLICATION | 46 | 0.681194442 | 2.16688143 | 1.32E-05 | 0.000778613 | 0.000659974 |
| GOBP_CHROMOSOME_LOCALIZATION | GOBP_CHROMOSOME_LOCALIZATION | 71 | 0.605729033 | 2.110676906 | 1.35E-05 | 0.000789891 | 0.000669535 |
| GOBP_OUTER_DYNEIN_ARM_ASSEMBLY | GOBP_OUTER_DYNEIN_ARM_ASSEMBLY | 17 | -0.842758751 | -2.094372071 | 1.37E-05 | 0.000798021 | 0.000676425 |
| GOBP_EMBRYONIC_SKELETAL_SYSTEM_DEVELOPMENT | GOBP_EMBRYONIC_SKELETAL_SYSTEM_DEVELOPMENT | 91 | 0.556248883 | 2.024356323 | 1.71E-05 | 0.000986066 | 0.000835818 |
| GOCC_MIDBODY | GOCC_MIDBODY | 182 | 0.458462603 | 1.837673919 | 1.73E-05 | 0.000986066 | 0.000835818 |
| GOBP_ATP_METABOLIC_PROCESS | GOBP_ATP_METABOLIC_PROCESS | 278 | 0.399457446 | 1.694363944 | 1.81E-05 | 0.001025036 | 0.00086885 |
| GOBP_DNA_REPLICATION_CHECKPOINT | GOBP_DNA_REPLICATION_CHECKPOINT | 15 | 0.846470285 | 2.109498404 | 1.86E-05 | 0.0010436 | 0.000884585 |
| GOBP_RDNA_HETEROCHROMATIN_ASSEMBLY | GOBP_RDNA_HETEROCHROMATIN_ASSEMBLY | 31 | -0.724504341 | -2.041274248 | 1.94E-05 | 0.001077567 | 0.000913376 |
| GOBP_KINETOCHORE_ORGANIZATION | GOBP_KINETOCHORE_ORGANIZATION | 22 | 0.797495019 | 2.157690418 | 2.05E-05 | 0.001123343 | 0.000952178 |
| GOBP_REGULATION_OF_MITOTIC_SISTER_CHROMATID_SEGREGATION | GOBP_REGULATION_OF_MITOTIC_SISTER_CHROMATID_SEGREGATION | 42 | 0.691804353 | 2.14095698 | 2.07E-05 | 0.001123343 | 0.000952178 |
| GOCC_EXTERNAL_ENCAPSULATING_STRUCTURE | GOCC_EXTERNAL_ENCAPSULATING_STRUCTURE | 422 | 0.357033979 | 1.577414826 | 2.08E-05 | 0.001123343 | 0.000952178 |
| GOBP_CELL_CYCLE_ARREST | GOBP_CELL_CYCLE_ARREST | 214 | 0.432428018 | 1.752438801 | 2.14E-05 | 0.001146861 | 0.000972112 |
| GOCC_PRERIBOSOME | GOCC_PRERIBOSOME | 76 | 0.578476074 | 2.057081676 | 2.24E-05 | 0.00119022 | 0.001008864 |
| GOBP_PROTEIN_LOCALIZATION_TO_CHROMOSOME_CENTROMERIC_REGION | GOBP_PROTEIN_LOCALIZATION_TO_CHROMOSOME_CENTROMERIC_REGION | 25 | 0.777671637 | 2.141258207 | 2.31E-05 | 0.001217379 | 0.001031885 |
| GOMF_CATALYTIC_ACTIVITY_ACTING_ON_DNA | GOMF_CATALYTIC_ACTIVITY_ACTING_ON_DNA | 178 | 0.445206937 | 1.777207408 | 2.34E-05 | 0.001219161 | 0.001033396 |
| GOBP_REGULATION_OF_DNA_METABOLIC_PROCESS | GOBP_REGULATION_OF_DNA_METABOLIC_PROCESS | 313 | 0.378330694 | 1.620434742 | 2.56E-05 | 0.001327193 | 0.001124967 |
| GOMF_CYTOKINE_ACTIVITY | GOMF_CYTOKINE_ACTIVITY | 149 | 0.47612607 | 1.863103456 | 2.60E-05 | 0.001331717 | 0.001128801 |
| GOBP_METAPHASE_PLATE_CONGRESSION | GOBP_METAPHASE_PLATE_CONGRESSION | 61 | 0.617443453 | 2.089369047 | 2.61E-05 | 0.001331717 | 0.001128801 |
| GOBP_GLAND_DEVELOPMENT | GOBP_GLAND_DEVELOPMENT | 350 | 0.360266454 | 1.568055228 | 2.70E-05 | 0.001364905 | 0.001156932 |
| GOBP_EMBRYONIC_SKELETAL_SYSTEM_MORPHOGENESIS | GOBP_EMBRYONIC_SKELETAL_SYSTEM_MORPHOGENESIS | 68 | 0.610332557 | 2.102443738 | 2.79E-05 | 0.001399575 | 0.00118632 |
| GOCC_MITOTIC_SPINDLE | GOCC_MITOTIC_SPINDLE | 148 | 0.482682736 | 1.881710235 | 2.98E-05 | 0.00147962 | 0.001254168 |
| GOBP_EMBRYONIC_ORGAN_DEVELOPMENT | GOBP_EMBRYONIC_ORGAN_DEVELOPMENT | 331 | 0.375493577 | 1.61218138 | 3.43E-05 | 0.001692781 | 0.001434849 |
| GOBP_NEGATIVE_REGULATION_OF_CELL_CYCLE_G2_M_PHASE_TRANSITION | GOBP_NEGATIVE_REGULATION_OF_CELL_CYCLE_G2_M_PHASE_TRANSITION | 104 | 0.526953374 | 1.974259172 | 3.52E-05 | 0.001721343 | 0.00145906 |
| GOBP_LONG_TERM_MEMORY | GOBP_LONG_TERM_MEMORY | 24 | -0.758968482 | -2.032761863 | 3.91E-05 | 0.001898192 | 0.001608961 |
| GOBP_FORMATION_OF_PRIMARY_GERM_LAYER | GOBP_FORMATION_OF_PRIMARY_GERM_LAYER | 95 | 0.528845655 | 1.954603389 | 4.12E-05 | 0.001982177 | 0.00168015 |
| GOBP_MITOTIC_SPINDLE_ASSEMBLY | GOBP_MITOTIC_SPINDLE_ASSEMBLY | 63 | 0.617934423 | 2.106991428 | 4.29E-05 | 0.002048211 | 0.001736122 |
| GOBP_RESPONSE_TO_INTERLEUKIN_1 | GOBP_RESPONSE_TO_INTERLEUKIN_1 | 182 | 0.445500585 | 1.785717747 | 5.25E-05 | 0.002491501 | 0.002111867 |
| GOBP_SISTER_CHROMATID_COHESION | GOBP_SISTER_CHROMATID_COHESION | 56 | 0.624179877 | 2.076027874 | 5.55E-05 | 0.002613379 | 0.002215175 |
| GOBP_PLACENTA_DEVELOPMENT | GOBP_PLACENTA_DEVELOPMENT | 118 | 0.502986743 | 1.911536516 | 5.62E-05 | 0.002623456 | 0.002223716 |
| GOBP_NEGATIVE_REGULATION_OF_MEGAKARYOCYTE_DIFFERENTIATION | GOBP_NEGATIVE_REGULATION_OF_MEGAKARYOCYTE_DIFFERENTIATION | 15 | -0.845608092 | -2.04631311 | 5.97E-05 | 0.002768851 | 0.002346957 |
| GOBP_REGULATION_OF_TRANSCRIPTION_INVOLVED_IN_G1_S_TRANSITION_OF_MITOTIC_CELL_CYCLE | GOBP_REGULATION_OF_TRANSCRIPTION_INVOLVED_IN_G1_S_TRANSITION_OF_MITOTIC_CELL_CYCLE | 33 | 0.714522711 | 2.137497472 | 6.13E-05 | 0.002820317 | 0.002390581 |
| GOBP_PROTEIN_LOCALIZATION_TO_KINETOCHORE | GOBP_PROTEIN_LOCALIZATION_TO_KINETOCHORE | 19 | 0.786554924 | 2.07781669 | 6.59E-05 | 0.003009197 | 0.002550681 |
| GOBP_RESPONSE_TO_HEAT | GOBP_RESPONSE_TO_HEAT | 145 | 0.471620724 | 1.831646587 | 6.89E-05 | 0.003126667 | 0.002650252 |
| GOCC_ENDOPEPTIDASE_COMPLEX | GOCC_ENDOPEPTIDASE_COMPLEX | 66 | 0.598529376 | 2.052924496 | 7.14E-05 | 0.003203185 | 0.002715111 |
| GOBP_GRANULOCYTE_CHEMOTAXIS | GOBP_GRANULOCYTE_CHEMOTAXIS | 112 | 0.511759899 | 1.917504116 | 7.17E-05 | 0.003203185 | 0.002715111 |
| GOBP_RESPONSE_TO_OXYGEN_LEVELS | GOBP_RESPONSE_TO_OXYGEN_LEVELS | 335 | 0.368301119 | 1.585621433 | 7.43E-05 | 0.00329926 | 0.002796547 |
| GOBP_CHROMOSOME_ORGANIZATION_INVOLVED_IN_MEIOTIC_CELL_CYCLE | GOBP_CHROMOSOME_ORGANIZATION_INVOLVED_IN_MEIOTIC_CELL_CYCLE | 41 | 0.655978111 | 2.026422261 | 7.50E-05 | 0.003302971 | 0.002799692 |
| GOCC_CELL_SUBSTRATE_JUNCTION | GOCC_CELL_SUBSTRATE_JUNCTION | 408 | 0.34166689 | 1.503512687 | 7.66E-05 | 0.003353527 | 0.002842545 |
| GOBP_NEGATIVE_REGULATION_OF_METAPHASE_ANAPHASE_TRANSITION_OF_CELL_CYCLE | GOBP_NEGATIVE_REGULATION_OF_METAPHASE_ANAPHASE_TRANSITION_OF_CELL_CYCLE | 39 | 0.687390556 | 2.095624877 | 7.99E-05 | 0.003452956 | 0.002926824 |
| GOBP_DOUBLE_STRAND_BREAK_REPAIR | GOBP_DOUBLE_STRAND_BREAK_REPAIR | 236 | 0.400256107 | 1.666391956 | 8.08E-05 | 0.003452956 | 0.002926824 |
| GOBP_REGULATION_OF_CYCLIN_DEPENDENT_PROTEIN_KINASE_ACTIVITY | GOBP_REGULATION_OF_CYCLIN_DEPENDENT_PROTEIN_KINASE_ACTIVITY | 95 | 0.517888914 | 1.914107484 | 8.11E-05 | 0.003452956 | 0.002926824 |
| GOBP_POSITIVE_REGULATION_OF_MACROPHAGE_DERIVED_FOAM_CELL_DIFFERENTIATION | GOBP_POSITIVE_REGULATION_OF_MACROPHAGE_DERIVED_FOAM_CELL_DIFFERENTIATION | 17 | -0.81256208 | -2.019329167 | 8.11E-05 | 0.003452956 | 0.002926824 |
| GOMF_PROTEASE_BINDING | GOMF_PROTEASE_BINDING | 117 | 0.489717908 | 1.858427186 | 9.22E-05 | 0.003898022 | 0.003304074 |
| GOBP_MEIOTIC_CHROMOSOME_SEGREGATION | GOBP_MEIOTIC_CHROMOSOME_SEGREGATION | 57 | 0.620106977 | 2.064442308 | 9.96E-05 | 0.004179247 | 0.003542449 |
| GOBP_RESPONSE_TO_INORGANIC_SUBSTANCE | GOBP_RESPONSE_TO_INORGANIC_SUBSTANCE | 436 | 0.345640618 | 1.526487092 | 0.000101972 | 0.004252017 | 0.003604131 |
| GOBP_ENDODERMAL_CELL_DIFFERENTIATION | GOBP_ENDODERMAL_CELL_DIFFERENTIATION | 40 | 0.668557138 | 2.057339924 | 0.000105074 | 0.004334361 | 0.003673928 |
| GOBP_ENDODERM_FORMATION | GOBP_ENDODERM_FORMATION | 47 | 0.635039798 | 2.029409135 | 0.000105342 | 0.004334361 | 0.003673928 |
| GOMF_NEUROPEPTIDE_HORMONE_ACTIVITY | GOMF_NEUROPEPTIDE_HORMONE_ACTIVITY | 11 | 0.891959832 | 2.072378877 | 0.000106166 | 0.004339529 | 0.003678308 |
| GOMF_SINGLE_STRANDED_DNA_BINDING | GOMF_SINGLE_STRANDED_DNA_BINDING | 106 | 0.508161981 | 1.899732326 | 0.000106888 | 0.004340491 | 0.003679124 |
| GOCC_BLOOD_MICROPARTICLE | GOCC_BLOOD_MICROPARTICLE | 109 | 0.498594929 | 1.877344657 | 0.000114284 | 0.004610691 | 0.003908153 |
| GOCC_COLLAGEN_CONTAINING_EXTRACELLULAR_MATRIX | GOCC_COLLAGEN_CONTAINING_EXTRACELLULAR_MATRIX | 328 | 0.361515736 | 1.547955657 | 0.000116471 | 0.004666745 | 0.003955666 |
| GOBP_MOLTING_CYCLE | GOBP_MOLTING_CYCLE | 81 | 0.535718675 | 1.927647738 | 0.000117176 | 0.004666745 | 0.003955666 |
| GOBP_REGULATION_OF_DNA_REPAIR | GOBP_REGULATION_OF_DNA_REPAIR | 119 | 0.480533386 | 1.825214098 | 0.000119518 | 0.004720864 | 0.004001539 |
| GOBP_ANAPHASE_PROMOTING_COMPLEX_DEPENDENT_CATABOLIC_PROCESS | GOBP_ANAPHASE_PROMOTING_COMPLEX_DEPENDENT_CATABOLIC_PROCESS | 80 | 0.556214842 | 1.995935569 | 0.000120054 | 0.004720864 | 0.004001539 |
| GOBP_CELLULAR_RESPONSE_TO_LIPID | GOBP_CELLULAR_RESPONSE_TO_LIPID | 458 | 0.333029885 | 1.461904155 | 0.000125174 | 0.004891247 | 0.00414596 |
| GOBP_FEMALE_GAMETE_GENERATION | GOBP_FEMALE_GAMETE_GENERATION | 92 | 0.522825033 | 1.913279584 | 0.000126783 | 0.004923137 | 0.004172991 |
| GOBP_MITOCHONDRIAL_GENE_EXPRESSION | GOBP_MITOCHONDRIAL_GENE_EXPRESSION | 165 | 0.43917178 | 1.750536474 | 0.000139979 | 0.005401817 | 0.004578734 |
| GOBP_REGULATION_OF_HAIR_FOLLICLE_DEVELOPMENT | GOBP_REGULATION_OF_HAIR_FOLLICLE_DEVELOPMENT | 15 | 0.80712429 | 2.011443792 | 0.000143196 | 0.005463691 | 0.00463118 |
| GOBP_DNA_BIOSYNTHETIC_PROCESS | GOBP_DNA_BIOSYNTHETIC_PROCESS | 175 | 0.423455875 | 1.696312246 | 0.000143342 | 0.005463691 | 0.00463118 |
| GOBP_EXTERNAL_ENCAPSULATING_STRUCTURE_ORGANIZATION | GOBP_EXTERNAL_ENCAPSULATING_STRUCTURE_ORGANIZATION | 332 | 0.358924639 | 1.543936536 | 0.000145177 | 0.005499917 | 0.004661887 |
| GOBP_RRNA_METABOLIC_PROCESS | GOBP_RRNA_METABOLIC_PROCESS | 234 | 0.391293748 | 1.621560771 | 0.000151185 | 0.005692793 | 0.004825373 |
| GOCC_CONDENSED_NUCLEAR_CHROMOSOME | GOCC_CONDENSED_NUCLEAR_CHROMOSOME | 65 | 0.582085878 | 2.006782299 | 0.000154121 | 0.005768396 | 0.004889457 |
| GOBP_POSITIVE_REGULATION_OF_DNA_METABOLIC_PROCESS | GOBP_POSITIVE_REGULATION_OF_DNA_METABOLIC_PROCESS | 179 | 0.428774118 | 1.715755788 | 0.00015869 | 0.005854404 | 0.00496236 |
| GOBP_MOTILE_CILIUM_ASSEMBLY | GOBP_MOTILE_CILIUM_ASSEMBLY | 20 | -0.775565871 | -2.000363588 | 0.000159245 | 0.005854404 | 0.00496236 |
| GOBP_AROMATIC_AMINO_ACID_FAMILY_CATABOLIC_PROCESS | GOBP_AROMATIC_AMINO_ACID_FAMILY_CATABOLIC_PROCESS | 18 | 0.793897886 | 2.089464411 | 0.000159246 | 0.005854404 | 0.00496236 |
| GOBP_DNA_UNWINDING_INVOLVED_IN_DNA_REPLICATION | GOBP_DNA_UNWINDING_INVOLVED_IN_DNA_REPLICATION | 15 | 0.805476162 | 2.007336473 | 0.000162288 | 0.005907141 | 0.005007061 |
| GOBP_RNA_LOCALIZATION | GOBP_RNA_LOCALIZATION | 217 | 0.402810741 | 1.644597316 | 0.000162582 | 0.005907141 | 0.005007061 |
| GOCC_DNA_REPLICATION_PREINITIATION_COMPLEX | GOCC_DNA_REPLICATION_PREINITIATION_COMPLEX | 12 | 0.87658575 | 2.089261181 | 0.000167433 | 0.006048014 | 0.005126469 |
| GOMF_DNA_DEPENDENT_ATPASE_ACTIVITY | GOMF_DNA_DEPENDENT_ATPASE_ACTIVITY | 48 | 0.621264194 | 1.998317833 | 0.000168782 | 0.006061535 | 0.00513793 |
| GOBP_REGULATION_OF_ATTACHMENT_OF_SPINDLE_MICROTUBULES_TO_KINETOCHORE | GOBP_REGULATION_OF_ATTACHMENT_OF_SPINDLE_MICROTUBULES_TO_KINETOCHORE | 13 | 0.839574401 | 2.047692193 | 0.000169774 | 0.006062112 | 0.005138418 |
| GOBP_RESPONSE_TO_ZINC_ION | GOBP_RESPONSE_TO_ZINC_ION | 41 | 0.63457802 | 1.960313924 | 0.00017406 | 0.006179639 | 0.005238038 |
| GOCC_CILIARY_BASAL_BODY | GOCC_CILIARY_BASAL_BODY | 140 | -0.485627357 | -1.746535471 | 0.000179002 | 0.006318989 | 0.005356155 |
| GOBP_CELLULAR_RESPONSE_TO_HEAT | GOBP_CELLULAR_RESPONSE_TO_HEAT | 112 | 0.496632539 | 1.860823677 | 0.000181608 | 0.006374732 | 0.005403405 |
| GOCC_REPLICATION_FORK | GOCC_REPLICATION_FORK | 63 | 0.592070719 | 2.018803102 | 0.0001856 | 0.006459936 | 0.005475626 |
| GOMF_STRUCTURAL_CONSTITUENT_OF_CYTOSKELETON | GOMF_STRUCTURAL_CONSTITUENT_OF_CYTOSKELETON | 74 | 0.55638922 | 1.970331689 | 0.000186114 | 0.006459936 | 0.005475626 |
| GOBP_NEUROPEPTIDE_SIGNALING_PATHWAY | GOBP_NEUROPEPTIDE_SIGNALING_PATHWAY | 41 | 0.63316194 | 1.955939424 | 0.000191612 | 0.006613816 | 0.005606059 |
| GOBP_MITOCHONDRIAL_TRANSLATION | GOBP_MITOCHONDRIAL_TRANSLATION | 134 | 0.467940975 | 1.798478084 | 0.000206124 | 0.007075393 | 0.005997305 |
| GOBP_MITOTIC_METAPHASE_PLATE_CONGRESSION | GOBP_MITOTIC_METAPHASE_PLATE_CONGRESSION | 49 | 0.636918229 | 2.057673034 | 0.000209495 | 0.00713248 | 0.006045693 |
| GOBP_REGULATION_OF_NERVOUS_SYSTEM_DEVELOPMENT | GOBP_REGULATION_OF_NERVOUS_SYSTEM_DEVELOPMENT | 328 | 0.354914398 | 1.519689725 | 0.000210083 | 0.00713248 | 0.006045693 |
| GOBP_DNA_STRAND_ELONGATION | GOBP_DNA_STRAND_ELONGATION | 24 | 0.755147031 | 2.064594191 | 0.000214206 | 0.007204161 | 0.006106452 |
| GOMF_RIBONUCLEOPROTEIN_COMPLEX_BINDING | GOMF_RIBONUCLEOPROTEIN_COMPLEX_BINDING | 129 | 0.465497257 | 1.797598142 | 0.000214513 | 0.007204161 | 0.006106452 |
| GOBP_PYRUVATE_METABOLIC_PROCESS | GOBP_PYRUVATE_METABOLIC_PROCESS | 127 | 0.468072255 | 1.810194395 | 0.000215825 | 0.007209254 | 0.006110769 |
| GOCC_NUCLEAR_CHROMOSOME | GOCC_NUCLEAR_CHROMOSOME | 216 | 0.398756723 | 1.622098808 | 0.000221527 | 0.007360146 | 0.006238669 |
| GOBP_PROTEIN_OXIDATION | GOBP_PROTEIN_OXIDATION | 13 | 0.834496486 | 2.035307338 | 0.000225049 | 0.007437378 | 0.006304133 |
| GOBP_POSITIVE_REGULATION_OF_RESPONSE_TO_EXTERNAL_STIMULUS | GOBP_POSITIVE_REGULATION_OF_RESPONSE_TO_EXTERNAL_STIMULUS | 427 | 0.332518353 | 1.469709418 | 0.000230456 | 0.007575779 | 0.006421446 |
| GOCC_CMG_COMPLEX | GOCC_CMG_COMPLEX | 10 | 0.881794507 | 1.966569053 | 0.000234585 | 0.007670943 | 0.00650211 |
| GOBP_KINETOCHORE_ASSEMBLY | GOBP_KINETOCHORE_ASSEMBLY | 17 | 0.807111619 | 2.062859559 | 0.000238232 | 0.007749393 | 0.006568607 |
| GOBP_SIGNAL_TRANSDUCTION_IN_RESPONSE_TO_DNA_DAMAGE | GOBP_SIGNAL_TRANSDUCTION_IN_RESPONSE_TO_DNA_DAMAGE | 127 | 0.466101154 | 1.80257148 | 0.000246903 | 0.007989636 | 0.006772243 |
| GOBP_CELL_CHEMOTAXIS | GOBP_CELL_CHEMOTAXIS | 261 | 0.376717066 | 1.578637248 | 0.000251638 | 0.008069472 | 0.006839914 |
| GOBP_ATTACHMENT_OF_MITOTIC_SPINDLE_MICROTUBULES_TO_KINETOCHORE | GOBP_ATTACHMENT_OF_MITOTIC_SPINDLE_MICROTUBULES_TO_KINETOCHORE | 15 | 0.794553913 | 1.980117011 | 0.000252295 | 0.008069472 | 0.006839914 |
| GOBP_NUCLEOSIDE_PHOSPHATE_BIOSYNTHETIC_PROCESS | GOBP_NUCLEOSIDE_PHOSPHATE_BIOSYNTHETIC_PROCESS | 230 | 0.388555182 | 1.606670931 | 0.000254269 | 0.008069472 | 0.006839914 |
| GOMF_DNA_REPLICATION_ORIGIN_BINDING | GOMF_DNA_REPLICATION_ORIGIN_BINDING | 22 | 0.748785705 | 2.025903239 | 0.000254566 | 0.008069472 | 0.006839914 |
| GOBP_DNA_DEPENDENT_DNA_REPLICATION_MAINTENANCE_OF_FIDELITY | GOBP_DNA_DEPENDENT_DNA_REPLICATION_MAINTENANCE_OF_FIDELITY | 44 | 0.634810959 | 1.986409133 | 0.00026208 | 0.008217742 | 0.006965593 |
| GOBP_REGULATION_OF_HAIR_CYCLE | GOBP_REGULATION_OF_HAIR_CYCLE | 22 | 0.747584879 | 2.022654301 | 0.000263099 | 0.008217742 | 0.006965593 |
| GOBP_CELLULAR_RESPONSE_TO_OXYGEN_LEVELS | GOBP_CELLULAR_RESPONSE_TO_OXYGEN_LEVELS | 201 | 0.40428643 | 1.631119785 | 0.000263211 | 0.008217742 | 0.006965593 |
| GOBP_INTRINSIC_APOPTOTIC_SIGNALING_PATHWAY | GOBP_INTRINSIC_APOPTOTIC_SIGNALING_PATHWAY | 265 | 0.378775953 | 1.589504738 | 0.00026506 | 0.008234079 | 0.00697944 |
| GOCC_NUCLEAR_PORE | GOCC_NUCLEAR_PORE | 71 | 0.55225183 | 1.924334348 | 0.00027294 | 0.008436692 | 0.00715118 |
| GOCC_SPINDLE_MIDZONE | GOCC_SPINDLE_MIDZONE | 35 | 0.69349286 | 2.089662524 | 0.000295394 | 0.009085548 | 0.007701169 |
| GOBP_HORMONE_TRANSPORT | GOBP_HORMONE_TRANSPORT | 244 | 0.378132042 | 1.575546916 | 0.000302608 | 0.009209894 | 0.007806568 |
| GOBP_ESTABLISHMENT_OF_MITOTIC_SPINDLE_LOCALIZATION | GOBP_ESTABLISHMENT_OF_MITOTIC_SPINDLE_LOCALIZATION | 30 | 0.689135585 | 1.997706355 | 0.000305078 | 0.009209894 | 0.007806568 |
| GOBP_HEMIDESMOSOME_ASSEMBLY | GOBP_HEMIDESMOSOME_ASSEMBLY | 12 | 0.86608452 | 2.064232469 | 0.000305215 | 0.009209894 | 0.007806568 |
| GOBP_DNA_CONFORMATION_CHANGE | GOBP_DNA_CONFORMATION_CHANGE | 295 | 0.356964912 | 1.522163121 | 0.000305366 | 0.009209894 | 0.007806568 |
| GOBP_HISTONE_PHOSPHORYLATION | GOBP_HISTONE_PHOSPHORYLATION | 37 | 0.657021337 | 1.99466665 | 0.000321327 | 0.009644466 | 0.008174924 |
| GOBP_RESPONSE_TO_OXIDATIVE_STRESS | GOBP_RESPONSE_TO_OXIDATIVE_STRESS | 389 | 0.330612609 | 1.442626359 | 0.000323003 | 0.00964816 | 0.008178055 |
| GOCC_MHC_CLASS_II_PROTEIN_COMPLEX | GOCC_MHC_CLASS_II_PROTEIN_COMPLEX | 14 | -0.816678301 | -1.943885883 | 0.000352909 | 0.010491009 | 0.008892478 |
| GOBP_GENERATION_OF_PRECURSOR_METABOLITES_AND_ENERGY | GOBP_GENERATION_OF_PRECURSOR_METABOLITES_AND_ENERGY | 466 | 0.324453275 | 1.432781371 | 0.000355151 | 0.010491465 | 0.008892865 |
| GOCC_CYCLIN_DEPENDENT_PROTEIN_KINASE_HOLOENZYME_COMPLEX | GOCC_CYCLIN_DEPENDENT_PROTEIN_KINASE_HOLOENZYME_COMPLEX | 42 | 0.63628378 | 1.969135052 | 0.000356301 | 0.010491465 | 0.008892865 |
| GOBP_GASTRULATION | GOBP_GASTRULATION | 150 | 0.441713181 | 1.738584683 | 0.000358444 | 0.010504762 | 0.008904135 |
| GOCC_SPINDLE_MICROTUBULE | GOCC_SPINDLE_MICROTUBULE | 68 | 0.558786155 | 1.924879216 | 0.000360196 | 0.010506553 | 0.008905653 |
| GOBP_REGULATION_OF_BINDING | GOBP_REGULATION_OF_BINDING | 321 | 0.341976709 | 1.465004578 | 0.000377188 | 0.010950795 | 0.009282206 |
| GOBP_MITOTIC_DNA_INTEGRITY_CHECKPOINT | GOBP_MITOTIC_DNA_INTEGRITY_CHECKPOINT | 102 | 0.481142134 | 1.792399934 | 0.000380503 | 0.010995665 | 0.009320239 |
| GOBP_LEUKOCYTE_MIGRATION | GOBP_LEUKOCYTE_MIGRATION | 443 | 0.325290633 | 1.436348094 | 0.000398711 | 0.011468466 | 0.009720999 |
| GOBP_RESPONSE_TO_TEMPERATURE_STIMULUS | GOBP_RESPONSE_TO_TEMPERATURE_STIMULUS | 195 | 0.405024625 | 1.635571893 | 0.000407255 | 0.011571343 | 0.0098082 |
| GOBP_DOUBLE_STRAND_BREAK_REPAIR_VIA_BREAK_INDUCED_REPLICATION | GOBP_DOUBLE_STRAND_BREAK_REPAIR_VIA_BREAK_INDUCED_REPLICATION | 11 | 0.869309709 | 2.019753596 | 0.000407558 | 0.011571343 | 0.0098082 |
| GOBP_CONNECTIVE_TISSUE_DEVELOPMENT | GOBP_CONNECTIVE_TISSUE_DEVELOPMENT | 201 | 0.397757307 | 1.604777615 | 0.000407874 | 0.011571343 | 0.0098082 |
| GOMF_LIPASE_ACTIVITY | GOMF_LIPASE_ACTIVITY | 103 | -0.494668472 | -1.697140339 | 0.000412513 | 0.011649749 | 0.009874659 |
| GOBP_RESPONSE_TO_TOXIC_SUBSTANCE | GOBP_RESPONSE_TO_TOXIC_SUBSTANCE | 194 | 0.397442543 | 1.608810416 | 0.000416467 | 0.011708186 | 0.009924192 |
| GOCC_DNA_PACKAGING_COMPLEX | GOCC_DNA_PACKAGING_COMPLEX | 82 | -0.529729014 | -1.743354318 | 0.000441322 | 0.012340832 | 0.010460441 |
| GOBP_PRODUCTION_OF_MOLECULAR_MEDIATOR_OF_IMMUNE_RESPONSE | GOBP_PRODUCTION_OF_MOLECULAR_MEDIATOR_OF_IMMUNE_RESPONSE | 247 | 0.372197087 | 1.547937372 | 0.000442943 | 0.012340832 | 0.010460441 |
| GOBP_TRANSLATIONAL_TERMINATION | GOBP_TRANSLATIONAL_TERMINATION | 105 | 0.478272484 | 1.790501581 | 0.000453217 | 0.012570698 | 0.010655281 |
| GOBP_GLUTAMINE_METABOLIC_PROCESS | GOBP_GLUTAMINE_METABOLIC_PROCESS | 19 | 0.745685719 | 1.969853834 | 0.000458794 | 0.01266882 | 0.010738453 |
| GOBP_REGULATION_OF_INNATE_IMMUNE_RESPONSE | GOBP_REGULATION_OF_INNATE_IMMUNE_RESPONSE | 269 | 0.357339868 | 1.50659199 | 0.000466757 | 0.012828614 | 0.010873899 |
| GOBP_SPINDLE_ASSEMBLY | GOBP_SPINDLE_ASSEMBLY | 109 | 0.471898548 | 1.776825566 | 0.00046871 | 0.012828614 | 0.010873899 |
| GOBP_TRANSITION_METAL_ION_HOMEOSTASIS | GOBP_TRANSITION_METAL_ION_HOMEOSTASIS | 116 | 0.464486373 | 1.758650987 | 0.000489854 | 0.013348534 | 0.011314597 |
| GOBP_POSITIVE_REGULATION_OF_RESPONSE_TO_BIOTIC_STIMULUS | GOBP_POSITIVE_REGULATION_OF_RESPONSE_TO_BIOTIC_STIMULUS | 215 | 0.39087256 | 1.583813266 | 0.000497707 | 0.013503304 | 0.011445785 |
| GOBP_RESPONSE_TO_ESTROGEN | GOBP_RESPONSE_TO_ESTROGEN | 60 | 0.571403757 | 1.919690696 | 0.000505401 | 0.013652421 | 0.011572181 |
| GOBP_DNA_DAMAGE_RESPONSE_SIGNAL_TRANSDUCTION_BY_P53_CLASS_MEDIATOR | GOBP_DNA_DAMAGE_RESPONSE_SIGNAL_TRANSDUCTION_BY_P53_CLASS_MEDIATOR | 105 | 0.475534358 | 1.7802509 | 0.00051549 | 0.013864667 | 0.011752087 |
| GOBP_EPITHELIAL_CELL_PROLIFERATION | GOBP_EPITHELIAL_CELL_PROLIFERATION | 344 | 0.348038447 | 1.503515609 | 0.000521121 | 0.013918666 | 0.011797858 |
| GOMF_CARBOXYLIC_ESTER_HYDROLASE_ACTIVITY | GOMF_CARBOXYLIC_ESTER_HYDROLASE_ACTIVITY | 113 | -0.489119609 | -1.706171832 | 0.000521978 | 0.013918666 | 0.011797858 |
| GOMF_CALCIUM_DEPENDENT_PROTEIN_BINDING | GOMF_CALCIUM_DEPENDENT_PROTEIN_BINDING | 64 | 0.563726518 | 1.925431825 | 0.000528308 | 0.014027248 | 0.011889895 |
| GOBP_MICROTUBULE_ORGANIZING_CENTER_ORGANIZATION | GOBP_MICROTUBULE_ORGANIZING_CENTER_ORGANIZATION | 132 | 0.460148828 | 1.772076966 | 0.000535863 | 0.01416731 | 0.012008615 |
| GOBP_MAINTENANCE_OF_LOCATION | GOBP_MAINTENANCE_OF_LOCATION | 270 | 0.370305193 | 1.561613664 | 0.000539341 | 0.014198832 | 0.012035335 |
| GOBP_POSITIVE_REGULATION_OF_MITOTIC_CELL_CYCLE | GOBP_POSITIVE_REGULATION_OF_MITOTIC_CELL_CYCLE | 102 | 0.473489067 | 1.763889945 | 0.000589271 | 0.015416151 | 0.013067169 |
| GOBP_EMBRYONIC_ORGAN_MORPHOGENESIS | GOBP_EMBRYONIC_ORGAN_MORPHOGENESIS | 210 | 0.392104033 | 1.590553174 | 0.000590543 | 0.015416151 | 0.013067169 |
| GOBP_NUCLEOSIDE_MONOPHOSPHATE_BIOSYNTHETIC_PROCESS | GOBP_NUCLEOSIDE_MONOPHOSPHATE_BIOSYNTHETIC_PROCESS | 39 | 0.641070016 | 1.954408977 | 0.00059553 | 0.015457134 | 0.013101907 |
| GOBP_INTERMEDIATE_FILAMENT_BASED_PROCESS | GOBP_INTERMEDIATE_FILAMENT_BASED_PROCESS | 38 | 0.625200686 | 1.890755875 | 0.000597089 | 0.015457134 | 0.013101907 |
| GOBP_FOREBRAIN_DEVELOPMENT | GOBP_FOREBRAIN_DEVELOPMENT | 264 | 0.362369928 | 1.523924629 | 0.000603784 | 0.01554379 | 0.01317536 |
| GOBP_NCRNA_TRANSCRIPTION | GOBP_NCRNA_TRANSCRIPTION | 105 | 0.473389711 | 1.772222019 | 0.00060544 | 0.01554379 | 0.01317536 |
| GOBP_PHOSPHATIDYLSERINE_ACYL_CHAIN_REMODELING | GOBP_PHOSPHATIDYLSERINE_ACYL_CHAIN_REMODELING | 18 | -0.762809243 | -1.893902983 | 0.000608995 | 0.01557072 | 0.013198186 |
| GOBP_TRANSCYTOSIS | GOBP_TRANSCYTOSIS | 20 | -0.742820521 | -1.915905765 | 0.000612097 | 0.015585902 | 0.013211055 |
| GOBP_SPERM_MOTILITY | GOBP_SPERM_MOTILITY | 53 | -0.586913421 | -1.83602569 | 0.000620043 | 0.015723778 | 0.013327922 |
| GOBP_RESPONSE_TO_DEXAMETHASONE | GOBP_RESPONSE_TO_DEXAMETHASONE | 33 | 0.662955017 | 1.983232514 | 0.000649139 | 0.016329456 | 0.013841312 |
| GOBP_RESPONSE_TO_FOOD | GOBP_RESPONSE_TO_FOOD | 22 | 0.717968506 | 1.942524692 | 0.000649183 | 0.016329456 | 0.013841312 |
| GOBP_L_PHENYLALANINE_METABOLIC_PROCESS | GOBP_L_PHENYLALANINE_METABOLIC_PROCESS | 10 | 0.860099447 | 1.918184954 | 0.000658528 | 0.016477297 | 0.013966626 |
| GOBP_FEMALE_MEIOTIC_NUCLEAR_DIVISION | GOBP_FEMALE_MEIOTIC_NUCLEAR_DIVISION | 21 | 0.744562725 | 1.993646722 | 0.000660365 | 0.016477297 | 0.013966626 |
| GOBP_RESPONSE_TO_KETONE | GOBP_RESPONSE_TO_KETONE | 158 | 0.423389296 | 1.679381219 | 0.000669878 | 0.01658998 | 0.01406214 |
| GOBP_PALLIUM_DEVELOPMENT | GOBP_PALLIUM_DEVELOPMENT | 122 | 0.465136318 | 1.776273818 | 0.000670221 | 0.01658998 | 0.01406214 |
| GOBP_REGULATION_OF_APOPTOTIC_SIGNALING_PATHWAY | GOBP_REGULATION_OF_APOPTOTIC_SIGNALING_PATHWAY | 315 | 0.349283144 | 1.497074937 | 0.000690125 | 0.01701487 | 0.014422289 |
| GOBP_REGENERATION | GOBP_REGENERATION | 164 | 0.418038767 | 1.66698367 | 0.0006992 | 0.017170463 | 0.014554174 |
| GOBP_REGULATION_OF_HORMONE_LEVELS | GOBP_REGULATION_OF_HORMONE_LEVELS | 387 | 0.330214177 | 1.438731494 | 0.000705964 | 0.017268317 | 0.014637118 |
| GOBP_MYELOID_LEUKOCYTE_MIGRATION | GOBP_MYELOID_LEUKOCYTE_MIGRATION | 194 | 0.391193092 | 1.58351322 | 0.000714483 | 0.017404653 | 0.014752679 |
| GOBP_REGULATION_OF_FEEDING_BEHAVIOR | GOBP_REGULATION_OF_FEEDING_BEHAVIOR | 14 | -0.805438256 | -1.91713194 | 0.00071714 | 0.017404653 | 0.014752679 |
| GOBP_RIBONUCLEOSIDE_MONOPHOSPHATE_BIOSYNTHETIC_PROCESS | GOBP_RIBONUCLEOSIDE_MONOPHOSPHATE_BIOSYNTHETIC_PROCESS | 31 | 0.651875911 | 1.908475684 | 0.000741101 | 0.017916183 | 0.015186267 |
| GOBP_SYNAPSE_ORGANIZATION | GOBP_SYNAPSE_ORGANIZATION | 297 | 0.349567434 | 1.492719028 | 0.000749036 | 0.018008093 | 0.015264173 |
| GOBP_ZINC_ION_HOMEOSTASIS | GOBP_ZINC_ION_HOMEOSTASIS | 30 | 0.665364914 | 1.92879855 | 0.0007507 | 0.018008093 | 0.015264173 |
| GOBP_MORPHOGENESIS_OF_AN_EPITHELIUM | GOBP_MORPHOGENESIS_OF_AN_EPITHELIUM | 465 | 0.313164582 | 1.379513556 | 0.000762717 | 0.018225993 | 0.015448871 |
| GOBP_DETOXIFICATION_OF_INORGANIC_COMPOUND | GOBP_DETOXIFICATION_OF_INORGANIC_COMPOUND | 13 | 0.807361279 | 1.969125531 | 0.000781515 | 0.018603641 | 0.015768976 |
| GOBP_SIGNAL_TRANSDUCTION_INVOLVED_IN_CELL_CYCLE_CHECKPOINT | GOBP_SIGNAL_TRANSDUCTION_INVOLVED_IN_CELL_CYCLE_CHECKPOINT | 73 | 0.532262325 | 1.883153005 | 0.000789252 | 0.01868226 | 0.015835615 |
| GOBP_PHOSPHATIDYLSERINE_METABOLIC_PROCESS | GOBP_PHOSPHATIDYLSERINE_METABOLIC_PROCESS | 31 | -0.655818678 | -1.847753978 | 0.000795579 | 0.01868226 | 0.015835615 |
| GOBP_GLUCOSE_CATABOLIC_PROCESS | GOBP_GLUCOSE_CATABOLIC_PROCESS | 31 | 0.649838863 | 1.902511887 | 0.000798549 | 0.01868226 | 0.015835615 |
| GOBP_CARTILAGE_DEVELOPMENT | GOBP_CARTILAGE_DEVELOPMENT | 154 | 0.422560106 | 1.670288437 | 0.000799366 | 0.01868226 | 0.015835615 |
| GOMF_G_PROTEIN_COUPLED_RECEPTOR_BINDING | GOMF_G_PROTEIN_COUPLED_RECEPTOR_BINDING | 207 | 0.394729317 | 1.597661871 | 0.000799852 | 0.01868226 | 0.015835615 |
| GOMF_RAGE_RECEPTOR_BINDING | GOMF_RAGE_RECEPTOR_BINDING | 10 | 0.855001487 | 1.906815536 | 0.000861971 | 0.020024827 | 0.016973614 |
| GOMF_ATPASE_ACTIVITY | GOMF_ATPASE_ACTIVITY | 411 | 0.323227578 | 1.426322467 | 0.000865325 | 0.020024827 | 0.016973614 |
| GOBP_SKELETAL_SYSTEM_MORPHOGENESIS | GOBP_SKELETAL_SYSTEM_MORPHOGENESIS | 168 | 0.422176108 | 1.676851226 | 0.000867001 | 0.020024827 | 0.016973614 |
| GOBP_REGULATION_OF_CILIUM_BEAT_FREQUENCY | GOBP_REGULATION_OF_CILIUM_BEAT_FREQUENCY | 13 | -0.781187987 | -1.812968786 | 0.000872009 | 0.020065897 | 0.017008426 |
| GOBP_DNA_REPLICATION_DEPENDENT_NUCLEOSOME_ORGANIZATION | GOBP_DNA_REPLICATION_DEPENDENT_NUCLEOSOME_ORGANIZATION | 26 | -0.691898038 | -1.867888837 | 0.000875689 | 0.020076218 | 0.017017175 |
| GOBP_POSITIVE_REGULATION_OF_RESPONSE_TO_DNA_DAMAGE_STIMULUS | GOBP_POSITIVE_REGULATION_OF_RESPONSE_TO_DNA_DAMAGE_STIMULUS | 98 | 0.476530392 | 1.760403283 | 0.00088396 | 0.020191332 | 0.017114748 |
| GOBP_DNA_STRAND_ELONGATION_INVOLVED_IN_DNA_REPLICATION | GOBP_DNA_STRAND_ELONGATION_INVOLVED_IN_DNA_REPLICATION | 18 | 0.753293099 | 1.982596439 | 0.000898474 | 0.020447695 | 0.017332049 |
| GOBP_REGULATION_OF_CELL_DEVELOPMENT | GOBP_REGULATION_OF_CELL_DEVELOPMENT | 394 | 0.322137147 | 1.410000075 | 0.000904676 | 0.020513698 | 0.017387995 |
| GOMF_CALCIUM_DEPENDENT_PHOSPHOLIPASE_A2_ACTIVITY | GOMF_CALCIUM_DEPENDENT_PHOSPHOLIPASE_A2_ACTIVITY | 11 | -0.841172213 | -1.869854982 | 0.000931133 | 0.02098967 | 0.017791442 |
| GOCC_ORGANELLAR_RIBOSOME | GOCC_ORGANELLAR_RIBOSOME | 87 | 0.496580181 | 1.797562438 | 0.000932424 | 0.02098967 | 0.017791442 |
| GOBP_HOMOLOGOUS_CHROMOSOME_SEGREGATION | GOBP_HOMOLOGOUS_CHROMOSOME_SEGREGATION | 33 | 0.651079384 | 1.94770651 | 0.000941538 | 0.021118319 | 0.017900489 |
| GOBP_EXTRACELLULAR_TRANSPORT | GOBP_EXTRACELLULAR_TRANSPORT | 32 | -0.660780894 | -1.877988164 | 0.000945551 | 0.021132049 | 0.017912127 |
| GOBP_POSITIVE_REGULATION_OF_SMALL_MOLECULE_METABOLIC_PROCESS | GOBP_POSITIVE_REGULATION_OF_SMALL_MOLECULE_METABOLIC_PROCESS | 107 | 0.463859233 | 1.738854982 | 0.000953255 | 0.021227853 | 0.017993333 |
| GOBP_INTERSTRAND_CROSS_LINK_REPAIR | GOBP_INTERSTRAND_CROSS_LINK_REPAIR | 51 | 0.578827693 | 1.886111852 | 0.000969621 | 0.021515201 | 0.018236897 |
| GOBP_ANTIMICROBIAL_HUMORAL_IMMUNE_RESPONSE_MEDIATED_BY_ANTIMICROBIAL_PEPTIDE | GOBP_ANTIMICROBIAL_HUMORAL_IMMUNE_RESPONSE_MEDIATED_BY_ANTIMICROBIAL_PEPTIDE | 53 | 0.567542868 | 1.86634513 | 0.00098445 | 0.021766499 | 0.018449904 |
| GOBP_MITOTIC_DNA_REPLICATION | GOBP_MITOTIC_DNA_REPLICATION | 15 | 0.765766674 | 1.908375999 | 0.001030189 | 0.022679737 | 0.019223991 |
| GOCC_DISTAL_AXON | GOCC_DISTAL_AXON | 221 | 0.374765815 | 1.531747659 | 0.001035975 | 0.022679737 | 0.019223991 |
| GOBP_CYTOKINESIS | GOBP_CYTOKINESIS | 144 | 0.415751979 | 1.610314002 | 0.001036705 | 0.022679737 | 0.019223991 |
| GOBP_REGULATION_OF_CELL_AGING | GOBP_REGULATION_OF_CELL_AGING | 45 | 0.594895357 | 1.868351047 | 0.001063526 | 0.023144848 | 0.019618232 |
| GOBP_EMBRYONIC_CAMERA_TYPE_EYE_FORMATION | GOBP_EMBRYONIC_CAMERA_TYPE_EYE_FORMATION | 10 | 0.850041963 | 1.895754856 | 0.001065415 | 0.023144848 | 0.019618232 |
| GOBP_POSITIVE_REGULATION_OF_CELL_CYCLE_PHASE_TRANSITION | GOBP_POSITIVE_REGULATION_OF_CELL_CYCLE_PHASE_TRANSITION | 88 | 0.497798304 | 1.800933406 | 0.001076078 | 0.023226768 | 0.01968767 |
| GOBP_POSITIVE_REGULATION_OF_PROTEIN_LOCALIZATION_TO_NUCLEUS | GOBP_POSITIVE_REGULATION_OF_PROTEIN_LOCALIZATION_TO_NUCLEUS | 79 | 0.514139309 | 1.840416284 | 0.001076663 | 0.023226768 | 0.01968767 |
| GOBP_POSITIVE_REGULATION_OF_TELOMERASE_RNA_LOCALIZATION_TO_CAJAL_BODY | GOBP_POSITIVE_REGULATION_OF_TELOMERASE_RNA_LOCALIZATION_TO_CAJAL_BODY | 15 | 0.764334484 | 1.904806822 | 0.001096297 | 0.023568491 | 0.019977324 |
| GOBP_REGULATION_OF_WATER_LOSS_VIA_SKIN | GOBP_REGULATION_OF_WATER_LOSS_VIA_SKIN | 20 | 0.733125016 | 1.949927734 | 0.001110287 | 0.023786952 | 0.020162498 |
| GOBP_REGULATION_OF_MACROPHAGE_DERIVED_FOAM_CELL_DIFFERENTIATION | GOBP_REGULATION_OF_MACROPHAGE_DERIVED_FOAM_CELL_DIFFERENTIATION | 28 | -0.671484865 | -1.83940096 | 0.001138364 | 0.024304667 | 0.020601328 |
| GOBP_SPINDLE_LOCALIZATION | GOBP_SPINDLE_LOCALIZATION | 44 | 0.597763639 | 1.87048307 | 0.001167593 | 0.024843347 | 0.021057929 |
| GOBP_AMINO_SUGAR_CATABOLIC_PROCESS | GOBP_AMINO_SUGAR_CATABOLIC_PROCESS | 13 | -0.776133835 | -1.801239189 | 0.001176005 | 0.024906835 | 0.021111743 |
| GOBP_ENDODERM_DEVELOPMENT | GOBP_ENDODERM_DEVELOPMENT | 66 | 0.53444225 | 1.833109001 | 0.001178595 | 0.024906835 | 0.021111743 |
| GOBP_MITOCHONDRIAL_TRANSLATIONAL_TERMINATION | GOBP_MITOCHONDRIAL_TRANSLATIONAL_TERMINATION | 89 | 0.490035924 | 1.769110099 | 0.001212286 | 0.025410776 | 0.021538898 |
| GOBP_SKIN_MORPHOGENESIS | GOBP_SKIN_MORPHOGENESIS | 10 | 0.843743008 | 1.881706991 | 0.001215955 | 0.025410776 | 0.021538898 |
| GOBP_NUCLEOSOME_POSITIONING | GOBP_NUCLEOSOME_POSITIONING | 12 | -0.798645568 | -1.817985508 | 0.001220788 | 0.025410776 | 0.021538898 |
| GOBP_POSITIVE_REGULATION_OF_BINDING | GOBP_POSITIVE_REGULATION_OF_BINDING | 152 | 0.426071369 | 1.680363611 | 0.001221827 | 0.025410776 | 0.021538898 |
| GOBP_ANTIBACTERIAL_HUMORAL_RESPONSE | GOBP_ANTIBACTERIAL_HUMORAL_RESPONSE | 46 | -0.587035935 | -1.788586104 | 0.001222891 | 0.025410776 | 0.021538898 |
| GOBP_MAINTENANCE_OF_CELL_NUMBER | GOBP_MAINTENANCE_OF_CELL_NUMBER | 123 | 0.432023767 | 1.645854555 | 0.001230306 | 0.025451691 | 0.021573579 |
| GOBP_SENSORY_ORGAN_MORPHOGENESIS | GOBP_SENSORY_ORGAN_MORPHOGENESIS | 178 | 0.392547338 | 1.566997231 | 0.001233053 | 0.025451691 | 0.021573579 |
| GOBP_MUSCLE_TISSUE_DEVELOPMENT | GOBP_MUSCLE_TISSUE_DEVELOPMENT | 282 | 0.350855549 | 1.490966842 | 0.001244836 | 0.025609813 | 0.021707607 |
| GOBP_TELENCEPHALON_DEVELOPMENT | GOBP_TELENCEPHALON_DEVELOPMENT | 178 | 0.392147692 | 1.565401894 | 0.001265289 | 0.025944696 | 0.021991463 |
| GOBP_CELLULAR_TRANSITION_METAL_ION_HOMEOSTASIS | GOBP_CELLULAR_TRANSITION_METAL_ION_HOMEOSTASIS | 97 | 0.474576528 | 1.754212633 | 0.001270033 | 0.025956295 | 0.022001295 |
| GOBP_NEGATIVE_REGULATION_OF_GENE_EXPRESSION_EPIGENETIC | GOBP_NEGATIVE_REGULATION_OF_GENE_EXPRESSION_EPIGENETIC | 100 | -0.490663962 | -1.669966447 | 0.001310706 | 0.026699733 | 0.022631454 |
| GOBP_TELOMERE_MAINTENANCE_VIA_SEMI_CONSERVATIVE_REPLICATION | GOBP_TELOMERE_MAINTENANCE_VIA_SEMI_CONSERVATIVE_REPLICATION | 26 | 0.688455458 | 1.923818645 | 0.001317912 | 0.026758778 | 0.022681503 |
| GOBP_DESMOSOME_ORGANIZATION | GOBP_DESMOSOME_ORGANIZATION | 10 | 0.841587963 | 1.876900831 | 0.001323559 | 0.026785904 | 0.022704495 |
| GOBP_INTERLEUKIN_1_MEDIATED_SIGNALING_PATHWAY | GOBP_INTERLEUKIN_1_MEDIATED_SIGNALING_PATHWAY | 97 | 0.472850137 | 1.747831248 | 0.001349825 | 0.027228769 | 0.023079881 |
| GOBP_REGULATION_OF_SECRETION | GOBP_REGULATION_OF_SECRETION | 466 | 0.311398814 | 1.375133043 | 0.001361099 | 0.027367334 | 0.023197332 |
| GOBP_PROTEIN_MODIFICATION_BY_SMALL_PROTEIN_REMOVAL | GOBP_PROTEIN_MODIFICATION_BY_SMALL_PROTEIN_REMOVAL | 266 | 0.346147593 | 1.453251393 | 0.001378853 | 0.027627365 | 0.023417742 |
| GOBP_POSITIVE_REGULATION_OF_CELL_GROWTH | GOBP_POSITIVE_REGULATION_OF_CELL_GROWTH | 134 | 0.43171905 | 1.659263222 | 0.001382925 | 0.027627365 | 0.023417742 |
| GOBP_LEUKOCYTE_CHEMOTAXIS | GOBP_LEUKOCYTE_CHEMOTAXIS | 197 | 0.374717728 | 1.511495629 | 0.001395559 | 0.027790403 | 0.023555937 |
| GOBP_RNA_EXPORT_FROM_NUCLEUS | GOBP_RNA_EXPORT_FROM_NUCLEUS | 131 | 0.430466641 | 1.661764726 | 0.001448337 | 0.028749248 | 0.024368682 |
| GOMF_HISTONE_KINASE_ACTIVITY | GOMF_HISTONE_KINASE_ACTIVITY | 16 | 0.749376183 | 1.896829611 | 0.001458554 | 0.028859858 | 0.024462438 |
| GOBP_TELOMERASE_RNA_LOCALIZATION | GOBP_TELOMERASE_RNA_LOCALIZATION | 19 | 0.718246333 | 1.897368095 | 0.001480487 | 0.029200836 | 0.024751461 |
| GOBP_CEREBRAL_CORTEX_DEVELOPMENT | GOBP_CEREBRAL_CORTEX_DEVELOPMENT | 90 | 0.478733454 | 1.740656456 | 0.001487087 | 0.029238199 | 0.02478313 |
| GOBP_POSITIVE_REGULATION_OF_STEROID_METABOLIC_PROCESS | GOBP_POSITIVE_REGULATION_OF_STEROID_METABOLIC_PROCESS | 22 | 0.692446059 | 1.873471548 | 0.001512614 | 0.029646275 | 0.025129027 |
| GOBP_RESPONSE_TO_PEPTIDE_HORMONE | GOBP_RESPONSE_TO_PEPTIDE_HORMONE | 349 | 0.327894565 | 1.423847374 | 0.001536401 | 0.029944459 | 0.025381777 |
| GOBP_ESTABLISHMENT_OF_ORGANELLE_LOCALIZATION | GOBP_ESTABLISHMENT_OF_ORGANELLE_LOCALIZATION | 383 | 0.326960976 | 1.426303765 | 0.001537467 | 0.029944459 | 0.025381777 |
| GOBP_STRESS_RESPONSE_TO_METAL_ION | GOBP_STRESS_RESPONSE_TO_METAL_ION | 13 | 0.787071341 | 1.919639091 | 0.001549377 | 0.030009845 | 0.025437199 |
| GOBP_REGULATION_OF_CELL_CYCLE_ARREST | GOBP_REGULATION_OF_CELL_CYCLE_ARREST | 99 | 0.458778331 | 1.701073612 | 0.001550484 | 0.030009845 | 0.025437199 |
| GOBP_RESPONSE_TO_NUTRIENT | GOBP_RESPONSE_TO_NUTRIENT | 137 | 0.424396989 | 1.639383867 | 0.001571262 | 0.030251581 | 0.025642102 |
| GOBP_NERVE_DEVELOPMENT | GOBP_NERVE_DEVELOPMENT | 46 | 0.579853278 | 1.844514904 | 0.001572712 | 0.030251581 | 0.025642102 |
| GOBP_NEGATIVE_REGULATION_OF_PHOSPHORYLATION | GOBP_NEGATIVE_REGULATION_OF_PHOSPHORYLATION | 349 | 0.327155813 | 1.420639424 | 0.001593657 | 0.03055985 | 0.025903399 |
| GOBP_POSITIVE_REGULATION_OF_CELLULAR_PROTEIN_LOCALIZATION | GOBP_POSITIVE_REGULATION_OF_CELLULAR_PROTEIN_LOCALIZATION | 275 | 0.346340544 | 1.464632443 | 0.001621667 | 0.03100128 | 0.026277568 |
| GOBP_TROPHOBLAST_GIANT_CELL_DIFFERENTIATION | GOBP_TROPHOBLAST_GIANT_CELL_DIFFERENTIATION | 11 | 0.839514808 | 1.950528142 | 0.001667893 | 0.031751567 | 0.026913533 |
| GOBP_REGULATION_OF_SMALL_MOLECULE_METABOLIC_PROCESS | GOBP_REGULATION_OF_SMALL_MOLECULE_METABOLIC_PROCESS | 369 | 0.316584864 | 1.376894413 | 0.0016746 | 0.031751567 | 0.026913533 |
| GOMF_FRIZZLED_BINDING | GOMF_FRIZZLED_BINDING | 28 | 0.656647959 | 1.871229423 | 0.001676246 | 0.031751567 | 0.026913533 |
| GOBP_TRNA_TRANSPORT | GOBP_TRNA_TRANSPORT | 36 | 0.62267067 | 1.876426659 | 0.001693837 | 0.031923891 | 0.027059599 |
| GOCC_SMALL_SUBUNIT_PROCESSOME | GOCC_SMALL_SUBUNIT_PROCESSOME | 37 | 0.613034797 | 1.861126871 | 0.00169562 | 0.031923891 | 0.027059599 |
| GOBP_REGULATION_OF_CYTOKINESIS | GOBP_REGULATION_OF_CYTOKINESIS | 70 | 0.53058867 | 1.843151588 | 0.001733455 | 0.032444833 | 0.027501165 |
| GOBP_CELLULAR_RESPONSE_TO_CHEMICAL_STRESS | GOBP_CELLULAR_RESPONSE_TO_CHEMICAL_STRESS | 306 | 0.336004974 | 1.436484495 | 0.001733733 | 0.032444833 | 0.027501165 |
| GOMF_MICROTUBULE_BINDING | GOMF_MICROTUBULE_BINDING | 238 | 0.355344283 | 1.480462054 | 0.001747067 | 0.03259618 | 0.027629451 |
| GOBP_NCRNA_EXPORT_FROM_NUCLEUS | GOBP_NCRNA_EXPORT_FROM_NUCLEUS | 38 | 0.589971279 | 1.784213752 | 0.001764007 | 0.032813703 | 0.027813829 |
| GOMF_STEROID_HYDROXYLASE_ACTIVITY | GOMF_STEROID_HYDROXYLASE_ACTIVITY | 17 | -0.749608468 | -1.862880735 | 0.001789757 | 0.033193306 | 0.028135592 |
| GOBP_REGULATION_OF_DNA_BINDING | GOBP_REGULATION_OF_DNA_BINDING | 103 | 0.463611562 | 1.730940987 | 0.001803326 | 0.033292502 | 0.028219673 |
| GOCC_MITOTIC_SPINDLE_MIDZONE | GOCC_MITOTIC_SPINDLE_MIDZONE | 13 | 0.781988534 | 1.907242305 | 0.001805822 | 0.033292502 | 0.028219673 |
| GOBP_CELL_GROWTH | GOBP_CELL_GROWTH | 397 | 0.314446797 | 1.375656263 | 0.001813986 | 0.033342865 | 0.028262362 |
| GOBP_DETOXIFICATION | GOBP_DETOXIFICATION | 108 | 0.454965088 | 1.709280136 | 0.001819287 | 0.033342865 | 0.028262362 |
| GOBP_RESPONSE_TO_GLUCAGON | GOBP_RESPONSE_TO_GLUCAGON | 27 | 0.6789467 | 1.913079493 | 0.001826797 | 0.033344162 | 0.028263462 |
| GOBP_COLLAGEN_CATABOLIC_PROCESS | GOBP_COLLAGEN_CATABOLIC_PROCESS | 38 | 0.588623556 | 1.780137915 | 0.001836006 | 0.033344162 | 0.028263462 |
| GOBP_PEPTIDE_SECRETION | GOBP_PEPTIDE_SECRETION | 314 | 0.330081217 | 1.410997633 | 0.001837786 | 0.033344162 | 0.028263462 |
| GOBP_G0_TO_G1_TRANSITION | GOBP_G0_TO_G1_TRANSITION | 43 | 0.593005737 | 1.850300332 | 0.001840825 | 0.033344162 | 0.028263462 |
| GOBP_FOAM_CELL_DIFFERENTIATION | GOBP_FOAM_CELL_DIFFERENTIATION | 33 | -0.627949565 | -1.797849106 | 0.001851701 | 0.033376304 | 0.028290706 |
| GOBP_POSITIVE_REGULATION_OF_G_PROTEIN_COUPLED_RECEPTOR_SIGNALING_PATHWAY | GOBP_POSITIVE_REGULATION_OF_G_PROTEIN_COUPLED_RECEPTOR_SIGNALING_PATHWAY | 19 | 0.713599639 | 1.885093074 | 0.001853344 | 0.033376304 | 0.028290706 |
| GOBP_CELLULAR_RESPONSE_TO_ORGANIC_CYCLIC_COMPOUND | GOBP_CELLULAR_RESPONSE_TO_ORGANIC_CYCLIC_COMPOUND | 431 | 0.309057753 | 1.370067552 | 0.001896963 | 0.034063098 | 0.028872852 |
| GOBP_CELL_DIFFERENTIATION_INVOLVED_IN_EMBRYONIC_PLACENTA_DEVELOPMENT | GOBP_CELL_DIFFERENTIATION_INVOLVED_IN_EMBRYONIC_PLACENTA_DEVELOPMENT | 21 | 0.714544683 | 1.913270188 | 0.001907216 | 0.034111147 | 0.02891358 |
| GOMF_CATALYTIC_ACTIVITY_ACTING_ON_RNA | GOMF_CATALYTIC_ACTIVITY_ACTING_ON_RNA | 358 | 0.324620543 | 1.410232614 | 0.00191062 | 0.034111147 | 0.02891358 |
| GOCC_SITE_OF_DNA_DAMAGE | GOCC_SITE_OF_DNA_DAMAGE | 87 | 0.478643239 | 1.732632795 | 0.00195495 | 0.034802583 | 0.029499661 |
| GOMF_DYNEIN_HEAVY_CHAIN_BINDING | GOMF_DYNEIN_HEAVY_CHAIN_BINDING | 13 | -0.762264623 | -1.769051741 | 0.00200989 | 0.035678426 | 0.03024205 |
| GOMF_PHOSPHOLIPASE_A2_ACTIVITY | GOMF_PHOSPHOLIPASE_A2_ACTIVITY | 26 | -0.673091831 | -1.817118487 | 0.002066111 | 0.036571927 | 0.030999407 |
| GOMF_3_5_DNA_HELICASE_ACTIVITY | GOMF_3_5_DNA_HELICASE_ACTIVITY | 17 | 0.743740926 | 1.900893313 | 0.002135475 | 0.03769235 | 0.031949109 |
| GOBP_RETROGRADE_VESICLE_MEDIATED_TRANSPORT_GOLGI_TO_ENDOPLASMIC_RETICULUM | GOBP_RETROGRADE_VESICLE_MEDIATED_TRANSPORT_GOLGI_TO_ENDOPLASMIC_RETICULUM | 81 | 0.470488963 | 1.692935168 | 0.002165375 | 0.03811182 | 0.032304665 |
| GOBP_CEREBROSPINAL_FLUID_CIRCULATION | GOBP_CEREBROSPINAL_FLUID_CIRCULATION | 10 | -0.807212983 | -1.765199355 | 0.002191363 | 0.038460275 | 0.032600025 |
| GOMF_DYNEIN_INTERMEDIATE_CHAIN_BINDING | GOMF_DYNEIN_INTERMEDIATE_CHAIN_BINDING | 24 | -0.668579663 | -1.790671515 | 0.002211805 | 0.038616787 | 0.032732689 |
| GOBP_REGULATION_OF_NEUROGENESIS | GOBP_REGULATION_OF_NEUROGENESIS | 280 | 0.349260748 | 1.48539147 | 0.002213581 | 0.038616787 | 0.032732689 |
| GOCC_CATALYTIC_STEP_2_SPLICEOSOME | GOCC_CATALYTIC_STEP_2_SPLICEOSOME | 86 | 0.465481522 | 1.680482636 | 0.002218927 | 0.038616787 | 0.032732689 |
| GOBP_NEUTROPHIL_MIGRATION | GOBP_NEUTROPHIL_MIGRATION | 110 | 0.45675964 | 1.718248092 | 0.002234347 | 0.038689927 | 0.032794684 |
| GOMF_TRANSLATION_REGULATOR_ACTIVITY | GOMF_TRANSLATION_REGULATOR_ACTIVITY | 123 | 0.422856386 | 1.610930144 | 0.002241395 | 0.038689927 | 0.032794684 |
| GOCC_DENSE_CORE_GRANULE | GOCC_DENSE_CORE_GRANULE | 21 | 0.708631248 | 1.897436328 | 0.002241811 | 0.038689927 | 0.032794684 |
| GOCC_CLATHRIN_COATED_ENDOCYTIC_VESICLE | GOCC_CLATHRIN_COATED_ENDOCYTIC_VESICLE | 49 | -0.566563728 | -1.74341099 | 0.002266295 | 0.039004134 | 0.033061015 |
| GOBP_MUSCLE_STRUCTURE_DEVELOPMENT | GOBP_MUSCLE_STRUCTURE_DEVELOPMENT | 462 | 0.303827078 | 1.338697596 | 0.002369435 | 0.040666581 | 0.034470152 |
| GOCC_PRONUCLEUS | GOCC_PRONUCLEUS | 10 | 0.826873263 | 1.844084258 | 0.002443919 | 0.041829396 | 0.035455788 |
| GOBP_RIBOSOMAL_LARGE_SUBUNIT_BIOGENESIS | GOBP_RIBOSOMAL_LARGE_SUBUNIT_BIOGENESIS | 70 | 0.519631358 | 1.805088227 | 0.002452506 | 0.041861052 | 0.03548262 |
| GOBP_COLLAGEN_METABOLIC_PROCESS | GOBP_COLLAGEN_METABOLIC_PROCESS | 89 | 0.470246801 | 1.697668115 | 0.002464076 | 0.041943298 | 0.035552334 |
| GOBP_PURINE_CONTAINING_COMPOUND_METABOLIC_PROCESS | GOBP_PURINE_CONTAINING_COMPOUND_METABOLIC_PROCESS | 384 | 0.31156964 | 1.359528731 | 0.002487302 | 0.042222967 | 0.03578939 |
| GOBP_ESTABLISHMENT_OF_MITOTIC_SPINDLE_ORIENTATION | GOBP_ESTABLISHMENT_OF_MITOTIC_SPINDLE_ORIENTATION | 25 | 0.67602497 | 1.861382036 | 0.002548876 | 0.043150319 | 0.03657544 |
| GOBP_REGULATION_OF_MEGAKARYOCYTE_DIFFERENTIATION | GOBP_REGULATION_OF_MEGAKARYOCYTE_DIFFERENTIATION | 73 | -0.51330523 | -1.654351739 | 0.002568113 | 0.043264643 | 0.036672344 |
| GOBP_AROMATIC_AMINO_ACID_FAMILY_METABOLIC_PROCESS | GOBP_AROMATIC_AMINO_ACID_FAMILY_METABOLIC_PROCESS | 24 | 0.68614306 | 1.875935304 | 0.002569556 | 0.043264643 | 0.036672344 |
| GOBP_OSTEOBLAST_DIFFERENTIATION | GOBP_OSTEOBLAST_DIFFERENTIATION | 191 | 0.374165998 | 1.51199743 | 0.00261273 | 0.043818357 | 0.037141688 |
| GOBP_RESPONSE_TO_RADIATION | GOBP_RESPONSE_TO_RADIATION | 365 | 0.316188225 | 1.373376903 | 0.002616548 | 0.043818357 | 0.037141688 |
| GOBP_OXIDATIVE_PHOSPHORYLATION | GOBP_OXIDATIVE_PHOSPHORYLATION | 140 | 0.408281172 | 1.583726254 | 0.002638384 | 0.044065261 | 0.03735097 |
| GOBP_CELLULAR_RESPONSE_TO_ABIOTIC_STIMULUS | GOBP_CELLULAR_RESPONSE_TO_ABIOTIC_STIMULUS | 274 | 0.340015563 | 1.437708566 | 0.002666747 | 0.044300799 | 0.037550619 |
| GOBP_RESPONSE_TO_TUMOR_NECROSIS_FACTOR | GOBP_RESPONSE_TO_TUMOR_NECROSIS_FACTOR | 281 | 0.333541999 | 1.416917128 | 0.002666747 | 0.044300799 | 0.037550619 |
| GOBP_GLYCOLYTIC_PROCESS_THROUGH_FRUCTOSE_6_PHOSPHATE | GOBP_GLYCOLYTIC_PROCESS_THROUGH_FRUCTOSE_6_PHOSPHATE | 24 | 0.681477252 | 1.863178848 | 0.002708448 | 0.044747935 | 0.037929625 |
| GOMF_DNA_SECONDARY_STRUCTURE_BINDING | GOMF_DNA_SECONDARY_STRUCTURE_BINDING | 34 | 0.61692867 | 1.839429681 | 0.002711223 | 0.044747935 | 0.037929625 |
| GOBP_NEGATIVE_REGULATION_OF_CELL_AGING | GOBP_NEGATIVE_REGULATION_OF_CELL_AGING | 22 | 0.67812587 | 1.834727061 | 0.00271527 | 0.044747935 | 0.037929625 |
| GOBP_COLUMNAR_CUBOIDAL_EPITHELIAL_CELL_DIFFERENTIATION | GOBP_COLUMNAR_CUBOIDAL_EPITHELIAL_CELL_DIFFERENTIATION | 64 | 0.522608123 | 1.784990202 | 0.002766549 | 0.045416249 | 0.038496107 |
| GOBP_PROTEIN_NITROSYLATION | GOBP_PROTEIN_NITROSYLATION | 13 | 0.76410628 | 1.863628121 | 0.002770442 | 0.045416249 | 0.038496107 |
| GOBP_RESPONSE_TO_ACID_CHEMICAL | GOBP_RESPONSE_TO_ACID_CHEMICAL | 100 | 0.451517386 | 1.675173692 | 0.002782928 | 0.045500878 | 0.038567841 |
| GOBP_NEUTROPHIL_CHEMOTAXIS | GOBP_NEUTROPHIL_CHEMOTAXIS | 93 | 0.450821816 | 1.65626555 | 0.002791113 | 0.045514928 | 0.03857975 |
| GOBP_NEGATIVE_REGULATION_OF_APOPTOTIC_SIGNALING_PATHWAY | GOBP_NEGATIVE_REGULATION_OF_APOPTOTIC_SIGNALING_PATHWAY | 199 | 0.385823961 | 1.556662659 | 0.002901067 | 0.04718411 | 0.039994596 |
| GOBP_POSITIVE_REGULATION_OF_CELL_CYCLE_ARREST | GOBP_POSITIVE_REGULATION_OF_CELL_CYCLE_ARREST | 76 | 0.489246816 | 1.739779233 | 0.002918832 | 0.047236504 | 0.040039006 |
| GOBP_POSITIVE_REGULATION_OF_ANION_TRANSMEMBRANE_TRANSPORT | GOBP_POSITIVE_REGULATION_OF_ANION_TRANSMEMBRANE_TRANSPORT | 50 | -0.557563094 | -1.72267799 | 0.002919494 | 0.047236504 | 0.040039006 |
| GOBP_RIBONUCLEOPROTEIN_COMPLEX_BIOGENESIS | GOBP_RIBONUCLEOPROTEIN_COMPLEX_BIOGENESIS | 445 | 0.305681829 | 1.343975254 | 0.002950363 | 0.047611966 | 0.040357259 |
| GOMF_PHOSPHOLIPASE_A2_ACTIVITY_CONSUMING_1_2_DIPALMITOYLPHOSPHATIDYLCHOLINE | GOMF_PHOSPHOLIPASE_A2_ACTIVITY_CONSUMING_1_2_DIPALMITOYLPHOSPHATIDYLCHOLINE | 22 | -0.669667847 | -1.768074791 | 0.00296957 | 0.047744202 | 0.040469346 |
| GOBP_NUCLEAR_TRANSPORT | GOBP_NUCLEAR_TRANSPORT | 314 | 0.324540723 | 1.387313691 | 0.002973927 | 0.047744202 | 0.040469346 |
| GOBP_RNA_3_END_PROCESSING | GOBP_RNA_3_END_PROCESSING | 135 | 0.410804726 | 1.581847496 | 0.002984209 | 0.047785805 | 0.04050461 |
| GOBP_REGULATION_OF_SIGNALING_RECEPTOR_ACTIVITY | GOBP_REGULATION_OF_SIGNALING_RECEPTOR_ACTIVITY | 125 | 0.423877004 | 1.62342987 | 0.003013722 | 0.048036632 | 0.040717218 |
| GOBP_RESPONSE_TO_METAL_ION | GOBP_RESPONSE_TO_METAL_ION | 274 | 0.338569369 | 1.431593535 | 0.003015337 | 0.048036632 | 0.040717218 |
| GOMF_TUBULIN_BINDING | GOMF_TUBULIN_BINDING | 324 | 0.321179726 | 1.373781707 | 0.00303642 | 0.048248796 | 0.040897054 |
| GOBP_TRNA_METABOLIC_PROCESS | GOBP_TRNA_METABOLIC_PROCESS | 176 | 0.387897595 | 1.558345439 | 0.003082077 | 0.048800963 | 0.041365086 |
| GOBP_POSITIVE_REGULATION_OF_DNA_DEPENDENT_DNA_REPLICATION | GOBP_POSITIVE_REGULATION_OF_DNA_DEPENDENT_DNA_REPLICATION | 10 | 0.821745771 | 1.832648979 | 0.003092732 | 0.048800963 | 0.041365086 |
| GOBP_REGULATION_OF_NUCLEOTIDE_BIOSYNTHETIC_PROCESS | GOBP_REGULATION_OF_NUCLEOTIDE_BIOSYNTHETIC_PROCESS | 33 | 0.617785796 | 1.848108613 | 0.003094734 | 0.048800963 | 0.041365086 |
| GOMF_CYTOKINE_RECEPTOR_BINDING | GOMF_CYTOKINE_RECEPTOR_BINDING | 198 | 0.360636933 | 1.450564284 | 0.003148444 | 0.049522233 | 0.041976455 |
| GOBP_RESPONSE_TO_PEPTIDE | GOBP_RESPONSE_TO_PEPTIDE | 421 | 0.30795632 | 1.359134228 | 0.003166739 | 0.049681149 | 0.042111157 |
| GOBP_DE_NOVO_PROTEIN_FOLDING | GOBP_DE_NOVO_PROTEIN_FOLDING | 41 | 0.558252868 | 1.724533212 | 0.00317454 | 0.049681149 | 0.042111157 |
| GOBP_SIGNAL_RELEASE | GOBP_SIGNAL_RELEASE | 356 | 0.313345177 | 1.362313445 | 0.003193137 | 0.049846628 | 0.042251422 |
| GOBP_RESPONSE_TO_DRUG | GOBP_RESPONSE_TO_DRUG | 283 | 0.339238376 | 1.439487683 | 0.003203495 | 0.049882993 | 0.042282246 |
| GOBP_POSITIVE_REGULATION_OF_ESTABLISHMENT_OF_PROTEIN_LOCALIZATION_TO_TELOMERE | GOBP_POSITIVE_REGULATION_OF_ESTABLISHMENT_OF_PROTEIN_LOCALIZATION_TO_TELOMERE | 10 | 0.821340772 | 1.831745754 | 0.003222495 | 0.050053407 | 0.042426694 |
| GOBP_NUCLEOBASE_BIOSYNTHETIC_PROCESS | GOBP_NUCLEOBASE_BIOSYNTHETIC_PROCESS | 18 | 0.713902044 | 1.878922893 | 0.003242912 | 0.050244921 | 0.042589026 |
| GOBP_MUSCLE_CELL_MIGRATION | GOBP_MUSCLE_CELL_MIGRATION | 75 | 0.487776051 | 1.733635321 | 0.003256219 | 0.050325587 | 0.042657401 |
| GOBP_POSITIVE_REGULATION_OF_DNA_REPLICATION | GOBP_POSITIVE_REGULATION_OF_DNA_REPLICATION | 36 | 0.60474915 | 1.822419912 | 0.003309177 | 0.051017156 | 0.043243595 |
| GOCC_NUCLEAR_REPLICATION_FORK | GOCC_NUCLEAR_REPLICATION_FORK | 33 | 0.615728458 | 1.84195408 | 0.003330968 | 0.051176966 | 0.043379055 |
| GOBP_NEGATIVE_REGULATION_OF_PHOSPHORUS_METABOLIC_PROCESS | GOBP_NEGATIVE_REGULATION_OF_PHOSPHORUS_METABOLIC_PROCESS | 444 | 0.30278348 | 1.333641263 | 0.003336017 | 0.051176966 | 0.043379055 |
| GOCC_CONDENSED_NUCLEAR_CHROMOSOME_KINETOCHORE | GOCC_CONDENSED_NUCLEAR_CHROMOSOME_KINETOCHORE | 17 | 0.727876969 | 1.860347354 | 0.003348251 | 0.051238139 | 0.043430906 |
| GOBP_POSITIVE_REGULATION_OF_RELEASE_OF_SEQUESTERED_CALCIUM_ION_INTO_CYTOSOL | GOBP_POSITIVE_REGULATION_OF_RELEASE_OF_SEQUESTERED_CALCIUM_ION_INTO_CYTOSOL | 33 | 0.614830885 | 1.839268986 | 0.003378215 | 0.05156966 | 0.043711913 |
| GOBP_PHOSPHATIDYLETHANOLAMINE_ACYL_CHAIN_REMODELING | GOBP_PHOSPHATIDYLETHANOLAMINE_ACYL_CHAIN_REMODELING | 22 | -0.666290114 | -1.759156809 | 0.00345868 | 0.05266858 | 0.044643389 |
| GOMF_UNFOLDED_PROTEIN_BINDING | GOMF_UNFOLDED_PROTEIN_BINDING | 105 | 0.43715449 | 1.636568759 | 0.00351118 | 0.053337321 | 0.045210233 |
| GOBP_PHOSPHATIDYLGLYCEROL_ACYL_CHAIN_REMODELING | GOBP_PHOSPHATIDYLGLYCEROL_ACYL_CHAIN_REMODELING | 14 | -0.754834127 | -1.796682246 | 0.00353173 | 0.053518637 | 0.045363921 |
| GOMF_HEAT_SHOCK_PROTEIN_BINDING | GOMF_HEAT_SHOCK_PROTEIN_BINDING | 112 | 0.436671098 | 1.636155214 | 0.003550365 | 0.053670118 | 0.045492321 |
| GOCC_PROTEIN_KINASE_COMPLEX | GOCC_PROTEIN_KINASE_COMPLEX | 94 | 0.453650075 | 1.672974993 | 0.00358296 | 0.054031387 | 0.045798543 |
| GOCC_LAMELLAR_BODY | GOCC_LAMELLAR_BODY | 17 | -0.730583118 | -1.815600107 | 0.003659747 | 0.05496084 | 0.046586374 |
| GOBP_CELLULAR_RESPONSE_TO_DEXAMETHASONE_STIMULUS | GOBP_CELLULAR_RESPONSE_TO_DEXAMETHASONE_STIMULUS | 26 | 0.654248818 | 1.828231672 | 0.003662287 | 0.05496084 | 0.046586374 |
| GOBP_GLUCOSE_6_PHOSPHATE_METABOLIC_PROCESS | GOBP_GLUCOSE_6_PHOSPHATE_METABOLIC_PROCESS | 21 | 0.695303694 | 1.86175037 | 0.003698768 | 0.055374573 | 0.046937066 |
| GOBP_REPLICATION_FORK_PROCESSING | GOBP_REPLICATION_FORK_PROCESSING | 35 | 0.622643431 | 1.876175977 | 0.003728063 | 0.055622305 | 0.04714705 |
| GOBP_POSITIVE_REGULATION_OF_UBIQUITIN_PROTEIN_TRANSFERASE_ACTIVITY | GOBP_POSITIVE_REGULATION_OF_UBIQUITIN_PROTEIN_TRANSFERASE_ACTIVITY | 32 | 0.611080189 | 1.811267417 | 0.003745811 | 0.055622305 | 0.04714705 |
| GOBP_SKELETAL_MUSCLE_ORGAN_DEVELOPMENT | GOBP_SKELETAL_MUSCLE_ORGAN_DEVELOPMENT | 119 | 0.4181157 | 1.588132461 | 0.00374828 | 0.055622305 | 0.04714705 |
| GOBP_MITOTIC_CHROMOSOME_CONDENSATION | GOBP_MITOTIC_CHROMOSOME_CONDENSATION | 14 | 0.77832294 | 1.910504631 | 0.003751126 | 0.055622305 | 0.04714705 |
| GOCC_FICOLIN_1_RICH_GRANULE_LUMEN | GOCC_FICOLIN_1_RICH_GRANULE_LUMEN | 121 | 0.416182954 | 1.587493346 | 0.003761139 | 0.055637985 | 0.047160341 |
| GOBP_SPINDLE_MIDZONE_ASSEMBLY | GOBP_SPINDLE_MIDZONE_ASSEMBLY | 11 | 0.819041546 | 1.90296058 | 0.003828056 | 0.056493384 | 0.047885402 |
| GOBP_PEPTIDE_HORMONE_SECRETION | GOBP_PEPTIDE_HORMONE_SECRETION | 191 | 0.368290634 | 1.488255201 | 0.003867716 | 0.056943418 | 0.048266863 |
| GOBP_ANTERIOR_POSTERIOR_PATTERN_SPECIFICATION | GOBP_ANTERIOR_POSTERIOR_PATTERN_SPECIFICATION | 146 | 0.39921894 | 1.55583206 | 0.003900312 | 0.057199591 | 0.048484003 |
| GOBP_MORPHOGENESIS_OF_AN_EPITHELIAL_SHEET | GOBP_MORPHOGENESIS_OF_AN_EPITHELIAL_SHEET | 54 | 0.534183025 | 1.777335139 | 0.003903529 | 0.057199591 | 0.048484003 |
| GOBP_REGULATION_OF_NUCLEOTIDE_METABOLIC_PROCESS | GOBP_REGULATION_OF_NUCLEOTIDE_METABOLIC_PROCESS | 100 | 0.441268585 | 1.637149637 | 0.003917684 | 0.057200897 | 0.04848511 |
| GOBP_ESTABLISHMENT_OF_SPINDLE_ORIENTATION | GOBP_ESTABLISHMENT_OF_SPINDLE_ORIENTATION | 29 | 0.647497225 | 1.86500494 | 0.003922032 | 0.057200897 | 0.04848511 |
| GOCC_ORGANELLE_INNER_MEMBRANE | GOCC_ORGANELLE_INNER_MEMBRANE | 497 | 0.293245389 | 1.298529708 | 0.003991699 | 0.058080616 | 0.049230785 |
| GOBP_PURINE_NUCLEOSIDE_MONOPHOSPHATE_BIOSYNTHETIC_PROCESS | GOBP_PURINE_NUCLEOSIDE_MONOPHOSPHATE_BIOSYNTHETIC_PROCESS | 22 | 0.663545855 | 1.795279594 | 0.00401486 | 0.058281129 | 0.049400745 |
| GOMF_CHROMATIN_BINDING | GOMF_CHROMATIN_BINDING | 485 | 0.297636219 | 1.311550457 | 0.004054072 | 0.058587271 | 0.04966024 |
| GOBP_G2_DNA_DAMAGE_CHECKPOINT | GOBP_G2_DNA_DAMAGE_CHECKPOINT | 34 | 0.60105549 | 1.792102333 | 0.004054809 | 0.058587271 | 0.04966024 |
| GOBP_REGULATION_OF_VASCULAR_ENDOTHELIAL_GROWTH_FACTOR_RECEPTOR_SIGNALING_PATHWAY | GOBP_REGULATION_OF_VASCULAR_ENDOTHELIAL_GROWTH_FACTOR_RECEPTOR_SIGNALING_PATHWAY | 25 | 0.657871312 | 1.811397353 | 0.004092197 | 0.058990303 | 0.050001861 |
| GOBP_GLYCOSYL_COMPOUND_METABOLIC_PROCESS | GOBP_GLYCOSYL_COMPOUND_METABOLIC_PROCESS | 115 | 0.422108152 | 1.597716789 | 0.0041172 | 0.05921334 | 0.050190914 |
| GOCC_MULTIVESICULAR_BODY | GOCC_MULTIVESICULAR_BODY | 54 | -0.534707136 | -1.675340358 | 0.004138184 | 0.05937769 | 0.050330222 |
| GOBP_RIBOSOMAL_SMALL_SUBUNIT_BIOGENESIS | GOBP_RIBOSOMAL_SMALL_SUBUNIT_BIOGENESIS | 74 | 0.488569974 | 1.730164548 | 0.004160021 | 0.059553475 | 0.050479222 |
| GOCC_TRANSFERASE_COMPLEX_TRANSFERRING_PHOSPHORUS_CONTAINING_GROUPS | GOCC_TRANSFERASE_COMPLEX_TRANSFERRING_PHOSPHORUS_CONTAINING_GROUPS | 228 | 0.356164132 | 1.473247349 | 0.004190785 | 0.05985597 | 0.050735626 |
| GOBP_REGULATION_OF_CILIUM_MOVEMENT | GOBP_REGULATION_OF_CILIUM_MOVEMENT | 20 | -0.687031918 | -1.772014067 | 0.004273302 | 0.060573638 | 0.051343941 |
| GOMF_ATP_DEPENDENT_MICROTUBULE_MOTOR_ACTIVITY_MINUS_END_DIRECTED | GOMF_ATP_DEPENDENT_MICROTUBULE_MOTOR_ACTIVITY_MINUS_END_DIRECTED | 12 | -0.765119557 | -1.741669048 | 0.004278329 | 0.060573638 | 0.051343941 |
| GOCC_90S_PRERIBOSOME | GOCC_90S_PRERIBOSOME | 28 | 0.629295974 | 1.793285316 | 0.004285781 | 0.060573638 | 0.051343941 |
| GOBP_NAD_METABOLIC_PROCESS | GOBP_NAD_METABOLIC_PROCESS | 45 | 0.550938479 | 1.730298399 | 0.004296224 | 0.060573638 | 0.051343941 |
| GOBP_ALDITOL_METABOLIC_PROCESS | GOBP_ALDITOL_METABOLIC_PROCESS | 16 | 0.713711918 | 1.806555811 | 0.004298061 | 0.060573638 | 0.051343941 |
| GOBP_MESENCHYMAL_TO_EPITHELIAL_TRANSITION | GOBP_MESENCHYMAL_TO_EPITHELIAL_TRANSITION | 12 | 0.795864988 | 1.896870698 | 0.004299529 | 0.060573638 | 0.051343941 |
| GOBP_ENTRY_OF_BACTERIUM_INTO_HOST_CELL | GOBP_ENTRY_OF_BACTERIUM_INTO_HOST_CELL | 15 | 0.723799908 | 1.803790135 | 0.004384096 | 0.061549134 | 0.0521708 |
| GOBP_CELL_AGING | GOBP_CELL_AGING | 98 | 0.440603192 | 1.627680665 | 0.004388583 | 0.061549134 | 0.0521708 |
| GOBP_RESPONSE_TO_FUNGUS | GOBP_RESPONSE_TO_FUNGUS | 31 | 0.586542136 | 1.717200139 | 0.004479 | 0.062565543 | 0.053032337 |
| GOMF_UBIQUITIN_LIKE_PROTEIN_LIGASE_BINDING | GOMF_UBIQUITIN_LIKE_PROTEIN_LIGASE_BINDING | 294 | 0.326826825 | 1.390977781 | 0.004481195 | 0.062565543 | 0.053032337 |
| GOBP_CELL_JUNCTION_ASSEMBLY | GOBP_CELL_JUNCTION_ASSEMBLY | 330 | 0.313639757 | 1.347093175 | 0.004510302 | 0.062830729 | 0.053257116 |
| GOBP_NUCLEOBASE_METABOLIC_PROCESS | GOBP_NUCLEOBASE_METABOLIC_PROCESS | 31 | 0.586100444 | 1.715907011 | 0.004525414 | 0.062900224 | 0.053316022 |
| GOBP_IMMUNOGLOBULIN_PRODUCTION | GOBP_IMMUNOGLOBULIN_PRODUCTION | 170 | 0.379470261 | 1.512731035 | 0.004543981 | 0.063017302 | 0.053415261 |
| GOBP_REGULATION_OF_EXTRINSIC_APOPTOTIC_SIGNALING_PATHWAY_IN_ABSENCE_OF_LIGAND | GOBP_REGULATION_OF_EXTRINSIC_APOPTOTIC_SIGNALING_PATHWAY_IN_ABSENCE_OF_LIGAND | 35 | 0.61496942 | 1.853052319 | 0.004725367 | 0.065276087 | 0.055329871 |
| GOBP_RESPONSE_TO_ACTIVITY | GOBP_RESPONSE_TO_ACTIVITY | 54 | 0.528708022 | 1.759118696 | 0.004727867 | 0.065276087 | 0.055329871 |
| GOBP_EMBRYONIC_PLACENTA_DEVELOPMENT | GOBP_EMBRYONIC_PLACENTA_DEVELOPMENT | 73 | 0.483738756 | 1.711475806 | 0.004744715 | 0.065363445 | 0.055403918 |
| GOBP_SKIN_EPIDERMIS_DEVELOPMENT | GOBP_SKIN_EPIDERMIS_DEVELOPMENT | 71 | 0.481234274 | 1.676872024 | 0.004759348 | 0.065419976 | 0.055451835 |
| GOBP_MITOTIC_G2_M_TRANSITION_CHECKPOINT | GOBP_MITOTIC_G2_M_TRANSITION_CHECKPOINT | 35 | 0.612333548 | 1.845109794 | 0.004820348 | 0.066112195 | 0.05603858 |
| GOBP_PURINE_CONTAINING_COMPOUND_BIOSYNTHETIC_PROCESS | GOBP_PURINE_CONTAINING_COMPOUND_BIOSYNTHETIC_PROCESS | 177 | 0.36521577 | 1.460327584 | 0.004898624 | 0.067037782 | 0.056823134 |
| GOBP_REGULATION_OF_PROTEIN_SERINE_THREONINE_KINASE_ACTIVITY | GOBP_REGULATION_OF_PROTEIN_SERINE_THREONINE_KINASE_ACTIVITY | 436 | 0.305163619 | 1.347724489 | 0.004935739 | 0.067397239 | 0.057127819 |
| GOBP_REGULATION_OF_INTRINSIC_APOPTOTIC_SIGNALING_PATHWAY | GOBP_REGULATION_OF_INTRINSIC_APOPTOTIC_SIGNALING_PATHWAY | 149 | 0.391856112 | 1.53335119 | 0.004994414 | 0.067815933 | 0.057482716 |
| GOBP_REGULATION_OF_CELLULAR_RESPONSE_TO_HEAT | GOBP_REGULATION_OF_CELLULAR_RESPONSE_TO_HEAT | 76 | 0.478488233 | 1.701521327 | 0.005000044 | 0.067815933 | 0.057482716 |
| GOCC_NUCLEAR_PERIPHERY | GOCC_NUCLEAR_PERIPHERY | 119 | 0.411279387 | 1.562166037 | 0.005007223 | 0.067815933 | 0.057482716 |
| GOBP_MONOSACCHARIDE_CATABOLIC_PROCESS | GOBP_MONOSACCHARIDE_CATABOLIC_PROCESS | 53 | 0.526135943 | 1.730179885 | 0.005014196 | 0.067815933 | 0.057482716 |
| GOBP_CRANIAL_NERVE_DEVELOPMENT | GOBP_CRANIAL_NERVE_DEVELOPMENT | 28 | 0.623867367 | 1.777815582 | 0.005020977 | 0.067815933 | 0.057482716 |
| GOMF_DAMAGED_DNA_BINDING | GOMF_DAMAGED_DNA_BINDING | 65 | 0.497999957 | 1.71689013 | 0.005054852 | 0.068070391 | 0.057698402 |
| GOBP_RIBONUCLEOSIDE_TRIPHOSPHATE_METABOLIC_PROCESS | GOBP_RIBONUCLEOSIDE_TRIPHOSPHATE_METABOLIC_PROCESS | 81 | 0.444350003 | 1.598880753 | 0.005064758 | 0.068070391 | 0.057698402 |
| GOMF_CYCLIN_DEPENDENT_PROTEIN_SERINE_THREONINE_KINASE_REGULATOR_ACTIVITY | GOMF_CYCLIN_DEPENDENT_PROTEIN_SERINE_THREONINE_KINASE_REGULATOR_ACTIVITY | 47 | 0.540024382 | 1.725766507 | 0.005072685 | 0.068070391 | 0.057698402 |
| GOBP_GENE_SILENCING | GOBP_GENE_SILENCING | 277 | -0.368894412 | -1.434060011 | 0.005100491 | 0.068296014 | 0.057889647 |
| GOCC_PROTEASOME_ACCESSORY_COMPLEX | GOCC_PROTEASOME_ACCESSORY_COMPLEX | 24 | 0.656935077 | 1.79607982 | 0.00516449 | 0.069004253 | 0.058489971 |
| GOBP_SMALL_MOLECULE_CATABOLIC_PROCESS | GOBP_SMALL_MOLECULE_CATABOLIC_PROCESS | 361 | 0.30335924 | 1.31606771 | 0.005200416 | 0.069335154 | 0.058770451 |
| GOBP_ELECTRON_TRANSPORT_CHAIN | GOBP_ELECTRON_TRANSPORT_CHAIN | 167 | 0.375336716 | 1.491614556 | 0.005233402 | 0.069398294 | 0.058823971 |
| GOCC_MITOCHONDRIAL_LARGE_RIBOSOMAL_SUBUNIT | GOCC_MITOCHONDRIAL_LARGE_RIBOSOMAL_SUBUNIT | 57 | 0.525160079 | 1.748347827 | 0.005239916 | 0.069398294 | 0.058823971 |
| GOCC_INNER_MITOCHONDRIAL_MEMBRANE_PROTEIN_COMPLEX | GOCC_INNER_MITOCHONDRIAL_MEMBRANE_PROTEIN_COMPLEX | 137 | 0.401350904 | 1.55036019 | 0.00524404 | 0.069398294 | 0.058823971 |
| GOBP_MRNA_5_SPLICE_SITE_RECOGNITION | GOBP_MRNA_5_SPLICE_SITE_RECOGNITION | 14 | -0.741242449 | -1.764330864 | 0.005249831 | 0.069398294 | 0.058823971 |
| GOBP_REGULATION_OF_INSULIN_LIKE_GROWTH_FACTOR_RECEPTOR_SIGNALING_PATHWAY | GOBP_REGULATION_OF_INSULIN_LIKE_GROWTH_FACTOR_RECEPTOR_SIGNALING_PATHWAY | 15 | 0.719389155 | 1.792798046 | 0.005322717 | 0.070139503 | 0.059452241 |
| GOCC_MICROVILLUS_MEMBRANE | GOCC_MICROVILLUS_MEMBRANE | 23 | 0.64175871 | 1.7440299 | 0.005338014 | 0.070139503 | 0.059452241 |
| GOCC_CLEAVAGE_FURROW | GOCC_CLEAVAGE_FURROW | 48 | 0.526374946 | 1.693103277 | 0.005339769 | 0.070139503 | 0.059452241 |
| GOBP_MONOSACCHARIDE_METABOLIC_PROCESS | GOBP_MONOSACCHARIDE_METABOLIC_PROCESS | 235 | 0.342645906 | 1.419828556 | 0.005398667 | 0.070763544 | 0.059981195 |
| GOBP_PYRIMIDINE_NUCLEOSIDE_METABOLIC_PROCESS | GOBP_PYRIMIDINE_NUCLEOSIDE_METABOLIC_PROCESS | 31 | 0.579093408 | 1.695392743 | 0.005457356 | 0.071382223 | 0.060505605 |
| GOBP_RESPONSE_TO_ACIDIC_PH | GOBP_RESPONSE_TO_ACIDIC_PH | 17 | 0.710029992 | 1.814733083 | 0.005555303 | 0.072308187 | 0.061290479 |
| GOBP_CELLULAR_RESPONSE_TO_CORTICOSTEROID_STIMULUS | GOBP_CELLULAR_RESPONSE_TO_CORTICOSTEROID_STIMULUS | 47 | 0.537413321 | 1.717422286 | 0.005565164 | 0.072308187 | 0.061290479 |
| GOBP_POSITIVE_REGULATION_OF_UBIQUITIN_PROTEIN_LIGASE_ACTIVITY | GOBP_POSITIVE_REGULATION_OF_UBIQUITIN_PROTEIN_LIGASE_ACTIVITY | 13 | 0.740042587 | 1.804937629 | 0.005568246 | 0.072308187 | 0.061290479 |
| GOBP_RESPONSE_TO_TESTOSTERONE | GOBP_RESPONSE_TO_TESTOSTERONE | 38 | 0.553461874 | 1.673800608 | 0.005574702 | 0.072308187 | 0.061290479 |
| GOBP_DNA_TEMPLATED_TRANSCRIPTION_TERMINATION | GOBP_DNA_TEMPLATED_TRANSCRIPTION_TERMINATION | 73 | 0.479053839 | 1.694900493 | 0.005600728 | 0.072494421 | 0.061448336 |
| GOMF_ANNEALING_ACTIVITY | GOMF_ANNEALING_ACTIVITY | 13 | 0.739453433 | 1.803500701 | 0.005654574 | 0.072703135 | 0.061625249 |
| GOBP_RESPONSE_TO_X_RAY | GOBP_RESPONSE_TO_X_RAY | 32 | 0.599776701 | 1.7777634 | 0.005658181 | 0.072703135 | 0.061625249 |
| GOBP_IN_UTERO_EMBRYONIC_DEVELOPMENT | GOBP_IN_UTERO_EMBRYONIC_DEVELOPMENT | 299 | 0.318933548 | 1.359000596 | 0.005661187 | 0.072703135 | 0.061625249 |
| GOBP_POSITIVE_REGULATION_OF_DNA_BINDING | GOBP_POSITIVE_REGULATION_OF_DNA_BINDING | 46 | 0.543525973 | 1.728957642 | 0.00566366 | 0.072703135 | 0.061625249 |
| GOBP_DNA_DAMAGE_RESPONSE_DETECTION_OF_DNA_DAMAGE | GOBP_DNA_DAMAGE_RESPONSE_DETECTION_OF_DNA_DAMAGE | 38 | 0.55112351 | 1.66672884 | 0.0057686 | 0.073897544 | 0.062637663 |
| GOBP_EXTRACELLULAR_REGULATION_OF_SIGNAL_TRANSDUCTION | GOBP_EXTRACELLULAR_REGULATION_OF_SIGNAL_TRANSDUCTION | 10 | 0.802877153 | 1.790568381 | 0.005781331 | 0.073908246 | 0.062646735 |
| GOBP_BINDING_OF_SPERM_TO_ZONA_PELLUCIDA | GOBP_BINDING_OF_SPERM_TO_ZONA_PELLUCIDA | 21 | 0.673816383 | 1.804215785 | 0.005812111 | 0.074128399 | 0.062833343 |
| GOBP_MITOTIC_G1_S_TRANSITION_CHECKPOINT | GOBP_MITOTIC_G1_S_TRANSITION_CHECKPOINT | 61 | 0.494877245 | 1.674616829 | 0.005828695 | 0.074128399 | 0.062833343 |
| GOBP_RESPONSE_TO_VITAMIN | GOBP_RESPONSE_TO_VITAMIN | 75 | 0.47458932 | 1.686767536 | 0.005834345 | 0.074128399 | 0.062833343 |
| GOBP_REGULATION_OF_TRANSCRIPTION_REGULATORY_REGION_DNA_BINDING | GOBP_REGULATION_OF_TRANSCRIPTION_REGULATORY_REGION_DNA_BINDING | 43 | 0.554070309 | 1.728813759 | 0.005880944 | 0.074567965 | 0.063205931 |
| GOCC_DESMOSOME | GOCC_DESMOSOME | 21 | 0.673415666 | 1.803142824 | 0.00590222 | 0.074685318 | 0.063305403 |
| GOBP_MULTI_ORGANISM_LOCALIZATION | GOBP_MULTI_ORGANISM_LOCALIZATION | 65 | 0.493566584 | 1.701605762 | 0.005955689 | 0.075064559 | 0.063626858 |
| GOBP_PROTEIN_LOCALIZATION_TO_MITOCHONDRION | GOBP_PROTEIN_LOCALIZATION_TO_MITOCHONDRION | 138 | 0.394662438 | 1.525055078 | 0.005956354 | 0.075064559 | 0.063626858 |
| GOBP_PROTEIN_LOCALIZATION_TO_CILIUM | GOBP_PROTEIN_LOCALIZATION_TO_CILIUM | 53 | -0.53343269 | -1.668723339 | 0.005976251 | 0.075162849 | 0.063710172 |
| GOBP_CELLULAR_RESPONSE_TO_GLUCAGON_STIMULUS | GOBP_CELLULAR_RESPONSE_TO_GLUCAGON_STIMULUS | 17 | 0.703899776 | 1.799065145 | 0.006006944 | 0.075396244 | 0.063908004 |
| GOBP_REGULATION_OF_RESPONSE_TO_EXTRACELLULAR_STIMULUS | GOBP_REGULATION_OF_RESPONSE_TO_EXTRACELLULAR_STIMULUS | 18 | 0.690062981 | 1.816180726 | 0.006086213 | 0.076131597 | 0.06453131 |
| GOBP_REGULATION_OF_UBIQUITIN_PROTEIN_TRANSFERASE_ACTIVITY | GOBP_REGULATION_OF_UBIQUITIN_PROTEIN_TRANSFERASE_ACTIVITY | 50 | 0.536528009 | 1.744303213 | 0.006090038 | 0.076131597 | 0.06453131 |
| GOBP_MITOTIC_CYTOKINESIS | GOBP_MITOTIC_CYTOKINESIS | 67 | 0.494475403 | 1.698227858 | 0.006137594 | 0.076572025 | 0.06490463 |
| GOBP_CHONDROCYTE_DIFFERENTIATION | GOBP_CHONDROCYTE_DIFFERENTIATION | 89 | 0.445900654 | 1.609774531 | 0.006172574 | 0.076854112 | 0.065143734 |
| GOBP_ALCOHOL_BIOSYNTHETIC_PROCESS | GOBP_ALCOHOL_BIOSYNTHETIC_PROCESS | 133 | 0.401657403 | 1.544266984 | 0.00619187 | 0.076940172 | 0.065216681 |
| GOBP_CELLULAR_RESPONSE_TO_FATTY_ACID | GOBP_CELLULAR_RESPONSE_TO_FATTY_ACID | 32 | 0.595659941 | 1.765561153 | 0.00621931 | 0.077126896 | 0.065374954 |
| GOBP_NUCLEOTIDE_PHOSPHORYLATION | GOBP_NUCLEOTIDE_PHOSPHORYLATION | 113 | 0.408662089 | 1.534303708 | 0.006270248 | 0.077263666 | 0.065490884 |
| GOBP_RIBOSE_PHOSPHATE_BIOSYNTHETIC_PROCESS | GOBP_RIBOSE_PHOSPHATE_BIOSYNTHETIC_PROCESS | 171 | 0.366357776 | 1.467088711 | 0.006276158 | 0.077263666 | 0.065490884 |
| GOBP_NADPH_REGENERATION | GOBP_NADPH_REGENERATION | 17 | 0.70282567 | 1.796319888 | 0.006277929 | 0.077263666 | 0.065490884 |
| GOBP_NEGATIVE_REGULATION_OF_ORGANELLE_ORGANIZATION | GOBP_NEGATIVE_REGULATION_OF_ORGANELLE_ORGANIZATION | 303 | 0.321152673 | 1.368198594 | 0.006280082 | 0.077263666 | 0.065490884 |
| GOCC_MCM_COMPLEX | GOCC_MCM_COMPLEX | 11 | 0.805183823 | 1.870763554 | 0.006302945 | 0.077391696 | 0.065599406 |
| GOBP_CHRONIC_INFLAMMATORY_RESPONSE | GOBP_CHRONIC_INFLAMMATORY_RESPONSE | 18 | 0.687871094 | 1.810411888 | 0.006345196 | 0.077756816 | 0.065908892 |
| GOBP_NUCLEUS_ORGANIZATION | GOBP_NUCLEUS_ORGANIZATION | 109 | 0.420105402 | 1.581810376 | 0.006383438 | 0.07807146 | 0.066175594 |
| GOBP_MITOTIC_SISTER_CHROMATID_COHESION | GOBP_MITOTIC_SISTER_CHROMATID_COHESION | 26 | 0.629864316 | 1.760091665 | 0.006434877 | 0.078545952 | 0.066577786 |
| GOBP_CELLULAR_RESPONSE_TO_KETONE | GOBP_CELLULAR_RESPONSE_TO_KETONE | 79 | 0.467224561 | 1.672479957 | 0.006465666 | 0.078767031 | 0.066765179 |
| GOMF_COPPER_ION_BINDING | GOMF_COPPER_ION_BINDING | 48 | 0.516828054 | 1.662395366 | 0.006515468 | 0.079092609 | 0.067041149 |
| GOMF_WIDE_PORE_CHANNEL_ACTIVITY | GOMF_WIDE_PORE_CHANNEL_ACTIVITY | 18 | 0.687015291 | 1.808159493 | 0.006517852 | 0.079092609 | 0.067041149 |
| GOCC_NUCLEAR_ENVELOPE | GOCC_NUCLEAR_ENVELOPE | 407 | 0.299910616 | 1.320363347 | 0.006550743 | 0.079156862 | 0.067095611 |
| GOMF_STRUCTURAL_CONSTITUENT_OF_NUCLEAR_PORE | GOMF_STRUCTURAL_CONSTITUENT_OF_NUCLEAR_PORE | 24 | 0.645482488 | 1.764768104 | 0.006560265 | 0.079156862 | 0.067095611 |
| GOCC_NUCLEOID | GOCC_NUCLEOID | 43 | 0.551120472 | 1.719609657 | 0.006561369 | 0.079156862 | 0.067095611 |
| GOBP_MATURATION_OF_SSU_RRNA | GOBP_MATURATION_OF_SSU_RRNA | 49 | 0.538471279 | 1.739623364 | 0.006578635 | 0.079211361 | 0.067141806 |
| GOBP_REGULATION_OF_DOUBLE_STRAND_BREAK_REPAIR | GOBP_REGULATION_OF_DOUBLE_STRAND_BREAK_REPAIR | 78 | 0.482089023 | 1.715539634 | 0.006672563 | 0.080186918 | 0.067968716 |
| GOBP_NEGATIVE_REGULATION_OF_HUMORAL_IMMUNE_RESPONSE | GOBP_NEGATIVE_REGULATION_OF_HUMORAL_IMMUNE_RESPONSE | 13 | -0.733152757 | -1.70148938 | 0.00669022 | 0.080243899 | 0.068017015 |
| GOBP_RESPONSE_TO_ALCOHOL | GOBP_RESPONSE_TO_ALCOHOL | 182 | 0.370153539 | 1.483701179 | 0.006704703 | 0.080262659 | 0.068032916 |
| GOBP_REGULATION_OF_STEROID_BIOSYNTHETIC_PROCESS | GOBP_REGULATION_OF_STEROID_BIOSYNTHETIC_PROCESS | 75 | 0.467913219 | 1.66303959 | 0.006747526 | 0.080619956 | 0.068335771 |
| GOBP_ACTIVATION_OF_INNATE_IMMUNE_RESPONSE | GOBP_ACTIVATION_OF_INNATE_IMMUNE_RESPONSE | 130 | 0.399483215 | 1.53964132 | 0.00680125 | 0.081105886 | 0.068747659 |
| GOBP_CELLULAR_RESPONSE_TO_EXTERNAL_STIMULUS | GOBP_CELLULAR_RESPONSE_TO_EXTERNAL_STIMULUS | 277 | 0.328816171 | 1.39447921 | 0.006849117 | 0.081520235 | 0.069098873 |
| GOBP_PYRIMIDINE_CONTAINING_COMPOUND_BIOSYNTHETIC_PROCESS | GOBP_PYRIMIDINE_CONTAINING_COMPOUND_BIOSYNTHETIC_PROCESS | 36 | 0.580775449 | 1.750174832 | 0.006938126 | 0.082357226 | 0.06980833 |
| GOBP_CELLULAR_AMINO_ACID_METABOLIC_PROCESS | GOBP_CELLULAR_AMINO_ACID_METABOLIC_PROCESS | 286 | 0.321711752 | 1.359383083 | 0.00694595 | 0.082357226 | 0.06980833 |
| GOBP_NUCLEAR_EXPORT | GOBP_NUCLEAR_EXPORT | 188 | 0.363198001 | 1.467117251 | 0.007032866 | 0.083199553 | 0.070522311 |
| GOBP_INTESTINAL_EPITHELIAL_CELL_DIFFERENTIATION | GOBP_INTESTINAL_EPITHELIAL_CELL_DIFFERENTIATION | 16 | 0.701226336 | 1.774952164 | 0.007043774 | 0.083199553 | 0.070522311 |
| GOBP_MONOCYTE_CHEMOTAXIS | GOBP_MONOCYTE_CHEMOTAXIS | 57 | 0.513926041 | 1.710947791 | 0.007071293 | 0.083208228 | 0.070529664 |
| GOBP_SIGNAL_TRANSDUCTION_IN_ABSENCE_OF_LIGAND | GOBP_SIGNAL_TRANSDUCTION_IN_ABSENCE_OF_LIGAND | 57 | 0.513540107 | 1.709662952 | 0.007071293 | 0.083208228 | 0.070529664 |
| GOMF_PHOSPHOLIPASE_ACTIVITY | GOMF_PHOSPHOLIPASE_ACTIVITY | 86 | -0.460415239 | -1.535776968 | 0.007194388 | 0.084496657 | 0.071621773 |
| GOCC_MHC_PROTEIN_COMPLEX | GOCC_MHC_PROTEIN_COMPLEX | 23 | -0.65764505 | -1.755429475 | 0.007260612 | 0.085113554 | 0.072144673 |
| GOBP_EMBRYONIC_EYE_MORPHOGENESIS | GOBP_EMBRYONIC_EYE_MORPHOGENESIS | 29 | 0.620881549 | 1.788343042 | 0.00732308 | 0.08568417 | 0.072628343 |
| GOBP_MEMBRANE_DISASSEMBLY | GOBP_MEMBRANE_DISASSEMBLY | 15 | 0.708002473 | 1.764421164 | 0.007380197 | 0.086190155 | 0.073057231 |
| GOBP_DEVELOPMENT_OF_PRIMARY_SEXUAL_CHARACTERISTICS | GOBP_DEVELOPMENT_OF_PRIMARY_SEXUAL_CHARACTERISTICS | 161 | 0.380372946 | 1.506805299 | 0.007419546 | 0.08641054 | 0.073244036 |
| GOBP_REGULATION_OF_RESPONSE_TO_FOOD | GOBP_REGULATION_OF_RESPONSE_TO_FOOD | 11 | 0.801474344 | 1.862144953 | 0.007433112 | 0.08641054 | 0.073244036 |
| GOBP_MITOCHONDRIAL_MEMBRANE_ORGANIZATION | GOBP_MITOCHONDRIAL_MEMBRANE_ORGANIZATION | 138 | 0.390005328 | 1.507059065 | 0.007489628 | 0.08641054 | 0.073244036 |
| GOBP_PYRIMIDINE_CONTAINING_COMPOUND_METABOLIC_PROCESS | GOBP_PYRIMIDINE_CONTAINING_COMPOUND_METABOLIC_PROCESS | 76 | 0.464608727 | 1.652165306 | 0.007498869 | 0.08641054 | 0.073244036 |
| GOBP_RESPONSE_TO_ELECTRICAL_STIMULUS | GOBP_RESPONSE_TO_ELECTRICAL_STIMULUS | 28 | 0.606575404 | 1.72853921 | 0.007508375 | 0.08641054 | 0.073244036 |
| GOBP_TRANSCRIPTION_COUPLED_NUCLEOTIDE_EXCISION_REPAIR | GOBP_TRANSCRIPTION_COUPLED_NUCLEOTIDE_EXCISION_REPAIR | 71 | 0.467354126 | 1.628506325 | 0.007519584 | 0.08641054 | 0.073244036 |
| GOMF_NADP_RETINOL_DEHYDROGENASE_ACTIVITY | GOMF_NADP_RETINOL_DEHYDROGENASE_ACTIVITY | 10 | 0.794119252 | 1.7710366 | 0.007520048 | 0.08641054 | 0.073244036 |
| GOBP_REGULATION_OF_MRNA_METABOLIC_PROCESS | GOBP_REGULATION_OF_MRNA_METABOLIC_PROCESS | 301 | 0.318944959 | 1.358103985 | 0.007527922 | 0.08641054 | 0.073244036 |
| GOBP_SCF_DEPENDENT_PROTEASOMAL_UBIQUITIN_DEPENDENT_PROTEIN_CATABOLIC_PROCESS | GOBP_SCF_DEPENDENT_PROTEASOMAL_UBIQUITIN_DEPENDENT_PROTEIN_CATABOLIC_PROCESS | 88 | 0.454742776 | 1.645167229 | 0.007527941 | 0.08641054 | 0.073244036 |
| GOBP_SYNAPTIC_SIGNALING | GOBP_SYNAPTIC_SIGNALING | 432 | 0.293603918 | 1.303372072 | 0.007538148 | 0.08641054 | 0.073244036 |
| GOCC_VESICLE_LUMEN | GOCC_VESICLE_LUMEN | 296 | 0.323992879 | 1.381233154 | 0.007568768 | 0.086601758 | 0.073406117 |
| GOBP_POSITIVE_REGULATION_OF_CELL_CYCLE_G2_M_PHASE_TRANSITION | GOBP_POSITIVE_REGULATION_OF_CELL_CYCLE_G2_M_PHASE_TRANSITION | 28 | 0.604728246 | 1.72327542 | 0.007600501 | 0.086804988 | 0.073578381 |
| GOBP_ESTABLISHMENT_OF_PROTEIN_LOCALIZATION_TO_TELOMERE | GOBP_ESTABLISHMENT_OF_PROTEIN_LOCALIZATION_TO_TELOMERE | 17 | 0.696413668 | 1.779931747 | 0.007632856 | 0.087014557 | 0.073756017 |
| GOBP_NEGATIVE_REGULATION_OF_TRANSFERASE_ACTIVITY | GOBP_NEGATIVE_REGULATION_OF_TRANSFERASE_ACTIVITY | 262 | 0.322351215 | 1.351439794 | 0.007669653 | 0.087273913 | 0.073975855 |
| GOMF_FATTY_ACID_SYNTHASE_ACTIVITY | GOMF_FATTY_ACID_SYNTHASE_ACTIVITY | 10 | 0.792829157 | 1.768159444 | 0.00769392 | 0.087367004 | 0.074054761 |
| GOBP_REGULATION_OF_GENE_SILENCING | GOBP_REGULATION_OF_GENE_SILENCING | 125 | -0.428504007 | -1.519352387 | 0.007705958 | 0.087367004 | 0.074054761 |
| GOBP_SENSORY_ORGAN_DEVELOPMENT | GOBP_SENSORY_ORGAN_DEVELOPMENT | 382 | 0.293300248 | 1.279529439 | 0.007808567 | 0.088222423 | 0.074779839 |
| GOCC_KINESIN_COMPLEX | GOCC_KINESIN_COMPLEX | 44 | 0.541599482 | 1.694737846 | 0.007809807 | 0.088222423 | 0.074779839 |
| GOBP_PROTEIN_LOCALIZATION_TO_NUCLEAR_BODY | GOBP_PROTEIN_LOCALIZATION_TO_NUCLEAR_BODY | 12 | 0.768352242 | 1.83129661 | 0.007875285 | 0.088800625 | 0.075269939 |
| GOBP_NEGATIVE_REGULATION_OF_RESPONSE_TO_EXTERNAL_STIMULUS | GOBP_NEGATIVE_REGULATION_OF_RESPONSE_TO_EXTERNAL_STIMULUS | 298 | 0.314793019 | 1.344912315 | 0.007894017 | 0.088850594 | 0.075312294 |
| GOBP_POSITIVE_REGULATION_OF_GLUCOSE_TRANSMEMBRANE_TRANSPORT | GOBP_POSITIVE_REGULATION_OF_GLUCOSE_TRANSMEMBRANE_TRANSPORT | 35 | -0.583618146 | -1.688943272 | 0.007940688 | 0.089210695 | 0.075617526 |
| GOBP_POSITIVE_REGULATION_OF_VASCULAR_ENDOTHELIAL_GROWTH_FACTOR_RECEPTOR_SIGNALING_PATHWAY | GOBP_POSITIVE_REGULATION_OF_VASCULAR_ENDOTHELIAL_GROWTH_FACTOR_RECEPTOR_SIGNALING_PATHWAY | 10 | 0.791099866 | 1.764302796 | 0.007954728 | 0.089210695 | 0.075617526 |
| GOBP_PHOSPHATIDYLCHOLINE_ACYL_CHAIN_REMODELING | GOBP_PHOSPHATIDYLCHOLINE_ACYL_CHAIN_REMODELING | 26 | -0.633129949 | -1.709235028 | 0.007975328 | 0.089217711 | 0.075623474 |
| GOBP_AMMONIUM_ION_METABOLIC_PROCESS | GOBP_AMMONIUM_ION_METABOLIC_PROCESS | 18 | -0.679901192 | -1.688058853 | 0.007989255 | 0.089217711 | 0.075623474 |
| GOBP_CYTOKINETIC_PROCESS | GOBP_CYTOKINETIC_PROCESS | 38 | 0.541330764 | 1.63711324 | 0.007998433 | 0.089217711 | 0.075623474 |
| GOBP_PROTEIN_REFOLDING | GOBP_PROTEIN_REFOLDING | 23 | 0.622095657 | 1.690594002 | 0.00807782 | 0.089941754 | 0.076237193 |
| GOBP_PROTEIN_FOLDING | GOBP_PROTEIN_FOLDING | 199 | 0.366350502 | 1.478094169 | 0.008093387 | 0.089953867 | 0.07624746 |
| GOMF_MAGNESIUM_ION_BINDING | GOMF_MAGNESIUM_ION_BINDING | 192 | 0.355173717 | 1.434961314 | 0.008223093 | 0.090984838 | 0.07712134 |
| GOBP_TELOMERE_MAINTENANCE_VIA_TELOMERE_LENGTHENING | GOBP_TELOMERE_MAINTENANCE_VIA_TELOMERE_LENGTHENING | 73 | 0.466915737 | 1.651955684 | 0.008224433 | 0.090984838 | 0.07712134 |
| GOMF_COLLAGEN_BINDING | GOMF_COLLAGEN_BINDING | 63 | 0.486394741 | 1.658476226 | 0.008235724 | 0.090984838 | 0.07712134 |
| GOBP_POSITIVE_REGULATION_OF_STEROID_BIOSYNTHETIC_PROCESS | GOBP_POSITIVE_REGULATION_OF_STEROID_BIOSYNTHETIC_PROCESS | 14 | 0.752163014 | 1.846291362 | 0.008244723 | 0.090984838 | 0.07712134 |
| GOBP_REGULATION_OF_SYNAPSE_STRUCTURE_OR_ACTIVITY | GOBP_REGULATION_OF_SYNAPSE_STRUCTURE_OR_ACTIVITY | 155 | 0.376258254 | 1.491861305 | 0.008276369 | 0.091139237 | 0.077252213 |
| GOBP_GLUCOSE_METABOLIC_PROCESS | GOBP_GLUCOSE_METABOLIC_PROCESS | 180 | 0.355729889 | 1.425378798 | 0.008294367 | 0.091139237 | 0.077252213 |
| GOMF_MOLECULAR_CARRIER_ACTIVITY | GOMF_MOLECULAR_CARRIER_ACTIVITY | 59 | 0.478903681 | 1.608882858 | 0.008302721 | 0.091139237 | 0.077252213 |
| GOBP_NUCLEOSIDE_SALVAGE | GOBP_NUCLEOSIDE_SALVAGE | 15 | 0.703273931 | 1.75263711 | 0.008364212 | 0.091652288 | 0.07768709 |
| GOBP_PHOSPHATIDYLGLYCEROL_METABOLIC_PROCESS | GOBP_PHOSPHATIDYLGLYCEROL_METABOLIC_PROCESS | 32 | -0.595988473 | -1.693843313 | 0.008414006 | 0.092035595 | 0.078011992 |
| GOBP_CRANIAL_NERVE_MORPHOGENESIS | GOBP_CRANIAL_NERVE_MORPHOGENESIS | 17 | 0.693388068 | 1.772198752 | 0.008445814 | 0.092221161 | 0.078169283 |
| GOBP_POSITIVE_REGULATION_OF_SECRETION | GOBP_POSITIVE_REGULATION_OF_SECRETION | 237 | 0.327561532 | 1.364336854 | 0.008481861 | 0.092392176 | 0.078314241 |
| GOBP_KILLING_OF_CELLS_OF_OTHER_ORGANISM | GOBP_KILLING_OF_CELLS_OF_OTHER_ORGANISM | 41 | 0.519163016 | 1.603778348 | 0.008491217 | 0.092392176 | 0.078314241 |
| GOBP_NEGATIVE_REGULATION_OF_SYNAPTIC_TRANSMISSION | GOBP_NEGATIVE_REGULATION_OF_SYNAPTIC_TRANSMISSION | 42 | -0.553615689 | -1.669284296 | 0.008542845 | 0.092791428 | 0.078652658 |
| GOBP_GLYCOSYL_COMPOUND_BIOSYNTHETIC_PROCESS | GOBP_GLYCOSYL_COMPOUND_BIOSYNTHETIC_PROCESS | 40 | 0.542615832 | 1.669782806 | 0.008622682 | 0.09322793 | 0.079022649 |
| GOBP_PYRIMIDINE_NUCLEOSIDE_BIOSYNTHETIC_PROCESS | GOBP_PYRIMIDINE_NUCLEOSIDE_BIOSYNTHETIC_PROCESS | 15 | 0.702412061 | 1.750489233 | 0.00863258 | 0.09322793 | 0.079022649 |
| GOBP_GLYCOPROTEIN_CATABOLIC_PROCESS | GOBP_GLYCOPROTEIN_CATABOLIC_PROCESS | 23 | 0.617726309 | 1.678719955 | 0.008644679 | 0.09322793 | 0.079022649 |
| GOBP_POSITIVE_REGULATION_OF_DNA_BIOSYNTHETIC_PROCESS | GOBP_POSITIVE_REGULATION_OF_DNA_BIOSYNTHETIC_PROCESS | 65 | 0.480808361 | 1.657620886 | 0.008658217 | 0.09322793 | 0.079022649 |
| GOBP_MUSCLE_ORGAN_DEVELOPMENT | GOBP_MUSCLE_ORGAN_DEVELOPMENT | 234 | 0.332724316 | 1.378843133 | 0.008680144 | 0.09322793 | 0.079022649 |
| GOBP_COLLAGEN_BIOSYNTHETIC_PROCESS | GOBP_COLLAGEN_BIOSYNTHETIC_PROCESS | 41 | 0.518841599 | 1.602785441 | 0.008683107 | 0.09322793 | 0.079022649 |
| GOBP_STEM_CELL_DIFFERENTIATION | GOBP_STEM_CELL_DIFFERENTIATION | 213 | 0.343785168 | 1.397892355 | 0.008688069 | 0.09322793 | 0.079022649 |
| GOBP_HEPATOCYTE_DIFFERENTIATION | GOBP_HEPATOCYTE_DIFFERENTIATION | 15 | 0.701610638 | 1.748491999 | 0.008722036 | 0.093431047 | 0.079194817 |
| GOBP_PROTEIN_LOCALIZATION_TO_NUCLEUS | GOBP_PROTEIN_LOCALIZATION_TO_NUCLEUS | 252 | 0.325999046 | 1.357512887 | 0.008750102 | 0.093570363 | 0.079312905 |
| GOBP_MEMORY | GOBP_MEMORY | 68 | -0.476947014 | -1.536715505 | 0.008814811 | 0.093931726 | 0.079619207 |
| GOBP_RESPONSE_TO_ETHANOL | GOBP_RESPONSE_TO_ETHANOL | 94 | 0.433797277 | 1.599761659 | 0.008825634 | 0.093931726 | 0.079619207 |
| GOBP_MATURATION_OF_5_8S_RRNA | GOBP_MATURATION_OF_5_8S_RRNA | 35 | 0.587230118 | 1.769467059 | 0.00882925 | 0.093931726 | 0.079619207 |
| GOBP_REGULATION_OF_RESPONSE_TO_BIOTIC_STIMULUS | GOBP_REGULATION_OF_RESPONSE_TO_BIOTIC_STIMULUS | 358 | 0.304878099 | 1.324466511 | 0.008852775 | 0.09402101 | 0.079694886 |
| GOBP_NEGATIVE_REGULATION_OF_RESPONSE_TO_WOUNDING | GOBP_NEGATIVE_REGULATION_OF_RESPONSE_TO_WOUNDING | 74 | 0.466840756 | 1.653215236 | 0.008874073 | 0.094086375 | 0.079750292 |
| GOBP_OSSIFICATION | GOBP_OSSIFICATION | 343 | 0.308853655 | 1.331674125 | 0.008898126 | 0.094180676 | 0.079830224 |
| GOBP_POSITIVE_REGULATION_OF_CALCIUM_ION_TRANSPORT | GOBP_POSITIVE_REGULATION_OF_CALCIUM_ION_TRANSPORT | 90 | 0.428920588 | 1.559538788 | 0.008983234 | 0.094919422 | 0.080456406 |
| GOBP_ICOSANOID_METABOLIC_PROCESS | GOBP_ICOSANOID_METABOLIC_PROCESS | 91 | -0.453267381 | -1.525272641 | 0.00901069 | 0.094919422 | 0.080456406 |
| GOMF_SODIUM_ION_TRANSMEMBRANE_TRANSPORTER_ACTIVITY | GOMF_SODIUM_ION_TRANSMEMBRANE_TRANSPORTER_ACTIVITY | 77 | -0.475161447 | -1.545918273 | 0.009013755 | 0.094919422 | 0.080456406 |
| GOBP_NEGATIVE_REGULATION_OF_DNA_DEPENDENT_DNA_REPLICATION | GOBP_NEGATIVE_REGULATION_OF_DNA_DEPENDENT_DNA_REPLICATION | 17 | 0.686474912 | 1.754529734 | 0.009078115 | 0.09543541 | 0.080893772 |
| GOMF_ALDITOL_NADPPLUS_1_OXIDOREDUCTASE_ACTIVITY | GOMF_ALDITOL_NADPPLUS_1_OXIDOREDUCTASE_ACTIVITY | 10 | 0.785777433 | 1.752432761 | 0.009173547 | 0.096275751 | 0.081606069 |
| GOBP_BASE_EXCISION_REPAIR | GOBP_BASE_EXCISION_REPAIR | 39 | 0.550654629 | 1.678762574 | 0.009234604 | 0.096516845 | 0.081810427 |
| GOBP_REPLICATIVE_SENESCENCE | GOBP_REPLICATIVE_SENESCENCE | 15 | 0.694654753 | 1.731157156 | 0.009259653 | 0.096516845 | 0.081810427 |
| GOCC_SPINDLE_POLE_CENTROSOME | GOCC_SPINDLE_POLE_CENTROSOME | 15 | 0.696166652 | 1.734924977 | 0.009259653 | 0.096516845 | 0.081810427 |
| GOMF_STEROL_TRANSFER_ACTIVITY | GOMF_STEROL_TRANSFER_ACTIVITY | 15 | 0.696744136 | 1.736364133 | 0.009259653 | 0.096516845 | 0.081810427 |
| GOBP_RNA_DEPENDENT_DNA_BIOSYNTHETIC_PROCESS | GOBP_RNA_DEPENDENT_DNA_BIOSYNTHETIC_PROCESS | 67 | 0.480321317 | 1.64961702 | 0.009285014 | 0.096516845 | 0.081810427 |
| GOBP_RESPIRATORY_GASEOUS_EXCHANGE_BY_RESPIRATORY_SYSTEM | GOBP_RESPIRATORY_GASEOUS_EXCHANGE_BY_RESPIRATORY_SYSTEM | 53 | -0.520615092 | -1.628626386 | 0.009289727 | 0.096516845 | 0.081810427 |
| GOMF_ATP_DEPENDENT_MICROTUBULE_MOTOR_ACTIVITY_PLUS_END_DIRECTED | GOMF_ATP_DEPENDENT_MICROTUBULE_MOTOR_ACTIVITY_PLUS_END_DIRECTED | 15 | 0.694506234 | 1.730787032 | 0.009439446 | 0.097908649 | 0.08299016 |
| GOBP_QUINONE_METABOLIC_PROCESS | GOBP_QUINONE_METABOLIC_PROCESS | 31 | 0.559854949 | 1.639068938 | 0.00946867 | 0.098048075 | 0.083108341 |
| GOBP_POSITIVE_REGULATION_OF_TELOMERE_MAINTENANCE | GOBP_POSITIVE_REGULATION_OF_TELOMERE_MAINTENANCE | 48 | 0.501876629 | 1.614303588 | 0.009552706 | 0.098753677 | 0.08370643 |
| GOBP_RIBOSOME_ASSEMBLY | GOBP_RIBOSOME_ASSEMBLY | 60 | 0.482957446 | 1.622546063 | 0.009699674 | 0.100083675 | 0.084833774 |
| GOCC_AXON | GOCC_AXON | 469 | 0.28284937 | 1.242305115 | 0.009727991 | 0.100083675 | 0.084833774 |
| GOBP_INSULIN_LIKE_GROWTH_FACTOR_RECEPTOR_SIGNALING_PATHWAY | GOBP_INSULIN_LIKE_GROWTH_FACTOR_RECEPTOR_SIGNALING_PATHWAY | 25 | 0.630547194 | 1.736162527 | 0.009729686 | 0.100083675 | 0.084833774 |
| GOBP_APPENDAGE_DEVELOPMENT | GOBP_APPENDAGE_DEVELOPMENT | 141 | 0.379707504 | 1.473519392 | 0.009774859 | 0.100382149 | 0.085086769 |
| GOBP_PHOSPHATIDYLINOSITOL_ACYL_CHAIN_REMODELING | GOBP_PHOSPHATIDYLINOSITOL_ACYL_CHAIN_REMODELING | 15 | -0.710632588 | -1.719681724 | 0.00981089 | 0.100502406 | 0.085188702 |
| GOBP_NEGATIVE_REGULATION_OF_DNA_REPLICATION | GOBP_NEGATIVE_REGULATION_OF_DNA_REPLICATION | 30 | 0.580430177 | 1.682584791 | 0.009818922 | 0.100502406 | 0.085188702 |
| GOBP_RESPONSE_TO_CORTICOSTEROID | GOBP_RESPONSE_TO_CORTICOSTEROID | 115 | 0.397354844 | 1.504023325 | 0.00989533 | 0.101117905 | 0.085710416 |
| GOMF_GDP_BINDING | GOMF_GDP_BINDING | 69 | 0.475314413 | 1.647794994 | 0.00995512 | 0.101561836 | 0.086086706 |
| GOBP_REGULATION_OF_HORMONE_METABOLIC_PROCESS | GOBP_REGULATION_OF_HORMONE_METABOLIC_PROCESS | 30 | 0.580173176 | 1.68183978 | 0.010005943 | 0.101674762 | 0.086182425 |
| GOCC_HOST_CELLULAR_COMPONENT | GOCC_HOST_CELLULAR_COMPONENT | 59 | 0.472048443 | 1.585852601 | 0.010013566 | 0.101674762 | 0.086182425 |
| GOBP_MACROPHAGE_PROLIFERATION | GOBP_MACROPHAGE_PROLIFERATION | 11 | -0.772134708 | -1.716390422 | 0.010015283 | 0.101674762 | 0.086182425 |
| GOCC_SPLICEOSOMAL_COMPLEX | GOCC_SPLICEOSOMAL_COMPLEX | 183 | 0.351374368 | 1.415954444 | 0.010048796 | 0.101810324 | 0.08629733 |
| GOBP_INTERMEDIATE_FILAMENT_ORGANIZATION | GOBP_INTERMEDIATE_FILAMENT_ORGANIZATION | 16 | 0.686036043 | 1.736502319 | 0.01006141 | 0.101810324 | 0.08629733 |
| GOBP_ENDOCRINE_SYSTEM_DEVELOPMENT | GOBP_ENDOCRINE_SYSTEM_DEVELOPMENT | 90 | 0.42543892 | 1.546879579 | 0.010085459 | 0.101887731 | 0.086362943 |
| GOCC_PRESYNAPSE | GOCC_PRESYNAPSE | 341 | 0.298956546 | 1.291233129 | 0.010121939 | 0.102090276 | 0.086534626 |
| GOBP_REGULATION_OF_CELL_DIVISION | GOBP_REGULATION_OF_CELL_DIVISION | 136 | 0.380216783 | 1.464391319 | 0.010202358 | 0.102734602 | 0.087080775 |
| GOBP_PROTEASOMAL_UBIQUITIN_INDEPENDENT_PROTEIN_CATABOLIC_PROCESS | GOBP_PROTEASOMAL_UBIQUITIN_INDEPENDENT_PROTEIN_CATABOLIC_PROCESS | 20 | 0.657898759 | 1.749844855 | 0.010264038 | 0.103188463 | 0.08746548 |
| GOMF_CADHERIN_BINDING_INVOLVED_IN_CELL_CELL_ADHESION | GOMF_CADHERIN_BINDING_INVOLVED_IN_CELL_CELL_ADHESION | 18 | 0.662738466 | 1.744265178 | 0.010323947 | 0.103568695 | 0.087787777 |
| GOCC_SMALL_NUCLEAR_RIBONUCLEOPROTEIN_COMPLEX | GOCC_SMALL_NUCLEAR_RIBONUCLEOPROTEIN_COMPLEX | 83 | -0.465183691 | -1.54312149 | 0.010335199 | 0.103568695 | 0.087787777 |
| GOMF_FATTY_ACID_BINDING | GOMF_FATTY_ACID_BINDING | 30 | 0.579181585 | 1.678965299 | 0.010379986 | 0.103850009 | 0.088026226 |
| GOBP_REGULATION_OF_STEROID_METABOLIC_PROCESS | GOBP_REGULATION_OF_STEROID_METABOLIC_PROCESS | 99 | 0.405859944 | 1.504861051 | 0.010402509 | 0.103908023 | 0.088075401 |
| GOBP_RIBONUCLEOSIDE_TRIPHOSPHATE_BIOSYNTHETIC_PROCESS | GOBP_RIBONUCLEOSIDE_TRIPHOSPHATE_BIOSYNTHETIC_PROCESS | 67 | 0.475994095 | 1.634755596 | 0.010439072 | 0.104105862 | 0.088243094 |
| GOBP_PEPTIDYL_METHIONINE_MODIFICATION | GOBP_PEPTIDYL_METHIONINE_MODIFICATION | 13 | 0.708433148 | 1.727843328 | 0.010670963 | 0.106029848 | 0.08987392 |
| GOCC_SYNAPTONEMAL_STRUCTURE | GOCC_SYNAPTONEMAL_STRUCTURE | 25 | 0.622754457 | 1.714705833 | 0.010674287 | 0.106029848 | 0.08987392 |
| GOBP_MITOTIC_NUCLEAR_ENVELOPE_DISASSEMBLY | GOBP_MITOTIC_NUCLEAR_ENVELOPE_DISASSEMBLY | 12 | 0.753553742 | 1.796025753 | 0.010694892 | 0.106029848 | 0.08987392 |
| GOBP_NEGATIVE_REGULATION_OF_SIGNAL_TRANSDUCTION_IN_ABSENCE_OF_LIGAND | GOBP_NEGATIVE_REGULATION_OF_SIGNAL_TRANSDUCTION_IN_ABSENCE_OF_LIGAND | 26 | 0.603486505 | 1.68638156 | 0.01070026 | 0.106029848 | 0.08987392 |
| GOBP_POSTSYNAPSE_ORGANIZATION | GOBP_POSTSYNAPSE_ORGANIZATION | 125 | 0.39041269 | 1.495263053 | 0.010787488 | 0.106579185 | 0.090339553 |
| GOBP_VIRAL_GENE_EXPRESSION | GOBP_VIRAL_GENE_EXPRESSION | 197 | 0.340561324 | 1.373719243 | 0.010812714 | 0.106579185 | 0.090339553 |
| GOBP_ODONTOGENESIS | GOBP_ODONTOGENESIS | 93 | 0.415154174 | 1.52522689 | 0.010838213 | 0.106579185 | 0.090339553 |
| GOBP_FATTY_ACID_ELONGATION | GOBP_FATTY_ACID_ELONGATION | 13 | 0.707001638 | 1.724351926 | 0.010844471 | 0.106579185 | 0.090339553 |
| GOCC_GOLGI_LUMEN | GOCC_GOLGI_LUMEN | 73 | 0.455482197 | 1.61150363 | 0.010851132 | 0.106579185 | 0.090339553 |
| GOBP_RIBONUCLEOSIDE_MONOPHOSPHATE_METABOLIC_PROCESS | GOBP_RIBONUCLEOSIDE_MONOPHOSPHATE_METABOLIC_PROCESS | 49 | 0.518578275 | 1.675355618 | 0.01087167 | 0.106579185 | 0.090339553 |
| GOCC_PRECATALYTIC_SPLICEOSOME | GOCC_PRECATALYTIC_SPLICEOSOME | 53 | 0.495798675 | 1.630416825 | 0.010876139 | 0.106579185 | 0.090339553 |
| GOBP_POSITIVE_REGULATION_OF_CATION_TRANSMEMBRANE_TRANSPORT | GOBP_POSITIVE_REGULATION_OF_CATION_TRANSMEMBRANE_TRANSPORT | 105 | 0.41357992 | 1.548312995 | 0.010896 | 0.106579185 | 0.090339553 |
| GOMF_GROWTH_FACTOR_ACTIVITY | GOMF_GROWTH_FACTOR_ACTIVITY | 104 | 0.408180613 | 1.529270633 | 0.010922028 | 0.106579185 | 0.090339553 |
| GOBP_TISSUE_REGENERATION | GOBP_TISSUE_REGENERATION | 57 | 0.497598052 | 1.65658912 | 0.010940564 | 0.106579185 | 0.090339553 |
| GOBP_PHOSPHATIDYLINOSITOL_METABOLIC_PROCESS | GOBP_PHOSPHATIDYLINOSITOL_METABOLIC_PROCESS | 171 | -0.387801968 | -1.430149622 | 0.010944394 | 0.106579185 | 0.090339553 |
| GOBP_POSITIVE_REGULATION_OF_AMINE_TRANSPORT | GOBP_POSITIVE_REGULATION_OF_AMINE_TRANSPORT | 20 | 0.657335502 | 1.748346732 | 0.010978041 | 0.106739544 | 0.090475478 |
| GOMF_RNA_BINDING_INVOLVED_IN_POSTTRANSCRIPTIONAL_GENE_SILENCING | GOMF_RNA_BINDING_INVOLVED_IN_POSTTRANSCRIPTIONAL_GENE_SILENCING | 23 | -0.644932657 | -1.721496718 | 0.011043378 | 0.107207047 | 0.090871747 |
| GOBP_POSITIVE_REGULATION_OF_DEFENSE_RESPONSE | GOBP_POSITIVE_REGULATION_OF_DEFENSE_RESPONSE | 301 | 0.314046661 | 1.337246472 | 0.011065404 | 0.10725328 | 0.090910935 |
| GOBP_NEGATIVE_REGULATION_OF_BLOOD_CIRCULATION | GOBP_NEGATIVE_REGULATION_OF_BLOOD_CIRCULATION | 18 | -0.669467988 | -1.662155292 | 0.011106954 | 0.107488325 | 0.091110166 |
| GOBP_NUCLEOTIDE_EXCISION_REPAIR | GOBP_NUCLEOTIDE_EXCISION_REPAIR | 102 | 0.409676662 | 1.526169441 | 0.011154486 | 0.107575244 | 0.091183841 |
| GOBP_NUCLEOSIDE_MONOPHOSPHATE_METABOLIC_PROCESS | GOBP_NUCLEOSIDE_MONOPHOSPHATE_METABOLIC_PROCESS | 65 | 0.471033572 | 1.623921608 | 0.011165499 | 0.107575244 | 0.091183841 |
| GOBP_TRANSLATIONAL_ELONGATION | GOBP_TRANSLATIONAL_ELONGATION | 131 | 0.386766645 | 1.493066145 | 0.011190054 | 0.107575244 | 0.091183841 |
| GOBP_POSITIVE_REGULATION_OF_CHROMOSOME_SEGREGATION | GOBP_POSITIVE_REGULATION_OF_CHROMOSOME_SEGREGATION | 13 | 0.704199115 | 1.717516671 | 0.011191486 | 0.107575244 | 0.091183841 |
| GOBP_MATURATION_OF_LSU_RRNA | GOBP_MATURATION_OF_LSU_RRNA | 28 | 0.58807246 | 1.67581194 | 0.011202508 | 0.107575244 | 0.091183841 |
| GOBP_POSITIVE_REGULATION_OF_NUCLEAR_DIVISION | GOBP_POSITIVE_REGULATION_OF_NUCLEAR_DIVISION | 43 | 0.519820999 | 1.62194884 | 0.011233869 | 0.107709917 | 0.091297994 |
| GOBP_INNATE_IMMUNE_RESPONSE_ACTIVATING_SIGNAL_TRANSDUCTION | GOBP_INNATE_IMMUNE_RESPONSE_ACTIVATING_SIGNAL_TRANSDUCTION | 103 | 0.416056022 | 1.553387533 | 0.011288474 | 0.10785924 | 0.091424564 |
| GOCC_REPLISOME | GOCC_REPLISOME | 23 | 0.606026182 | 1.646923938 | 0.011298402 | 0.10785924 | 0.091424564 |
| GOBP_SURFACTANT_HOMEOSTASIS | GOBP_SURFACTANT_HOMEOSTASIS | 11 | -0.76544265 | -1.701514542 | 0.011307561 | 0.10785924 | 0.091424564 |
| GOBP_WATER_TRANSPORT | GOBP_WATER_TRANSPORT | 14 | -0.716707962 | -1.705933031 | 0.011318884 | 0.10785924 | 0.091424564 |
| GOMF_ALCOHOL_DEHYDROGENASE_NADPPLUS_ACTIVITY | GOMF_ALCOHOL_DEHYDROGENASE_NADPPLUS_ACTIVITY | 17 | 0.677075524 | 1.730506271 | 0.011347019 | 0.107961763 | 0.091511465 |
| GOBP_ESTABLISHMENT_OF_RNA_LOCALIZATION | GOBP_ESTABLISHMENT_OF_RNA_LOCALIZATION | 184 | 0.355991344 | 1.436546204 | 0.011439179 | 0.108553235 | 0.092012814 |
| GOBP_CRANIAL_SKELETAL_SYSTEM_DEVELOPMENT | GOBP_CRANIAL_SKELETAL_SYSTEM_DEVELOPMENT | 50 | 0.514412892 | 1.672404879 | 0.011444128 | 0.108553235 | 0.092012814 |
| GOBP_LONG_TERM_SYNAPTIC_DEPRESSION | GOBP_LONG_TERM_SYNAPTIC_DEPRESSION | 21 | -0.652686956 | -1.702163119 | 0.011524359 | 0.109115244 | 0.092489189 |
| GOBP_DEFENSE_RESPONSE_TO_FUNGUS | GOBP_DEFENSE_RESPONSE_TO_FUNGUS | 18 | 0.655464953 | 1.725121977 | 0.011538502 | 0.109115244 | 0.092489189 |
| GOMF_LIGASE_ACTIVITY_FORMING_CARBON_OXYGEN_BONDS | GOMF_LIGASE_ACTIVITY_FORMING_CARBON_OXYGEN_BONDS | 41 | 0.504794778 | 1.559392544 | 0.011666861 | 0.110161407 | 0.093375947 |
| GOBP_DNA_SYNTHESIS_INVOLVED_IN_DNA_REPAIR | GOBP_DNA_SYNTHESIS_INVOLVED_IN_DNA_REPAIR | 48 | 0.491649587 | 1.581407954 | 0.011716861 | 0.110465641 | 0.093633824 |
| GOCC_CAJAL_BODY | GOCC_CAJAL_BODY | 65 | -0.488221426 | -1.556192249 | 0.011954508 | 0.112375383 | 0.095252576 |
| GOBP_NEGATIVE_REGULATION_OF_RESPONSE_TO_DNA_DAMAGE_STIMULUS | GOBP_NEGATIVE_REGULATION_OF_RESPONSE_TO_DNA_DAMAGE_STIMULUS | 79 | 0.448038752 | 1.603802316 | 0.011955598 | 0.112375383 | 0.095252576 |
| GOMF_NUCLEAR_LOCALIZATION_SEQUENCE_BINDING | GOMF_NUCLEAR_LOCALIZATION_SEQUENCE_BINDING | 22 | 0.611134122 | 1.653475204 | 0.012093571 | 0.113500539 | 0.09620629 |
| GOBP_NEGATIVE_REGULATION_OF_INTRINSIC_APOPTOTIC_SIGNALING_PATHWAY | GOBP_NEGATIVE_REGULATION_OF_INTRINSIC_APOPTOTIC_SIGNALING_PATHWAY | 87 | 0.4226338 | 1.529885147 | 0.01215069 | 0.113864611 | 0.096514888 |
| GOBP_MEGAKARYOCYTE_DIFFERENTIATION | GOBP_MEGAKARYOCYTE_DIFFERENTIATION | 89 | -0.459739217 | -1.544037005 | 0.012187842 | 0.114040753 | 0.096664191 |
| GOCC_PROTEASOME_CORE_COMPLEX | GOCC_PROTEASOME_CORE_COMPLEX | 18 | 0.653145705 | 1.719017937 | 0.012232535 | 0.114286827 | 0.09687277 |
| GOMF_NUCLEOCYTOPLASMIC_CARRIER_ACTIVITY | GOMF_NUCLEOCYTOPLASMIC_CARRIER_ACTIVITY | 31 | 0.551647888 | 1.615041395 | 0.012281064 | 0.114567948 | 0.097111057 |
| GOBP_REGULATION_OF_MICROTUBULE_BASED_MOVEMENT | GOBP_REGULATION_OF_MICROTUBULE_BASED_MOVEMENT | 33 | -0.571381663 | -1.635892544 | 0.012345179 | 0.114737229 | 0.097254544 |
| GOBP_PHOSPHATIDYLETHANOLAMINE_METABOLIC_PROCESS | GOBP_PHOSPHATIDYLETHANOLAMINE_METABOLIC_PROCESS | 31 | -0.567535539 | -1.599018275 | 0.012364982 | 0.114737229 | 0.097254544 |
| GOBP_POSITIVE_REGULATION_OF_DNA_REPAIR | GOBP_POSITIVE_REGULATION_OF_DNA_REPAIR | 66 | 0.468245385 | 1.606057211 | 0.012371434 | 0.114737229 | 0.097254544 |
| GOBP_POSITIVE_REGULATION_OF_EPITHELIAL_TO_MESENCHYMAL_TRANSITION | GOBP_POSITIVE_REGULATION_OF_EPITHELIAL_TO_MESENCHYMAL_TRANSITION | 46 | 0.511646134 | 1.627547785 | 0.01237308 | 0.114737229 | 0.097254544 |
| GOBP_REGULATION_OF_NEURON_PROJECTION_DEVELOPMENT | GOBP_REGULATION_OF_NEURON_PROJECTION_DEVELOPMENT | 359 | 0.298897668 | 1.2986288 | 0.012426286 | 0.115058892 | 0.097527195 |
| GOBP_POSITIVE_REGULATION_OF_GLUCOSE_IMPORT | GOBP_POSITIVE_REGULATION_OF_GLUCOSE_IMPORT | 30 | -0.588486127 | -1.649386168 | 0.012508691 | 0.115613751 | 0.097997509 |
| GOBP_KERATINOCYTE_PROLIFERATION | GOBP_KERATINOCYTE_PROLIFERATION | 39 | 0.540874435 | 1.648946018 | 0.012535634 | 0.115613751 | 0.097997509 |
| GOBP_REGULATION_OF_TELOMERE_MAINTENANCE | GOBP_REGULATION_OF_TELOMERE_MAINTENANCE | 75 | 0.447446979 | 1.59029925 | 0.012542036 | 0.115613751 | 0.097997509 |
| GOBP_CELL_ADHESION_MEDIATED_BY_INTEGRIN | GOBP_CELL_ADHESION_MEDIATED_BY_INTEGRIN | 68 | 0.453555636 | 1.562386271 | 0.012650023 | 0.116436435 | 0.09869484 |
| GOMF_CALCIUM_ION_BINDING | GOMF_CALCIUM_ION_BINDING | 490 | 0.284484899 | 1.259899884 | 0.012724436 | 0.116702635 | 0.098920478 |
| GOBP_HOMOLOGOUS_RECOMBINATION | GOBP_HOMOLOGOUS_RECOMBINATION | 44 | 0.519015821 | 1.624070524 | 0.01273514 | 0.116702635 | 0.098920478 |
| GOBP_VASCULAR_ENDOTHELIAL_CELL_PROLIFERATION | GOBP_VASCULAR_ENDOTHELIAL_CELL_PROLIFERATION | 22 | -0.622262486 | -1.6429139 | 0.012735295 | 0.116702635 | 0.098920478 |
| GOBP_RESPONSE_TO_GAMMA_RADIATION | GOBP_RESPONSE_TO_GAMMA_RADIATION | 52 | 0.49035856 | 1.598413079 | 0.01280952 | 0.117209935 | 0.09935048 |
| GOBP_PEPTIDYL_ARGININE_MODIFICATION | GOBP_PEPTIDYL_ARGININE_MODIFICATION | 18 | 0.650509798 | 1.712080478 | 0.013100077 | 0.119652987 | 0.101421281 |
| GOBP_NUCLEOSIDE_DIPHOSPHATE_METABOLIC_PROCESS | GOBP_NUCLEOSIDE_DIPHOSPHATE_METABOLIC_PROCESS | 134 | 0.380293927 | 1.461616591 | 0.01311503 | 0.119652987 | 0.101421281 |
| GOBP_ADULT_BEHAVIOR | GOBP_ADULT_BEHAVIOR | 76 | 0.442296678 | 1.572822857 | 0.013226302 | 0.12044025 | 0.102088587 |
| GOBP_CELLULAR_RESPONSE_TO_IONIZING_RADIATION | GOBP_CELLULAR_RESPONSE_TO_IONIZING_RADIATION | 62 | 0.475587248 | 1.613742053 | 0.013240092 | 0.12044025 | 0.102088587 |
| GOBP_CELLULAR_RESPONSE_TO_EXTRACELLULAR_STIMULUS | GOBP_CELLULAR_RESPONSE_TO_EXTRACELLULAR_STIMULUS | 213 | 0.334746842 | 1.361140897 | 0.013383325 | 0.121409072 | 0.102909788 |
| GOBP_REGULATION_OF_CELL_CYCLE_CHECKPOINT | GOBP_REGULATION_OF_CELL_CYCLE_CHECKPOINT | 31 | 0.547988663 | 1.604328404 | 0.013406027 | 0.121409072 | 0.102909788 |
| GOMF_CHEMOKINE_RECEPTOR_BINDING | GOMF_CHEMOKINE_RECEPTOR_BINDING | 52 | 0.489159316 | 1.594503924 | 0.013414682 | 0.121409072 | 0.102909788 |
| GOBP_REGULATION_OF_REPRODUCTIVE_PROCESS | GOBP_REGULATION_OF_REPRODUCTIVE_PROCESS | 100 | 0.407599987 | 1.512235843 | 0.01342476 | 0.121409072 | 0.102909788 |
| GOBP_REGULATION_OF_MEIOTIC_NUCLEAR_DIVISION | GOBP_REGULATION_OF_MEIOTIC_NUCLEAR_DIVISION | 19 | 0.64996973 | 1.717004003 | 0.013444815 | 0.121413719 | 0.102913727 |
| GOBP_PROTEIN_HYDROXYLATION | GOBP_PROTEIN_HYDROXYLATION | 26 | 0.594396912 | 1.660981618 | 0.013491569 | 0.121659097 | 0.103121717 |
| GOBP_EYELID_DEVELOPMENT_IN_CAMERA_TYPE_EYE | GOBP_EYELID_DEVELOPMENT_IN_CAMERA_TYPE_EYE | 11 | 0.778416236 | 1.808571761 | 0.013541791 | 0.121934999 | 0.103355579 |
| GOBP_REGULATION_OF_PEPTIDYL_THREONINE_PHOSPHORYLATION | GOBP_REGULATION_OF_PEPTIDYL_THREONINE_PHOSPHORYLATION | 41 | 0.497456398 | 1.536723102 | 0.013595227 | 0.122132322 | 0.103522836 |
| GOBP_CELLULAR_RESPONSE_TO_CADMIUM_ION | GOBP_CELLULAR_RESPONSE_TO_CADMIUM_ION | 32 | 0.566569138 | 1.679334785 | 0.013627847 | 0.122132322 | 0.103522836 |
| GOBP_COLLAGEN_FIBRIL_ORGANIZATION | GOBP_COLLAGEN_FIBRIL_ORGANIZATION | 50 | 0.503251705 | 1.636118807 | 0.013633389 | 0.122132322 | 0.103522836 |
| GOBP_REGULATION_OF_HORMONE_SECRETION | GOBP_REGULATION_OF_HORMONE_SECRETION | 204 | 0.345612185 | 1.395613386 | 0.013642336 | 0.122132322 | 0.103522836 |
| GOBP_POSITIVE_REGULATION_OF_CYTOKINESIS | GOBP_POSITIVE_REGULATION_OF_CYTOKINESIS | 26 | 0.594249258 | 1.660569014 | 0.013677657 | 0.122272347 | 0.103641524 |
| GOBP_DEVELOPMENTAL_GROWTH | GOBP_DEVELOPMENTAL_GROWTH | 490 | 0.284073327 | 1.25807715 | 0.013758921 | 0.122662278 | 0.103972041 |
| GOCC_PRERIBOSOME_LARGE_SUBUNIT_PRECURSOR | GOCC_PRERIBOSOME_LARGE_SUBUNIT_PRECURSOR | 23 | 0.597920153 | 1.624895164 | 0.013766907 | 0.122662278 | 0.103972041 |
| GOBP_MRNA_3_END_PROCESSING | GOBP_MRNA_3_END_PROCESSING | 95 | 0.407572553 | 1.50638033 | 0.013780504 | 0.122662278 | 0.103972041 |
| GOBP_EMBRYONIC_HEART_TUBE_DEVELOPMENT | GOBP_EMBRYONIC_HEART_TUBE_DEVELOPMENT | 59 | -0.483101722 | -1.528211066 | 0.013804549 | 0.122700519 | 0.104004455 |
| GOBP_ALPHA_AMINO_ACID_METABOLIC_PROCESS | GOBP_ALPHA_AMINO_ACID_METABOLIC_PROCESS | 157 | 0.371599876 | 1.469898311 | 0.013834438 | 0.122786483 | 0.104077321 |
| GOMF_GLYCOSAMINOGLYCAN_BINDING | GOMF_GLYCOSAMINOGLYCAN_BINDING | 181 | 0.348345374 | 1.396682422 | 0.013875838 | 0.122786483 | 0.104077321 |
| GOBP_RESPONSE_TO_MOLECULE_OF_BACTERIAL_ORIGIN | GOBP_RESPONSE_TO_MOLECULE_OF_BACTERIAL_ORIGIN | 287 | 0.315871635 | 1.331757537 | 0.013888168 | 0.122786483 | 0.104077321 |
| GOBP_NEGATIVE_REGULATION_OF_REPRODUCTIVE_PROCESS | GOBP_NEGATIVE_REGULATION_OF_REPRODUCTIVE_PROCESS | 36 | 0.553487532 | 1.667942319 | 0.013944774 | 0.122786483 | 0.104077321 |
| GOCC_METHYLOSOME | GOCC_METHYLOSOME | 12 | 0.740746554 | 1.765501001 | 0.013946078 | 0.122786483 | 0.104077321 |
| GOBP_PROTEIN_LOCALIZATION_TO_CHROMOSOME_TELOMERIC_REGION | GOBP_PROTEIN_LOCALIZATION_TO_CHROMOSOME_TELOMERIC_REGION | 29 | 0.590044028 | 1.699520839 | 0.013968508 | 0.122786483 | 0.104077321 |
| GOBP_SPERM_AXONEME_ASSEMBLY | GOBP_SPERM_AXONEME_ASSEMBLY | 18 | -0.658409353 | -1.634698911 | 0.013968694 | 0.122786483 | 0.104077321 |
| GOBP_RESPONSE_TO_MECHANICAL_STIMULUS | GOBP_RESPONSE_TO_MECHANICAL_STIMULUS | 160 | 0.350981768 | 1.391002026 | 0.013972323 | 0.122786483 | 0.104077321 |
| GOBP_ATP_SYNTHESIS_COUPLED_ELECTRON_TRANSPORT | GOBP_ATP_SYNTHESIS_COUPLED_ELECTRON_TRANSPORT | 97 | 0.409262728 | 1.512788361 | 0.013997515 | 0.122834126 | 0.104117705 |
| GOBP_CELLULAR_RESPONSE_TO_DRUG | GOBP_CELLULAR_RESPONSE_TO_DRUG | 52 | 0.488045564 | 1.590873444 | 0.014019844 | 0.122856547 | 0.104136709 |
| GOBP_EPITHELIAL_CELL_DEVELOPMENT | GOBP_EPITHELIAL_CELL_DEVELOPMENT | 171 | 0.348281903 | 1.394703436 | 0.014112984 | 0.123355672 | 0.104559782 |
| GOBP_SEROTONIN_TRANSPORT | GOBP_SEROTONIN_TRANSPORT | 12 | -0.719103076 | -1.636920084 | 0.014116511 | 0.123355672 | 0.104559782 |
| GOBP_NUCLEOBASE_CONTAINING_SMALL_MOLECULE_CATABOLIC_PROCESS | GOBP_NUCLEOBASE_CONTAINING_SMALL_MOLECULE_CATABOLIC_PROCESS | 45 | 0.508017398 | 1.595498814 | 0.014144899 | 0.123430136 | 0.1046229 |
| GOBP_VITAMIN_METABOLIC_PROCESS | GOBP_VITAMIN_METABOLIC_PROCESS | 111 | 0.390686833 | 1.46771849 | 0.014206335 | 0.123626787 | 0.104789586 |
| GOBP_POSITIVE_REGULATION_OF_TRANSLATION | GOBP_POSITIVE_REGULATION_OF_TRANSLATION | 119 | 0.382038738 | 1.451101026 | 0.014216904 | 0.123626787 | 0.104789586 |
| GOBP_METAL_ION_HOMEOSTASIS | GOBP_METAL_ION_HOMEOSTASIS | 485 | 0.281653421 | 1.241121376 | 0.014236109 | 0.123626787 | 0.104789586 |
| GOBP_SEX_DIFFERENTIATION | GOBP_SEX_DIFFERENTIATION | 192 | 0.344073043 | 1.390112729 | 0.014256506 | 0.123626787 | 0.104789586 |
| GOMF_DNA_BINDING_BENDING | GOMF_DNA_BINDING_BENDING | 14 | 0.720266568 | 1.767996988 | 0.014266925 | 0.123626787 | 0.104789586 |
| GOBP_RESPONSE_TO_UV_A | GOBP_RESPONSE_TO_UV_A | 12 | 0.737520219 | 1.757811331 | 0.014288308 | 0.123639638 | 0.104800479 |
| GOBP_REGULATION_OF_PEPTIDASE_ACTIVITY | GOBP_REGULATION_OF_PEPTIDASE_ACTIVITY | 349 | 0.296006306 | 1.285375992 | 0.014333677 | 0.123859718 | 0.104987025 |
| GOBP_FEEDING_BEHAVIOR | GOBP_FEEDING_BEHAVIOR | 45 | 0.506518156 | 1.590790238 | 0.014546166 | 0.125300425 | 0.10620821 |
| GOBP_TELOMERE_CAPPING | GOBP_TELOMERE_CAPPING | 49 | -0.51665539 | -1.589834719 | 0.014551919 | 0.125300425 | 0.10620821 |
| GOBP_CELLULAR_RESPONSE_TO_RADIATION | GOBP_CELLULAR_RESPONSE_TO_RADIATION | 157 | 0.370492184 | 1.465516731 | 0.014568992 | 0.125300425 | 0.10620821 |
| GOBP_REGULATION_OF_HISTONE_H3_K4_METHYLATION | GOBP_REGULATION_OF_HISTONE_H3_K4_METHYLATION | 26 | 0.59269619 | 1.656229123 | 0.014608096 | 0.125300425 | 0.10620821 |
| GOBP_GANGLION_DEVELOPMENT | GOBP_GANGLION_DEVELOPMENT | 14 | 0.718542799 | 1.763765751 | 0.014621378 | 0.125300425 | 0.10620821 |
| GOBP_GLUTAMINE_FAMILY_AMINO_ACID_METABOLIC_PROCESS | GOBP_GLUTAMINE_FAMILY_AMINO_ACID_METABOLIC_PROCESS | 57 | 0.482407251 | 1.606016343 | 0.014621408 | 0.125300425 | 0.10620821 |
| GOMF_ENDORIBONUCLEASE_ACTIVITY_PRODUCING_5_PHOSPHOMONOESTERS | GOMF_ENDORIBONUCLEASE_ACTIVITY_PRODUCING_5_PHOSPHOMONOESTERS | 27 | 0.59227625 | 1.668866714 | 0.014647706 | 0.125352893 | 0.106252683 |
| GOBP_REGIONALIZATION | GOBP_REGIONALIZATION | 241 | 0.323146921 | 1.344776566 | 0.014752 | 0.126071768 | 0.106862022 |
| GOBP_NEGATIVE_REGULATION_OF_CELLULAR_SENESCENCE | GOBP_NEGATIVE_REGULATION_OF_CELLULAR_SENESCENCE | 17 | 0.666387187 | 1.703188441 | 0.014796448 | 0.126277929 | 0.10703677 |
| GOMF_TRANSLATION_INITIATION_FACTOR_ACTIVITY | GOMF_TRANSLATION_INITIATION_FACTOR_ACTIVITY | 48 | 0.477816019 | 1.536911803 | 0.014867547 | 0.126543411 | 0.1072618 |
| GOBP_LABYRINTHINE_LAYER_DEVELOPMENT | GOBP_LABYRINTHINE_LAYER_DEVELOPMENT | 40 | 0.525793379 | 1.618015348 | 0.014868291 | 0.126543411 | 0.1072618 |
| GOBP_REGULATION_OF_GRANULOCYTE_CHEMOTAXIS | GOBP_REGULATION_OF_GRANULOCYTE_CHEMOTAXIS | 46 | 0.504053171 | 1.603394549 | 0.01494663 | 0.127036131 | 0.107679444 |
| GOBP_FLUID_TRANSPORT | GOBP_FLUID_TRANSPORT | 24 | -0.607499166 | -1.627078287 | 0.014985585 | 0.127193225 | 0.107812601 |
| GOBP_REGULATION_OF_MEIOTIC_CELL_CYCLE | GOBP_REGULATION_OF_MEIOTIC_CELL_CYCLE | 31 | 0.545029811 | 1.59566587 | 0.015093472 | 0.127782158 | 0.108311797 |
| GOBP_GLYCOSYL_COMPOUND_CATABOLIC_PROCESS | GOBP_GLYCOSYL_COMPOUND_CATABOLIC_PROCESS | 37 | 0.526415566 | 1.598157496 | 0.015096106 | 0.127782158 | 0.108311797 |
| GOBP_CARBOHYDRATE_BIOSYNTHETIC_PROCESS | GOBP_CARBOHYDRATE_BIOSYNTHETIC_PROCESS | 178 | 0.352951158 | 1.408934498 | 0.015124042 | 0.127844455 | 0.108364602 |
| GOMF_CYSTEINE_TYPE_ENDOPEPTIDASE_ACTIVITY_INVOLVED_IN_APOPTOTIC_PROCESS | GOMF_CYSTEINE_TYPE_ENDOPEPTIDASE_ACTIVITY_INVOLVED_IN_APOPTOTIC_PROCESS | 13 | 0.691301924 | 1.686060879 | 0.01518218 | 0.128161524 | 0.108633359 |
| GOBP_SPLICEOSOMAL_SNRNP_ASSEMBLY | GOBP_SPLICEOSOMAL_SNRNP_ASSEMBLY | 49 | -0.513207714 | -1.579225645 | 0.015273485 | 0.128757347 | 0.109138395 |
| GOBP_CYTOSKELETON_DEPENDENT_CYTOKINESIS | GOBP_CYTOSKELETON_DEPENDENT_CYTOKINESIS | 89 | 0.420444938 | 1.51787522 | 0.015311974 | 0.128906907 | 0.109265166 |
| GOBP_PEPTIDYL_ASPARAGINE_MODIFICATION | GOBP_PEPTIDYL_ASPARAGINE_MODIFICATION | 31 | 0.544344847 | 1.593660524 | 0.015468461 | 0.130048101 | 0.110232475 |
| GOCC_RESPIRATORY_CHAIN_COMPLEX | GOCC_RESPIRATORY_CHAIN_COMPLEX | 81 | 0.414263555 | 1.490622299 | 0.015524676 | 0.130344344 | 0.110483579 |
| GOBP_EYE_MORPHOGENESIS | GOBP_EYE_MORPHOGENESIS | 107 | 0.400367052 | 1.500843776 | 0.015555581 | 0.13042756 | 0.110554115 |
| GOMF_SULFUR_COMPOUND_BINDING | GOMF_SULFUR_COMPOUND_BINDING | 213 | 0.330749218 | 1.344885837 | 0.015677545 | 0.131268535 | 0.11126695 |
| GOBP_POSITIVE_REGULATION_OF_MITOCHONDRION_ORGANIZATION | GOBP_POSITIVE_REGULATION_OF_MITOCHONDRION_ORGANIZATION | 79 | 0.442423278 | 1.583701131 | 0.015698136 | 0.131268535 | 0.11126695 |
| GOBP_APOPTOTIC_MITOCHONDRIAL_CHANGES | GOBP_APOPTOTIC_MITOCHONDRIAL_CHANGES | 103 | 0.404091354 | 1.508716228 | 0.015759032 | 0.131598062 | 0.111546266 |
| GOBP_HISTONE_MRNA_METABOLIC_PROCESS | GOBP_HISTONE_MRNA_METABOLIC_PROCESS | 24 | 0.602075877 | 1.646093153 | 0.015803642 | 0.131598062 | 0.111546266 |
| GOBP_TERMINATION_OF_RNA_POLYMERASE_II_TRANSCRIPTION | GOBP_TERMINATION_OF_RNA_POLYMERASE_II_TRANSCRIPTION | 35 | 0.56518677 | 1.703045096 | 0.015827265 | 0.131598062 | 0.111546266 |
| GOBP_REGULATION_OF_ESTABLISHMENT_OF_PROTEIN_LOCALIZATION_TO_CHROMOSOME | GOBP_REGULATION_OF_ESTABLISHMENT_OF_PROTEIN_LOCALIZATION_TO_CHROMOSOME | 12 | 0.732917064 | 1.746840135 | 0.015828348 | 0.131598062 | 0.111546266 |
| GOBP_RNA_5_END_PROCESSING | GOBP_RNA_5_END_PROCESSING | 22 | 0.592787041 | 1.603835621 | 0.015843449 | 0.131598062 | 0.111546266 |
| GOBP_NEGATIVE_REGULATION_OF_CHONDROCYTE_DIFFERENTIATION | GOBP_NEGATIVE_REGULATION_OF_CHONDROCYTE_DIFFERENTIATION | 20 | 0.642571687 | 1.709078706 | 0.01597608 | 0.132326917 | 0.112164064 |
| GOCC_GERM_CELL_NUCLEUS | GOCC_GERM_CELL_NUCLEUS | 12 | 0.731893003 | 1.744399381 | 0.015999464 | 0.132326917 | 0.112164064 |
| GOBP_NEGATIVE_REGULATION_OF_CELL_DEVELOPMENT | GOBP_NEGATIVE_REGULATION_OF_CELL_DEVELOPMENT | 135 | 0.373905867 | 1.439764496 | 0.016034237 | 0.132326917 | 0.112164064 |
| GOBP_IMP_METABOLIC_PROCESS | GOBP_IMP_METABOLIC_PROCESS | 13 | 0.687265327 | 1.676215762 | 0.016049724 | 0.132326917 | 0.112164064 |
| GOMF_INSULIN_LIKE_GROWTH_FACTOR_I_BINDING | GOMF_INSULIN_LIKE_GROWTH_FACTOR_I_BINDING | 13 | 0.687696666 | 1.677267782 | 0.016049724 | 0.132326917 | 0.112164064 |
| GOBP_POSITIVE_REGULATION_OF_CHROMOSOME_ORGANIZATION | GOBP_POSITIVE_REGULATION_OF_CHROMOSOME_ORGANIZATION | 152 | 0.372724871 | 1.469972768 | 0.016071486 | 0.132326917 | 0.112164064 |
| GOBP_CELLULAR_OXIDANT_DETOXIFICATION | GOBP_CELLULAR_OXIDANT_DETOXIFICATION | 80 | 0.429537067 | 1.541361799 | 0.016113975 | 0.132326917 | 0.112164064 |
| GOBP_REGULATION_OF_GLUCOSE_IMPORT | GOBP_REGULATION_OF_GLUCOSE_IMPORT | 46 | -0.509124946 | -1.551206234 | 0.016131834 | 0.132326917 | 0.112164064 |
| GOBP_MALE_SEX_DIFFERENTIATION | GOBP_MALE_SEX_DIFFERENTIATION | 113 | 0.384329374 | 1.442947608 | 0.01613572 | 0.132326917 | 0.112164064 |
| GOBP_POSITIVE_REGULATION_OF_RELEASE_OF_CYTOCHROME_C_FROM_MITOCHONDRIA | GOBP_POSITIVE_REGULATION_OF_RELEASE_OF_CYTOCHROME_C_FROM_MITOCHONDRIA | 25 | 0.608062236 | 1.674251949 | 0.016153007 | 0.132326917 | 0.112164064 |
| GOBP_RESPONSE_TO_TOPOLOGICALLY_INCORRECT_PROTEIN | GOBP_RESPONSE_TO_TOPOLOGICALLY_INCORRECT_PROTEIN | 194 | 0.333619181 | 1.350459386 | 0.01616548 | 0.132326917 | 0.112164064 |
| GOBP_NEURONAL_STEM_CELL_POPULATION_MAINTENANCE | GOBP_NEURONAL_STEM_CELL_POPULATION_MAINTENANCE | 18 | 0.640989427 | 1.687023759 | 0.016223232 | 0.132624925 | 0.112416664 |
| GOBP_ORGANIC_HYDROXY_COMPOUND_BIOSYNTHETIC_PROCESS | GOBP_ORGANIC_HYDROXY_COMPOUND_BIOSYNTHETIC_PROCESS | 205 | 0.332787829 | 1.341852515 | 0.016291256 | 0.132981924 | 0.112719267 |
| GOMF_DYNEIN_LIGHT_INTERMEDIATE_CHAIN_BINDING | GOMF_DYNEIN_LIGHT_INTERMEDIATE_CHAIN_BINDING | 23 | -0.626330666 | -1.671843059 | 0.01630971 | 0.132981924 | 0.112719267 |
| GOBP_NEGATIVE_REGULATION_OF_DNA_REPAIR | GOBP_NEGATIVE_REGULATION_OF_DNA_REPAIR | 35 | 0.563658264 | 1.698439335 | 0.016402795 | 0.133565619 | 0.113214023 |
| GOBP_NEGATIVE_REGULATION_OF_CALCIUM_ION_TRANSMEMBRANE_TRANSPORTER_ACTIVITY | GOBP_NEGATIVE_REGULATION_OF_CALCIUM_ION_TRANSMEMBRANE_TRANSPORTER_ACTIVITY | 24 | -0.602666463 | -1.614134751 | 0.016471727 | 0.13375537 | 0.113374862 |
| GOBP_SECRETION_BY_TISSUE | GOBP_SECRETION_BY_TISSUE | 20 | -0.638353077 | -1.646460088 | 0.016495236 | 0.13375537 | 0.113374862 |
| GOBP_HUMORAL_IMMUNE_RESPONSE | GOBP_HUMORAL_IMMUNE_RESPONSE | 281 | -0.345432519 | -1.346774146 | 0.016499327 | 0.13375537 | 0.113374862 |
| GOBP_HISTONE_SERINE_PHOSPHORYLATION | GOBP_HISTONE_SERINE_PHOSPHORYLATION | 10 | 0.763320983 | 1.702350616 | 0.016512211 | 0.13375537 | 0.113374862 |
| GOCC_SITE_OF_DOUBLE_STRAND_BREAK | GOCC_SITE_OF_DOUBLE_STRAND_BREAK | 65 | 0.456235845 | 1.572905397 | 0.01659724 | 0.134269081 | 0.113810298 |
| GOBP_MEMBRANE_REPOLARIZATION | GOBP_MEMBRANE_REPOLARIZATION | 32 | -0.570480143 | -1.621346754 | 0.016664237 | 0.13463577 | 0.114121114 |
| GOBP_RESPONSE_TO_INTERLEUKIN_12 | GOBP_RESPONSE_TO_INTERLEUKIN_12 | 43 | 0.501785106 | 1.565673132 | 0.016704159 | 0.134734041 | 0.114204411 |
| GOBP_REGULATION_OF_STEM_CELL_POPULATION_MAINTENANCE | GOBP_REGULATION_OF_STEM_CELL_POPULATION_MAINTENANCE | 25 | 0.606722005 | 1.670561726 | 0.016719772 | 0.134734041 | 0.114204411 |
| GOBP_REGULATION_OF_DNA_BIOSYNTHETIC_PROCESS | GOBP_REGULATION_OF_DNA_BIOSYNTHETIC_PROCESS | 101 | 0.405436764 | 1.510770498 | 0.016917311 | 0.136149293 | 0.115404019 |
| GOBP_PYRIMIDINE_NUCLEOBASE_METABOLIC_PROCESS | GOBP_PYRIMIDINE_NUCLEOBASE_METABOLIC_PROCESS | 15 | 0.672967577 | 1.677110295 | 0.016990826 | 0.136511886 | 0.115711364 |
| GOBP_POSITIVE_REGULATION_OF_MACROPHAGE_CHEMOTAXIS | GOBP_POSITIVE_REGULATION_OF_MACROPHAGE_CHEMOTAXIS | 16 | 0.655139898 | 1.658297642 | 0.017006309 | 0.136511886 | 0.115711364 |
| GOBP_POSITIVE_REGULATION_OF_CALCIUM_ION_TRANSPORT_INTO_CYTOSOL | GOBP_POSITIVE_REGULATION_OF_CALCIUM_ION_TRANSPORT_INTO_CYTOSOL | 41 | 0.489085148 | 1.510862961 | 0.017066296 | 0.136816642 | 0.115969684 |
| GOBP_NEGATIVE_REGULATION_OF_NOTCH_SIGNALING_PATHWAY | GOBP_NEGATIVE_REGULATION_OF_NOTCH_SIGNALING_PATHWAY | 35 | 0.56254938 | 1.695097999 | 0.017170169 | 0.137471984 | 0.11652517 |
| GOBP_POSITIVE_REGULATION_OF_HORMONE_METABOLIC_PROCESS | GOBP_POSITIVE_REGULATION_OF_HORMONE_METABOLIC_PROCESS | 11 | 0.771481014 | 1.792458471 | 0.017211134 | 0.137622623 | 0.116652856 |
| GOMF_UBIQUITIN_LIKE_PROTEIN_CONJUGATING_ENZYME_ACTIVITY | GOMF_UBIQUITIN_LIKE_PROTEIN_CONJUGATING_ENZYME_ACTIVITY | 34 | 0.536089321 | 1.598399713 | 0.017349025 | 0.138307786 | 0.11723362 |
| GOBP_NUCLEOSIDE_TRIPHOSPHATE_METABOLIC_PROCESS | GOBP_NUCLEOSIDE_TRIPHOSPHATE_METABOLIC_PROCESS | 101 | 0.404674682 | 1.507930771 | 0.017353881 | 0.138307786 | 0.11723362 |
| GOBP_CDC42_PROTEIN_SIGNAL_TRANSDUCTION | GOBP_CDC42_PROTEIN_SIGNAL_TRANSDUCTION | 12 | 0.728343749 | 1.735940063 | 0.017368389 | 0.138307786 | 0.11723362 |
| GOMF_STRUCTURAL_CONSTITUENT_OF_SYNAPSE | GOMF_STRUCTURAL_CONSTITUENT_OF_SYNAPSE | 11 | 0.770921781 | 1.791159151 | 0.017385865 | 0.138307786 | 0.11723362 |
| GOBP_REGULATION_OF_TRANSCRIPTION_FROM_RNA_POLYMERASE_II_PROMOTER_IN_RESPONSE_TO_HYPOXIA | GOBP_REGULATION_OF_TRANSCRIPTION_FROM_RNA_POLYMERASE_II_PROMOTER_IN_RESPONSE_TO_HYPOXIA | 74 | 0.444579486 | 1.574381779 | 0.017423405 | 0.138429179 | 0.117336515 |
| GOMF_CATALYTIC_ACTIVITY_ACTING_ON_A_TRNA | GOMF_CATALYTIC_ACTIVITY_ACTING_ON_A_TRNA | 120 | 0.386995254 | 1.472287121 | 0.01756229 | 0.139035685 | 0.117850607 |
| GOCC_RIBOSOME | GOCC_RIBOSOME | 219 | 0.327558067 | 1.33711878 | 0.017568016 | 0.139035685 | 0.117850607 |
| GOBP_GAMETE_GENERATION | GOBP_GAMETE_GENERATION | 438 | 0.283656608 | 1.255015597 | 0.017587027 | 0.139035685 | 0.117850607 |
| GOMF_NEUROPEPTIDE_RECEPTOR_BINDING | GOMF_NEUROPEPTIDE_RECEPTOR_BINDING | 18 | 0.637131283 | 1.676869486 | 0.017611304 | 0.139035685 | 0.117850607 |
| GOBP_NEGATIVE_REGULATION_OF_KINASE_ACTIVITY | GOBP_NEGATIVE_REGULATION_OF_KINASE_ACTIVITY | 235 | 0.322746622 | 1.337371503 | 0.017629658 | 0.139035685 | 0.117850607 |
| GOBP_SNRNA_TRANSCRIPTION | GOBP_SNRNA_TRANSCRIPTION | 72 | 0.447183891 | 1.567787677 | 0.017640328 | 0.139035685 | 0.117850607 |
| GOCC_RIBOSOMAL_SUBUNIT | GOCC_RIBOSOMAL_SUBUNIT | 183 | 0.337667829 | 1.360720379 | 0.017656391 | 0.139035685 | 0.117850607 |
| GOBP_REGULATION_OF_CELLULAR_RESPONSE_TO_GROWTH_FACTOR_STIMULUS | GOBP_REGULATION_OF_CELLULAR_RESPONSE_TO_GROWTH_FACTOR_STIMULUS | 228 | 0.330057233 | 1.365258037 | 0.01770447 | 0.139123338 | 0.117924905 |
| GOCC_CHROMOCENTER | GOCC_CHROMOCENTER | 12 | 0.726140297 | 1.730688336 | 0.017712307 | 0.139123338 | 0.117924905 |
| GOBP_CARDIAC_MYOFIBRIL_ASSEMBLY | GOBP_CARDIAC_MYOFIBRIL_ASSEMBLY | 10 | 0.761725318 | 1.698791982 | 0.017735327 | 0.139128266 | 0.117929082 |
| GOBP_ACUTE_INFLAMMATORY_RESPONSE | GOBP_ACUTE_INFLAMMATORY_RESPONSE | 80 | 0.42450164 | 1.523292545 | 0.017777331 | 0.13921779 | 0.118004964 |
| GOMF_DISULFIDE_OXIDOREDUCTASE_ACTIVITY | GOMF_DISULFIDE_OXIDOREDUCTASE_ACTIVITY | 36 | 0.543023829 | 1.636409807 | 0.017791554 | 0.13921779 | 0.118004964 |
| GOBP_RESPONSE_TO_STARVATION | GOBP_RESPONSE_TO_STARVATION | 176 | 0.35554028 | 1.428352689 | 0.017910435 | 0.139919634 | 0.118599868 |
| GOBP_REGULATION_OF_IMMUNE_EFFECTOR_PROCESS | GOBP_REGULATION_OF_IMMUNE_EFFECTOR_PROCESS | 405 | 0.284039594 | 1.247491509 | 0.017926288 | 0.139919634 | 0.118599868 |
| GOCC_COPI_COATED_VESICLE_MEMBRANE | GOCC_COPI_COATED_VESICLE_MEMBRANE | 18 | 0.636746361 | 1.675856409 | 0.017960031 | 0.140007119 | 0.118674022 |
| GOBP_NEGATIVE_REGULATION_OF_MUSCLE_HYPERTROPHY | GOBP_NEGATIVE_REGULATION_OF_MUSCLE_HYPERTROPHY | 24 | 0.597792886 | 1.634383329 | 0.018047921 | 0.140515958 | 0.119105329 |
| GOBP_REGULATION_OF_NUCLEASE_ACTIVITY | GOBP_REGULATION_OF_NUCLEASE_ACTIVITY | 22 | 0.587677809 | 1.59001216 | 0.018093382 | 0.140664568 | 0.119231295 |
| GOCC_RESPIRASOME | GOCC_RESPIRASOME | 96 | 0.392272277 | 1.446507 | 0.018120741 | 0.140664568 | 0.119231295 |
| GOCC_LARGE_RIBOSOMAL_SUBUNIT | GOCC_LARGE_RIBOSOMAL_SUBUNIT | 112 | 0.390689002 | 1.463865712 | 0.01813493 | 0.140664568 | 0.119231295 |
| GOBP_BLASTOCYST_GROWTH | GOBP_BLASTOCYST_GROWTH | 17 | 0.655880741 | 1.676335496 | 0.018245888 | 0.141348641 | 0.119811135 |
| GOBP_NEGATIVE_REGULATION_OF_NERVOUS_SYSTEM_DEVELOPMENT | GOBP_NEGATIVE_REGULATION_OF_NERVOUS_SYSTEM_DEVELOPMENT | 105 | 0.395854835 | 1.481955856 | 0.018268624 | 0.141348641 | 0.119811135 |
| GOMF_OLIGOSACCHARIDE_BINDING | GOMF_OLIGOSACCHARIDE_BINDING | 11 | -0.733503941 | -1.630517481 | 0.01831136 | 0.141503084 | 0.119942045 |
| GOBP_ESTABLISHMENT_OF_TISSUE_POLARITY | GOBP_ESTABLISHMENT_OF_TISSUE_POLARITY | 116 | 0.383426824 | 1.451741108 | 0.018377868 | 0.141840611 | 0.120228143 |
| GOBP_REGULATION_OF_LYMPHOCYTE_CHEMOTAXIS | GOBP_REGULATION_OF_LYMPHOCYTE_CHEMOTAXIS | 23 | 0.582840552 | 1.58391516 | 0.018514051 | 0.142560246 | 0.120838126 |
| GOCC_U5_SNRNP | GOCC_U5_SNRNP | 23 | -0.619978895 | -1.654888493 | 0.018517 | 0.142560246 | 0.120838126 |
| GOBP_REGULATION_OF_GENE_SILENCING_BY_RNA | GOBP_REGULATION_OF_GENE_SILENCING_BY_RNA | 109 | -0.407447344 | -1.416247143 | 0.01859091 | 0.142952136 | 0.121170303 |
| GOBP_GLYCOPROTEIN_METABOLIC_PROCESS | GOBP_GLYCOPROTEIN_METABOLIC_PROCESS | 354 | 0.28957255 | 1.258873459 | 0.018687652 | 0.143518393 | 0.121650279 |
| GOBP_POSITIVE_REGULATION_OF_SMOOTH_MUSCLE_CONTRACTION | GOBP_POSITIVE_REGULATION_OF_SMOOTH_MUSCLE_CONTRACTION | 16 | 0.652585602 | 1.65183218 | 0.018790568 | 0.144073679 | 0.122120954 |
| GOBP_CELLULAR_PROTEIN_COMPLEX_DISASSEMBLY | GOBP_CELLULAR_PROTEIN_COMPLEX_DISASSEMBLY | 206 | 0.333571905 | 1.3469098 | 0.018806334 | 0.144073679 | 0.122120954 |
| GOBP_CELL_SUBSTRATE_ADHESION | GOBP_CELL_SUBSTRATE_ADHESION | 313 | 0.294013135 | 1.259292745 | 0.018835133 | 0.144116605 | 0.122157339 |
| GOMF_SNORNA_BINDING | GOMF_SNORNA_BINDING | 32 | 0.554649119 | 1.644003347 | 0.018890944 | 0.144365851 | 0.122368608 |
| GOBP_POSITIVE_REGULATION_OF_NERVOUS_SYSTEM_DEVELOPMENT | GOBP_POSITIVE_REGULATION_OF_NERVOUS_SYSTEM_DEVELOPMENT | 204 | 0.33439898 | 1.350333445 | 0.018940187 | 0.144564351 | 0.122536862 |
| GOCC_FLEMMING_BODY | GOCC_FLEMMING_BODY | 31 | 0.534470532 | 1.564751816 | 0.019030855 | 0.14507816 | 0.122972381 |
| GOBP_NEGATIVE_REGULATION_OF_EXTRINSIC_APOPTOTIC_SIGNALING_PATHWAY | GOBP_NEGATIVE_REGULATION_OF_EXTRINSIC_APOPTOTIC_SIGNALING_PATHWAY | 91 | 0.417228712 | 1.518420276 | 0.019105994 | 0.145472473 | 0.123306612 |
| GOMF_GLYCEROPHOSPHOLIPID_FLIPPASE_ACTIVITY | GOMF_GLYCEROPHOSPHOLIPID_FLIPPASE_ACTIVITY | 12 | -0.708504677 | -1.612794569 | 0.019129725 | 0.145474882 | 0.123308654 |
| GOBP_POSITIVE_REGULATION_OF_CYTOKINE_PRODUCTION | GOBP_POSITIVE_REGULATION_OF_CYTOKINE_PRODUCTION | 375 | 0.282393093 | 1.228774862 | 0.019193291 | 0.14561021 | 0.123423362 |
| GOBP_POSITIVE_REGULATION_OF_TRANSCRIPTION_REGULATORY_REGION_DNA_BINDING | GOBP_POSITIVE_REGULATION_OF_TRANSCRIPTION_REGULATORY_REGION_DNA_BINDING | 20 | 0.631540085 | 1.679737427 | 0.019194393 | 0.14561021 | 0.123423362 |
| GOBP_REGULATION_OF_PEPTIDE_SECRETION | GOBP_REGULATION_OF_PEPTIDE_SECRETION | 245 | 0.325520275 | 1.355599614 | 0.019353427 | 0.146637615 | 0.12429422 |
| GOCC_IMMUNOGLOBULIN_COMPLEX | GOCC_IMMUNOGLOBULIN_COMPLEX | 141 | 0.362140044 | 1.405345883 | 0.019402208 | 0.146828158 | 0.124455729 |
| GOBP_RESPONSE_TO_ENDOPLASMIC_RETICULUM_STRESS | GOBP_RESPONSE_TO_ENDOPLASMIC_RETICULUM_STRESS | 279 | 0.310072291 | 1.317108587 | 0.019448581 | 0.147000039 | 0.124601421 |
| GOMF_RNA_HELICASE_ACTIVITY | GOMF_RNA_HELICASE_ACTIVITY | 71 | 0.434195074 | 1.512962835 | 0.01963712 | 0.147741912 | 0.125230253 |
| GOBP_REGULATION_OF_UBIQUITIN_PROTEIN_LIGASE_ACTIVITY | GOBP_REGULATION_OF_UBIQUITIN_PROTEIN_LIGASE_ACTIVITY | 23 | 0.579146366 | 1.573875917 | 0.019655238 | 0.147741912 | 0.125230253 |
| GOBP_POSITIVE_REGULATION_OF_LAMELLIPODIUM_ORGANIZATION | GOBP_POSITIVE_REGULATION_OF_LAMELLIPODIUM_ORGANIZATION | 34 | 0.529008524 | 1.577287666 | 0.019675128 | 0.147741912 | 0.125230253 |
| GOCC_GAP_JUNCTION | GOCC_GAP_JUNCTION | 18 | 0.634858331 | 1.670887292 | 0.019703671 | 0.147741912 | 0.125230253 |
| GOBP_RESPONSE_TO_LAMINAR_FLUID_SHEAR_STRESS | GOBP_RESPONSE_TO_LAMINAR_FLUID_SHEAR_STRESS | 13 | -0.69497552 | -1.612888249 | 0.019712687 | 0.147741912 | 0.125230253 |
| GOBP_NEGATIVE_REGULATION_OF_PROTEOLYSIS | GOBP_NEGATIVE_REGULATION_OF_PROTEOLYSIS | 255 | 0.316189811 | 1.319120268 | 0.019728412 | 0.147741912 | 0.125230253 |
| GOMF_SIGNAL_SEQUENCE_BINDING | GOMF_SIGNAL_SEQUENCE_BINDING | 42 | 0.518676095 | 1.60516944 | 0.019738295 | 0.147741912 | 0.125230253 |
| GOBP_NEGATIVE_REGULATION_OF_DNA_BINDING | GOBP_NEGATIVE_REGULATION_OF_DNA_BINDING | 45 | 0.49787608 | 1.563648604 | 0.01976265 | 0.147741912 | 0.125230253 |
| GOBP_SYMPATHETIC_NERVOUS_SYSTEM_DEVELOPMENT | GOBP_SYMPATHETIC_NERVOUS_SYSTEM_DEVELOPMENT | 14 | 0.702374381 | 1.724078062 | 0.019769692 | 0.147741912 | 0.125230253 |
| GOBP_RESPONSE_TO_VITAMIN_D | GOBP_RESPONSE_TO_VITAMIN_D | 29 | 0.573231647 | 1.651095652 | 0.019784528 | 0.147741912 | 0.125230253 |
| GOMF_PROTEIN_N_TERMINUS_BINDING | GOMF_PROTEIN_N_TERMINUS_BINDING | 99 | 0.389828423 | 1.445418841 | 0.01981312 | 0.147777809 | 0.125260681 |
| GOMF_CARBOXYLIC_ACID_BINDING | GOMF_CARBOXYLIC_ACID_BINDING | 140 | 0.371686654 | 1.441775798 | 0.019985254 | 0.148882957 | 0.126197435 |
| GOBP_POSITIVE_REGULATION_OF_COLLAGEN_METABOLIC_PROCESS | GOBP_POSITIVE_REGULATION_OF_COLLAGEN_METABOLIC_PROCESS | 26 | 0.57898431 | 1.617912672 | 0.020010154 | 0.148889927 | 0.126203343 |
| GOBP_NUCLEOTIDE_EXCISION_REPAIR_DNA_GAP_FILLING | GOBP_NUCLEOTIDE_EXCISION_REPAIR_DNA_GAP_FILLING | 23 | 0.577542217 | 1.569516517 | 0.020036881 | 0.148910459 | 0.126220747 |
| GOBP_INTRACILIARY_TRANSPORT | GOBP_INTRACILIARY_TRANSPORT | 53 | -0.490453097 | -1.534271418 | 0.020098281 | 0.149188319 | 0.126456269 |
| GOBP_POSITIVE_REGULATION_OF_CELL_AGING | GOBP_POSITIVE_REGULATION_OF_CELL_AGING | 14 | 0.701565054 | 1.722091455 | 0.020125893 | 0.149215001 | 0.126478886 |
| GOBP_CELLULAR_RESPONSE_TO_COPPER_ION | GOBP_CELLULAR_RESPONSE_TO_COPPER_ION | 22 | 0.582303433 | 1.57547133 | 0.020161365 | 0.149299831 | 0.12655079 |
| GOBP_EMBRYONIC_CAMERA_TYPE_EYE_MORPHOGENESIS | GOBP_EMBRYONIC_CAMERA_TYPE_EYE_MORPHOGENESIS | 21 | 0.623660436 | 1.669917848 | 0.020202604 | 0.149427121 | 0.126658685 |
| GOBP_POSITIVE_REGULATION_OF_LAMELLIPODIUM_ASSEMBLY | GOBP_POSITIVE_REGULATION_OF_LAMELLIPODIUM_ASSEMBLY | 26 | 0.575931534 | 1.609382002 | 0.020384165 | 0.150494578 | 0.127563492 |
| GOMF_PEPTIDE_DISULFIDE_OXIDOREDUCTASE_ACTIVITY | GOMF_PEPTIDE_DISULFIDE_OXIDOREDUCTASE_ACTIVITY | 13 | 0.673628614 | 1.642956303 | 0.020401128 | 0.150494578 | 0.127563492 |
| GOCC_CELL_DIVISION_SITE | GOCC_CELL_DIVISION_SITE | 65 | 0.449245932 | 1.548807176 | 0.020419593 | 0.150494578 | 0.127563492 |
| GOBP_POSITIVE_REGULATION_OF_MICROTUBULE_POLYMERIZATION_OR_DEPOLYMERIZATION | GOBP_POSITIVE_REGULATION_OF_MICROTUBULE_POLYMERIZATION_OR_DEPOLYMERIZATION | 29 | 0.570972319 | 1.644588045 | 0.020538202 | 0.150938076 | 0.127939413 |
| GOBP_REGULATION_OF_GENE_EXPRESSION_EPIGENETIC | GOBP_REGULATION_OF_GENE_EXPRESSION_EPIGENETIC | 175 | -0.373628815 | -1.382109117 | 0.020544638 | 0.150938076 | 0.127939413 |
| GOBP_CHONDROCYTE_DEVELOPMENT | GOBP_CHONDROCYTE_DEVELOPMENT | 27 | 0.578907167 | 1.631196424 | 0.02055265 | 0.150938076 | 0.127939413 |
| GOBP_INSULIN_SECRETION | GOBP_INSULIN_SECRETION | 161 | 0.360372459 | 1.427575582 | 0.020676697 | 0.151669796 | 0.128559639 |
| GOMF_FLIPPASE_ACTIVITY | GOMF_FLIPPASE_ACTIVITY | 15 | -0.680243525 | -1.646142294 | 0.020736399 | 0.151928355 | 0.128778801 |
| GOBP_REGULATION_OF_PROTEIN_LOCALIZATION_TO_CHROMOSOME_TELOMERIC_REGION | GOBP_REGULATION_OF_PROTEIN_LOCALIZATION_TO_CHROMOSOME_TELOMERIC_REGION | 14 | 0.700106949 | 1.718512328 | 0.020838295 | 0.152492519 | 0.129257003 |
| GOBP_MATURATION_OF_5_8S_RRNA_FROM_TRICISTRONIC_RRNA_TRANSCRIPT_SSU_RRNA_5_8S_RRNA_LSU_RRNA | GOBP_MATURATION_OF_5_8S_RRNA_FROM_TRICISTRONIC_RRNA_TRANSCRIPT_SSU_RRNA_5_8S_RRNA_LSU_RRNA | 24 | 0.592338468 | 1.619470792 | 0.020862489 | 0.152492519 | 0.129257003 |
| GOCC_BRUSH_BORDER_MEMBRANE | GOCC_BRUSH_BORDER_MEMBRANE | 44 | -0.514070966 | -1.561724521 | 0.020945017 | 0.152536814 | 0.129294549 |
| GOBP_REGULATION_OF_TRANSMEMBRANE_RECEPTOR_PROTEIN_SERINE_THREONINE_KINASE_SIGNALING_PATHWAY | GOBP_REGULATION_OF_TRANSMEMBRANE_RECEPTOR_PROTEIN_SERINE_THREONINE_KINASE_SIGNALING_PATHWAY | 197 | 0.328687031 | 1.325822011 | 0.020951429 | 0.152536814 | 0.129294549 |
| GOCC_SM_LIKE_PROTEIN_FAMILY_COMPLEX | GOCC_SM_LIKE_PROTEIN_FAMILY_COMPLEX | 95 | -0.422873023 | -1.427661975 | 0.020999491 | 0.152536814 | 0.129294549 |
| GOCC_HETEROCHROMATIN | GOCC_HETEROCHROMATIN | 68 | 0.436956297 | 1.505205678 | 0.021013522 | 0.152536814 | 0.129294549 |
| GOBP_MESODERM_MORPHOGENESIS | GOBP_MESODERM_MORPHOGENESIS | 54 | 0.470533031 | 1.565558715 | 0.02106332 | 0.152536814 | 0.129294549 |
| GOBP_MATURATION_OF_SSU_RRNA_FROM_TRICISTRONIC_RRNA_TRANSCRIPT_SSU_RRNA_5_8S_RRNA_LSU_RRNA | GOBP_MATURATION_OF_SSU_RRNA_FROM_TRICISTRONIC_RRNA_TRANSCRIPT_SSU_RRNA_5_8S_RRNA_LSU_RRNA | 36 | 0.537657482 | 1.620238244 | 0.021068905 | 0.152536814 | 0.129294549 |
| GOBP_UTERUS_DEVELOPMENT | GOBP_UTERUS_DEVELOPMENT | 13 | 0.672739909 | 1.640788783 | 0.021098586 | 0.152536814 | 0.129294549 |
| GOBP_REGULATION_OF_T_CELL_CHEMOTAXIS | GOBP_REGULATION_OF_T_CELL_CHEMOTAXIS | 15 | 0.662378757 | 1.650721774 | 0.0211403 | 0.152536814 | 0.129294549 |
| GOBP_MYOBLAST_MIGRATION | GOBP_MYOBLAST_MIGRATION | 12 | 0.717873063 | 1.71098415 | 0.02115149 | 0.152536814 | 0.129294549 |
| GOBP_MONOSACCHARIDE_BIOSYNTHETIC_PROCESS | GOBP_MONOSACCHARIDE_BIOSYNTHETIC_PROCESS | 83 | 0.424246913 | 1.515797205 | 0.021158915 | 0.152536814 | 0.129294549 |
| GOMF_TRANSLATION_REGULATOR_ACTIVITY_NUCLEIC_ACID_BINDING | GOMF_TRANSLATION_REGULATOR_ACTIVITY_NUCLEIC_ACID_BINDING | 96 | 0.387872423 | 1.430282505 | 0.021158915 | 0.152536814 | 0.129294549 |
| GOBP_NEGATIVE_REGULATION_OF_WOUND_HEALING | GOBP_NEGATIVE_REGULATION_OF_WOUND_HEALING | 60 | 0.453984543 | 1.525208566 | 0.021163163 | 0.152536814 | 0.129294549 |
| GOBP_RESPIRATORY_ELECTRON_TRANSPORT_CHAIN | GOBP_RESPIRATORY_ELECTRON_TRANSPORT_CHAIN | 112 | 0.384276175 | 1.439837604 | 0.021216642 | 0.152741671 | 0.129468192 |
| GOBP_WOUND_HEALING | GOBP_WOUND_HEALING | 439 | 0.276413071 | 1.222486444 | 0.021240754 | 0.152741671 | 0.129468192 |
| GOBP_RESPONSE_TO_GONADOTROPIN | GOBP_RESPONSE_TO_GONADOTROPIN | 17 | 0.651468033 | 1.665057258 | 0.021346546 | 0.153147914 | 0.129812534 |
| GOBP_TETRAHYDROFOLATE_METABOLIC_PROCESS | GOBP_TETRAHYDROFOLATE_METABOLIC_PROCESS | 17 | 0.651543824 | 1.665250971 | 0.021346546 | 0.153147914 | 0.129812534 |
| GOBP_REGULATION_OF_STEM_CELL_DIFFERENTIATION | GOBP_REGULATION_OF_STEM_CELL_DIFFERENTIATION | 97 | 0.395454525 | 1.461748071 | 0.021375928 | 0.153181823 | 0.129841276 |
| GOBP_SOMATIC_DIVERSIFICATION_OF_IMMUNE_RECEPTORS_VIA_SOMATIC_MUTATION | GOBP_SOMATIC_DIVERSIFICATION_OF_IMMUNE_RECEPTORS_VIA_SOMATIC_MUTATION | 14 | 0.696610869 | 1.7099307 | 0.021550699 | 0.154256326 | 0.130752056 |
| GOBP_REGULATION_OF_NERVOUS_SYSTEM_PROCESS | GOBP_REGULATION_OF_NERVOUS_SYSTEM_PROCESS | 86 | 0.397253913 | 1.434167139 | 0.021683519 | 0.155028425 | 0.13140651 |
| GOBP_CHEMICAL_HOMEOSTASIS_WITHIN_A_TISSUE | GOBP_CHEMICAL_HOMEOSTASIS_WITHIN_A_TISSUE | 13 | -0.690989349 | -1.603637206 | 0.02180142 | 0.155692207 | 0.13196915 |
| GOMF_RECEPTOR_INHIBITOR_ACTIVITY | GOMF_RECEPTOR_INHIBITOR_ACTIVITY | 23 | 0.57597826 | 1.56526634 | 0.021945101 | 0.156538358 | 0.132686372 |
| GOBP_REGULATION_OF_MRNA_CATABOLIC_PROCESS | GOBP_REGULATION_OF_MRNA_CATABOLIC_PROCESS | 190 | 0.334521599 | 1.35046388 | 0.022021746 | 0.15690494 | 0.132997097 |
| GOBP_CELLULAR_RESPONSE_TO_ZINC_ION | GOBP_CELLULAR_RESPONSE_TO_ZINC_ION | 17 | 0.649277935 | 1.659459687 | 0.022076325 | 0.157078402 | 0.133144128 |
| GOBP_ER_NUCLEUS_SIGNALING_PATHWAY | GOBP_ER_NUCLEUS_SIGNALING_PATHWAY | 52 | 0.470437793 | 1.533477708 | 0.022096656 | 0.157078402 | 0.133144128 |
| GOBP_DRUG_METABOLIC_PROCESS | GOBP_DRUG_METABOLIC_PROCESS | 25 | -0.588024995 | -1.582667225 | 0.022268837 | 0.158121467 | 0.13402826 |
| GOBP_REGULATION_OF_DNA_TEMPLATED_TRANSCRIPTION_IN_RESPONSE_TO_STRESS | GOBP_REGULATION_OF_DNA_TEMPLATED_TRANSCRIPTION_IN_RESPONSE_TO_STRESS | 109 | 0.382603547 | 1.440605758 | 0.022307679 | 0.158216449 | 0.134108769 |
| GOBP_CELLULAR_RESPONSE_TO_MOLECULE_OF_BACTERIAL_ORIGIN | GOBP_CELLULAR_RESPONSE_TO_MOLECULE_OF_BACTERIAL_ORIGIN | 171 | 0.340690844 | 1.364304852 | 0.02235579 | 0.158376883 | 0.134244758 |
| GOBP_RESPONSE_TO_REACTIVE_OXYGEN_SPECIES | GOBP_RESPONSE_TO_REACTIVE_OXYGEN_SPECIES | 196 | 0.328087196 | 1.325089507 | 0.022434564 | 0.158753924 | 0.134564348 |
| GOBP_DRUG_CATABOLIC_PROCESS | GOBP_DRUG_CATABOLIC_PROCESS | 11 | -0.723374862 | -1.608001393 | 0.022467066 | 0.158803053 | 0.134605991 |
| GOMF_PHOSPHATIDYLGLYCEROL_BINDING | GOMF_PHOSPHATIDYLGLYCEROL_BINDING | 13 | 0.668498974 | 1.630445292 | 0.022493503 | 0.158809244 | 0.13461124 |
| GOBP_CELLULAR_RESPONSE_TO_TOXIC_SUBSTANCE | GOBP_CELLULAR_RESPONSE_TO_TOXIC_SUBSTANCE | 94 | 0.404846541 | 1.492996863 | 0.022528811 | 0.158877983 | 0.134669504 |
| GOBP_TRANSMEMBRANE_RECEPTOR_PROTEIN_SERINE_THREONINE_KINASE_SIGNALING_PATHWAY | GOBP_TRANSMEMBRANE_RECEPTOR_PROTEIN_SERINE_THREONINE_KINASE_SIGNALING_PATHWAY | 287 | 0.307229274 | 1.295320171 | 0.022596698 | 0.159176062 | 0.134922164 |
| GOBP_NEGATIVE_REGULATION_OF_NEURON_PROJECTION_DEVELOPMENT | GOBP_NEGATIVE_REGULATION_OF_NEURON_PROJECTION_DEVELOPMENT | 113 | 0.374169025 | 1.404801026 | 0.022731242 | 0.15951757 | 0.135211637 |
| GOBP_T_CELL_CHEMOTAXIS | GOBP_T_CELL_CHEMOTAXIS | 24 | 0.589230016 | 1.610972191 | 0.022741943 | 0.15951757 | 0.135211637 |
| GOBP_AUTONOMIC_NERVOUS_SYSTEM_DEVELOPMENT | GOBP_AUTONOMIC_NERVOUS_SYSTEM_DEVELOPMENT | 31 | 0.524224406 | 1.534754568 | 0.022799228 | 0.15951757 | 0.135211637 |
| GOBP_REGULATION_OF_SISTER_CHROMATID_COHESION | GOBP_REGULATION_OF_SISTER_CHROMATID_COHESION | 22 | 0.575668513 | 1.55751999 | 0.022799228 | 0.15951757 | 0.135211637 |
| GOBP_REGULATION_OF_TRANSCRIPTION_BY_RNA_POLYMERASE_I | GOBP_REGULATION_OF_TRANSCRIPTION_BY_RNA_POLYMERASE_I | 31 | 0.525628245 | 1.538864543 | 0.022799228 | 0.15951757 | 0.135211637 |
| GOCC_CYTOCHROME_COMPLEX | GOCC_CYTOCHROME_COMPLEX | 31 | 0.525925812 | 1.539735718 | 0.022799228 | 0.15951757 | 0.135211637 |
| GOBP_CORTICOSTEROID_HORMONE_SECRETION | GOBP_CORTICOSTEROID_HORMONE_SECRETION | 13 | -0.687959871 | -1.596606443 | 0.022845787 | 0.159663525 | 0.135335352 |
| GOBP_NOSE_DEVELOPMENT | GOBP_NOSE_DEVELOPMENT | 15 | 0.65735464 | 1.638201113 | 0.022947121 | 0.160191528 | 0.135782903 |
| GOCC_U2_TYPE_SPLICEOSOMAL_COMPLEX | GOCC_U2_TYPE_SPLICEOSOMAL_COMPLEX | 92 | 0.39504778 | 1.44567839 | 0.023169491 | 0.161290903 | 0.136714764 |
| GOBP_NUCLEOSIDE_CATABOLIC_PROCESS | GOBP_NUCLEOSIDE_CATABOLIC_PROCESS | 29 | 0.565427334 | 1.628616665 | 0.023176066 | 0.161290903 | 0.136714764 |
| GOBP_EAR_MORPHOGENESIS | GOBP_EAR_MORPHOGENESIS | 79 | 0.426726517 | 1.527512907 | 0.023193493 | 0.161290903 | 0.136714764 |
| GOBP_EPIBOLY | GOBP_EPIBOLY | 33 | 0.544861108 | 1.629954124 | 0.023208445 | 0.161290903 | 0.136714764 |
| GOBP_SODIUM_ION_HOMEOSTASIS | GOBP_SODIUM_ION_HOMEOSTASIS | 35 | -0.542779176 | -1.570758628 | 0.023269514 | 0.161418071 | 0.136822556 |
| GOBP_RESPONSE_TO_STEROID_HORMONE | GOBP_RESPONSE_TO_STEROID_HORMONE | 263 | 0.307702504 | 1.292402906 | 0.023278705 | 0.161418071 | 0.136822556 |
| GOBP_MRNA_TRANSPORT | GOBP_MRNA_TRANSPORT | 140 | 0.367288107 | 1.424713793 | 0.02357538 | 0.163261739 | 0.138385301 |
| GOBP_RESPONSE_TO_XENOBIOTIC_STIMULUS | GOBP_RESPONSE_TO_XENOBIOTIC_STIMULUS | 89 | -0.441340345 | -1.482244279 | 0.023597142 | 0.163261739 | 0.138385301 |
| GOBP_MATURATION_OF_LSU_RRNA_FROM_TRICISTRONIC_RRNA_TRANSCRIPT_SSU_RRNA_5_8S_RRNA_LSU_RRNA | GOBP_MATURATION_OF_LSU_RRNA_FROM_TRICISTRONIC_RRNA_TRANSCRIPT_SSU_RRNA_5_8S_RRNA_LSU_RRNA | 15 | 0.655110076 | 1.632607409 | 0.02366985 | 0.163582622 | 0.13865729 |
| GOBP_DEVELOPMENTAL_MATURATION | GOBP_DEVELOPMENTAL_MATURATION | 200 | 0.336642107 | 1.358478799 | 0.023708276 | 0.163666132 | 0.138728076 |
| GOCC_NEURON_PROJECTION_TERMINUS | GOCC_NEURON_PROJECTION_TERMINUS | 82 | 0.413610369 | 1.487623964 | 0.023744031 | 0.163731035 | 0.138783089 |
| GOBP_HEMATOPOIETIC_STEM_CELL_DIFFERENTIATION | GOBP_HEMATOPOIETIC_STEM_CELL_DIFFERENTIATION | 83 | 0.421449504 | 1.505802307 | 0.023771621 | 0.163739559 | 0.138790315 |
| GOBP_APOPTOTIC_CELL_CLEARANCE | GOBP_APOPTOTIC_CELL_CLEARANCE | 44 | -0.509910991 | -1.549086705 | 0.023817448 | 0.163873534 | 0.138903876 |
| GOBP_PROTEIN_POLYUBIQUITINATION | GOBP_PROTEIN_POLYUBIQUITINATION | 310 | 0.290515271 | 1.244016256 | 0.023849952 | 0.163915655 | 0.138939578 |
| GOBP_ACUTE_PHASE_RESPONSE | GOBP_ACUTE_PHASE_RESPONSE | 32 | 0.54640826 | 1.619577094 | 0.023990172 | 0.164697166 | 0.13960201 |
| GOBP_NEGATIVE_REGULATION_OF_LIPASE_ACTIVITY | GOBP_NEGATIVE_REGULATION_OF_LIPASE_ACTIVITY | 14 | 0.690729256 | 1.695493444 | 0.024044114 | 0.164703507 | 0.139607384 |
| GOBP_NUCLEAR_PORE_ORGANIZATION | GOBP_NUCLEAR_PORE_ORGANIZATION | 14 | 0.691996233 | 1.69860342 | 0.024044114 | 0.164703507 | 0.139607384 |
| GOBP_RESPONSE_TO_VIRUS | GOBP_RESPONSE_TO_VIRUS | 304 | 0.300731851 | 1.283160508 | 0.024161728 | 0.165296844 | 0.140110314 |
| GOBP_CYTOKINE_PRODUCTION_INVOLVED_IN_IMMUNE_RESPONSE | GOBP_CYTOKINE_PRODUCTION_INVOLVED_IN_IMMUNE_RESPONSE | 84 | 0.400440319 | 1.433401053 | 0.024207786 | 0.165296844 | 0.140110314 |
| GOBP_INNER_CELL_MASS_CELL_PROLIFERATION | GOBP_INNER_CELL_MASS_CELL_PROLIFERATION | 13 | 0.667049257 | 1.626909485 | 0.024237152 | 0.165296844 | 0.140110314 |
| GOBP_MUSCLE_CELL_CELLULAR_HOMEOSTASIS | GOBP_MUSCLE_CELL_CELLULAR_HOMEOSTASIS | 18 | 0.619482881 | 1.630420556 | 0.024237152 | 0.165296844 | 0.140110314 |
| GOBP_INNERVATION | GOBP_INNERVATION | 16 | 0.641768553 | 1.624451941 | 0.024517065 | 0.167022503 | 0.141573031 |
| GOBP_ADRENAL_GLAND_DEVELOPMENT | GOBP_ADRENAL_GLAND_DEVELOPMENT | 18 | 0.618611494 | 1.628127148 | 0.024585882 | 0.167154853 | 0.141685215 |
| GOBP_POSITIVE_REGULATION_OF_LEUKOCYTE_CHEMOTAXIS | GOBP_POSITIVE_REGULATION_OF_LEUKOCYTE_CHEMOTAXIS | 81 | 0.400001667 | 1.439304515 | 0.0245903 | 0.167154853 | 0.141685215 |
| GOBP_NUCLEOSIDE_METABOLIC_PROCESS | GOBP_NUCLEOSIDE_METABOLIC_PROCESS | 94 | 0.402447307 | 1.484148947 | 0.024716009 | 0.16782575 | 0.142253886 |
| GOBP_REGULATION_OF_TELOMERE_MAINTENANCE_VIA_TELOMERE_LENGTHENING | GOBP_REGULATION_OF_TELOMERE_MAINTENANCE_VIA_TELOMERE_LENGTHENING | 56 | 0.464274119 | 1.544179887 | 0.024798064 | 0.167877259 | 0.142297547 |
| GOBP_NEGATIVE_REGULATION_OF_MULTICELLULAR_ORGANISM_GROWTH | GOBP_NEGATIVE_REGULATION_OF_MULTICELLULAR_ORGANISM_GROWTH | 10 | -0.71728311 | -1.568542268 | 0.024804655 | 0.167877259 | 0.142297547 |
| GOMF_ACYL_COA_DEHYDROGENASE_ACTIVITY | GOMF_ACYL_COA_DEHYDROGENASE_ACTIVITY | 10 | -0.716888558 | -1.567679468 | 0.024804655 | 0.167877259 | 0.142297547 |
| GOBP_POSITIVE_REGULATION_OF_MUSCLE_CONTRACTION | GOBP_POSITIVE_REGULATION_OF_MUSCLE_CONTRACTION | 25 | 0.588271185 | 1.619758832 | 0.024871365 | 0.168145583 | 0.142524986 |
| GOBP_POSITIVE_REGULATION_OF_STEM_CELL_PROLIFERATION | GOBP_POSITIVE_REGULATION_OF_STEM_CELL_PROLIFERATION | 18 | 0.616482387 | 1.622523539 | 0.024934612 | 0.168207106 | 0.142577135 |
| GOCC_HIGH_DENSITY_LIPOPROTEIN_PARTICLE | GOCC_HIGH_DENSITY_LIPOPROTEIN_PARTICLE | 18 | 0.616757798 | 1.623248395 | 0.024934612 | 0.168207106 | 0.142577135 |
| GOBP_TRANSLESION_SYNTHESIS | GOBP_TRANSLESION_SYNTHESIS | 41 | 0.474340228 | 1.465313522 | 0.024999256 | 0.168306825 | 0.142661659 |
| GOBP_CELLULAR_RESPONSE_TO_VIRUS | GOBP_CELLULAR_RESPONSE_TO_VIRUS | 58 | 0.465198586 | 1.55389223 | 0.025003573 | 0.168306825 | 0.142661659 |
| GOBP_REGULATION_OF_CENTROSOME_CYCLE | GOBP_REGULATION_OF_CENTROSOME_CYCLE | 44 | 0.490838399 | 1.535899569 | 0.025199225 | 0.168950305 | 0.143207092 |
| GOMF_MONOCARBOXYLIC_ACID_BINDING | GOMF_MONOCARBOXYLIC_ACID_BINDING | 55 | 0.475473778 | 1.581818681 | 0.025203512 | 0.168950305 | 0.143207092 |
| GOMF_RIBOSOME_BINDING | GOMF_RIBOSOME_BINDING | 55 | 0.475247578 | 1.581066153 | 0.025203512 | 0.168950305 | 0.143207092 |
| GOBP_PROTEIN_SUMOYLATION | GOBP_PROTEIN_SUMOYLATION | 75 | 0.422417335 | 1.501339828 | 0.02520794 | 0.168950305 | 0.143207092 |
| GOBP_HOMOLOGOUS_CHROMOSOME_PAIRING_AT_MEIOSIS | GOBP_HOMOLOGOUS_CHROMOSOME_PAIRING_AT_MEIOSIS | 26 | 0.562491774 | 1.57182596 | 0.02524632 | 0.169025199 | 0.143270574 |
| GOBP_RESPONSE_TO_IRON_ION | GOBP_RESPONSE_TO_IRON_ION | 27 | 0.562084723 | 1.583795542 | 0.025366666 | 0.169648111 | 0.143798572 |
| GOMF_LIPID_PHOSPHATASE_ACTIVITY | GOMF_LIPID_PHOSPHATASE_ACTIVITY | 13 | -0.684669378 | -1.588969917 | 0.025456707 | 0.170067226 | 0.144153826 |
| GOBP_PEPTIDYL_SERINE_MODIFICATION | GOBP_PEPTIDYL_SERINE_MODIFICATION | 280 | 0.311167477 | 1.323382367 | 0.02561392 | 0.170732608 | 0.144717822 |
| GOBP_RHYTHMIC_PROCESS | GOBP_RHYTHMIC_PROCESS | 238 | 0.315080673 | 1.312712775 | 0.025636165 | 0.170732608 | 0.144717822 |
| GOCC_ENDOSOME_LUMEN | GOCC_ENDOSOME_LUMEN | 24 | -0.584241412 | -1.564786535 | 0.025638745 | 0.170732608 | 0.144717822 |
| GOBP_REGULATION_OF_HEMATOPOIETIC_STEM_CELL_DIFFERENTIATION | GOBP_REGULATION_OF_HEMATOPOIETIC_STEM_CELL_DIFFERENTIATION | 70 | 0.455783641 | 1.58329491 | 0.025772824 | 0.171156414 | 0.145077052 |
| GOBP_ANATOMICAL_STRUCTURE_ARRANGEMENT | GOBP_ANATOMICAL_STRUCTURE_ARRANGEMENT | 11 | 0.745805708 | 1.732804482 | 0.02581258 | 0.171156414 | 0.145077052 |
| GOBP_MEIOTIC_SPINDLE_ORGANIZATION | GOBP_MEIOTIC_SPINDLE_ORGANIZATION | 10 | 0.742307733 | 1.655487082 | 0.02581258 | 0.171156414 | 0.145077052 |
| GOBP_REGULATION_OF_VITAMIN_METABOLIC_PROCESS | GOBP_REGULATION_OF_VITAMIN_METABOLIC_PROCESS | 10 | 0.741667047 | 1.654058231 | 0.02581258 | 0.171156414 | 0.145077052 |
| GOBP_MORPHOGENESIS_OF_A_POLARIZED_EPITHELIUM | GOBP_MORPHOGENESIS_OF_A_POLARIZED_EPITHELIUM | 134 | 0.364563496 | 1.401158464 | 0.025884654 | 0.171451339 | 0.145327039 |
| GOCC_FIBRILLAR_COLLAGEN_TRIMER | GOCC_FIBRILLAR_COLLAGEN_TRIMER | 12 | 0.706855949 | 1.684725875 | 0.025966367 | 0.171451881 | 0.145327499 |
| GOBP_LEUKOCYTE_AGGREGATION | GOBP_LEUKOCYTE_AGGREGATION | 13 | 0.664507077 | 1.620709197 | 0.025980803 | 0.171451881 | 0.145327499 |
| GOBP_WOUND_HEALING_SPREADING_OF_EPIDERMAL_CELLS | GOBP_WOUND_HEALING_SPREADING_OF_EPIDERMAL_CELLS | 18 | 0.615109372 | 1.618909892 | 0.025980803 | 0.171451881 | 0.145327499 |
| GOBP_PROTEIN_MATURATION | GOBP_PROTEIN_MATURATION | 250 | 0.306010127 | 1.278612626 | 0.026025625 | 0.171451881 | 0.145327499 |
| GOBP_NADH_METABOLIC_PROCESS | GOBP_NADH_METABOLIC_PROCESS | 38 | 0.495467139 | 1.498410709 | 0.026044301 | 0.171451881 | 0.145327499 |
| GOCC_CLATHRIN_COATED_ENDOCYTIC_VESICLE_MEMBRANE | GOCC_CLATHRIN_COATED_ENDOCYTIC_VESICLE_MEMBRANE | 32 | -0.553301815 | -1.572524673 | 0.02605031 | 0.171451881 | 0.145327499 |
| GOBP_SOMATIC_DIVERSIFICATION_OF_IMMUNOGLOBULINS | GOBP_SOMATIC_DIVERSIFICATION_OF_IMMUNOGLOBULINS | 59 | 0.441166235 | 1.48210344 | 0.026222046 | 0.172399546 | 0.146130766 |
| GOBP_MAINTENANCE_OF_LOCATION_IN_CELL | GOBP_MAINTENANCE_OF_LOCATION_IN_CELL | 176 | 0.347612349 | 1.396502907 | 0.026296428 | 0.172614379 | 0.146312865 |
| GOMF_HYALURONIC_ACID_BINDING | GOMF_HYALURONIC_ACID_BINDING | 12 | 0.706276374 | 1.683344512 | 0.026310288 | 0.172614379 | 0.146312865 |
| GOMF_ORGANIC_ACID_BINDING | GOMF_ORGANIC_ACID_BINDING | 83 | 0.420085084 | 1.500927352 | 0.026388611 | 0.172945609 | 0.146593625 |
| GOBP_CAMERA_TYPE_EYE_DEVELOPMENT | GOBP_CAMERA_TYPE_EYE_DEVELOPMENT | 219 | 0.321464965 | 1.312246239 | 0.026416956 | 0.172948941 | 0.146596449 |
| GOBP_MITOTIC_G2_DNA_DAMAGE_CHECKPOINT | GOBP_MITOTIC_G2_DNA_DAMAGE_CHECKPOINT | 23 | 0.567631791 | 1.542584152 | 0.026524842 | 0.173472468 | 0.147040206 |
| GOMF_LIGASE_ACTIVITY_FORMING_CARBON_NITROGEN_BONDS | GOMF_LIGASE_ACTIVITY_FORMING_CARBON_NITROGEN_BONDS | 42 | 0.506813744 | 1.568458507 | 0.026645192 | 0.173952306 | 0.14744693 |
| GOBP_HIGH_DENSITY_LIPOPROTEIN_PARTICLE_ASSEMBLY | GOBP_HIGH_DENSITY_LIPOPROTEIN_PARTICLE_ASSEMBLY | 12 | 0.70541195 | 1.681284238 | 0.026654208 | 0.173952306 | 0.14744693 |
| GOBP_RESPONSE_TO_COPPER_ION | GOBP_RESPONSE_TO_COPPER_ION | 35 | 0.544111641 | 1.63954061 | 0.026798294 | 0.174301297 | 0.147742745 |
| GOCC_POTASSIUM_CHANNEL_COMPLEX | GOCC_POTASSIUM_CHANNEL_COMPLEX | 45 | 0.486936963 | 1.529292798 | 0.026816504 | 0.174301297 | 0.147742745 |
| GOMF_FOUR_WAY_JUNCTION_DNA_BINDING | GOMF_FOUR_WAY_JUNCTION_DNA_BINDING | 17 | 0.63602468 | 1.625586302 | 0.0268199 | 0.174301297 | 0.147742745 |
| GOMF_RIBOSOMAL_SMALL_SUBUNIT_BINDING | GOMF_RIBOSOMAL_SMALL_SUBUNIT_BINDING | 17 | 0.63526428 | 1.62364283 | 0.0268199 | 0.174301297 | 0.147742745 |
| GOCC_U2_TYPE_CATALYTIC_STEP_2_SPLICEOSOME | GOCC_U2_TYPE_CATALYTIC_STEP_2_SPLICEOSOME | 30 | 0.535254667 | 1.55162739 | 0.026876755 | 0.174488276 | 0.147901233 |
| GOBP_RNA_CATABOLIC_PROCESS | GOBP_RNA_CATABOLIC_PROCESS | 388 | 0.286352274 | 1.248451902 | 0.026927365 | 0.174634363 | 0.148025061 |
| GOBP_EOSINOPHIL_CHEMOTAXIS | GOBP_EOSINOPHIL_CHEMOTAXIS | 16 | 0.638025176 | 1.614976662 | 0.027022416 | 0.175068059 | 0.148392674 |
| GOBP_REGULATION_OF_TELOMERASE_ACTIVITY | GOBP_REGULATION_OF_TELOMERASE_ACTIVITY | 45 | 0.486743563 | 1.528685396 | 0.027219752 | 0.176162831 | 0.149320634 |
| GOBP_HOMOPHILIC_CELL_ADHESION_VIA_PLASMA_MEMBRANE_ADHESION_MOLECULES | GOBP_HOMOPHILIC_CELL_ADHESION_VIA_PLASMA_MEMBRANE_ADHESION_MOLECULES | 96 | 0.381947624 | 1.408434764 | 0.027697108 | 0.178900894 | 0.151641494 |
| GOBP_SENSORY_SYSTEM_DEVELOPMENT | GOBP_SENSORY_SYSTEM_DEVELOPMENT | 265 | 0.310248144 | 1.301932953 | 0.027700412 | 0.178900894 | 0.151641494 |
| GOBP_REGULATION_OF_ATP_BIOSYNTHETIC_PROCESS | GOBP_REGULATION_OF_ATP_BIOSYNTHETIC_PROCESS | 19 | 0.608932103 | 1.608596233 | 0.027738231 | 0.178959116 | 0.151690845 |
| GOBP_NEGATIVE_REGULATION_OF_FATTY_ACID_OXIDATION | GOBP_NEGATIVE_REGULATION_OF_FATTY_ACID_OXIDATION | 12 | -0.692692728 | -1.576801263 | 0.02784213 | 0.179443107 | 0.152101089 |
| GOBP_ARACHIDONIC_ACID_METABOLIC_PROCESS | GOBP_ARACHIDONIC_ACID_METABOLIC_PROCESS | 40 | -0.521283349 | -1.55299401 | 0.027896934 | 0.179610001 | 0.152242553 |
| GOCC_ORGANELLE_ENVELOPE_LUMEN | GOCC_ORGANELLE_ENVELOPE_LUMEN | 88 | 0.41433725 | 1.498988221 | 0.028190231 | 0.181125029 | 0.153526734 |
| GOBP_POSITIVE_REGULATION_OF_TELOMERASE_ACTIVITY | GOBP_POSITIVE_REGULATION_OF_TELOMERASE_ACTIVITY | 33 | 0.537731654 | 1.608626335 | 0.02819528 | 0.181125029 | 0.153526734 |
| GOBP_REGULATION_OF_CALCIUM_ION_TRANSMEMBRANE_TRANSPORTER_ACTIVITY | GOBP_REGULATION_OF_CALCIUM_ION_TRANSMEMBRANE_TRANSPORTER_ACTIVITY | 59 | -0.455940899 | -1.442292372 | 0.028219705 | 0.181125029 | 0.153526734 |
| GOBP_CLEAVAGE_INVOLVED_IN_RRNA_PROCESSING | GOBP_CLEAVAGE_INVOLVED_IN_RRNA_PROCESSING | 27 | 0.558432275 | 1.573503977 | 0.028329146 | 0.181639821 | 0.153963086 |
| GOBP_REGULATION_OF_SPINDLE_ORGANIZATION | GOBP_REGULATION_OF_SPINDLE_ORGANIZATION | 37 | 0.499044655 | 1.515061498 | 0.028433072 | 0.181690638 | 0.15400616 |
| GOBP_REGULATION_OF_G_PROTEIN_COUPLED_RECEPTOR_SIGNALING_PATHWAY | GOBP_REGULATION_OF_G_PROTEIN_COUPLED_RECEPTOR_SIGNALING_PATHWAY | 104 | 0.376347668 | 1.410006789 | 0.028443437 | 0.181690638 | 0.15400616 |
| GOMF_TRANSLATION_INITIATION_FACTOR_BINDING | GOMF_TRANSLATION_INITIATION_FACTOR_BINDING | 29 | 0.56153118 | 1.61739446 | 0.028451813 | 0.181690638 | 0.15400616 |
| GOBP_EOSINOPHIL_MIGRATION | GOBP_EOSINOPHIL_MIGRATION | 19 | 0.606819675 | 1.603015899 | 0.028454046 | 0.181690638 | 0.15400616 |
| GOMF_TRIGLYCERIDE_LIPASE_ACTIVITY | GOMF_TRIGLYCERIDE_LIPASE_ACTIVITY | 18 | -0.61930567 | -1.537612276 | 0.028600105 | 0.182403834 | 0.154610686 |
| GOBP_CENTROSOME_DUPLICATION | GOBP_CENTROSOME_DUPLICATION | 66 | 0.436387023 | 1.496784694 | 0.02862607 | 0.182403834 | 0.154610686 |
| GOBP_CARBOHYDRATE_CATABOLIC_PROCESS | GOBP_CARBOHYDRATE_CATABOLIC_PROCESS | 165 | 0.350107605 | 1.395527129 | 0.028653813 | 0.182403834 | 0.154610686 |
| GOMF_PROTEIN_DISULFIDE_OXIDOREDUCTASE_ACTIVITY | GOMF_PROTEIN_DISULFIDE_OXIDOREDUCTASE_ACTIVITY | 18 | 0.605079705 | 1.592512755 | 0.028770648 | 0.182960118 | 0.155082208 |
| GOMF_INSULIN_LIKE_GROWTH_FACTOR_BINDING | GOMF_INSULIN_LIKE_GROWTH_FACTOR_BINDING | 28 | 0.544041219 | 1.550337474 | 0.02884224 | 0.183200241 | 0.155285743 |
| GOMF_PROTEIN_C_TERMINUS_BINDING | GOMF_PROTEIN_C_TERMINUS_BINDING | 171 | 0.334398883 | 1.339108538 | 0.028867381 | 0.183200241 | 0.155285743 |
| GOBP_OOCYTE_DIFFERENTIATION | GOBP_OOCYTE_DIFFERENTIATION | 34 | 0.511329092 | 1.524574809 | 0.029025428 | 0.183967357 | 0.155935972 |
| GOCC_FICOLIN_1_RICH_GRANULE | GOCC_FICOLIN_1_RICH_GRANULE | 172 | 0.329389057 | 1.316575457 | 0.029062431 | 0.183967357 | 0.155935972 |
| GOBP_NEGATIVE_REGULATION_OF_CELL_ADHESION | GOBP_NEGATIVE_REGULATION_OF_CELL_ADHESION | 245 | 0.316963013 | 1.319963676 | 0.029105679 | 0.183967357 | 0.155935972 |
| GOBP_EPHRIN_RECEPTOR_SIGNALING_PATHWAY | GOBP_EPHRIN_RECEPTOR_SIGNALING_PATHWAY | 77 | 0.417442374 | 1.486411215 | 0.029136308 | 0.183967357 | 0.155935972 |
| GOBP_SMOOTH_MUSCLE_CELL_MIGRATION | GOBP_SMOOTH_MUSCLE_CELL_MIGRATION | 63 | 0.435326176 | 1.484346055 | 0.029136308 | 0.183967357 | 0.155935972 |
| GOCC_RESPIRATORY_CHAIN_COMPLEX_IV | GOCC_RESPIRATORY_CHAIN_COMPLEX_IV | 19 | 0.60638091 | 1.601856828 | 0.029169862 | 0.183992237 | 0.155957062 |
| GOMF_SOLUTE_SODIUM_SYMPORTER_ACTIVITY | GOMF_SOLUTE_SODIUM_SYMPORTER_ACTIVITY | 37 | -0.521703342 | -1.523628676 | 0.029329989 | 0.184814624 | 0.15665414 |
| GOBP_DEOXYRIBONUCLEOSIDE_MONOPHOSPHATE_METABOLIC_PROCESS | GOBP_DEOXYRIBONUCLEOSIDE_MONOPHOSPHATE_METABOLIC_PROCESS | 14 | 0.679266735 | 1.667357052 | 0.029387162 | 0.184933274 | 0.156754711 |
| GOBP_REGULATION_OF_CHONDROCYTE_DIFFERENTIATION | GOBP_REGULATION_OF_CHONDROCYTE_DIFFERENTIATION | 42 | 0.500415087 | 1.548656306 | 0.029408349 | 0.184933274 | 0.156754711 |
| GOMF_HYDROLASE_ACTIVITY_HYDROLYZING_O_GLYCOSYL_COMPOUNDS | GOMF_HYDROLASE_ACTIVITY_HYDROLYZING_O_GLYCOSYL_COMPOUNDS | 68 | -0.444480679 | -1.432109503 | 0.02955734 | 0.185682258 | 0.157389571 |
| GOBP_MUSCLE_ATROPHY | GOBP_MUSCLE_ATROPHY | 10 | 0.735906877 | 1.641211959 | 0.029675632 | 0.186112701 | 0.157754427 |
| GOBP_POSITIVE_REGULATION_OF_ESTABLISHMENT_OF_PROTEIN_LOCALIZATION | GOBP_POSITIVE_REGULATION_OF_ESTABLISHMENT_OF_PROTEIN_LOCALIZATION | 296 | 0.300154021 | 1.279604312 | 0.02968577 | 0.186112701 | 0.157754427 |
| GOBP_AMINO_ACID_ACTIVATION | GOBP_AMINO_ACID_ACTIVATION | 49 | 0.482979876 | 1.560348914 | 0.029724657 | 0.186136567 | 0.157774657 |
| GOMF_INTERLEUKIN_1_RECEPTOR_BINDING | GOMF_INTERLEUKIN_1_RECEPTOR_BINDING | 12 | 0.691130556 | 1.647245853 | 0.029749495 | 0.186136567 | 0.157774657 |
| GOBP_EMBRYONIC_HEART_TUBE_MORPHOGENESIS | GOBP_EMBRYONIC_HEART_TUBE_MORPHOGENESIS | 53 | -0.478538834 | -1.497000345 | 0.029878147 | 0.186572079 | 0.158143809 |
| GOBP_EXTRACELLULAR_MATRIX_DISASSEMBLY | GOBP_EXTRACELLULAR_MATRIX_DISASSEMBLY | 67 | 0.443611948 | 1.523542251 | 0.02987916 | 0.186572079 | 0.158143809 |
| GOBP_NEGATIVE_REGULATION_OF_CARTILAGE_DEVELOPMENT | GOBP_NEGATIVE_REGULATION_OF_CARTILAGE_DEVELOPMENT | 23 | 0.562304314 | 1.528106313 | 0.029959658 | 0.186793401 | 0.158331408 |
| GOBP_GLYCOSYLATION | GOBP_GLYCOSYLATION | 230 | 0.309040664 | 1.27787937 | 0.029974734 | 0.186793401 | 0.158331408 |
| GOBP_CELLULAR_RESPONSE_TO_GONADOTROPIN_STIMULUS | GOBP_CELLULAR_RESPONSE_TO_GONADOTROPIN_STIMULUS | 10 | 0.735044429 | 1.639288537 | 0.030026818 | 0.186930484 | 0.158447603 |
| GOMF_HEPARIN_BINDING | GOMF_HEPARIN_BINDING | 134 | 0.359918983 | 1.383307805 | 0.030159073 | 0.187565885 | 0.158986187 |
| GOMF_PROTEIN_FOLDING_CHAPERONE | GOMF_PROTEIN_FOLDING_CHAPERONE | 34 | 0.509040062 | 1.517749854 | 0.03019422 | 0.18759669 | 0.159012298 |
| GOBP_AMINE_TRANSPORT | GOBP_AMINE_TRANSPORT | 53 | 0.462276117 | 1.520179051 | 0.030282493 | 0.18795717 | 0.159317851 |
| GOMF_GUANYL_NUCLEOTIDE_BINDING | GOMF_GUANYL_NUCLEOTIDE_BINDING | 347 | 0.286163637 | 1.238953263 | 0.030378792 | 0.188366701 | 0.159664982 |
| GOBP_ORGANELLE_INHERITANCE | GOBP_ORGANELLE_INHERITANCE | 14 | 0.678205174 | 1.664751298 | 0.030455773 | 0.18865575 | 0.159909988 |
| GOBP_CHROMATIN_ORGANIZATION_INVOLVED_IN_REGULATION_OF_TRANSCRIPTION | GOBP_CHROMATIN_ORGANIZATION_INVOLVED_IN_REGULATION_OF_TRANSCRIPTION | 127 | -0.399912761 | -1.415553479 | 0.030514784 | 0.188775018 | 0.160011083 |
| GOBP_RETROGRADE_AXONAL_TRANSPORT | GOBP_RETROGRADE_AXONAL_TRANSPORT | 15 | 0.642338796 | 1.600779953 | 0.030535795 | 0.188775018 | 0.160011083 |
| GOBP_POSTREPLICATION_REPAIR | GOBP_POSTREPLICATION_REPAIR | 51 | 0.484243581 | 1.577909226 | 0.030601538 | 0.188993394 | 0.160196184 |
| GOCC_NUCLEAR_LAMINA | GOCC_NUCLEAR_LAMINA | 10 | 0.73387968 | 1.636690924 | 0.030729192 | 0.189593318 | 0.160704697 |
| GOBP_CORONARY_VASCULATURE_DEVELOPMENT | GOBP_CORONARY_VASCULATURE_DEVELOPMENT | 37 | -0.519677199 | -1.51771135 | 0.030808778 | 0.189783516 | 0.160865915 |
| GOBP_MRNA_CIS_SPLICING_VIA_SPLICEOSOME | GOBP_MRNA_CIS_SPLICING_VIA_SPLICEOSOME | 28 | -0.555073152 | -1.520513925 | 0.030821112 | 0.189783516 | 0.160865915 |
| GOBP_GLUCOSAMINE_CONTAINING_COMPOUND_METABOLIC_PROCESS | GOBP_GLUCOSAMINE_CONTAINING_COMPOUND_METABOLIC_PROCESS | 25 | -0.572354715 | -1.54049072 | 0.030910122 | 0.190091353 | 0.161126846 |
| GOBP_BEHAVIOR | GOBP_BEHAVIOR | 361 | 0.275059996 | 1.193296697 | 0.030932296 | 0.190091353 | 0.161126846 |
| GOBP_LONG_CHAIN_FATTY_ACID_METABOLIC_PROCESS | GOBP_LONG_CHAIN_FATTY_ACID_METABOLIC_PROCESS | 85 | -0.428834364 | -1.429039731 | 0.031114017 | 0.19072872 | 0.161667097 |
| GOBP_REGULATION_OF_HISTONE_PHOSPHORYLATION | GOBP_REGULATION_OF_HISTONE_PHOSPHORYLATION | 12 | 0.687462874 | 1.638504272 | 0.031125179 | 0.19072872 | 0.161667097 |
| GOBP_CELLULAR_RESPONSE_TO_TOPOLOGICALLY_INCORRECT_PROTEIN | GOBP_CELLULAR_RESPONSE_TO_TOPOLOGICALLY_INCORRECT_PROTEIN | 158 | 0.34962967 | 1.386812341 | 0.031128106 | 0.19072872 | 0.161667097 |
| GOCC_PRERIBOSOME_SMALL_SUBUNIT_PRECURSOR | GOCC_PRERIBOSOME_SMALL_SUBUNIT_PRECURSOR | 15 | 0.640946181 | 1.597309402 | 0.031258527 | 0.191339142 | 0.162184507 |
| GOBP_POSITIVE_REGULATION_OF_HORMONE_SECRETION | GOBP_POSITIVE_REGULATION_OF_HORMONE_SECRETION | 102 | 0.375247833 | 1.397911645 | 0.031297755 | 0.191390699 | 0.162228209 |
| GOBP_PATHWAY_RESTRICTED_SMAD_PROTEIN_PHOSPHORYLATION | GOBP_PATHWAY_RESTRICTED_SMAD_PROTEIN_PHOSPHORYLATION | 52 | 0.449659919 | 1.46574844 | 0.031421627 | 0.191959264 | 0.16271014 |
| GOBP_POSITIVE_REGULATION_OF_TRANSMEMBRANE_RECEPTOR_PROTEIN_SERINE_THREONINE_KINASE_SIGNALING_PATHWAY | GOBP_POSITIVE_REGULATION_OF_TRANSMEMBRANE_RECEPTOR_PROTEIN_SERINE_THREONINE_KINASE_SIGNALING_PATHWAY | 85 | 0.395693153 | 1.421665077 | 0.031461613 | 0.192014739 | 0.162757163 |
| GOBP_CENTROSOME_SEPARATION | GOBP_CENTROSOME_SEPARATION | 14 | 0.676920072 | 1.661596833 | 0.031524385 | 0.192164088 | 0.162883755 |
| GOBP_SIGNAL_TRANSDUCTION_INVOLVED_IN_G2_DNA_DAMAGE_CHECKPOINT | GOBP_SIGNAL_TRANSDUCTION_INVOLVED_IN_G2_DNA_DAMAGE_CHECKPOINT | 13 | 0.656155671 | 1.600340413 | 0.031560496 | 0.192164088 | 0.162883755 |
| GOBP_NUCLEOBASE_CONTAINING_SMALL_MOLECULE_BIOSYNTHETIC_PROCESS | GOBP_NUCLEOBASE_CONTAINING_SMALL_MOLECULE_BIOSYNTHETIC_PROCESS | 104 | 0.373670152 | 1.399975335 | 0.031578872 | 0.192164088 | 0.162883755 |
| GOCC_PHOTORECEPTOR_CONNECTING_CILIUM | GOCC_PHOTORECEPTOR_CONNECTING_CILIUM | 30 | -0.551878015 | -1.546782368 | 0.031612652 | 0.192181414 | 0.162898441 |
| GOBP_CELLULAR_CARBOHYDRATE_METABOLIC_PROCESS | GOBP_CELLULAR_CARBOHYDRATE_METABOLIC_PROCESS | 238 | 0.309600708 | 1.289881734 | 0.031647879 | 0.192207501 | 0.162920553 |
| GOBP_PEPTIDE_CROSS_LINKING | GOBP_PEPTIDE_CROSS_LINKING | 25 | 0.577634033 | 1.590470264 | 0.031706126 | 0.192373201 | 0.163061006 |
| GOCC_COPI_COATED_VESICLE | GOCC_COPI_COATED_VESICLE | 28 | 0.535897201 | 1.527129715 | 0.031819464 | 0.192872518 | 0.163484241 |
| GOBP_CHAPERONE_COFACTOR_DEPENDENT_PROTEIN_REFOLDING | GOBP_CHAPERONE_COFACTOR_DEPENDENT_PROTEIN_REFOLDING | 32 | 0.535132526 | 1.586155345 | 0.031923785 | 0.193316256 | 0.163860366 |
| GOCC_ANCHORED_COMPONENT_OF_EXTERNAL_SIDE_OF_PLASMA_MEMBRANE | GOCC_ANCHORED_COMPONENT_OF_EXTERNAL_SIDE_OF_PLASMA_MEMBRANE | 15 | -0.659420134 | -1.595751128 | 0.031961487 | 0.193356103 | 0.163894141 |
| GOBP_REGULATION_OF_ATP_METABOLIC_PROCESS | GOBP_REGULATION_OF_ATP_METABOLIC_PROCESS | 104 | 0.373470566 | 1.399227574 | 0.032026792 | 0.193380402 | 0.163914737 |
| GOCC_MITOTIC_SPINDLE_POLE | GOCC_MITOTIC_SPINDLE_POLE | 33 | 0.534949298 | 1.600302907 | 0.032031317 | 0.193380402 | 0.163914737 |
| GOBP_CELLULAR_RESPONSE_TO_BIOTIC_STIMULUS | GOBP_CELLULAR_RESPONSE_TO_BIOTIC_STIMULUS | 195 | 0.325293739 | 1.313602341 | 0.032058879 | 0.193380402 | 0.163914737 |
| GOBP_REGULATION_OF_CELLULAR_AMIDE_METABOLIC_PROCESS | GOBP_REGULATION_OF_CELLULAR_AMIDE_METABOLIC_PROCESS | 392 | 0.275817238 | 1.208832752 | 0.032356183 | 0.194838038 | 0.165150272 |
| GOMF_CHOLESTEROL_BINDING | GOMF_CHOLESTEROL_BINDING | 41 | 0.461026477 | 1.424185196 | 0.032363247 | 0.194838038 | 0.165150272 |
| GOBP_PEPTIDYL_LYSINE_MODIFICATION | GOBP_PEPTIDYL_LYSINE_MODIFICATION | 357 | 0.281182312 | 1.220171519 | 0.032484117 | 0.195226494 | 0.165479538 |
| GOBP_REGULATION_OF_PEPTIDE_HORMONE_SECRETION | GOBP_REGULATION_OF_PEPTIDE_HORMONE_SECRETION | 164 | 0.349596958 | 1.394063102 | 0.032490616 | 0.195226494 | 0.165479538 |
| GOMF_CHEMOKINE_ACTIVITY | GOMF_CHEMOKINE_ACTIVITY | 40 | 0.490218305 | 1.508540756 | 0.032548154 | 0.195366829 | 0.16559849 |
| GOBP_PROTEIN_LOCALIZATION_TO_NUCLEOPLASM | GOBP_PROTEIN_LOCALIZATION_TO_NUCLEOPLASM | 14 | 0.672175978 | 1.649951778 | 0.032592997 | 0.195366829 | 0.16559849 |
| GOBP_REGULATION_OF_B_CELL_RECEPTOR_SIGNALING_PATHWAY | GOBP_REGULATION_OF_B_CELL_RECEPTOR_SIGNALING_PATHWAY | 24 | -0.576270375 | -1.543437532 | 0.032608305 | 0.195366829 | 0.16559849 |
| GOBP_MRNA_EXPORT_FROM_NUCLEUS | GOBP_MRNA_EXPORT_FROM_NUCLEUS | 106 | 0.389762243 | 1.457102184 | 0.03269729 | 0.195414568 | 0.165638955 |
| GOBP_PYRIMIDINE_NUCLEOSIDE_MONOPHOSPHATE_METABOLIC_PROCESS | GOBP_PYRIMIDINE_NUCLEOSIDE_MONOPHOSPHATE_METABOLIC_PROCESS | 15 | 0.638738785 | 1.591808326 | 0.032703992 | 0.195414568 | 0.165638955 |
| GOBP_ENDOPLASMIC_RETICULUM_UNFOLDED_PROTEIN_RESPONSE | GOBP_ENDOPLASMIC_RETICULUM_UNFOLDED_PROTEIN_RESPONSE | 119 | 0.358853112 | 1.363034863 | 0.032755725 | 0.195414568 | 0.165638955 |
| GOBP_CELLULAR_RESPONSE_TO_FLUID_SHEAR_STRESS | GOBP_CELLULAR_RESPONSE_TO_FLUID_SHEAR_STRESS | 17 | -0.642840624 | -1.597547872 | 0.032761367 | 0.195414568 | 0.165638955 |
| GOMF_EXTRACELLULAR_MATRIX_STRUCTURAL_CONSTITUENT_CONFERRING_COMPRESSION_RESISTANCE | GOMF_EXTRACELLULAR_MATRIX_STRUCTURAL_CONSTITUENT_CONFERRING_COMPRESSION_RESISTANCE | 13 | -0.669074784 | -1.55277823 | 0.032798175 | 0.195414568 | 0.165638955 |
| GOMF_ANTIOXIDANT_ACTIVITY | GOMF_ANTIOXIDANT_ACTIVITY | 66 | 0.43080157 | 1.477626883 | 0.032804989 | 0.195414568 | 0.165638955 |
| GOBP_LEUKOTRIENE_BIOSYNTHETIC_PROCESS | GOBP_LEUKOTRIENE_BIOSYNTHETIC_PROCESS | 15 | -0.657506295 | -1.59111977 | 0.032984233 | 0.196294099 | 0.16638447 |
| GOBP_PTERIDINE_CONTAINING_COMPOUND_BIOSYNTHETIC_PROCESS | GOBP_PTERIDINE_CONTAINING_COMPOUND_BIOSYNTHETIC_PROCESS | 15 | 0.635587314 | 1.583954507 | 0.033065359 | 0.196400645 | 0.166474781 |
| GOBP_REGULATION_OF_SKELETAL_MUSCLE_CELL_DIFFERENTIATION | GOBP_REGULATION_OF_SKELETAL_MUSCLE_CELL_DIFFERENTIATION | 15 | 0.636996665 | 1.587466767 | 0.033065359 | 0.196400645 | 0.166474781 |
| GOBP_NEGATIVE_REGULATION_OF_MYELOID_CELL_DIFFERENTIATION | GOBP_NEGATIVE_REGULATION_OF_MYELOID_CELL_DIFFERENTIATION | 81 | -0.424859259 | -1.393534719 | 0.033123211 | 0.196445384 | 0.166512704 |
| GOMF_ACETYLGALACTOSAMINYLTRANSFERASE_ACTIVITY | GOMF_ACETYLGALACTOSAMINYLTRANSFERASE_ACTIVITY | 41 | 0.460106861 | 1.421344356 | 0.033138406 | 0.196445384 | 0.166512704 |
| GOBP_REGULATION_OF_RESPONSE_TO_WOUNDING | GOBP_REGULATION_OF_RESPONSE_TO_WOUNDING | 135 | 0.354821609 | 1.366278521 | 0.033167746 | 0.196445384 | 0.166512704 |
| GOBP_MESENCHYME_DEVELOPMENT | GOBP_MESENCHYME_DEVELOPMENT | 238 | 0.308269867 | 1.284337084 | 0.033217161 | 0.196550684 | 0.166601959 |
| GOBP_DNA_DOUBLE_STRAND_BREAK_PROCESSING | GOBP_DNA_DOUBLE_STRAND_BREAK_PROCESSING | 21 | 0.593279496 | 1.588569616 | 0.033306712 | 0.196801705 | 0.166814731 |
| GOBP_POSITIVE_THYMIC_T_CELL_SELECTION | GOBP_POSITIVE_THYMIC_T_CELL_SELECTION | 13 | -0.669050913 | -1.552722831 | 0.033322935 | 0.196801705 | 0.166814731 |
| GOBP_PURINE_NUCLEOSIDE_MONOPHOSPHATE_METABOLIC_PROCESS | GOBP_PURINE_NUCLEOSIDE_MONOPHOSPHATE_METABOLIC_PROCESS | 39 | 0.495301012 | 1.51000783 | 0.033397976 | 0.197057571 | 0.167031611 |
| GOMF_CELL_CELL_ADHESION_MEDIATOR_ACTIVITY | GOMF_CELL_CELL_ADHESION_MEDIATOR_ACTIVITY | 41 | 0.459822743 | 1.420466669 | 0.033525985 | 0.197562014 | 0.167459191 |
| GOBP_REGULATION_OF_COAGULATION | GOBP_REGULATION_OF_COAGULATION | 61 | 0.428217495 | 1.44904667 | 0.033547067 | 0.197562014 | 0.167459191 |
| GOBP_POSTSYNAPTIC_CYTOSKELETON_ORGANIZATION | GOBP_POSTSYNAPTIC_CYTOSKELETON_ORGANIZATION | 11 | 0.735206203 | 1.7081776 | 0.033889876 | 0.199203218 | 0.168850322 |
| GOBP_PYRIMIDINE_CONTAINING_COMPOUND_SALVAGE | GOBP_PYRIMIDINE_CONTAINING_COMPOUND_SALVAGE | 10 | 0.728963502 | 1.625726916 | 0.033889876 | 0.199203218 | 0.168850322 |
| GOBP_VESICLE_DOCKING_INVOLVED_IN_EXOCYTOSIS | GOBP_VESICLE_DOCKING_INVOLVED_IN_EXOCYTOSIS | 39 | 0.493208913 | 1.503629717 | 0.034179206 | 0.200713995 | 0.1701309 |
| GOBP_HIPPOCAMPUS_DEVELOPMENT | GOBP_HIPPOCAMPUS_DEVELOPMENT | 52 | 0.446404493 | 1.455136788 | 0.034259672 | 0.200996547 | 0.170370399 |
| GOBP_POSITIVE_REGULATION_OF_MITOTIC_NUCLEAR_DIVISION | GOBP_POSITIVE_REGULATION_OF_MITOTIC_NUCLEAR_DIVISION | 35 | 0.530061535 | 1.597204224 | 0.034509915 | 0.202273683 | 0.171452936 |
| GOCC_OUTER_MITOCHONDRIAL_MEMBRANE_PROTEIN_COMPLEX | GOCC_OUTER_MITOCHONDRIAL_MEMBRANE_PROTEIN_COMPLEX | 21 | 0.58948532 | 1.5784103 | 0.034762729 | 0.203563463 | 0.17254619 |
| GOBP_REGULATION_OF_SYSTEM_PROCESS | GOBP_REGULATION_OF_SYSTEM_PROCESS | 381 | 0.276626447 | 1.206855866 | 0.035064542 | 0.205137479 | 0.173880371 |
| GOBP_CELLULAR_RESPONSE_TO_STARVATION | GOBP_CELLULAR_RESPONSE_TO_STARVATION | 146 | 0.351069224 | 1.368183468 | 0.035157811 | 0.205422245 | 0.174121747 |
| GOCC_RECEPTOR_COMPLEX | GOCC_RECEPTOR_COMPLEX | 335 | -0.32237348 | -1.273562272 | 0.035202086 | 0.205422245 | 0.174121747 |
| GOBP_POSITIVE_REGULATION_OF_MONOCYTE_CHEMOTAXIS | GOBP_POSITIVE_REGULATION_OF_MONOCYTE_CHEMOTAXIS | 17 | 0.619405092 | 1.583109059 | 0.035212408 | 0.205422245 | 0.174121747 |
| GOMF_2_OXOGLUTARATE_DEPENDENT_DIOXYGENASE_ACTIVITY | GOMF_2_OXOGLUTARATE_DEPENDENT_DIOXYGENASE_ACTIVITY | 42 | 0.493078134 | 1.525950321 | 0.035329412 | 0.20591148 | 0.174536436 |
| GOMF_INTERMEDIATE_FILAMENT_BINDING | GOMF_INTERMEDIATE_FILAMENT_BINDING | 13 | 0.652700319 | 1.591912934 | 0.035396542 | 0.206109385 | 0.174704186 |
| GOBP_NEGATIVE_REGULATION_OF_COAGULATION | GOBP_NEGATIVE_REGULATION_OF_COAGULATION | 44 | 0.470124562 | 1.471083178 | 0.035516836 | 0.206616201 | 0.175133778 |
| GOBP_REGULATION_OF_ASTROCYTE_DIFFERENTIATION | GOBP_REGULATION_OF_ASTROCYTE_DIFFERENTIATION | 15 | 0.63184287 | 1.574622936 | 0.035594925 | 0.206876771 | 0.175354644 |
| GOMF_PEPTIDE_TRANSMEMBRANE_TRANSPORTER_ACTIVITY | GOMF_PEPTIDE_TRANSMEMBRANE_TRANSPORTER_ACTIVITY | 34 | 0.502745108 | 1.49898087 | 0.03564859 | 0.206921949 | 0.175392939 |
| GOMF_METALLOENDOPEPTIDASE_ACTIVITY | GOMF_METALLOENDOPEPTIDASE_ACTIVITY | 73 | 0.414324575 | 1.465887275 | 0.035669308 | 0.206921949 | 0.175392939 |
| GOBP_REGULATION_OF_SYSTEMIC_ARTERIAL_BLOOD_PRESSURE_BY_RENIN_ANGIOTENSIN | GOBP_REGULATION_OF_SYSTEMIC_ARTERIAL_BLOOD_PRESSURE_BY_RENIN_ANGIOTENSIN | 20 | -0.59902707 | -1.545029228 | 0.035723363 | 0.207042215 | 0.175494879 |
| GOMF_RECEPTOR_ANTAGONIST_ACTIVITY | GOMF_RECEPTOR_ANTAGONIST_ACTIVITY | 16 | 0.625430089 | 1.583095832 | 0.035970121 | 0.20803661 | 0.176337757 |
| GOBP_OVULATION_CYCLE_PROCESS | GOBP_OVULATION_CYCLE_PROCESS | 34 | 0.501056844 | 1.493947154 | 0.036038188 | 0.20803661 | 0.176337757 |
| GOBP_PROTEIN_TRIMERIZATION | GOBP_PROTEIN_TRIMERIZATION | 15 | -0.648903667 | -1.570302005 | 0.036052475 | 0.20803661 | 0.176337757 |
| GOMF_PROTEIN_TRANSMEMBRANE_TRANSPORTER_ACTIVITY | GOMF_PROTEIN_TRANSMEMBRANE_TRANSPORTER_ACTIVITY | 20 | 0.600174856 | 1.596313822 | 0.03605638 | 0.20803661 | 0.176337757 |
| GOMF_TRNA_BINDING | GOMF_TRNA_BINDING | 64 | 0.442408373 | 1.511064556 | 0.036062358 | 0.20803661 | 0.176337757 |
| GOBP_DIVALENT_INORGANIC_CATION_HOMEOSTASIS | GOBP_DIVALENT_INORGANIC_CATION_HOMEOSTASIS | 367 | 0.275586283 | 1.198483232 | 0.036238516 | 0.208579644 | 0.176798048 |
| GOBP_INNER_EAR_MORPHOGENESIS | GOBP_INNER_EAR_MORPHOGENESIS | 62 | 0.437215598 | 1.483541032 | 0.036249384 | 0.208579644 | 0.176798048 |
| GOMF_FIBRONECTIN_BINDING | GOMF_FIBRONECTIN_BINDING | 25 | 0.568348147 | 1.564902299 | 0.036262643 | 0.208579644 | 0.176798048 |
| GOBP_DICARBOXYLIC_ACID_BIOSYNTHETIC_PROCESS | GOBP_DICARBOXYLIC_ACID_BIOSYNTHETIC_PROCESS | 12 | 0.675591093 | 1.610208979 | 0.036290777 | 0.208579644 | 0.176798048 |
| GOBP_PURINE_NUCLEOBASE_BIOSYNTHETIC_PROCESS | GOBP_PURINE_NUCLEOBASE_BIOSYNTHETIC_PROCESS | 10 | 0.727470055 | 1.622396244 | 0.036351648 | 0.208736404 | 0.176930922 |
| GOBP_ODONTOGENESIS_OF_DENTIN_CONTAINING_TOOTH | GOBP_ODONTOGENESIS_OF_DENTIN_CONTAINING_TOOTH | 59 | 0.426823116 | 1.433917553 | 0.036385454 | 0.208737606 | 0.176931941 |
| GOBP_REGULATION_OF_HEMATOPOIETIC_PROGENITOR_CELL_DIFFERENTIATION | GOBP_REGULATION_OF_HEMATOPOIETIC_PROGENITOR_CELL_DIFFERENTIATION | 83 | 0.409768354 | 1.464066576 | 0.036420447 | 0.208745609 | 0.176938725 |
| GOMF_TRANSLATION_FACTOR_ACTIVITY_RNA_BINDING | GOMF_TRANSLATION_FACTOR_ACTIVITY_RNA_BINDING | 79 | 0.406949655 | 1.456719529 | 0.036566022 | 0.20905432 | 0.177200397 |
| GOBP_NUCLEOSIDE_TRIPHOSPHATE_BIOSYNTHETIC_PROCESS | GOBP_NUCLEOSIDE_TRIPHOSPHATE_BIOSYNTHETIC_PROCESS | 77 | 0.407080075 | 1.44951358 | 0.036575253 | 0.20905432 | 0.177200397 |
| GOMF_ION_CHANNEL_REGULATOR_ACTIVITY | GOMF_ION_CHANNEL_REGULATOR_ACTIVITY | 81 | 0.3866312 | 1.391194281 | 0.036575253 | 0.20905432 | 0.177200397 |
| GOBP_POSITIVE_REGULATION_OF_POTASSIUM_ION_TRANSMEMBRANE_TRANSPORTER_ACTIVITY | GOBP_POSITIVE_REGULATION_OF_POTASSIUM_ION_TRANSMEMBRANE_TRANSPORTER_ACTIVITY | 15 | 0.63003631 | 1.570120786 | 0.036679025 | 0.209262427 | 0.177376794 |
| GOBP_RRNA_CONTAINING_RIBONUCLEOPROTEIN_COMPLEX_EXPORT_FROM_NUCLEUS | GOBP_RRNA_CONTAINING_RIBONUCLEOPROTEIN_COMPLEX_EXPORT_FROM_NUCLEUS | 15 | 0.629477752 | 1.568728798 | 0.036679025 | 0.209262427 | 0.177376794 |
| GOBP_GLUTATHIONE_DERIVATIVE_METABOLIC_PROCESS | GOBP_GLUTATHIONE_DERIVATIVE_METABOLIC_PROCESS | 20 | -0.59817202 | -1.542823857 | 0.036751356 | 0.209482729 | 0.177563528 |
| GOCC_BASEMENT_MEMBRANE | GOCC_BASEMENT_MEMBRANE | 76 | 0.406450858 | 1.445353838 | 0.036886631 | 0.210061082 | 0.178053757 |
| GOCC_PROTEASOME_REGULATORY_PARTICLE_BASE_SUBCOMPLEX | GOCC_PROTEASOME_REGULATORY_PARTICLE_BASE_SUBCOMPLEX | 12 | 0.670979866 | 1.599218544 | 0.036982009 | 0.210263145 | 0.178225031 |
| GOBP_FOLIC_ACID_CONTAINING_COMPOUND_BIOSYNTHETIC_PROCESS | GOBP_FOLIC_ACID_CONTAINING_COMPOUND_BIOSYNTHETIC_PROCESS | 10 | 0.723761722 | 1.61412596 | 0.037057483 | 0.210263145 | 0.178225031 |
| GOBP_LAYER_FORMATION_IN_CEREBRAL_CORTEX | GOBP_LAYER_FORMATION_IN_CEREBRAL_CORTEX | 10 | 0.725857386 | 1.618799688 | 0.037057483 | 0.210263145 | 0.178225031 |
| GOBP_POSITIVE_REGULATION_OF_CELL_CYCLE_CHECKPOINT | GOBP_POSITIVE_REGULATION_OF_CELL_CYCLE_CHECKPOINT | 10 | 0.726299649 | 1.619786017 | 0.037057483 | 0.210263145 | 0.178225031 |
| GOBP_NEGATIVE_REGULATION_OF_LONG_TERM_SYNAPTIC_POTENTIATION | GOBP_NEGATIVE_REGULATION_OF_LONG_TERM_SYNAPTIC_POTENTIATION | 11 | -0.694910412 | -1.544727317 | 0.037325596 | 0.211398293 | 0.179187215 |
| GOBP_REGULATION_OF_PHOSPHATIDYLCHOLINE_METABOLIC_PROCESS | GOBP_REGULATION_OF_PHOSPHATIDYLCHOLINE_METABOLIC_PROCESS | 10 | -0.69775102 | -1.52582983 | 0.037325596 | 0.211398293 | 0.179187215 |
| GOBP_DETECTION_OF_TEMPERATURE_STIMULUS | GOBP_DETECTION_OF_TEMPERATURE_STIMULUS | 15 | 0.626454842 | 1.561195368 | 0.037405319 | 0.211656873 | 0.179406395 |
| GOBP_FORMATION_OF_EXTRACHROMOSOMAL_CIRCULAR_DNA | GOBP_FORMATION_OF_EXTRACHROMOSOMAL_CIRCULAR_DNA | 12 | 0.667489818 | 1.590900337 | 0.037673241 | 0.212785317 | 0.180362897 |
| GOMF_RNA_POLYMERASE_II_CTD_HEPTAPEPTIDE_REPEAT_KINASE_ACTIVITY | GOMF_RNA_POLYMERASE_II_CTD_HEPTAPEPTIDE_REPEAT_KINASE_ACTIVITY | 12 | 0.670659402 | 1.598454749 | 0.037673241 | 0.212785317 | 0.180362897 |
| GOBP_RESPONSE_TO_ACETYLCHOLINE | GOBP_RESPONSE_TO_ACETYLCHOLINE | 16 | 0.622015953 | 1.574453931 | 0.037766717 | 0.212946058 | 0.180499145 |
| GOBP_POSITIVE_REGULATION_OF_HISTONE_H3_K4_METHYLATION | GOBP_POSITIVE_REGULATION_OF_HISTONE_H3_K4_METHYLATION | 17 | 0.613215821 | 1.567290185 | 0.037770249 | 0.212946058 | 0.180499145 |
| GOMF_NUCLEAR_IMPORT_SIGNAL_RECEPTOR_ACTIVITY | GOMF_NUCLEAR_IMPORT_SIGNAL_RECEPTOR_ACTIVITY | 20 | 0.593021618 | 1.577288013 | 0.037857285 | 0.213006277 | 0.180550188 |
| GOBP_NEGATIVE_REGULATION_OF_SIGNALING_RECEPTOR_ACTIVITY | GOBP_NEGATIVE_REGULATION_OF_SIGNALING_RECEPTOR_ACTIVITY | 43 | 0.472665185 | 1.474812964 | 0.037892618 | 0.213006277 | 0.180550188 |
| GOBP_RESPONSE_TO_EPIDERMAL_GROWTH_FACTOR | GOBP_RESPONSE_TO_EPIDERMAL_GROWTH_FACTOR | 43 | 0.472929723 | 1.475638378 | 0.037892618 | 0.213006277 | 0.180550188 |
| GOCC_SODIUM_CHANNEL_COMPLEX | GOCC_SODIUM_CHANNEL_COMPLEX | 12 | -0.673306442 | -1.532671567 | 0.037918066 | 0.213006277 | 0.180550188 |
| GOBP_PEPTIDYL_THREONINE_MODIFICATION | GOBP_PEPTIDYL_THREONINE_MODIFICATION | 111 | 0.355654058 | 1.336108598 | 0.037991788 | 0.213227625 | 0.180737809 |
| GOBP_POSITIVE_REGULATION_OF_VASCULAR_ENDOTHELIAL_GROWTH_FACTOR_PRODUCTION | GOBP_POSITIVE_REGULATION_OF_VASCULAR_ENDOTHELIAL_GROWTH_FACTOR_PRODUCTION | 24 | 0.567045954 | 1.550320315 | 0.038153559 | 0.213942293 | 0.181343582 |
| GOBP_INTRINSIC_APOPTOTIC_SIGNALING_PATHWAY_IN_RESPONSE_TO_DNA_DAMAGE | GOBP_INTRINSIC_APOPTOTIC_SIGNALING_PATHWAY_IN_RESPONSE_TO_DNA_DAMAGE | 98 | 0.383099634 | 1.415250454 | 0.038287072 | 0.214165201 | 0.181532525 |
| GOBP_NEGATIVE_REGULATION_OF_OSSIFICATION | GOBP_NEGATIVE_REGULATION_OF_OSSIFICATION | 27 | 0.54325774 | 1.530746436 | 0.038331193 | 0.214165201 | 0.181532525 |
| GOBP_RESPONSE_TO_PH | GOBP_RESPONSE_TO_PH | 27 | 0.544600147 | 1.534528959 | 0.038331193 | 0.214165201 | 0.181532525 |
| GOMF_STEROL_TRANSPORTER_ACTIVITY | GOMF_STEROL_TRANSPORTER_ACTIVITY | 27 | 0.54391983 | 1.532612018 | 0.038331193 | 0.214165201 | 0.181532525 |
| GOBP_REGULATION_OF_REACTIVE_OXYGEN_SPECIES_METABOLIC_PROCESS | GOBP_REGULATION_OF_REACTIVE_OXYGEN_SPECIES_METABOLIC_PROCESS | 164 | 0.346475193 | 1.381614661 | 0.03853527 | 0.215097056 | 0.182322392 |
| GOBP_RESPONSE_TO_FLUID_SHEAR_STRESS | GOBP_RESPONSE_TO_FLUID_SHEAR_STRESS | 30 | -0.537676634 | -1.506979286 | 0.038582239 | 0.215097056 | 0.182322392 |
| GOBP_ATP_BIOSYNTHETIC_PROCESS | GOBP_ATP_BIOSYNTHETIC_PROCESS | 51 | 0.472713116 | 1.540337171 | 0.038601838 | 0.215097056 | 0.182322392 |
| GOCC_NUCLEAR_MEMBRANE | GOCC_NUCLEAR_MEMBRANE | 264 | 0.296533801 | 1.247054812 | 0.0386722 | 0.215296039 | 0.182491055 |
| GOMF_ENDONUCLEASE_ACTIVITY_ACTIVE_WITH_EITHER_RIBO_OR_DEOXYRIBONUCLEIC_ACIDS_AND_PRODUCING_5_PHOSPHOMONOESTERS | GOMF_ENDONUCLEASE_ACTIVITY_ACTIVE_WITH_EITHER_RIBO_OR_DEOXYRIBONUCLEIC_ACIDS_AND_PRODUCING_5_PHOSPHOMONOESTERS | 37 | 0.484076701 | 1.469619931 | 0.038737542 | 0.215466739 | 0.182635746 |
| GOCC_PROTEIN_DNA_COMPLEX | GOCC_PROTEIN_DNA_COMPLEX | 160 | -0.370047585 | -1.355705682 | 0.038819722 | 0.21573071 | 0.182859495 |
| GOBP_SYNAPTIC_TRANSMISSION_CHOLINERGIC | GOBP_SYNAPTIC_TRANSMISSION_CHOLINERGIC | 11 | -0.690171048 | -1.534192111 | 0.03889168 | 0.21593745 | 0.183034734 |
| GOBP_CELLULAR_RESPONSE_TO_UV | GOBP_CELLULAR_RESPONSE_TO_UV | 85 | 0.387243052 | 1.391305153 | 0.038941887 | 0.216023163 | 0.183107387 |
| GOBP_REGULATION_OF_EXTRINSIC_APOPTOTIC_SIGNALING_PATHWAY | GOBP_REGULATION_OF_EXTRINSIC_APOPTOTIC_SIGNALING_PATHWAY | 139 | 0.35116518 | 1.36473228 | 0.039058886 | 0.216341815 | 0.183377485 |
| GOBP_POSITIVE_REGULATION_OF_PEPTIDYL_THREONINE_PHOSPHORYLATION | GOBP_POSITIVE_REGULATION_OF_PEPTIDYL_THREONINE_PHOSPHORYLATION | 27 | 0.542339551 | 1.52815924 | 0.039075465 | 0.216341815 | 0.183377485 |
| GOBP_ESTABLISHMENT_OF_PROTEIN_LOCALIZATION_TO_MITOCHONDRIAL_MEMBRANE | GOBP_ESTABLISHMENT_OF_PROTEIN_LOCALIZATION_TO_MITOCHONDRIAL_MEMBRANE | 53 | 0.450081138 | 1.480076284 | 0.039106416 | 0.216341815 | 0.183377485 |
| GOBP_MESONEPHROS_DEVELOPMENT | GOBP_MESONEPHROS_DEVELOPMENT | 74 | -0.43997543 | -1.424824404 | 0.03916974 | 0.216341815 | 0.183377485 |
| GOBP_POLYOL_BIOSYNTHETIC_PROCESS | GOBP_POLYOL_BIOSYNTHETIC_PROCESS | 54 | 0.447903409 | 1.49026538 | 0.039173433 | 0.216341815 | 0.183377485 |
| GOBP_CELLULAR_SENESCENCE | GOBP_CELLULAR_SENESCENCE | 64 | 0.43923779 | 1.500235294 | 0.039359453 | 0.217176095 | 0.184084645 |
| GOBP_ASTROCYTE_DIFFERENTIATION | GOBP_ASTROCYTE_DIFFERENTIATION | 54 | 0.447817201 | 1.489978547 | 0.039567133 | 0.218128303 | 0.184891763 |
| GOBP_OVULATION_CYCLE | GOBP_OVULATION_CYCLE | 50 | 0.463039624 | 1.505385536 | 0.039801884 | 0.219227932 | 0.18582384 |
| GOMF_CAMP_DEPENDENT_PROTEIN_KINASE_REGULATOR_ACTIVITY | GOMF_CAMP_DEPENDENT_PROTEIN_KINASE_REGULATOR_ACTIVITY | 10 | 0.716972739 | 1.59898524 | 0.039880828 | 0.219321383 | 0.185903052 |
| GOBP_POSITIVE_REGULATION_OF_TELOMERE_MAINTENANCE_VIA_TELOMERE_LENGTHENING | GOBP_POSITIVE_REGULATION_OF_TELOMERE_MAINTENANCE_VIA_TELOMERE_LENGTHENING | 35 | 0.52217612 | 1.573443553 | 0.039911861 | 0.219321383 | 0.185903052 |
| GOBP_RESPONSE_TO_MUSCLE_ACTIVITY | GOBP_RESPONSE_TO_MUSCLE_ACTIVITY | 19 | 0.59428194 | 1.56989537 | 0.039924752 | 0.219321383 | 0.185903052 |
| GOMF_E_BOX_BINDING | GOMF_E_BOX_BINDING | 36 | 0.503442711 | 1.517131558 | 0.040014992 | 0.219622922 | 0.186158645 |
| GOBP_NEURON_PROJECTION_REGENERATION | GOBP_NEURON_PROJECTION_REGENERATION | 50 | 0.462473647 | 1.503545491 | 0.0402019 | 0.220454019 | 0.186863107 |
| GOMF_ALCOHOL_BINDING | GOMF_ALCOHOL_BINDING | 68 | 0.410521987 | 1.41414606 | 0.040553616 | 0.221864293 | 0.188058495 |
| GOBP_NEGATIVE_REGULATION_OF_GENE_SILENCING | GOBP_NEGATIVE_REGULATION_OF_GENE_SILENCING | 30 | -0.535980152 | -1.502224452 | 0.040573554 | 0.221864293 | 0.188058495 |
| GOBP_REGULATION_OF_VENTRICULAR_CARDIAC_MUSCLE_CELL_ACTION_POTENTIAL | GOBP_REGULATION_OF_VENTRICULAR_CARDIAC_MUSCLE_CELL_ACTION_POTENTIAL | 10 | 0.715413221 | 1.595507217 | 0.040586665 | 0.221864293 | 0.188058495 |
| GOBP_RESPONSE_TO_CADMIUM_ION | GOBP_RESPONSE_TO_CADMIUM_ION | 51 | 0.46836066 | 1.526154677 | 0.040601915 | 0.221864293 | 0.188058495 |
| GOBP_ANTIGEN_PROCESSING_AND_PRESENTATION_OF_PEPTIDE_ANTIGEN_VIA_MHC_CLASS_I | GOBP_ANTIGEN_PROCESSING_AND_PRESENTATION_OF_PEPTIDE_ANTIGEN_VIA_MHC_CLASS_I | 96 | 0.371469575 | 1.369796881 | 0.040782128 | 0.222653219 | 0.188727211 |
| GOBP_SOMATIC_DIVERSIFICATION_OF_IMMUNE_RECEPTORS | GOBP_SOMATIC_DIVERSIFICATION_OF_IMMUNE_RECEPTORS | 68 | 0.40900872 | 1.408933233 | 0.040973857 | 0.223503576 | 0.189447998 |
| GOBP_REGULATION_OF_PHOSPHATIDYLINOSITOL_3_KINASE_ACTIVITY | GOBP_REGULATION_OF_PHOSPHATIDYLINOSITOL_3_KINASE_ACTIVITY | 51 | -0.467341155 | -1.450093359 | 0.041401274 | 0.225636943 | 0.1912563 |
| GOMF_MRNA_5_UTR_BINDING | GOMF_MRNA_5_UTR_BINDING | 25 | 0.562757019 | 1.549507565 | 0.041593553 | 0.226339582 | 0.191851878 |
| GOBP_POSITIVE_REGULATION_OF_CALCIUM_ION_TRANSMEMBRANE_TRANSPORT | GOBP_POSITIVE_REGULATION_OF_CALCIUM_ION_TRANSMEMBRANE_TRANSPORT | 56 | 0.446462753 | 1.484939124 | 0.041603059 | 0.226339582 | 0.191851878 |
| GOBP_EMBRYONIC_APPENDAGE_MORPHOGENESIS | GOBP_EMBRYONIC_APPENDAGE_MORPHOGENESIS | 95 | 0.364457331 | 1.347027296 | 0.041654465 | 0.22642099 | 0.191920881 |
| GOBP_PHOSPHATIDIC_ACID_METABOLIC_PROCESS | GOBP_PHOSPHATIDIC_ACID_METABOLIC_PROCESS | 42 | -0.494125004 | -1.489905591 | 0.041733547 | 0.226652561 | 0.192117167 |
| GOBP_HIGH_DENSITY_LIPOPROTEIN_PARTICLE_REMODELING | GOBP_HIGH_DENSITY_LIPOPROTEIN_PARTICLE_REMODELING | 14 | 0.6554257 | 1.608835833 | 0.041882396 | 0.227262292 | 0.192633992 |
| GOBP_MATERNAL_PROCESS_INVOLVED_IN_FEMALE_PREGNANCY | GOBP_MATERNAL_PROCESS_INVOLVED_IN_FEMALE_PREGNANCY | 47 | 0.457615593 | 1.462411125 | 0.04198534 | 0.227622092 | 0.19293897 |
| GOBP_STEROID_HORMONE_SECRETION | GOBP_STEROID_HORMONE_SECRETION | 17 | -0.627541651 | -1.559527809 | 0.04222973 | 0.228747437 | 0.193892844 |
| GOBP_VASCULAR_ENDOTHELIAL_GROWTH_FACTOR_RECEPTOR_SIGNALING_PATHWAY | GOBP_VASCULAR_ENDOTHELIAL_GROWTH_FACTOR_RECEPTOR_SIGNALING_PATHWAY | 86 | 0.379954562 | 1.371712976 | 0.04228027 | 0.228821704 | 0.193955795 |
| GOBP_GLANDULAR_EPITHELIAL_CELL_DIFFERENTIATION | GOBP_GLANDULAR_EPITHELIAL_CELL_DIFFERENTIATION | 40 | 0.479742126 | 1.476302582 | 0.042464495 | 0.229618716 | 0.194631365 |
| GOBP_REGULATION_OF_RECEPTOR_SIGNALING_PATHWAY_VIA_STAT | GOBP_REGULATION_OF_RECEPTOR_SIGNALING_PATHWAY_VIA_STAT | 65 | 0.420547655 | 1.449867833 | 0.042666183 | 0.230508693 | 0.195385735 |
| GOBP_ANTIMICROBIAL_HUMORAL_RESPONSE | GOBP_ANTIMICROBIAL_HUMORAL_RESPONSE | 98 | -0.409595962 | -1.391681247 | 0.042794249 | 0.230999711 | 0.195801936 |
| GOBP_MULTI_MULTICELLULAR_ORGANISM_PROCESS | GOBP_MULTI_MULTICELLULAR_ORGANISM_PROCESS | 152 | 0.347163753 | 1.369163431 | 0.042987797 | 0.231843041 | 0.196516766 |
| GOCC_METHYLTRANSFERASE_COMPLEX | GOCC_METHYLTRANSFERASE_COMPLEX | 94 | 0.382771913 | 1.411589843 | 0.043088593 | 0.232010041 | 0.19665832 |
| GOBP_POSITIVE_REGULATION_OF_CELL_DEVELOPMENT | GOBP_POSITIVE_REGULATION_OF_CELL_DEVELOPMENT | 242 | 0.302958534 | 1.261096575 | 0.043093447 | 0.232010041 | 0.19665832 |
| GOMF_TOLL_LIKE_RECEPTOR_BINDING | GOMF_TOLL_LIKE_RECEPTOR_BINDING | 12 | 0.658209802 | 1.568782277 | 0.043203113 | 0.232340402 | 0.196938344 |
| GOBP_TRANSFORMING_GROWTH_FACTOR_BETA_PRODUCTION | GOBP_TRANSFORMING_GROWTH_FACTOR_BETA_PRODUCTION | 40 | 0.476084272 | 1.465046326 | 0.0432296 | 0.232340402 | 0.196938344 |
| GOBP_VESICLE_DOCKING | GOBP_VESICLE_DOCKING | 60 | 0.424495254 | 1.426136214 | 0.043377597 | 0.23290711 | 0.197418701 |
| GOBP_NUCLEOSIDE_MONOPHOSPHATE_CATABOLIC_PROCESS | GOBP_NUCLEOSIDE_MONOPHOSPHATE_CATABOLIC_PROCESS | 10 | 0.710477015 | 1.584498542 | 0.043410017 | 0.23290711 | 0.197418701 |
| GOBP_POSITIVE_REGULATION_OF_CATION_CHANNEL_ACTIVITY | GOBP_POSITIVE_REGULATION_OF_CATION_CHANNEL_ACTIVITY | 52 | 0.431621242 | 1.406948088 | 0.043596677 | 0.233706777 | 0.198096522 |
| GOBP_TRIGLYCERIDE_METABOLIC_PROCESS | GOBP_TRIGLYCERIDE_METABOLIC_PROCESS | 73 | 0.406995247 | 1.439955989 | 0.043834261 | 0.234777814 | 0.199004364 |
| GOMF_ORGANIC_ACID_SODIUM_SYMPORTER_ACTIVITY | GOMF_ORGANIC_ACID_SODIUM_SYMPORTER_ACTIVITY | 17 | -0.622679625 | -1.547445002 | 0.043918919 | 0.235028633 | 0.199216965 |
| GOBP_PEPTIDYL_CYSTEINE_MODIFICATION | GOBP_PEPTIDYL_CYSTEINE_MODIFICATION | 42 | 0.475298849 | 1.470927993 | 0.044033104 | 0.235436897 | 0.199563021 |
| GOBP_INTRINSIC_APOPTOTIC_SIGNALING_PATHWAY_IN_RESPONSE_TO_ENDOPLASMIC_RETICULUM_STRESS | GOBP_INTRINSIC_APOPTOTIC_SIGNALING_PATHWAY_IN_RESPONSE_TO_ENDOPLASMIC_RETICULUM_STRESS | 57 | 0.43366527 | 1.443745943 | 0.044195533 | 0.236102187 | 0.20012694 |
| GOMF_EXOGENOUS_PROTEIN_BINDING | GOMF_EXOGENOUS_PROTEIN_BINDING | 71 | 0.4007314 | 1.396357886 | 0.044439707 | 0.237202662 | 0.201059733 |
| GOBP_TUMOR_NECROSIS_FACTOR_MEDIATED_SIGNALING_PATHWAY | GOBP_TUMOR_NECROSIS_FACTOR_MEDIATED_SIGNALING_PATHWAY | 157 | 0.347082437 | 1.372917274 | 0.044537674 | 0.237521518 | 0.201330005 |
| GOBP_ADULT_LOCOMOTORY_BEHAVIOR | GOBP_ADULT_LOCOMOTORY_BEHAVIOR | 43 | 0.46722838 | 1.457849012 | 0.044591162 | 0.237602822 | 0.201398921 |
| GOBP_REGULATION_OF_CELL_MORPHOGENESIS_INVOLVED_IN_DIFFERENTIATION | GOBP_REGULATION_OF_CELL_MORPHOGENESIS_INVOLVED_IN_DIFFERENTIATION | 87 | 0.373893016 | 1.353449183 | 0.044662107 | 0.237651475 | 0.201440161 |
| GOBP_POSITIVE_REGULATION_OF_PATHWAY_RESTRICTED_SMAD_PROTEIN_PHOSPHORYLATION | GOBP_POSITIVE_REGULATION_OF_PATHWAY_RESTRICTED_SMAD_PROTEIN_PHOSPHORYLATION | 36 | 0.49726842 | 1.49852525 | 0.044676794 | 0.237651475 | 0.201440161 |
| GOCC_CONNEXIN_COMPLEX | GOCC_CONNEXIN_COMPLEX | 10 | 0.707669154 | 1.57823648 | 0.044821694 | 0.238014687 | 0.201748029 |
| GOMF_GAP_JUNCTION_CHANNEL_ACTIVITY | GOMF_GAP_JUNCTION_CHANNEL_ACTIVITY | 10 | 0.707669154 | 1.57823648 | 0.044821694 | 0.238014687 | 0.201748029 |
| GOBP_REGULATION_OF_PROTEIN_LOCALIZATION_TO_NUCLEUS | GOBP_REGULATION_OF_PROTEIN_LOCALIZATION_TO_NUCLEUS | 121 | 0.358380201 | 1.36700982 | 0.045213549 | 0.239890504 | 0.203338025 |
| GOBP_REGULATION_OF_ANION_TRANSMEMBRANE_TRANSPORT | GOBP_REGULATION_OF_ANION_TRANSMEMBRANE_TRANSPORT | 93 | -0.405006256 | -1.360955862 | 0.045317221 | 0.240235402 | 0.20363037 |
| GOBP_RESPONSE_TO_INSULIN | GOBP_RESPONSE_TO_INSULIN | 234 | 0.300218269 | 1.244134797 | 0.045453597 | 0.240752938 | 0.204069049 |
| GOBP_CENTROMERIC_SISTER_CHROMATID_COHESION | GOBP_CENTROMERIC_SISTER_CHROMATID_COHESION | 10 | 0.706928876 | 1.576585519 | 0.045527533 | 0.24093915 | 0.204226887 |
| GOBP_POSITIVE_REGULATION_OF_TRANSPORTER_ACTIVITY | GOBP_POSITIVE_REGULATION_OF_TRANSPORTER_ACTIVITY | 86 | 0.372736699 | 1.345655029 | 0.045757936 | 0.241842849 | 0.204992888 |
| GOBP_DICARBOXYLIC_ACID_METABOLIC_PROCESS | GOBP_DICARBOXYLIC_ACID_METABOLIC_PROCESS | 80 | 0.390644982 | 1.401800449 | 0.045776145 | 0.241842849 | 0.204992888 |
| GOMF_CORE_PROMOTER_SEQUENCE_SPECIFIC_DNA_BINDING | GOMF_CORE_PROMOTER_SEQUENCE_SPECIFIC_DNA_BINDING | 39 | 0.469446983 | 1.431187507 | 0.045928476 | 0.242441477 | 0.205500303 |
| GOBP_REGULATION_OF_SYSTEMIC_ARTERIAL_BLOOD_PRESSURE_BY_CIRCULATORY_RENIN_ANGIOTENSIN | GOBP_REGULATION_OF_SYSTEMIC_ARTERIAL_BLOOD_PRESSURE_BY_CIRCULATORY_RENIN_ANGIOTENSIN | 16 | -0.625037792 | -1.542781248 | 0.046232877 | 0.24384114 | 0.206686697 |
| GOBP_SENSORY_PERCEPTION_OF_PAIN | GOBP_SENSORY_PERCEPTION_OF_PAIN | 61 | 0.414037423 | 1.401062674 | 0.046486202 | 0.244895546 | 0.207580441 |
| GOBP_HETEROCHROMATIN_ORGANIZATION | GOBP_HETEROCHROMATIN_ORGANIZATION | 70 | -0.435130604 | -1.401622794 | 0.046511628 | 0.244895546 | 0.207580441 |
| GOBP_HISTONE_UBIQUITINATION | GOBP_HISTONE_UBIQUITINATION | 45 | 0.455347007 | 1.430080177 | 0.046603556 | 0.2451718 | 0.207814602 |
| GOBP_KIDNEY_EPITHELIUM_DEVELOPMENT | GOBP_KIDNEY_EPITHELIUM_DEVELOPMENT | 106 | -0.387405967 | -1.340844943 | 0.046783626 | 0.245910886 | 0.208441072 |
| GOMF_EXTRACELLULAR_MATRIX_BINDING | GOMF_EXTRACELLULAR_MATRIX_BINDING | 52 | 0.428390965 | 1.39641841 | 0.04685615 | 0.245956849 | 0.208480032 |
| GOBP_CELLULAR_RESPONSE_TO_LITHIUM_ION | GOBP_CELLULAR_RESPONSE_TO_LITHIUM_ION | 11 | -0.669432177 | -1.488091348 | 0.046875 | 0.245956849 | 0.208480032 |
| GOBP_REGULATION_OF_PEPTIDE_TRANSPORT | GOBP_REGULATION_OF_PEPTIDE_TRANSPORT | 495 | 0.260616008 | 1.152745109 | 0.046911132 | 0.245956849 | 0.208480032 |
| GOBP_CELLULAR_RESPONSE_TO_CAMP | GOBP_CELLULAR_RESPONSE_TO_CAMP | 40 | 0.47233953 | 1.453522695 | 0.047055133 | 0.246430271 | 0.208881318 |
| GOBP_LIPOXYGENASE_PATHWAY | GOBP_LIPOXYGENASE_PATHWAY | 13 | -0.657610939 | -1.526173117 | 0.047120419 | 0.246430271 | 0.208881318 |
| GOBP_REGULATION_OF_HUMORAL_IMMUNE_RESPONSE_MEDIATED_BY_CIRCULATING_IMMUNOGLOBULIN | GOBP_REGULATION_OF_HUMORAL_IMMUNE_RESPONSE_MEDIATED_BY_CIRCULATING_IMMUNOGLOBULIN | 13 | -0.657423116 | -1.525737221 | 0.047120419 | 0.246430271 | 0.208881318 |
| GOBP_POSITIVE_REGULATION_OF_INTRACELLULAR_PROTEIN_TRANSPORT | GOBP_POSITIVE_REGULATION_OF_INTRACELLULAR_PROTEIN_TRANSPORT | 166 | 0.331844578 | 1.317835655 | 0.047230936 | 0.24680051 | 0.209195143 |
| GOBP_DEVELOPMENT_OF_PRIMARY_FEMALE_SEXUAL_CHARACTERISTICS | GOBP_DEVELOPMENT_OF_PRIMARY_FEMALE_SEXUAL_CHARACTERISTICS | 75 | 0.393772927 | 1.399532948 | 0.047369642 | 0.247193012 | 0.209527838 |
| GOMF_ENDOPEPTIDASE_ACTIVITY | GOMF_ENDOPEPTIDASE_ACTIVITY | 298 | 0.281488587 | 1.202623453 | 0.047385623 | 0.247193012 | 0.209527838 |
| GOCC_SPLICEOSOMAL_TRI_SNRNP_COMPLEX | GOCC_SPLICEOSOMAL_TRI_SNRNP_COMPLEX | 40 | -0.501963687 | -1.495437368 | 0.047646567 | 0.248159103 | 0.210346725 |
| GOBP_MESENCHYMAL_CELL_DIFFERENTIATION | GOBP_MESENCHYMAL_CELL_DIFFERENTIATION | 201 | 0.310906548 | 1.254372605 | 0.047650702 | 0.248159103 | 0.210346725 |
| GOMF_LIGASE_ACTIVITY | GOMF_LIGASE_ACTIVITY | 147 | 0.334883306 | 1.303083654 | 0.047763094 | 0.248536099 | 0.210666277 |
| GOBP_MONONUCLEAR_CELL_MIGRATION | GOBP_MONONUCLEAR_CELL_MIGRATION | 165 | 0.334958341 | 1.335142237 | 0.047925003 | 0.249169909 | 0.211203513 |
| GOBP_RRNA_TRANSCRIPTION | GOBP_RRNA_TRANSCRIPTION | 33 | 0.514165707 | 1.538128713 | 0.048188088 | 0.250146911 | 0.212031647 |
| GOBP_POSITIVE_REGULATION_OF_TYPE_I_INTERFERON_PRODUCTION | GOBP_POSITIVE_REGULATION_OF_TYPE_I_INTERFERON_PRODUCTION | 75 | 0.391981904 | 1.393167361 | 0.048193442 | 0.250146911 | 0.212031647 |
| GOBP_PROTEIN_MONOUBIQUITINATION | GOBP_PROTEIN_MONOUBIQUITINATION | 64 | 0.425403732 | 1.452984482 | 0.04845897 | 0.251315176 | 0.213021903 |
| GOBP_DEOXYRIBONUCLEOTIDE_METABOLIC_PROCESS | GOBP_DEOXYRIBONUCLEOTIDE_METABOLIC_PROCESS | 36 | 0.493289991 | 1.486536198 | 0.048561639 | 0.251637584 | 0.213295185 |
| GOBP_REGULATION_OF_TRANSLATIONAL_INITIATION | GOBP_REGULATION_OF_TRANSLATIONAL_INITIATION | 74 | 0.402279648 | 1.424586083 | 0.048727441 | 0.252286325 | 0.213845076 |
| GOBP_POSITIVE_REGULATION_OF_VASCULAR_ENDOTHELIAL_CELL_PROLIFERATION | GOBP_POSITIVE_REGULATION_OF_VASCULAR_ENDOTHELIAL_CELL_PROLIFERATION | 17 | -0.609598862 | -1.514937495 | 0.048986486 | 0.253357829 | 0.214753314 |
| GOBP_PYRIMIDINE_NUCLEOTIDE_METABOLIC_PROCESS | GOBP_PYRIMIDINE_NUCLEOTIDE_METABOLIC_PROCESS | 44 | 0.453380704 | 1.418689386 | 0.049052167 | 0.253357829 | 0.214753314 |
| GOBP_GRANULOCYTE_MACROPHAGE_COLONY_STIMULATING_FACTOR_PRODUCTION | GOBP_GRANULOCYTE_MACROPHAGE_COLONY_STIMULATING_FACTOR_PRODUCTION | 11 | 0.701579016 | 1.630048216 | 0.049056731 | 0.253357829 | 0.214753314 |
| GOBP_POSITIVE_REGULATION_OF_INTRINSIC_APOPTOTIC_SIGNALING_PATHWAY | GOBP_POSITIVE_REGULATION_OF_INTRINSIC_APOPTOTIC_SIGNALING_PATHWAY | 57 | 0.429234371 | 1.428994722 | 0.049151904 | 0.253638521 | 0.214991235 |
| GOBP_CANONICAL_WNT_SIGNALING_PATHWAY | GOBP_CANONICAL_WNT_SIGNALING_PATHWAY | 297 | 0.287506721 | 1.22770805 | 0.049376338 | 0.254585217 | 0.215793683 |
| GOBP_POSITIVE_REGULATION_OF_T_CELL_MIGRATION | GOBP_POSITIVE_REGULATION_OF_T_CELL_MIGRATION | 29 | 0.531004566 | 1.529467774 | 0.049610621 | 0.255324693 | 0.216420484 |
| GOBP_ORGANELLE_FUSION | GOBP_ORGANELLE_FUSION | 135 | 0.344077372 | 1.324906689 | 0.049643044 | 0.255324693 | 0.216420484 |
| GOMF_EXTRACELLULAR_MATRIX_STRUCTURAL_CONSTITUENT | GOMF_EXTRACELLULAR_MATRIX_STRUCTURAL_CONSTITUENT | 137 | 0.346739571 | 1.339404553 | 0.049643044 | 0.255324693 | 0.216420484 |
| GOBP_CELLULAR_RESPIRATION | GOBP_CELLULAR_RESPIRATION | 177 | 0.318613264 | 1.273985889 | 0.049781253 | 0.255823758 | 0.216843505 |
| GOBP_REGULATION_OF_CARTILAGE_DEVELOPMENT | GOBP_REGULATION_OF_CARTILAGE_DEVELOPMENT | 55 | 0.441022982 | 1.467206865 | 0.049845464 | 0.255942039 | 0.216943763 |
