# Supplementary material for: A New Prognostic Indicator of Immune Microenvironment and Therapeutic Response in Lung Adenocarcinoma Based on Peroxisome-Related Genes
Source: J Immunol Res. 2022 Jul 26;2022:6084589. doi: 10.1155/2022/6084589 (PMC9346542; doi:10.1155/2022/6084589)
Supplement: Supplementary 5 — Table S4: drugs targeting signature genes. [file 6084589.f5.docx]

Table S4 Drugs targeting signature genes.

| Gene | Drug | cor | pvalue |
| --- | --- | --- | --- |
| ABCD1 | Vemurafenib | 0.550395585 | 5.22E-06 |
| ACSL3 | ARRY-162 | 0.482148078 | 9.59E-05 |
| ACSL3 | Cobimetinib (isomer 1) | 0.479463012 | 0.000106193 |
| ACSL1 | Dabrafenib | 0.476279494 | 0.00011976 |
| ACSL3 | Trametinib | 0.449816061 | 0.000311257 |
| ABCD1 | Dabrafenib | 0.446762793 | 0.000345816 |
| ACSL3 | Dabrafenib | 0.4439129 | 0.000381189 |
| ACSL3 | brigatinib | -0.436704258 | 0.000485838 |
| ACSL1 | Vemurafenib | 0.434004883 | 0.000531309 |
| ABCD1 | Encorafenib | 0.430748614 | 0.00059128 |
| ACSL3 | Selumetinib | 0.428547196 | 0.000635231 |
| ACSL3 | Vemurafenib | 0.421213489 | 0.00080383 |
| ACSL1 | Asparaginase | -0.414199683 | 0.001001871 |
| ACSL3 | Everolimus | -0.40223201 | 0.001443246 |
| CAT | Cyclophosphamide | 0.39633675 | 0.001719158 |
| ACSL3 | Nelfinavir | -0.38451494 | 0.002418681 |
| ACSL1 | Cobimetinib (isomer 1) | 0.381052296 | 0.002666809 |
| CAT | Hydroxyurea | 0.380067544 | 0.002741393 |
| LDHA | 6-Thioguanine | 0.379779179 | 0.002763582 |
| ABCD1 | Dasatinib | -0.375757604 | 0.003090112 |
| ACSL1 | Encorafenib | 0.372455499 | 0.003383374 |
| ACSL1 | Selumetinib | 0.368139772 | 0.003803721 |
| ACSL3 | MITOXANTRONE | -0.366547146 | 0.003970137 |
| ACSL1 | DIGOXIN | -0.366108963 | 0.004017039 |
| CAT | ABT-199 | 0.358435414 | 0.004921622 |
| ABCD1 | Selumetinib | 0.352271384 | 0.005773595 |
| LDHA | 6-THIOGUANINE | 0.347263878 | 0.006558406 |
| ACSL3 | Encorafenib | 0.339708415 | 0.007919255 |
| ACSL3 | JNJ-42756493 | -0.338827781 | 0.008092867 |
| ACSL1 | ARRY-162 | 0.338060449 | 0.008246838 |
| ACSL3 | Dasatinib | -0.329484731 | 0.010149039 |
| ACSL1 | Dexrazoxane | -0.326831216 | 0.010809917 |
| ACAT1 | Nandrolone phenpropionate | 0.325461371 | 0.011165436 |
| ABCD1 | Bosutinib | -0.322772485 | 0.011892779 |
| CAT | Vorinostat | 0.322674699 | 0.011919984 |
| ACSL1 | MITOXANTRONE | -0.322512196 | 0.011965312 |
| ACSL3 | Docetaxel | -0.318369465 | 0.01317219 |
| CAT | Everolimus | -0.317740771 | 0.013364209 |
| ACSL1 | Ifosfamide | -0.317650877 | 0.01339186 |
| ACAT1 | Carmustine | 0.31659725 | 0.013719648 |
| ABCD1 | ARRY-162 | 0.315470525 | 0.014077789 |
| ACSL3 | Alectinib | -0.314980054 | 0.014236183 |
| ACAT1 | Ifosfamide | 0.314041151 | 0.014543667 |
| ACSL3 | BMN-673 | -0.313116753 | 0.014851954 |
| ACSL1 | Trametinib | 0.312292965 | 0.015131396 |
| ACSL1 | Mitoxantrone | -0.309455104 | 0.016128831 |
| CAT | Nilotinib | 0.305541343 | 0.017596378 |
| ACSL1 | 6-THIOGUANINE | -0.304496102 | 0.018007074 |
| ACAT1 | Lomustine | 0.303423535 | 0.018436977 |
| ACAT1 | Hydroxyurea | 0.303327382 | 0.018475941 |
| LDHA | Trametinib | 0.299108841 | 0.020255859 |
| CAT | Olaparib | -0.29874138 | 0.020417576 |
| ABCD1 | Pipamperone | 0.298044027 | 0.02072749 |
| ACSL3 | Axitinib | -0.295557282 | 0.021865352 |
| ACSL1 | ABT-199 | 0.294678267 | 0.022280024 |
| ACSL1 | BMN-673 | -0.291910022 | 0.0236297 |
| LDHA | Pazopanib | 0.290292072 | 0.024450064 |
| ACSL1 | Acalabrutinib | 0.288353679 | 0.025464426 |
| ACSL1 | Docetaxel | -0.285945062 | 0.026774043 |
| CAT | Sulfatinib | 0.282187353 | 0.028930056 |
| ABCD1 | Palbociclib | -0.279455904 | 0.030587018 |
| CAT | LEE-011 | 0.27931732 | 0.030673159 |
| ACSL1 | DAUNORUBICIN | -0.278382629 | 0.031259459 |
| ACAT1 | Everolimus | -0.278165238 | 0.031397155 |
| ABCD1 | Neratinib | -0.277099463 | 0.032079571 |
| ACSL1 | Oxaliplatin | -0.27678623 | 0.032282472 |
| ACSL3 | Vorinostat | 0.276602129 | 0.032402225 |
| ABCD1 | umbralisib | -0.276526395 | 0.032451595 |
| LDHA | ABT-199 | 0.275353315 | 0.033224367 |
| CAT | Palbociclib | 0.273187388 | 0.034691481 |
| ABCD1 | brigatinib | -0.272610175 | 0.035091429 |
| ACSL1 | brigatinib | -0.27197339 | 0.035537095 |
| ACSL1 | Abiraterone | 0.271767858 | 0.035681939 |
| ACSL3 | Mitoxantrone | -0.270899823 | 0.036299091 |
| ACSL3 | Teniposide | -0.267226317 | 0.039009766 |
| ACSL1 | Sunitinib | 0.266756643 | 0.039368106 |
| ACSL1 | Valrubicin | -0.266030233 | 0.039927681 |
| LDHA | Doxorubicin | -0.265642638 | 0.040228935 |
| ACSL3 | Nitrogen mustard | -0.265487823 | 0.040349786 |
| LDHA | Epirubicin | -0.264585137 | 0.041060425 |
| CAT | Calusterone | 0.26276577 | 0.042524113 |
| ABCD1 | Dacomitinib | -0.26264549 | 0.042622374 |
| ACSL1 | Teniposide | -0.262452045 | 0.042780798 |
| ACSL3 | Oxaliplatin | -0.262271855 | 0.042928803 |
| ACSL1 | Etoposide | -0.261984605 | 0.043165615 |
| ACSL3 | umbralisib | -0.261697772 | 0.043403156 |
| LDHA | Dabrafenib | 0.261315173 | 0.043721678 |
| ACAT1 | JNJ-42756493 | -0.261263028 | 0.043765238 |
| LDHA | Allopurinol | 0.260028732 | 0.044806796 |
| CAT | Crizotinib | 0.259397881 | 0.045346949 |
| ACSL1 | Epirubicin | -0.258392986 | 0.046218398 |
| ACSL1 | umbralisib | -0.25624189 | 0.048130033 |
| ACAT1 | ETHINYL ESTRADIOL | 0.25540731 | 0.048888911 |
| LDHA | Encorafenib | 0.255288899 | 0.04899737 |
| ACSL1 | Dasatinib | -0.254983195 | 0.049278289 |
| ACAT1 | Parthenolide | 0.254770837 | 0.049474203 |
| ACSL3 | 6-Mercaptopurine | -0.254316597 | 0.049895407 |
| ABCD1 | Cobimetinib (isomer 1) | 0.254302878 | 0.049908173 |
